# 2024 Annual Congress of the Pulmonary Vascular Research Institute

**DOI:** 10.1002/pul2.12376

**Published:** 2024-05-15

## A001 PULMONARY RESISTANCE INDEX FOR RISK STRATIFICATION IN PATIENTS WITH LOW‐FLOW, LOW‐GRADIENT AORTIC STENOSIS

Yassine Abdeldjebbar1; Sébastien Hecht1; Kathia Abdoun1; Mohamed Salah Annabi1; Mathieu Bernier1; Jonathan Beaudoin1; Nancy Côté1; Kim O'Connor1; Josep Rodes‐Cabau1; Eric Larose1; Patrick Mathieu1; Marie‐Annick Clavel1; Abdellaziz Dahou1; Philippe Pibarot1

1Centre de recherche de l'Institut universitaire de cardiologie et de pneumologie de Québec, Université Laval (IUCPQ‐ULaval), Québec, Canada

Previous studies have demonstrated that patients with aortic stenosis (AS) and pulmonary hypertension have a poorer prognosis. Patients with low‐flow, low‐gradient aortic stenosis (LFLG AS) and reduced left ventricular ejection fraction (LVEF) are known to have a poor prognosis following transcatheter aortic valve implantation (TAVI). However, the prognostic value of the pulmonary artery systolic pressure (PASP) to stroke volume (SV) ratio in patients with LF‐LG AS has not been investigated. This study aims to evaluate the prognostic value of the PASP/SV ratio in patients with LF‐LG AS treated conservatively or with aortic valve replacement (AVR). The objective of this study was to determine the prognostic value of the PASP/SV ratio in LFLG‐AS patients. Clinical and echocardiographic data were prospectively collected from 381 patients enrolled in the TOPAS (Truly or Pseudo‐severe Aortic Stenosis #NCT01835028) study. The cohort was divided into two groups based on a cutoff value of PASP/SV 0.54 mmHg/mL, which showed the optimal prognostic value for predicting 5‐year outcomes using receiver operating characteristic (ROC) analysis. The mean age of the study population was 73 years, and 66% were male. During a median follow‐up of 1.5 (0.6–4.2) years, 143 (37%) patients died. Kaplan–Meier survival curve analysis revealed that patients with a PASP/SV < 0.54 mmHg/mL vs PASP/SV > 0.54 mmHg/mL had a 5‐year survival estimate of 82% and 52%, respectively (log‐rank *p* < 0.001). These findings were confirmed through multivariable Cox analysis adjusted for age, sex, BMI, AVR as a time‐dependent variable, diabetes, and STS score. Patients with a PASP/SV > 0.54 mmHg/mL had a significantly increased risk of all‐cause mortality at 5 years compared to those with a PASP/SV < 0.54 mmHg/mL (HR [95% CI] = 2.17 [1.36–3.46], *p* = 0.001). The pulmonary resistance index defined as the PASP/SV ratio is independently associated with increased risk of all‐cause mortality in patients with LF‐LG AS. The PASP/SV ratio may be useful to enhance risk stratification in this challenging population.

## A002 PULMONARY ARTERIAL HYPERTENSION DUE TO TOXIC OIL SYNDROME THROUGH TIME

Md Álvaro Aceña Navarro^1^, Alejandro Cruz^1^, Irene Martín‐De Miguel^1^, Natalia Calvente‐Vera^2^, Teresa Segura‐De La Cal^1^, Alejandro Cruz‐Utrilla^1,3^, Fernando Arribas‐Ynsaurriaga^1,3^, María Pilar Escribano‐Subias^1,3^

^1^Department of Cardiology. Hospital Universitario 12 de Octubre, Madrid, Spain, ^2^Department of Pneumology. Hospital Universitario San Cecilio, Granada, Spain, ^3^Centro de Investigación Biomédica en Red de Enfermedades Cardiovasculares (CIBERCV), Madrid, Spain

Toxic oil syndrome (TOS) is a systemic disease that developed in Spain due to the consumption of rapeseed oil denatured with aniline in 1981, which triggered a health crisis in the country. This disease is characterized by marked eosinophilia, myalgias, and pulmonary infiltrates. A manifestation in both acute and chronic phases is pulmonary arterial hypertension (PAH), secondary to non‐necrotizing endothelial damage in the pulmonary circulation. In this study, we analyzed patient characteristics according to when the diagnosis of TOS was made. Retrospective observational study including patients with PAH secondary to TOS from 1992 to 2018, excluding the acute phase of the disease. Demographic, clinical, functional, and hemodynamic variables were collected, and the sample was divided into two groups, before and after 2001 (median time of the cohort collected) into Groups 1 and 2, respectively, for comparison. Fifty‐six patients were included, 33 diagnosed before 2001 and 23 thereafter, with a predominance of women (62.5%). In group 1 median age at diagnosis was 31, in WHO functional class (FC) III (72%) and IV (28%), with DLCO 67.2% and the mean distance in 6‐min walking test (6MWT) of 342 m. On the other hand, Group 2, showed a median age of 48, FC II (41%) and III (46%), DLCO 59.3%, and distance in 6MWT of 416 m. Differences in age and FC are significant (*p*‐value < 0.001). Debut is similar in both groups, but eosinophilia is more frequent in group 1 (95.2% vs 72.7%), being statistically significant (*p*‐value 0.046). Hemodynamically, in means, the first group presents right atrial pressure (RAP) 12 mmHg, mean pulmonary artery pressure (PAP) 71.1 mmHg (±2.1DS), pulmonary capillary wedge pressure (PCWP) 11 mmHg, cardiac output (CO) 3 L/min, and pulmonary vascular resistance (PVR) 19.5WU. Group 2, on the other hand, shows RAP 6 mmHg, mean PAP 50 mmHg (±3.7DS), PCWP 9 mmHg, CO 4.2 L/min, and PVR 9.4WU. All these variables are significant, except for PCWP. In Group 1, only three patients (9.1%) were treated sequentially, the rest (50%) receiving monotherapy with systemic prostacyclins; in contrast to Group 2, in which 15 (68.2%) received sequential combination treatment, and 4 (21.1%) received monotherapy with systemic prostacyclins, statistically significant. Of Group 1, 4 patients (12.1%) were transplanted and 25 (75.8%) died, unlike the second group, in which only 1 (4.6%) was transplanted and 10 (45.5%) died. The Kaplan–Meier curve showed no significant differences, with a tendency towards better survival in Group 2. Patients with PAH diagnosed before 2001 presented greater severity at diagnosis, with worse functional class and greater haemodynamic severity. In addition, there is more use of systemic prostacyclins in monotherapy and a worse evolution, with higher mortality. All this is probably directly related to the historical moment, when the diagnosis of PAH was much later, in addition to the lack of the current therapeutic arsenal, with a very high subsequent mortality.

## A003 CLOSURE OF SIMPLE CONGENITAL DEFECTS WITH SEVERE PULMONARY VASCULAR DISEASE: MEDIUM‐TERM FOLLOW‐UP AT A NATIONAL REFERRAL HOSPITAL

Oscar Aguirre‐Zurita^1^, Victor Robles‐Velarde^2^, Jose Tapia‐Leonardo^3^, Ruth Villarroel^4^, Jose Ercilla‐Sanchez^5^

^1^Pulmonary hypertension & Congenital heart disease Unit‐INCOR, Lima/Jesus Maria, Peru, ^2^Cardiothoracic surgery Department‐ INCOR, Lima/Jesus Maria, Peru, ^3^Cardiothoracic postoperative care‐ INCOR, Lima/Jesus Maria, Peru, ^4^Cardiovascular Anesthesiology Department‐ INCOR, Lima/Jesus Maria, Peru, ^5^Interventional Cardiology Department‐ INCOR, Lima/Jesus Maria, Peru

Congenital heart defects (CHDs) are the most common type of birth defects. In South America, two new cases are born every 15 min, and 25% of them require intervention within the first year of life. Unfortunately, only 8% receive timely intervention due to delayed diagnosis. Some CHD patients can reach adulthood and can be classified into those who underwent interventions during childhood (90%) and those who were recently diagnosed (10%). The latter group often experiences complications such as heart failure, arrhythmias, or pulmonary hypertension (PH). The 2020 guidelines for adults with CHDs contraindicate defect closure if the Pulmonary Vascular Resistance (PVR) exceeds 5WU. The aim of this study is to assess whether partial defect closure can improve hemodynamics and functional class in the medium term. We conducted a prospective study from 2016 to the present, involving patients aged >15 years old with simple CHDs, PH, and PVR 5–8 WU measured by right heart catheterization (RHC). Patients with >1 shunt, moderate/high complexity CHDs, Eisenmenger syndrome, severe left‐sided valvulopathy, and obstructive or restrictive lung disease were excluded. Surgery involved repairing the atrial septal defect (ASD) with a patch, leaving a 10 mm diameter fenestration, and closing the patent ductus arteriosus (PDA) by in situ thrill palpation. RHC was performed after pharmacological therapy, and after partial defect closure. Hemodynamic values were presented as medians and compared using the Wilcoxon test. Among 583 patients with CHDs, age 42.4 ± 17 years, 72% female, functional class (FC): 2.4 ± 1.9. ASD: 77%, VSD 5% and PDA 18%. 472 patients met the criteria for PH, pre‐capillary PH: 18%, and 110 patients (19%) met combined pre/post‐capillary PH criteria. Among them, 11 patients were included: seven ASD and four PDA patients. Eight out of 11 patients had FC > 3, and the average distance covered in the 6‐min walk test (6MWT) was 306 m. The average PAPm was 57 mmHg, RA pressure was 10 mmHg, wedge pressure was 16 mmHg, PVR 6 WU, and PVR/SVR ratio was 0.28. Surgery was successful in all patients, RHC was performed in 7 of 10 patients after an average of 18.8 months, resulting in a 37% reduction in PAPm, 31% reduction in wedge pressure, 34% reduction in PVR (average 3.8 WU), and a 20% reduction in PVR/SVR ratio, Wilcoxon test, *p* = 0.009. Follow‐up was 42 ± 21.2 months. One out of 10 patients remained in FC 3, and the 6MWT distance increased to 421 m. A high prevalence of PAH was observed in this cohort (18%), three times the international estimated value (6%). This, along with the elevated rate of combined pre/post‐capillary PH, reflects the advanced stage of the disease at diagnosis. Current guidelines contraindicate surgery in this patient group. However, individualized selection and multidisciplinary management achieved a reduction in PAP and PVR in the medium term, resulting in an improved functional and hemodynamic profile. Early diagnosis is always the goal, but in the practical context of our country, the “treat and repair” approach may be an individualized decision to consider at referral centers.

## A004 CD4+ LYMPHOCYTES EXPRESSING HUMAN CHAT AS A NOVEL VASODILATOR

Mohamed Ahmed^1^

^1^Diamond Children Hospital, University of Arizona, Tucson, Arizona, USA

Recently, we have shown that CD4+ ChAT+ T cells have a vasodilatory effect, once infused systemically, in adult mice evident by an instant drop of mean blood pressure. ChAT+ T cells represent only 1.1% of total circulating T lymphocytes. Transgenic mice with CD4 TChAT−/− were studied and found to have a significantly elevated arterial blood pressure and peripheral systemic resistance as compared to wild type mice (CD4 TChAT+/+). To study the effect of CD4+ChAT+ Cells on animal model with PAH and to delineate the vasodilatory nature of the CHAT protein response in HPACEs. Mice housed in hypoxia (10% O_2_ for 3 weeks) to induce pulmonary arterial hypertension (PAH), were treated with different amounts of JTChAT and the vasodilator effect was monitored. In vitro, primary human pulmonary endothelial cells were exposed to hypoxia (FiO_2_ 1% for 48 h), to study the interaction between CHAT protein and many molecular pathway regulators of vasomotor and blood pressure. In animals with PAH, right ventricular pressure (RVP), was not changed upon JTCHAT‐ve cells infusion, while infusion of ChAT+ve cells (between 0.5 and 2.0 million cells/animal), induced a significant reduction in RVP (up to 50%), within 2–3 min post injection, and this effect was sustained until the animal was euthanized (30–40 min). The vasodilator effect was dose‐dependent, and we found that a dose ranging between 0.5 and 2 M cells count, was able to achieve the most effective reduction of RVP (at least 50%). ECHO studies and molecular studies were also done and showed a significant improvement of pulmonary pressure among treated groups with JCHAT or primary mouse CD4+hChat T cell. Co‐culture of JTChAT cells with endothelial cells, showed a significant increases in intracellular calcium, phosphorylated eNOS expression, and accumulation of nitrates/nitrites in the conditioned media, indicating increased NO release. Co‐culturing endothelial cells with a single specific cholinergic receptor (M1, M2, M3, & 7Sma) before adding JTChAT cells, was examined to define the specific target receptor for JTCHAT cells. Specific M1 blockade induced the most significant downregulation of eNOS expression upon the interaction between endothelial cells and JTChAT cells. Human pulmonary arterial endothelial cells were cultured in the presence or absence of either CD4+hCHAT T cells or CD4+ T cells. RNA sequencing studies showed a unique vasodilatory genes signature isolated (GPX1, PLOD3, SOD1, ADORA2B, TRPV4, F2RL1, KLF2, GRK2, EXT1, and PTPRM). JTChAT cells have a specific pulmonary vasodilator by augmenting NO release and interact with endothelial cells via regulation of many blood pressure and vasomotor gene regulators.

## A005 KLF6—A NEW REGULATOR OF ENDOTHELIAL FUNCTION IN PULMONARY ARTERIAL HYPERTENSION

Rehab Alharbi^1^, Nadia Fernandez^2^, Richard D Williams^2^, Chien‐Nien Chen^1^, Lan Zhao^1^, Natalie Lambie^2^, May Al‐Sahaf^1,3^, Nik Matthews^2^, Inês Cebola^2^, Beata Wojciak‐Stothard^1^

^1^National Heart and Lung Institute, Imperial College London, London, UK, ^2^Department of Metabolism, Digestion and Reproduction, Imperial College London, London, UK, ^3^Department of Thoracic Surgery, Hammersmith Hospital, London, UK

Loss of endothelial homeostasis is a key contributor to vascular remodelling in PAH. PAH shares many characteristics with cancer, including apoptosis resistance, increased cell proliferation, and angiogenesis. Accumulating evidence suggests that Krüppel‐like factor 6 (KLF6), a transcription factor implicated in the progression of cancer, may play a contributory role. (1) To study KLF6 expression in PAH; (2) to characterise KLF6 effects on endothelial transcriptome and function; (3) to compare KLF6 with KLF2 and KLF4, the two closely related transcription factors implicated in PAH. KLF6 mRNA levels were reduced in human PAH, MCT and Sugen/Hypoxia rats but were elevated in the blood‐derived late outgrowth endothelial colony forming cells (ECFCs) from PAH patients with BMPR2 mutations (*n* = 4–5). A transient increase in KLF6 expression was noted in early disease. KLF6 expression in HPAECs was induced by physiological flow and was markedly upregulated under a “double hit” condition of hypoxia combined with TNF‐α, whereas expressions of KLF2 and KLF4 were markedly reduced. RNA‐seq analysis of HPAECs overexpressing KLF2, 4 and 6 showed that KLF6, in contrast to KLF2 and KLF4, activates proangiogenic signalling and increases expression of genes regulating endothelial identity and survival including BMPR2, Erg, SOX17, CDH5 and KDR. Functional studies confirmed that, in addition to the antiproliferative, anti‐inflammatory, antiapoptotic and barrier‐protective effects, KLF6 overexpression induces endothelial angiogenesis, measured in the tube formation assay and the human pulmonary artery explant sprouting assay in vitro. The proangiogenic effects of KLF6 can be attributed to increased endothelial cell migration but not to endothelial cell proliferation. The loss‐of‐function experiments showed that siRNA‐mediated knockdown of KLF6 in HPAECs cultured under flow inhibits endothelial cell alignment, increases NFkB activity, induces endothelial apoptosis and reduces endothelial repair studied in a wound healing assay. Spatial transcriptomic analysis of human PAH lungs (*n* = 6/group of sex‐ and age‐matched PAH and controls) (GeoMx, Nanostring) showed increase in the expression of KLF6 regulated genes in the remodelled vessels, particularly in plexiform lesions. This study is first to demonstrate dysregulation of KLF6 signalling in PAH. KLF6 triggers endothelial repair in response to stress conditions induced by hypoxia and inflammation. Its role in PAH will require further studies but its is likely that KLF6 de‐activation following an early rise in its activity, may increase endothelial susceptibility to damage and dysregulate endothelial proliferation and angiogenesis.

## A006 ASSESSMENT OF CIRCULATORY CELL‐FREE DNA BIOGENESIS PATHWAYS IN HIGH ALTITUDE PULMONARY EDEMA

Manzoor Ali^1,2^, Kanika Singh^1^, Raushni Choudhary^1,2^, Aastha Mishra^1,2^

^1^Cardio Respiratory Disease Unit, CSIR‐Institute of Genomics and Integrative Biology, Delhi, India; ^2^Academy of Scientific and Innovative Research (AcSIR), Ghaziabad, India

High altitude (HA, altitude >2500 m above sea level) results in a maladaptive response such as high‐altitude pulmonary edema (HAPE) in a few sojourners triggered by individual susceptibilities and the speed of ascent. Extensive research into genetic and nongenetic markers has been conducted in HAPE but no study focused on circulating cell‐free (cf) DNA fragments, which are related to the nucleosomal organization, gene expression, and nuclease content of the tissue of origin. The utility of cfDNA has extended as a clinical biomarker in various diseases and its investigation in HAPE pathophysiology has the potential to provide valuable insights into the body's response to hypoxia and altitude‐related stress. The present study conducted a comparative analysis of cfDNA levels in the two groups, HAPE patients (*n* = 112) and healthy controls (*n* = 111). HAPE patients were further segregated into mild (*n* = 14), moderate (*n* = 24) and severe (*n* = 34). Our findings revealed that cfDNA levels as determined by the fluorometry and bioanalyzer‐based tests found significantly higher cfDNA concentration in ng/mL in HAPE, 25.99 ± 1.53, compared to controls, 14.92 ± 0.72 (mean ± SEM, (*p*  < 0.0001). The observed plasma cfDNA concentration differed significantly within the three categories of HAPE, exhibiting 1.3‐ and 1.8‐fold higher concentrations for the moderate and severe categories (*p* = 0.0036) of HAPE patients compared to the mild cases. Furthermore, the cfDNA population was characterized based on its fragment length in base pairs (bp) and their respective concentrations for each sample. Additionally, we examined the degree of cfDNA integrity that exhibited a lower DNA integrity index of 0.346 in HAPE patients (*p* = 0.001) indicating the presence of shorter DNA fragments circulating in their bloodstream compared to the healthy control group. The biological mechanisms underlying the increased release of cfDNA from cells remain complex and multifaceted. It is widely recognized that cfDNA can be released from cells through two distinct processes: cell death, which occurs via apoptosis or necrosis, and active secretions. To recognize the main pathways of cfDNA release, we quantitatively measured the cytokeratin 18 (CK18) protein in the plasma samples. The plasma membrane integrity is altered during cell death due to which CK18 is released in the extracellular compartment. When the full length of soluble CK18 is released, it signifies cell death through necrosis or apoptosis whereas, in the case of apoptosis, a caspase cleaved CK18 is released in the circulation. Thus, the difference between full‐length soluble CK18 and caspase cleaved CK18 highlights secondary necrosis or late apoptosis. In our study, we observed a significant increase in the concentration of full‐length soluble CK18 and caspase‐cleaved CK18 in HAPE compared to controls (*p* < 0.001). Our findings revealed a significant distinction and increase in both the markers in mild, moderate, and severe HAPE patient groups (*p* < 0.001). However, these results need validation in a larger sample size with additional and specific markers of cell death pathways such as apoptosis, secondary necrosis, and late apoptotic pathways. Additionally, the status and contribution of active secretion towards cfDNA release in circulation under the HA setting should also be tested.

## A007 IMPORTANCE OF PULMONARY VASCULAR ANGIOGRAPHY IN PEDIATRIC PATIENTS WITH TBX4

Maria Alvarez‐fuente^1^, Ignacio Hernandez‐Gonzalez, Elvira Garrido‐Lestache, Itziar Garcia‐Ormazabal, Maria Jesus Del Cerro

^1^Hospital Universitario Ramón Y Cajal, Madrid, Spain

T‐box transcription factor 4 (TBX4) is involved in the regulation of embryonic developmental processes. Pathogenic variants in TBX4 are a well‐established cause of PAH in children, typically associated with skeletal disorders (small patella syndrome), intellectual disabilities and other cardiovascular disorders. Mutations in this gene might also lead to diffuse developmental disorders in the lungs in neonates. We present three pediatric patients with TBX4 mutation. Patient 1 was diagnosed at birth and required treatment with tadalafil, macitentan and subcutaneous treprostinil. Treprostinil was withdrawn after 2 years of treatment. At 3 years of age the pulmonary pressures had decreased with two drugs: PAP 48/22/30 mmHg, PVR 3.33 UW*m^2^ and CI 5.1 L/min/m^2^. Patient 2 was diagnosed at birth requiring treatment with sildenafil during 3 years with normalisation of the pulmonary pressures with treatment withdrawal. At adolescence the echo showed rectification of the interventricular septum and in the catheterization the patient had: PAP 36/16/26 mmHg, PVR 3.2 UW*m2, CI 5 L/min/m^2^. Patient 3 was diagnosed at 4 moths of age. After treatment with sildenafil and bosentan for 4 years catheterization was repeated were a positive response to vasoreactivity test was observed. After initiating treatment with amlodipine, sildenafil was interrupted and a normalisation of the pressures was observed with amlodipine and bosentan: 24/12/18, PVR 3.1 UW*m^2^, CI 5.6 L/min/m^2^. All three patients had a tendency to pulmonary pressure normalisation with vasodilator therapy, and without drugs in one patients. Despite the reduction in pulmonary pressures, the pulmonary angiographies show vascular pruning because of an abnormal pulmonary vascular development. Patient two, the oldest of the three, had a recurrence of pulmonary hypertension at adolescence, after maintaining normal pulmonary pressures for 9 years. The vascular underdevelopment in pediatric patients with pulmonary hypertension and TBX4 mutations can play an important role in the evaluation of the patients' prognosis. Therefore, to evaluate vascular development, a pulmonary vascular angiography should be performed in these patients during a cardiac catheterization.

## A008 ADMINISTRATION OF KER‐012 TO HEALTHY POST‐MENOPAUSAL WOMEN ELICITED BIOMARKER CHANGES SUGGESTING ANTI‐INFLAMMATORY AND ANTI‐FIBROTIC EFFECTS THAT TARGET THE PATHOPHYSIOLOGY OF PULMONARY ARTERIAL HYPERTENSION

Harveen Natarajan^1^, Lorena Lerner^1^, Keith Babbs^1^, Martin Fisher^1^, Chris Materna^1^, Sylvain Bedard^1^, Jay Russak^1^, Kathryn MK Steiner^1^, Dena Grayson^1^, Simon Cooper^1^

^1^Keros Therapeutics, Lexington, USA

Dysregulated TGF‐β signaling, largely via increased pro‐proliferative activin signaling coupled with reduced anti‐proliferative bone morphogenetic protein (BMP) signaling, is believed to drive cellular proliferation, inflammation and immune dysregulation leading to obliterative remodeling and increased pulmonary vascular resistance in PAH. KER‐012, an investigational modified activin receptor type IIB (ActRIIB) ligand trap, is designed to specifically bind and inhibit select TGF‐β ligands including activins A and B and growth differentiation factors 8 and 11 while simultaneously permitting BMP signaling. Restoring homeostasis in these pro‐ and anti‐proliferative signaling pathways has the potential to halt or even reverse the pathophysiology of PAH while avoiding erythropoietic effects, maintaining vascular integrity, and reducing bleeding risk. In preclinical models of PAH, a research version of KER‐012 (RKER‐012) prevented increased pulmonary arterial pressure (PAP), lung inflammation and fibrosis. Furthermore, RKER‐012 prevented right ventricular hypertrophy, potentially both indirectly by reducing PAP and via direct effects on the myocardium. In a Phase 1 trial in healthy post‐menopausal women, KER‐012 was generally well tolerated at multiple doses up to 4.5 mg/kg. An exploratory post‐hoc analysis to determine if the administration of KER‐012 in this trial elicited changes in the expression of proteins that would be considered beneficial in the context of PAH is presented here. This Phase 1 trial assessed the safety, tolerability, pharmacokinetics, and pharmacodynamics of KER‐012 (0.75–5.0 mg/kg administered as single or multiple ascending doses) in healthy post‐menopausal women. Serum samples collected pre‐dose and post‐dose up to 28 days after a single dose of KER‐012 at 4.5 mg/kg or placebo were evaluated in a post‐hoc proteomic analysis using the SomaScan platform that analyzes the expression of ~7000 proteins including markers of inflammation and fibrosis associated with the pathophysiology of PAH. Serum N‐terminal pro‐brain natriuretic peptide (NT‐proBNP), a biomarker of cardiac dysfunction, was also assessed by ELISA. KER‐012 was generally well tolerated in this Phase 1 trial. Proteomic analyses revealed altered expression of 81 genes (63 upregulated, 18 downregulated) 28 days after a single 4.5 mg/kg dose of KER‐012 compared to placebo controls. Reductions in markers of fibrosis, collagens (types II and III) and matrix metalloproteinases (7 and 10), and inflammatory cytokines (IL‐6 and ‐11) were observed, accompanied by increases in anti‐inflammatory cytokines (IL‐4 and ‐35) and markers of macrophage polarization (MARCO and sCD163). Furthermore, reductions in serum levels of NT‐proBNP, as assessed by SomaScan and ELISA, were observed starting at 7 days after KER‐012 administration. Administration of a single 4.5 mg/kg dose of KER‐012 resulted in altered expression of serum biomarkers that may potentially be beneficial in the context of PAH, including markers of inflammation, fibrosis, macrophage polarization, and cardiac dysfunction. These findings, along with the favorable safety and tolerability profile observed in the Phase 1 trial, provided rationale for the ongoing Phase 2 TROPOS trial of KER‐012 in patients with PAH (NCT05975905).

## A009 DEVELOPMENT OF A NOVEL INVESTIGATIONAL ACTIVIN RECEPTOR TYPE IIB LIGAND TRAP WITH HIGH ACTIVIN/GDF SPECIFICITY AND TARGET ENGAGEMENT FOR THE TREATMENT OF PULMONARY ARTERIAL HYPERTENSION: RATIONALE AND DESIGN OF THE TROPOS PHASE 2 STUDY

Marc Humbert^1^, Raymond Benza^2^, Hossein Ardeschir Ghofrani^3,4^, Deputy Director Department of Respiratory Medicine Marius Hoeper^5^, Vallerie McLaughlin^6^, Ioana R Preston^7^, Sandeep Sahay^8^, Rogerio Souza^9^, Jay Russak^10^, Ying Jiang^10^, Harveen Natarajan^10^, Ajit Chavan^10^, Sylvain Bedard^10^, Kathryn MK Steiner^10^, Dena Grayson^10^, Simon Cooper^10^, Mardi Gomberg‐Maitland^11^

^1^Universite Paris‐Saclay, Paris, France, ^2^Ichan School of Medicine at Mount Sinai, New York, USA, ^3^Kerckhoff Heart, Rheuma and Thoracic Center, Bad Nauheim, Germany, ^4^Universities of Giessen and Marburg Lung Center, Giessen, Germany, ^5^Hannover Medical School, Hannover, Germany, ^6^University of Michigan Medical School, Ann Arbor, USA, ^7^Tufts University School of Medicine, Boston, USA, ^8^Houston Methodist Hospital, Houston, USA, ^9^ Universidade de Sao Paulo, Sao Paulo, Brazil, ^10^Keros Therapeutics, Lexington, USA, ^11^George Washington University School of Medicine and Health Sciences, Washington, USA

Accumulating evidence suggests dysregulated transforming growth factor‐β (TGF‐β) superfamily signaling has an important role in PAH pathophysiology. Pro‐proliferative signaling via activin receptors is increased, and anti‐proliferative signaling via bone morphogenetic protein (BMP) type 2 receptors is reduced, together promoting endothelial dysfunction and hyperproliferation of pulmonary arterial smooth muscle cells. Rebalancing these opposing signaling pathways represents an attractive approach to potentially halt or reverse this vascular remodeling. KER‐012, an investigational modified activin receptor type IIB (ActRIIB) ligand trap, was designed to bind and inhibit select TGF‐β ligands such as activins A and B and growth differentiation factors (GDFs) 8 and 11, while permitting BMP signaling in pulmonary artery endothelial and smooth muscle cells. In preclinical PAH models, a research version of KER‐012 (RKER‐012) prevented increased pulmonary arterial pressure (PAP), lung inflammation and fibrosis. Further, RKER‐012 prevented right ventricular hypertrophy, potentially both indirectly by reducing PAP and via direct effects on the myocardium. Safety, tolerability, pharmacokinetics (PK) and pharmacodynamics (PD) of KER‐012 were evaluated in a Phase 1 trial in healthy post‐menopausal women. KER‐012 was generally well tolerated following 3 monthly subcutaneous doses up to 4.5 mg/kg, and no dose‐limiting erythropoietic effects were observed. Decreases in serum follicle stimulating hormone and increases in bone‐specific alkaline phosphatase were observed, suggesting that high levels of activin/GDF target engagement were achieved. An exploratory post‐hoc serum proteomic analysis revealed changes that may potentially be beneficial in the context of PAH, including altered expression of biomarkers of inflammation, fibrosis and macrophage polarization, and decreased N‐terminal pro‐brain natriuretic peptide, a biomarker of cardiac dysfunction. These findings supported the rationale for evaluating KER‐012 as a potential disease‐modifying agent in PAH. TROPOS (NCT05975905) is a Phase 2 trial expected to be conducted at ~60 sites globally. Ninety adults with symptomatic PAH (WHO Group 1) receiving stable background therapy and whose 6‐min walk distance (6MWD) is >150 < 500 m, will undergo stratified randomization according to pulmonary vascular resistance (PVR) in a 2:2:2:3 ratio to one of 3 dose levels of KER‐012 (1.5, 3, or 4.5 mg/kg Q4W) or placebo. Dose selection was determined via PK/PD modeling to extrapolate potentially maximal levels of activin/GDF target engagement and optimal activin/GDF/BMP rebalancing (data to be shown). Participants will enter a 24‐week double‐blind placebo‐controlled treatment period in which changes in PVR (primary endpoint) and 6MWD (key secondary endpoint) will be evaluated, followed by a 72‐week extension period in which all participants will receive KER‐012 at one of the three dose levels. Other endpoints evaluate safety, tolerability, hemodynamics, WHO functional class, physical activity, PAH risk scores, quality of life, time to clinical worsening, and biomarkers relevant to PAH and cardiovascular disease. KER‐012 is a modified ActRIIB ligand trap designed to specifically inhibit activin and GDF signaling and preserve BMP signaling without dose‐limiting effects on erythropoiesis. TROPOS will evaluate safety and efficacy of KER‐012 in patients with PAH at a dose range between 1.5 and 4.5 mg/kg Q4W that is predicted to provide maximal levels of target engagement based on PK/PD modeling.

## A010 RKER‐012, A NOVEL MODIFIED ACTRIIB LIGAND TRAP, REDUCED PULMONARY VASCULAR PATHOLOGY IN A RAT MODEL OF PULMONARY ARTERIAL HYPERTENSION

Keith Babbs^1^, Emily Ledoux^1^, Evan Lema^1^, Chris Materna^1^, Martin Fisher^1^, Jasbir Seehra^1^, Jennifer Lachey^1^

^1^Keros Therapeutics, Lexington, USA

Pulmonary arterial hypertension (PAH) is a progressive disease characterized by remodeling and dysfunction of the pulmonary vasculature, leading to elevated pulmonary vascular resistance, right ventricle (RV) overload, and cardiac failure. The pulmonary arterial endothelial cell dysfunction that underpins PAH is associated with imbalanced TGF‐ß signaling, including inappropriately high SMAD2/3 signaling driven by increased ligand levels. RKER‐012, a research form of KER‐012, is an investigational modified ActRIIB ligand trap designed to inhibit ligands that signal through SMAD2/3, such as activins A and B, and growth and differentiation factor (GDF) 8 and GDF11. Here, we investigated the potential of RKER‐012 to prevent the pulmonary vascular effects of PAH modelled in human pulmonary arterial endothelial cells (HPAECs). Additionally, we evaluated the effect of RKER‐012 in an animal model of PAH. HPAECs were exposed to hypoxia (1% O_2_) to mimic the pulmonary cell environment observed in PAH. Gene and protein expression of hypoxia/normoxia cultured cells was assessed by qPCR, phosphorylation of SMAD2 (pSMAD2) by alphaLISA, and activin A protein levels by ELISA. Animal studies were conducted in an established PAH model, the SUGEN/hypoxia (SH) model. On day 1, groups of Sprague Dawley rats received a single 200 mg/kg subcutaneous (SQ) dose of SUGEN5416 and were placed in a hypoxic environment (10%–12% O_2_). For 3 weeks, SH rats received either vehicle (SH‐VEH) or 10 mg/kg RKER‐012 (SH‐RKER‐012) twice weekly SQ. A third healthy control group received vehicle (VEH) twice weekly and remained in a normoxic (NX) environment. After 3 weeks, rats were assessed for pulmonary vascular dysfunction and expression of associated markers. Exposure of HPAECs to hypoxia for 48 h led to a significant and sustained increase in Activin A expression and secretion, along with increased SMAD2/3 phosphorylation, which recapitulates the physiological changes observed in human PAH. The addition of activin A neutralizing antibody or RKER‐012 inhibited the endogenous increase in secreted activin and attenuated SMAD2/3 phosphorylation. In a rat PAH model, relative to NX rats, SH‐VEH rats had increased pulmonary arteriole dysfunction, including higher degrees of vascular fibrosis, inflammation, necrosis, and smooth muscle hypertrophy. This was associated with increased activin A gene expression in lung tissue, supporting the involvement of activin A. Lung tissue of SH‐VEH rats had increased expression of markers of endothelial dysfunction relative to NX rats, such as E‐selectin and VCAM‐1, and increased expression of endothelial to mesenchymal transition (EndoMT), such as CTGF and SPP1. However, relative to SH‐VEH, SH‐RKER‐012 rats had reduced pulmonary vascular dysfunction, as well as reduced expression of markers of endothelial dysfunction and EndoMT. These results demonstrate that RKER‐012 reduced hypoxia‐induced expression and secretion of activin A and phosphorylation of SMAD2/3 in a human in vitro model of PAH. Moreover, RKER‐012 reduced pulmonary vascular pathology in a rat PAH model. Together, these data demonstrate RKER‐012 can reduce PAH pathology in this model, potentially by altering the signaling in the pulmonary vascular endothelium that underlies the disease. These results suggest that KER‐012 has the potential to treat PAH in humans by attenuating the underlying pathology of the pulmonary vasculature.

## A011 EFFECTS OF INHALED SERALUTINIB ON RIGHT VENTRICULAR‐PULMONARY ARTERIAL (RV‐PA) COUPLING AND RIGHT HEART FUNCTION IN PULMONARY ARTERIAL HYPERTENSION (PAH)

Jean‐Luc Vachiéry^1^, Khodr Tello^2^, Victor M. Moles^3^, Scott Solomon^4^, Roberto Badagliacca^5^, Raymond L. Benza^6^, Richard N. Channick^7^, Kelly M. Chin^8^, Robert P. Frantz^9^, Anna R. Hemnes^10^, Luke S. Howard^11^, Vallerie V. McLaughlin^3^, Olivier Sitbon^12^, Roham T. Zamanian^13^, Matt Cravets^14^, Robin Osterhout^14^, Jean‐Marie Bruey^14^, Robert F. Roscigno^14^, Richard Aranda^14^, Lawrence S. Zisman^14^, Hossein‐Ardeschir Ghofrani^15^

^1^Université Libre de Bruxelles, HUB – Hôpital Erasme, Brussels, Belgium, ^2^Justus‐Liebig‐University Giessen, Giessen, Germany, ^3^University of Michigan, Ann Arbor, USA, ^4^Brigham and Women's Hospital, Harvard Medical School, Boston, USA, ^5^Sapienza University of Rome, Rome, Italy, ^6^Icahn School of Medicine at Mount Sinai, Mount Sinai Hospital, New York, USA, ^7^University of California Los Angeles, UCLA Medical Center, Los Angeles, USA, ^8^UT Southwestern Medical Center, Dallas, USA, ^9^Mayo Clinic, Rochester, USA, ^10^Vanderbilt University, Vanderbilt University Medical Center, Nashville, USA, ^11^Imperial College Healthcare NHS Trust, Hammersmith Hospital, London, UK, ^12^University of Michigan, Ann Arbor, USA, ^13^Hôpital Bicêtre (AP‐HP), Université Paris‐Saclay, Le Kremlin‐Bicêtre, France, ^14^Stanford University School of Medicine, Stanford Medicine, Stanford, USA, ^15^Gossamer Bio, Inc., San Diego, USA, ^16^Gossamer Bio, Inc., San Diego, USA, ^17^Gossamer Bio, Inc., San Diego, USA, ^18^Gossamer Bio, Inc., San Diego, USA, ^19^Gossamer Bio, Inc., San Diego, USA, ^20^Gossamer Bio, Inc., San Diego, USA, ^21^Justus‐Liebig‐University Giessen and Marburg Lung Center (UGMLC), Institute for Lung Health, Cardio‐Pulmonary Institute, Giessen, Germany

Impaired RV‐PA coupling portends a poor prognosis in PAH, and right ventricular free wall strain/systolic pulmonary artery pressure (RVFWS/sPAP) has been reported as a measure of RV‐PA coupling. Furthermore, increased RVFWS and right atrial area (RAA), as well as decreased pulmonary artery compliance (PAC) are associated with increased mortality risk in PAH. Seralutinib is a novel, small molecule kinase inhibitor that targets PDGFR, CSF1R, and c‐KIT administered via dry powder inhaler. TORREY, a phase 2, double‐blind, randomized, placebo‐controlled study of inhaled seralutinib in patients with PAH (NCT04456998) met its primary endpoint, demonstrating a significant reduction in pulmonary vascular resistance (PVR) from baseline (BL) to Week 24 compared to placebo. In addition, seralutinib significantly decreased NT‐proBNP. To test the hypothesis that seralutinib would improve RVFWS/sPAP and other measures of right heart function after 24 weeks of treatment compared to placebo in the TORREY study. Eighty‐six patients with WHO Group 1 PH (Functional Class II, III), ages ≥18 years, pulmonary vascular resistance ≥400 dyne*s/cm⁵, and on stable PAH standard of care therapy (the majority of whom were on double and triple therapy with approved PAH medications) were enrolled. Right heart catheterization (RHC) and full echocardiography were performed at BL and Week 24 and at BL, Week 12, and Week 24, respectively; both were analyzed in a blinded central laboratory. To calculate RVFWS/sPAP, the pulmonary artery systolic pressure from RHC was used. PAC was calculated from RHC data with the formula (SV/(PAS‐PAD)). Statistical analysis was performed using ANCOVA. At Week 12, the changes in RVFWS and RAA were lower in the seralutinib group vs placebo (LSMD[SE]: −3.3[1.201]; *p* = 0.0076 and LSMD[SE]: −2.12[1.037]; *p* = 0.0442, respectively). For both parameters the changes were also lower at Week 24 (LSMD[SE]: −2.62[1.240]; *p* = 0.0377 and LSMD[SE]: −1.99[0.897]; *p* = 0.0293, respectively). At Week 24, the change in RVFWS/sPAP was lower in the seralutinib group compared to placebo (LSMD[SE]: −0.051[0.020]; *p* = 0.0123). Change in NT‐proBNP correlated with change in RAA (*r* = 0.43). At Week 24, change in PAC was greater in the seralutinib group (−0.02 ± 0.085 placebo, 0.19 ± 0.089 seralutinib; LSMD[SE]: 0.22[0.104]; *p* = 0.0410). There was no effect of seralutinib on left ventricular ejection fraction. Treatment with seralutinib was associated with a significant reduction of RVFWS/sPAP. In addition, significant differences in RVFWS itself, RAA, and PAC were observed. These treatment effects support improved RV‐PA coupling and right heart function. In conjunction with concordant reductions in PVR and NT‐proBNP, these data suggest potential favourable effects of seralutinib in PAH. Based on these results, the phase 3 PROSERA study has been initiated (NCT05934526). These data have been previously presented at the ESC Congress 2023.

## A012 SERALUTINIB IMPROVES PULMONARY ARTERIAL BLOOD VESSEL VOLUME DISTRIBUTION IN PULMONARY ARTERIAL HYPERTENSION (PAH): RESULTS OF THE TORREY PHASE 2 IMAGING SUB‐STUDY

Luke S. Howard^1^, Farbod N. Rahaghi^2^, Marion Delcroix^3^, Sandeep Sahay^4^, Namita Sood^5^, Ronald L. Zolty^6^, Murali M. Chakinala^7^, Veronica Franco^8^, Pavel Jansa^9^, Shelley M. Shapiro^10^, Leslie A. Spikes^11^, Wendy Stevens^12^, James White^13^, Raymond L. Benza^14^, Richard N Channick^15^, Kelly M Chin^16^, Robert P Frantz^17^, Hossein‐Ardeschir Ghofrani^18^, Anna R Hemnes^19^, Vallerie V McLaughlin^20^, Olivier Sitbon^21^, Jean‐Luc Vachiéry^22^, Roham T Zamanian^23^, Patrick Muchmore^24^, Cso Benjamin Lavon^24^, Jan de Backer^24^, Thao Duong‐Verle^25^, Robert F Roscigno^25^, David Mottola^25^, Richard Aranda^25^, Matt Cravets^25^, Robin Osterhout^25^, Jean‐Marie Bruey^25^, Ed Parsley^25^, Lawrence S Zisman^25^

^1^Imperial College Healthcare NHS Trust, Hammersmith Hospital, London, UK, ^2^Brigham and Women's Hospital, Harvard Medical School, Boston, USA, ^3^University Hospitals of Leuven, Leuven, Belgium, ^4^Houston Methodist Hospital/Weill Cornell Medicine, Houston, USA, ^5^UC Davis Medical Center, Sacramento, USA, ^6^University of Nebraska Medical Center, Omaha, USA, ^7^Washington University School of Medicine, St. Louis, USA, ^8^The Ohio State University Wexner Medical Center, Columbus, USA, ^9^General University Hospital, Prague, Czech Republic, ^10^Greater Los Angeles VA Healthcare System and David Geffen UCLA School of Medicine, Los Angeles, USA, ^11^University of Kansas Medical Center, Kansas City, USA, ^12^The University of Melbourne at St. Vincent's Hospital, Fitzroy, Australia, ^13^University of Rochester Medical Center, Rochester, USA, ^14^Icahn School of Medicine at Mount Sinai, Mount Sinai Hospital, New York, USA, ^15^University of California Los Angeles, UCLA Medical Center, Los Angeles, USA, ^16^UT Southwestern Medical Center, Dallas, USA, ^17^Mayo Clinic, Rochester, USA, ^18^Justus‐Liebig‐University Giessen and Marburg Lung Center (UGMLC), Institute for Lung Health, Cardio‐Pulmonary Institute, Giessen, Germany, ^19^Vanderbilt University Medical Center, Nashville, USA, ^20^University of Michigan, Ann Arbor, USA, ^21^Hôpital Bicêtre (AP‐HP), Université Paris‐Saclay, Le Kremlin‐Bicêtre, France, ^22^Université Libre de Bruxelles, HUB – Hôpital Erasme, Brussels, Belgium, ^23^Stanford University, Stanford, USA, ^24^FLUIDDA, Inc., New York, USA, ^25^FLUIDDA, Inc., New York, USA, ^26^FLUIDDA, Inc., New York, USA, ^27^Gossamer Bio, Inc., San Diego, USA, ^28^Gossamer Bio, Inc., San Diego, USA, ^29^Gossamer Bio, Inc., San Diego, USA, ^30^Gossamer Bio, Inc., San Diego, USA, ^31^Gossamer Bio, Inc., San Diego, USA, ^32^Gossamer Bio, Inc., San Diego, USA, ^33^Gossamer Bio, Inc., San Diego, USA, ^34^Gossamer Bio, Inc., San Diego, USA, ^35^Gossamer Bio, Inc., San Diego, USA

Seralutinib, a novel, inhaled kinase inhibitor with anti‐inflammatory and antiproliferative effects, met the primary endpoint of reduction in pulmonary vascular resistance in the phase 2 TORREY trial in PAH (NCT04456998) and has the potential to treat pulmonary vascular remodeling. This abnormal remodeling in PAH includes distal pruning and proximal pulmonary arterial dilation. Quantitative analysis of these features is possible with CT imaging. The TORREY CT sub‐study used thin‐section, volumetric non‐contrast chest CTs followed by automated pulmonary vascular segmentation to assess pulmonary vascular blood volume distribution in patients treated with seralutinib. Baseline and Week 24 blood vessel volumes (BVVs) were determined at distinct levels defined by vessel cross‐sectional area (CSA) in 19 subjects on a background of 2–3 approved PAH therapies. BVVs of pulmonary arteries with a CSA < 5 mm² (BV5A) and >10 mm² (BV10A) were calculated. The BV5A to BV10A ratio (BV510ARATIO) was used to express relative redistribution of pulmonary arterial BVV. Linear regression was used to model the treatment effect. The BV510ARATIO increased from baseline to Week 24 in the seralutinib group (*n* = 7) versus placebo (*n* = 12; *p* = 0.028), and BV510ARATIO changes correlated with changes in stroke volume (*R* = 0.65, *p* = 0.0033) and pulmonary artery compliance (*R* = 0.56, *p* = 0.016). In heavily treated PAH subjects, adding seralutinib for 24 weeks led to a significant redistribution of pulmonary arterial BVV to smaller vessels. These data visualize and quantify seralutinib's treatment effect on the pulmonary arterial vasculature in PAH. Based on these results, the phase 3 PROSERA study has been initiated (NCT05934526). These data have been previously presented at the ERS International Congress 2023.

## A013 TRIAL IN PROGRESS: PROSERA, A PHASE 3 STUDY OF THE EFFICACY AND SAFETY OF SERALUTINIB IN ADULTS WITH PULMONARY ARTERIAL HYPERTENSION

Olivier Sitbon^1^, Raymond L. Benza^2^, Richard N. Channick^3^, Kelly M. Chin^4^, Robert P. Frantz^5^, Hossein‐Ardeschir Ghofrani^6^, Anna R. Hemnes^7^, Luke Howard^8^, Vallerie V. McLaughlin^9^, Roham T. Zamanian^10^, Jean‐Marie Bruey^11^, Matt Cravets^11^, David Mottola^11^, Lawrence S. Zisman^11^, Ed Parsley^11^, Robert F. Roscigno^11^, Richard Aranda^11^, Jean‐Luc Vachiéry^12^

^1^Hôpital Bicêtre (AP‐HP), Université Paris‐Saclay, Le Kremlin‐Bicêtre, France, ^2^Icahn School of Medicine at Mount Sinai, Mount Sinai Hospital, New York, USA, ^3^University of California Los Angeles, UCLA Medical Center, Los Angeles, USA, ^4^UT Southwestern Medical Center, Dallas, USA, ^5^Mayo Clinic, Rochester, USA, ^6^Justus‐Liebig‐University Giessen and Marburg Lung Center (UGMLC), Institute for Lung Health, Cardio‐Pulmonary Institute, Giessen, Germany, ^7^Vanderbilt University, Vanderbilt University Medical Center, Nashville, USA, ^8^Imperial College Healthcare NHS Trust, Hammersmith Hospital, London, UK, ^9^University of Michigan, Ann Arbor, USA, ^10^Stanford University School of Medicine, Stanford Medicine, Stanford, USA, ^11^Gossamer Bio, Inc., San Diego, USA, ^12^Gossamer Bio, Inc., San Diego, USA, ^13^Gossamer Bio, Inc., San Diego, USA, ^14^Gossamer Bio, Inc., San Diego, USA, ^15^Gossamer Bio, Inc, San Diego, USA, ^16^Gossamer Bio, Inc., San Diego, USA, ^17^Gossamer Bio, Inc., San Diego, USA, ^18^Université Libre de Bruxelles, HUB – Hôpital Erasme, Brussels, Belgium

Seralutinib is a highly potent inhibitor of PDGFRα, PDGFRβ, CSF1R, and c‐KIT kinase pathways that activate inflammation, proliferation, and fibrosis, and drive vascular remodeling in pulmonary arterial hypertension (PAH). Seralutinib is the first tyrosine kinase inhibitor specifically formulated for inhaled delivery to achieve deep lung deposition while minimizing systemic exposure. The phase 2 TORREY study of seralutinib in patients with WHO Group I Pulmonary Hypertension (PH) receiving standard of care (SOC) therapy met its primary endpoint, demonstrating a statistically significant reduction in pulmonary vascular resistance (PVR) compared to placebo, with favorable tolerability (NCT04456998). Significant improvements in N‐terminal pro B‐type natriuretic peptide (NT‐proBNP) and right heart function by echocardiography were also observed. PROSERA is a global phase 3, randomized, double‐blind, placebo‐controlled study to evaluate the efficacy and safety of inhaled seralutinib in adults with WHO Group 1 PH, Functional Class (FC) II or III, PVR ≥ 400 dyne·s/cm⁵, 6‐min walk distance (6MWD) 150–450 m, either REVEAL Lite 2 Risk Score ≥ 5 or NT‐proBNP ≥ 300 ng/L, and on stable treatment with up to three SOC PAH background therapies, including parenteral prostacyclins (NCT05934526). A total of 350 patients will be enrolled and randomized to receive either seralutinib 90 mg or placebo by dry powder inhaler twice daily for up to 48 weeks. The primary endpoint is change in 6MWD from baseline to Week 24. Key secondary endpoints (measured from baseline) are time to the first event of clinical worsening through Week 48, proportion of patients achieving clinical improvement (Week 24), change in NT‐proBNP (Week 24), and proportion of patients with ≥ 1 point decrease in REVEAL Lite 2 Risk Score (Week 24). Other secondary endpoints (measured from baseline) include the proportion of patients with each of the clinical worsening outcomes (through Week 48), proportion of patients who improve in WHO FC or maintain WHO FC II (through Week 48), and change in health‐related quality of life (PAH‐SYMPACT, EQ‐5D‐5L; Week 24). Patients who complete the study on blinded treatment may be eligible to enroll in a separate open‐label extension study. A functional respiratory imaging sub‐study will examine the effect of seralutinib on pulmonary vascular remodeling.

## A014 ASSOCIATION OF QTC PROLONGATION WITH ELEVATED MEAN ARTERIAL PRESSURE AND PULMONARY VASCULAR RESISTANCE

Athina Batsouli^1^, Athanasia Megarisiotou^1^, Panagioula Niarchou^1^, Panteleimon Papakonstantinou^1^, Panagiota‐Natalia Zimpounoumi‐Keratsa^1^, Efstathia Prappa^1^, Anastasia Anthi^2^, Efrosini Dima^2^, Anastasia Kotanidou^2^, Antonios Sideris^1^, Stylianos Orfanos^2^, Sotirios Xydonas^1^

^1^Evangelismos General Hospital, Athens, Greece, ^2^1st Department of Critical Care and Pulmonary Hypertension Center National & Kapodistrian University of Athens, Medical School, Evangelismos General Hospital, Greece

Pulmonary hypertension (PH) is a pathophysiological disorder that is associated with multiple cardiovascular and respiratory diseases. The early diagnosis of PH remains challenging, with right heart catheterization (RHC) being the gold standard in the diagnostic process. More diagnostic noninvasive tools are needed to further stratify this population and predict mortality.

Electrocardiogram (ECG) is a widely used, noninvasive and cost‐effective tool currently recommended in all patients with PH. Multiple ECG abnormalities have been described in this population reflecting alterations both in size and function of the right heart chambers but also in repolarization. QT interval reflects the repolarization of myocardial cells and QTc prolongation has been associated with increased mortality in patients with PH and RV dysfunction. Pulmonary vascular resistance (PVR) and mean pulmonary artery pressure (mPAP) as directly measured on RHC provide prognostic information in PH to varying degrees. We sought to investigate the correlation of QTc duration to mPAP and PVR. Fifty patients diagnosed in a referral center of PH over a period of 2 years, were enrolled. RHC was conducted under fluoroscopic guidance by the internal jugular vein or femoral vein approach, using a Swan‐Ganz catheter, according to standard protocol. All pressure measurements were performed at end‐expiration, and cardiac output (CO) was assessed using the  direct Fick method. PVR ([mPAP − PAWP]/CO) was calculated for each patient. 12‐lead ECG was carried out in supine and standard patients' position with paper speed of 25 mm per second and voltage lead of 10 mm per millivolt, within maximum 24 h of RHC. The QT interval was measured manually, from the beginning of the QRS complex to the end of the T wave, in any lead best showing the end of the T wave, and averaged over 3–5 beats. The QTc interval was calculated using Bazett's formula (corrected QT interval = QT/√RR). All 50 patients were included, of whom 66% were female (*n* = 33), and the mean age was 62 ± 13 years. The study population comprised of patients with PH divided in groups according to WHO classification: Pulmonary Arterial Hypertension (72%, *n* = 36), PH associated with left heart disease (10%, *n* = 5), PH associated with lung disease (6%, *n* = 3) and chronic thromboembolic pulmonary hypertension (12%, *n* = 6). The median QTc interval in this study was measured as 440.3 ± 37.64 ms. The median mPAP was 36 ± 11 mmHg. The median measured PVR was 5.3 ± 3 mmHg*min/L. Statistical analysis revealed a significant correlation between the mean value of QTc interval and the both mean value of mPAP (*p*‐value ~ 0.019) and the mean value of PVR (*p*‐value ~ 0.017).

In this study, prolonged QTc interval in a population with PH was associated with worse hemodynamics in the RHC. Specifically increased QTc duration correlated significantly with elevated values of mPAP and PVR, which suggests more severe disease. QTc prolongation may serve as a predictor of severe pulmonary hypertension and poor prognosis and define patients in need of intensive treatment.

## A015 P WAVE AREA PREDICTS INVASIVE HEMODYNAMICS IN PULMONARY HYPERTENSION

Athina Batsouli^1^, Panagioula Niarchou^1^, Athanasia Megarisiotou^1^, Panteleimon Papakonstantinou^1^, Natalia‐Panagiota Zimpounoumi‐Keratsa^1^, Efstathia Prappa^1^, Anastasia Anthi^2^, Efrosini Dima^2^, Dimitrios Eleftheriou^1^, Anastasia Kotanidou^2^, Antonios Sideris^1^, Stylianos Orfanos^2^, Sotirios Xydonas^1^

^1^Cardiology Department, Evangelismos General Hospital, Athens, Greece, ^2^1st Department of Critical Care and Pulmonary Hypertension Center National & Kapodistrian University of Athens, Medical School, Evangelismos General Hospital, Greece

Pulmonary hypertension (PH) is a potentially life‐threatening cardiovascular disease. The time from symptom onset to PH diagnosis remains increased, and the majority of patients presents with advanced stages of the disease. Current guidelines recommend electrocardiogram (ECG) as an additional diagnostic tool in PH, giving priority to echocardiography for screening and to right heart catheterization (RHC) for diagnosing PH. ECG is a noninvasive, easily accessible diagnostic tool and despite its low sensitivity and specificity in patients with PH, it can be used in the early diagnostic work‐up. P wave area is a P wave parameter that is associated with left atrial enlargement. Cardiopulmonary hemodynamics assessed by RHC provide important prognostic information and pulmonary vascular resistance (PVR) is recognized as an independent predictor of outcome. There are limited studies to access the association between different P wave parameters others than P wave amplitude and duration and PVR values. We sought to investigate the relation between P wave area and PVR. We enrolled 50 patients diagnosed with PH in a referral center over a period of 2 years. The RHC was conducted under fluoroscopic guidance using a Swan‐Ganz catheter through right internal jugular or right femoral vein according to standard protocol. All pressure measurements were performed at end‐expiration, and cardiac output (CO) was assessed using the direct Fick method. PVR ([Mpap − PAWP]/CO) was calculated for each patient. 12‐lead ECG was carried out in supine and standard patients' position with a paper speed of 25 mm per second and voltage of 10 mm per millivolt, within a maximum 24 h of RHC. P wave area was calculated in lead II using this formula: P wave area = ½ P wave duration* P wave voltage (normal value: <4 ms*mV). All patients were entered in the analysis and 66%(*n* = 33) were female. Minimum, maximum, and median age of these patients were 22, 85, and 61.8 ± 13.3 years old, respectively. Patients were divided based on WHO classification of PH into four different groups: 72% (*n* = 36) in group 1 PH, 10% (*n* = 5) in group 2 PH, and 6% (*n* = 3) in group 3 PH and 12%(*n *= 6) in group 4 PH. The median calculated P wave area was 6.17 ± 2.53 ms×mV. The median measured PVR was 5.3 ± 3.02 mmHg*min/L. Statistical analysis revealed a significant correlation between mean value of P wave area and mean value of PVR (mean = 6.17 ± 2.53 ms*mV vs mean = 5.3 ± 3.02 mmHg*min/L respectively, *p* ~ 0.000093). Based on the results of this study, increased values of P wave area correlated significantly with increased values of PVR. Therefore, P wave area may serve as an easily measured screening tool of pulmonary hypertension severity and can be used to identify patients who are needed to be referred early to specialized PH centers.

## A016 MITOCHONDRIA PLAY A KEY ROLE IN THE OXYGEN SENSING OF THE HUMAN DUCTUS ARTERIOSUS

Rachel Bentley^1^, Ashley Martin^1^, Patricia Lima^1^, Jeffrey Mewburn^1^, Charles Hindmarch^1^, Kimberly Dunham‐Snary^1^, Stephen Archer^1^

^1^Queen's University, Kingston, Canada

The fetal circulation relies on the ductus arteriosus (DA), a vessel connecting the pulmonary artery (PA) and aorta. The DA functions to divert placentally oxygenated blood away from the unventilated, developing, lungs to the systemic circulation. At birth, the DA rapidly constricts with the first breath in response to an increase in arterial oxygen diverting blood to the newly ventilated lungs. The PAs simultaneously dilate allowing uptake of oxygen from ventilated alveoli. Failure of the DA to close in response to oxygen results in persistent ductus arteriosus (PDA), one of the most common complications of preterm birth, which if unresolved leads to heart failure and pulmonary hypertension. Mitochondria are known to play an important role in the oxygen response of DA smooth muscle cells (DASMC), however the mechanism is still not fully understood. There are many conserved redox sensor proteins amongst specialized oxygen sensing tissues, one of which is a subunit of mitochondrial Complex I, NDUFS2 (NADH:Ubiquinone oxidoreductase core subunit 2). NDUFS2 contributes to oxygen sensing in adult pulmonary artery and carotid bodies. Here we assess whether NDUFS2 also plays a vital role in DA oxygen sensing. DASMC were isolated from DAs obtained from term infants at the time of congenital heart surgery (*n* = 6). Cells were purified via fluorescence activated cell sorting, excluding CD90+ and CD31+ cells (fibroblasts and endothelial cells, respectively). Smooth muscle cell purity was confirmed using flow cytometry for α‐smooth muscle actin (α‐SMA), caldesmon, and smoothelin‐B. Cell lines were ≥90% α‐SMA+ and 100% caldesmon+ and smoothelin‐B+. We then measured the oxygen responsiveness of DASMC using confocal microscopy. Cells were cultured in hypoxia (2.5% oxygen, pO_2_ = 41 mmHg), with an oxygen‐induced increase in calcium relative to hypoxic baseline (measured using Cal‐520AM) serving as a surrogate for DASMC constriction. DASMC were treated for 48 h with 30 pmol siRNA and 90 pmol Lipofectamine® RNAiMAX to knockdown gene expression of putative oxygen sensors or control proteins including: negative control siRNA, siRNA targeting NDUFS2 (siNDUFS2), siRNA targeting other Complex I subunits not implicated in oxygen sensing (siNDUFS1 and siNDUFS7), and siRNA targeting purported adult PA mitochondrial oxygen sensors in Complex III and IV (siUQCRFS1 and siCOX4i2, respectively). Knockdown was confirmed using qPCR and western blot. There was a 25.7% (±3.4%, *n* = 66) increase in intracellular calcium relative to hypoxic baseline in siNC‐treated cells, not significantly different from untreated cells (*p* = 0.96). The oxygen response was significantly depressed when treated with siNDUFS2 (*n* = 66, *p* = 0.0005) or siUQCRFS1 (*n *= 65, *p* = 0.0003), but was unchanged by the other siRNA treatments. These data suggest a critical role for the mitochondrial electron transport chain in DA oxygen responsiveness, specifically the iron–sulfur clusters NDUFS2 in Complex I and UQCRFS1 (Rieske Fe–S cluster) in Complex III. While further functional studies will be required, our findings suggest a novel potential therapeutic target to modulate DA patency within the mitochondrion.

## A017 MICRORNA‐224 ORCHESTRATES THE BMP PATHWAY AND REPRESENTS A NEW THERAPEUTIC TARGET FOR PULMONARY HYPERTENSION

Olympia Bikou^1,2^, Aymen Halouani^3^, Catherine Swarts^1^, Petros Avramopoulos^4^, Malik Bisserier^1^, Erik Kohlbrenner^1^, Roger Hajjar^5^, Stefan Engelhardt^4^, Lahouaria Hadri^1^, Sebastien Bonnet^6^, Yassine Sassi^3^

^1^Icahn School of Medicine at Mount Sinai, New York, USA, ^2^Department of Medicine I, LMU University Hospital, Munich, Germany, ^3^Fralin Biomedical Research Institute at Virginia Tech Carilion, Virginia, USA, ^4^Institute of Pharmacology and Toxicology, Technical University, Munich, Germany, ^5^Gene and Cell Therapy Institute, Massachusetts General Brigham, Boston, USA, ^6^Pulmonary Hypertension Research Group, Université Laval, Quebec, Canada

Pulmonary arterial hypertension (PAH) is a severe vascular disease that leads to right heart failure and death. PAH therapies primarily target pulmonary vasoconstriction, but only modestly affect pulmonary vascular remodeling. Currently, there is no cure for PAH, and new therapeutic options targeting the roots of the disease are desperately needed. MicroRNAs (miRs) have emerged in the last decades as key regulators in health and disease. We identified miR‐224 as a lung enriched miR and in silico approaches for PAH predicted miR‐224 among the miRs that target PAH related genes. In this study we aimed to investigate the role of miR‐224 in pulmonary vascular remodeling and to define the mechanism of miR‐224 action in PAH. We found pulmonary miR‐224 levels to be increased in PH‐diseased lungs (in mice, rat, pig, and humans) and in human pulmonary artery smooth muscle cells (hPASMCs) isolated from PAH patients. In vitro studies revealed that miR‐224 is enriched in hPASMCs and its expression is necessary and sufficient to induce hPASMCs proliferation. We next tested the therapeutic effect of miR‐224 inhibition using three different PAH animal models in mice and rats. In a first approach, we intra‐tracheally aerosolized an adeno‐associated virus1‐tough decoy‐miR‐224 (AAV1‐TuD‐224) to PH‐diseased mice (Sugen/hypoxia model). AAV‐Ctrl‐treated mice displayed all the hallmarks of PAH (i.e., increased Fulton index, RVSP, cardiomyocyte hypertrophy and pulmonary arterial medial thickness), whereas AAV1‐TuD‐224‐treated mice displayed a marked and significant decrease in these parameters. We next intra‐tracheally delivered a chemically modified antisense oligonucleotide specific for miR‐224 (LNA‐224) to PH‐diseased mice and rats (Sugen/hypoxia model). Consistent with our genetic approach, LNA‐224 significantly protected mice and rats from cardiac hypertrophy and pulmonary vascular remodeling at the tissue and cellular levels. We finally delivered LNA‐224 to monocrotaline‐treated rats and found (by magnetic resonance imaging, hemodynamics, morphometric and histological measurements) that miR‐224 inhibition improves survival and suppresses PAH. Mechanistically, we found that miR‐224 represses BMP signaling by directly targeting four pathway factors. Our data suggest that miR‐224 plays a pivotal role in pulmonary vascular remodeling by targeting the BMP pathway and may have therapeutic value for PAH.

## A018 LIVING WITH PULMONARY HYPERTENSION—2022 SURVEY OF UK PATIENTS

Iain Armstrong^1^, Catherine Billings^1^, Paul Sephton^1^, Shaun Clayton^1^, Mary Ferguson^1^, John Smith^1^

^1^Pulmonary Hypertension Association UK, Sheffield, UK

The Pulmonary Hypertension Association UK (PHA‐UK) has previously carried out surveys of UK patients' experiences of living with pulmonary hypertension (PH). Findings from these three previous surveys carried out in 2007, 2010, and 2017 [1] have had a big impact, underpinning the charity's work supporting PH patients. To update knowledge of patients' lived experience of PH in the UK. A quantitative survey covering diagnosis, treatment, and living with PH was made available, both online and in paper format in December 2022 to PHA‐UK members and patients on PH therapy. Eight hundred fifty‐nine responses were received. Mean age of respondents was 64 ± 14 years and 73% were female. Mean age±SD at diagnosis was 56 ± 17 years. The time between first experiencing symptoms and diagnosis was ≥1 year for 51% of patients. Delays were observed all along the diagnostic pathway. Initially, after the development of symptoms 33% of patients had waited ≥1 year before visiting a doctor. After seeing their GP, it took ≥1 year to be referred to the hospital for 40% of the patients. Time taken for a referral from a general hospital to a specialist centre to be made was ≥1 year for 28% of patients. 35% of patients saw 4+ doctors before receiving their diagnosis and 9% saw ≥7. In the group of patients whose time from first symptoms to diagnosis was ≥3 years, 55% saw 4+ doctors before receiving their diagnosis and 22% saw ≥7. After diagnosis 86% of respondents reported an improvement in QOL when on PH‐specific therapy and 78% felt their management and treatment had reduced their concerns over life expectancy. When asked “What matters to you most when it comes to your treatment?” 52% listed ‘Improvement in overall quality of life (QOL),” 33% put “Increasing life expectancy,” 14% “Reducing symptoms” and 3% felt “Lack of side effects” was most important. The findings of this survey differ little from the survey conducted by PHA‐UK in 2017. Disappointingly, despite much work by the PH community to raise awareness of PH, the time between first experiencing symptoms and diagnosis was ≥1 year for 51% of patients (48% in 2017). Delays are seen along the diagnostic pathway. PHA‐UK will continue to work to try to improve awareness of PH, by exploring innovative approaches, to continue to support patients.

## A019 PULMONARY HYPERTENSION AND NUTRITION: SURVEY OF UK PATIENTS

Iain Armstrong^1^, Shaun Clayton^1^, Catherine Billings^1^, Paul Sephton^1^, Mary Ferguson^1^, John Smith^1^

^1^Pulmonary Hypertension Association UK, Sheffield, UK

Nutritional deficiencies have been described in patients with pulmonary arterial hypertension (PAH) and it has been suggested that these deficiencies may trigger or aggravate disease progression. Patients are often given advice on diet such as increasing iron or reducing sodium intake to help with symptoms. Data on dietary habits in PAH patients are however lacking and a greater understanding is needed if dietary advice is to be an effective adjunct to therapy. A questionnaire, which included sections on dietary habits, food frequency, food preparation, thoughts and feelings about their diet, and the advice they had received about diet, was made available to members of PHA UK in paper format and online. Six hundred and eight questionnaires were returned. Mean ± SD age of respondents was 60 ± 17 years, 74% were female, 3% reported that they had or had previously had an eating disorder. Twenty‐three percent reported they were the only adult living in their home. Three percent of respondents reported they were vegetarian, 1% vegan, 2% pescatorian, 4% gluten‐free, and 2% lactose intolerant. Sixty‐one percent ate a home prepared meal every day and 55% reported cooking meals themselves. Respondents felt they ate too much of a number of foods (sugar 32%, salt 17%, and snacks/processed food 30%) and too little of others (fruit 41%, vegetables 31%, and protein 14%). Diet was felt by respondents to influence well‐being. When they ate well 39% reported they felt they had more energy/felt happier whereas when they ate badly 24% noticed they felt worse. Twenty nine percent felt bad if they ate too much. Sixty eight percent of patients felt they could make improvements to their diet. Respondents who reported that they did not eat as well as they would like to, felt that factors stopping them were cost of food (59%), motivation (46%) and PH symptoms preventing them from cooking (45%). When asked in which area of their diet they felt they needed the most help or support “Understanding how nutrition can influence my PH symptoms” ranked highly with 78%. Ten percent had already received dietary advice. Forty five percent would like to receive nutrition guidance as part of their PH care from their hospital/specialist centre. A strong interest was shown by PHA UK members in diet and its influence on PH with over 600 replies to the questionnaire. Many respondents felt their diets could be improved and dietary advice as part of the routine care may improve patient well‐being. The development of dietary advice addressing the areas of concern highlighted by this questionnaire will be the focus of further work by PHA UK.

## A020 A NOTCH SIGNAL DERIVED FROM ENDOTHELIAL CELLS IS ESSENTIAL FOR THE DEVELOPMENT OF PULMONARY HYPERTENSION ASSOCIATED WITH LUNG FIBROSIS

Zuriñe Blasco^1,^ Ana Pardo Saganta^1^, Jesús Ruiz Cabello^2^

^1^ILH, Institute for Lung Health, Giessen, Germany, ^2^CIC BIOMAGUNE, Centre for Cooperative Reasearch in Biomaterials, San Sebastian, Spain, ^3^CIBERES, CIBER de Enfermedades Respiratorias, Madrid, Spain

Pulmonary hypertension associated with lung fibrosis (PH‐PF), is a disease characterized not only by the presence of exaggerated ECM deposition (fibrosis) but by the existence of excessive vascular smooth muscle cell proliferation in small pulmonary arteries (PA), leading to elevated pulmonary vascular resistance with consequent right ventricle (RV) failure and death (1). Notch3 activation has been suggested to have a crucial role in pulmonary artery smooth muscle cell (PASMC) proliferation and differentiation in the context of PH (2)(3). We confirm here using a newly developed Notch3 inducible mouse model that indeed the inhibition of Notch3 specifically in PASMCs prevents from vascular remodeling and lung function decline following bleomycin administration. Interestingly, fibrosis was not reduced in the lung parenchyma. Next, we observed an increased expression of the Notch ligands, Jagged1(Jag1) and Jagged2 (Jag2), in endothelial cells following bleomycin administration. This suggested that endothelial cells could activate Notch3 in neighboring PASMCs driving the pathogenesis involved in PH. To test this hypothesis, we generated CDH5‐CreER; Jag1 f/f and CDH5‐CreER; Jag2 f/f transgenic mice, to inhibit Jag1 or Jag2 respectively, specifically in endothelial cells upon tamoxifen administration. Bleomycin‐treated CDH5‐CreER; Jag1f/f and CDH5‐CreER; Jag2 f/f not only showed a reduction in every vascular remodeling parameter (Fulton Index ((RV/LV + S)), Medial wall area and thickness of PA) but also, collagen content in the lung parenchyma was significantly decreased in both models (tested by hydroxyproline assay and collagen immunofluorescence). However, while the lack of endothelial Jag2 blocked the pathological differentiation of PASMC, no effect was observed when endothelial Jag1 was inhibited. Finally, we demonstrate that inhibition of endothelial‐derived Jag1 and Jag2 prevents lung and heart function decline analyzed by micro‐CT and lung function test (LFT). Altogether, our results suggest that endothelial Jag1/2—smooth muscle Notch3 signaling is involved in the development of PH‐PF, and therefore, targeting this pathway may be a novel therapeutical approach for this disease.

## A021 HEART RATE VARIABILITY AS A PROGNOSTIC MARKER IN PEDIATRIC PULMONARY ARTERIAL HYPERTENSION—A PROSPECTIVE OBSERVATIONAL STUDY

Prashant Bobhate^1^, Satish Kumar, Sangamesh Bawage, Tanuja Karande

^1^Kokilaben Dhirubai Ambani Hospital, Mumbai, India

PAH is a progressive disease with high morbidity and mortality. Risk stratification in now a standardized modality to assess as well as modify therapy in adult patients with PAH. However similar risk stratification in pediatric patients with PAH is challenging due to the vast variety in the age, body surface area and functional capability of pediatric patients. The current modalities of risk stratification are either invasive or subjective and may not be performed in across the pediatric age group. Hence there is a pressing need to identify a noninvasive objective parameter to assess risk in pediatric PAH. To study heart rate variability as a prognostic marker in paediatric patients with pulmonary arterial hypertension. Study Design: Prospective interventional study, Study period: Jan 2022–Sept 2023. All prevalent paediatric patients with idiopathic PAH who were stable on dual combination therapy for atleast 3 months were included in the study. All patients underwent a detailed clinical evaluation, echocardiogram, and Nt Pro BNP. Patients were divided into three risk categories using the European PVD risk calculator. Parameters measuring heart rate variability like standard deviation of normal‐to‐normal intervals (SDNN), Standard deviation of mean values for normal to normal intervals (SDANN) and square root of the mean square differences of successive RR intervals (RMSSD) were measured using a holter device. Patients were followed up in the PH clinic and and were assessed for disease progression (increase in NT‐pro‐BNP or decrease in 6‐min distance covered of >10% from baseline, or deterioration of functional by class I or mortality or listing for lung transplant/Potts shunt. Mean values of Holter parameters between progressive and nonprogressive group and among three risk groups were compared. 46 (28 Females) with idiopathic PAH patients were included in the study. Mean age 8.6 + 4.9 years. mean weight 22 + 13 kg and mean BSA 0.8 + 0.3. Fifteen were in a high‐risk group, 17 in the intermediate, and 14 in a low‐risk category at the time of recruitment. Patients in high‐risk category had significantly lower values of HRV [SDNN (60.2 ± 31.7 vs 79.5 ± 24.01 vs 80.6 ± 28.4 ms, *p* = 0.019), SDANN (49.5 ± 26.4 vs 62.2 ± 18.2 vs 63.4 ± 27.1 ms, *p* = 0.08), RMSDD (20.8 ± 11.5 vs 33.4 ± 18.3 ms vs 32.4 ± 14.4 ms, *p* = 0.05)] as compared intermediate and low risk respectively. Patients (10) who had clinical worsening had lower values of SDNN (62.4 ± 21.9 vs 78.6 ± 32.3 ms, *p* = 0.02), SDANN (50.3 ± 18.09 vs 62.05 ± 27.9 ms, *p* = 0.05) and RMSDD (22.7 ± 13.5 vs 31.6 ± 15.8 ms, *p* = 0.015)]. SDNN of 61.7 ms, SDANN of 49.7 ms, and RNSDD of 20.1 ms were able to predict disease progression with sensitivity and specificity of 64.2% and 78.7%, 53.5% and 76.5%, and 50% and 78.7%, respectively. HRV is significantly lower in patients who are in a high‐risk category and those who demonstrated disease progression and could be used as a noninvasive, objective parameter to predict disease progression.

## A023 SAFETY AND EFFICACY OF TRANS CATHETER POTTS SHUNT IN ADULTS WITH DRUG REFRACTORY PAH

Prashant Bobhate^1^

^1^Kokilaben Dhirubai Ambani Hospital, Mumbai, India

Reversed Potts shunt is a palliative treatment option for patients with end‐stage PAH not responding to medical therapy. Surgical mortality is higher in adults with PAH undergoing Potts shunt. Transcatheter Potts shunt an is option which has been frequently used in paediatric population, however safety and efficacy of this procedure is yet to be validated in adult population. This is a prospective single‐centre study performed to assess the feasibility and outcomes of Transcutaneous Potts shunt in adults with severe PAH. Nine patients with supra systemic PA pressures without significant intra or extra‐cardiac shunt were evaluated and counselled for undergoing the procedure. All of them were diagnosed with idiopathic PAH and were symptomatic on maximal medical therapy. All patients were evaluated with clinical, biochemical and radiographic evaluation of PAH. A distance of <4 mm between DAO and LPA was considered appropriate for the trans catheter approach. The procedure was done under general anaesthesia and mechanical ventilation. Femoral arterial access was taken, and hemodynamic right heart catheterization was done in all to confirm supra‐systemic PA pressures. Arterial and venous groin sheaths were upgraded to 8.5F Agilis and 7F destination sheaths, respectively, and simultaneous DAO and LPA angiograms were done to delineate the anatomy. Baylis RF ablation wire was taken through a micro catheter in Agilis and positioned in proximal Dao while a 35 mm snare was positioned right opposite in the LPA through the venous side, and a tract was created between using electrocautery. After pre‐dilating this tract, a covered stent was placed between DAO and LPA to achieve equalization of PA and AO pressures or lower limb saturation of 20% less than upper limb saturations. Post‐procedure patient shifted to cardiac ICU, where supports were slowly tapered, and pulmonary vasodilators optimised to achieve a difference in upper and lower limb saturations of 20% while maintaining a normal cardiac output. Nine patients (seven females) median age 31 years (12.5–46) were considered suitable for the procedure. Pre‐procedure mean PVRI of 37.3 WUm^2^ (17.3–42.1) and cardiac index of 2.03 L/min/m^2^ (1.39–2.70). Seven out of nine patients survived, while in two patients, the procedure could not be completed due to repeated PAH crisis. Three patients required post‐dilatation of the stent with an appropriate‐sized balloon. Median time of ventilation was 5.7 h (4–6 h) and ICU stay was 2.2 days (2–3 days). Median follow‐up is 9 months (1–19 months). All of them showed sustained clinical, echocardiographic, and biochemical improvement and reduced need for medical therapy. Transcutaneous Potts shunt is a feasible and promising treatment option for adult patients with Severe PAH with Supra systemic PA pressures. Optimal timing and pre and post‐procedure stabilization play a major role in outcomes. Long term follow‐up is required to evaluate sustained responses.

## A024 SELEXIPAG IN PEDIATRIC PULMONARY HYPERTENSION: SINGLE CENTER EXPERIENCE FROM LMIC

Prashant Bobhate^1^, Satish Kumar

^1^Kokilaben Dhirubai Ambani Hospital, Mumbai, India

PAH is a progressive disease with high morbidity and mortality. Initiation of prostacyclin analogues is recommended for high‐risk patients. Selexpiag is the first prostanoid to be launched in India. Experience in the pediatric population is limited to case reports. We report our institution's experience in the use of selexipag in children. To study efficacy and safety profile of selexipag in pediatric PAH. Prospective observational study of children with PAH, treated with oral add‐on selexipag. Patients showing evidence disease progression on dual pulmonary vasodilators were considered for initiating of Selexipag. All patients had right heart catheterization, clinical, echocardiographic, and N‐terminal pro b‐type natriuretic peptide (Nt pro BNP) before initiation of Selexipag and noninvasive assessment at follow up. Oral selexipag was started in day care with doses of 50, 100, and 200 μg BD in children weighing <15, 16–30, and >30 kg, respectively. Rapid uptitration with 50–100 μg per dose/3 days until maximal tolerated dose. Vital parameters were monitored before and up to 2 h. Patients were then asked to report side effects if any. Clinical improvement was defined as a reduction in NT‐pro‐BNP of >10% and improvement in FC > 1. Twelve (seven male) consecutive children with IPAH, median age 8.5 years (3.2–18), median Ht 128 cm (90–162), median Wt 23.8 Kg (6.6–46.3) were included. Five were in WHO FC 4, and seven in FC 3. Median mPAP 68 mmHg (23–98), median PVRi 15.8 Woodsunit.m2 (2.7–29.3) and median Rp/Rs ratio 0.93 (0.29–1.54). Median dose of 350 μg (100–800 μg) was achieved. Median follow‐up duration was 7.3 months (1–17). 9/12 (75%) demonstrated clinical improvement with WHO FC 2, median decrease in Nt Pro BNP of 53.7% (21–97), median increase of 38.6% (20–196) in TAPSE and median increase of 50% in RVFWS (22–176). 3/12 demonstrated disease progression (1 mortality, 1 transcatheter Potts, 1 referred for lung transplant). Transient side effects included headache, jaw pain, mild epistaxis, and loose stools. None of the patients discontinued the medications due to side effects. Selexipag may be used in paediatric PAH with acceptable benefits and minimal side effects.

## A025 UTILITY OF SPECKLE TRACK IMAGING AS EARLY ECHOCARDIOGRAPHIC MARKER FOR RISK STRATIFICATION, PROGNOSTICATION AND MANAGEMENT OF PATIENTS WITH IDIOPATHIC PULMONARY HYPERTENSION (IPAH)

Prashant Bobhate^1^, Sangamesh Bawage

^1^Kokilaben Dhirubai Ambani Hospital, Mumbai, India

To Access use of Speckle tracking echocardiographic (STE) parameters as early marker for risk stratification, prognostication and management of IPAH patients. To Compare STE parameters with conventional echocardiographic parameters in patients with IPAH. Total 76 patients of IPAH patients presented to PAH clinic were evaluated by both conventional echocardiographic and STE parameters along with other risk stratification parameters like NT pro BNP, 6‐min walk test, WHO functional class, etc. Risk stratification and prognostication of study population was done as Per REVEAL lite 2 score into High, intermediate, and low risk. Three groups were compared for conventional echo parameters for PAH severity like RV FAC, TAPSE, LV eccentricity index, Systolic: Diastolic ratio, etc. and STE parameters like RV Global longitudinal strain (RVGLS), RV free wall strain (RVFWS), and RA strain. Of the total 76 patients 49(64%) were female with median age of 13.9 years (0.25–69 years). 28 (36.8%) were in low risk, 20 (26.3%) in intermediate, 28 (36.8%) were in high‐risk category as per REVEAL lite 2 risk score. Mean overall RV GLS, RV FWS, and RA strain, respectively, was −12.95% (SD = +/−4.3), −13.99% (SD = +/−5.4), and 23.4% (SD = +/−9.9). Mean overall TAPSE and RVFAC were respectively 1.98 cm (SD = +/−1.93), and 20.5% (SD = +/−8.0). There was significant difference in Speckle track imaging parameters compared to conventional echo parameters for risk stratification—RV FWS (*p* = 0.030), RV basal segment strain (*p* = 0.03) RV mid‐segment strain (*p* = 0.004), RA strain (*p* = 0.008) had statistically significant correlation for risk stratification compared to conventional echo parameters like TAPSE(*p* = 0.276), RV FAC(*p* = 0.354). RV segmental, RV FWS, and RA strain were affected early when conventional parameters TAPSE were normal in low‐risk and intermediate‐risk category. Speckle tracking imaging parameters—RV free wall strain, RV basal and RV mid segmental strain, and RA strain had better correlation than conventional parameters for risk stratification in PAH patients. Speckle tracking echo parameters were found to be affected early than conventional parameters thus would help in early acceleration of treatment and help in better management of PAH patient.

## A026 LEFT VENTRICULAR SHORTENING INDEX IN IDIOPATHIC PULMONARY ARTERIAL HYPERTENSION

Yurii Botsiuk^1^, Yuriy Sirenko

^1^State Institution “National Scientific Center “the M.d. Strazhesko Institute Of Cardiology, Clinical And Regenerative Me, Kyiv, Ukraine

The interconnectedness of the work of both ventricles plays a significant role in idiopathic pulmonary arterial hypertension (IPAH). That the main problem with this disease lies in the pulmonary vascular system, the prognosis and quality of life mainly depends on the dysfunction and systolic insufficiency of the right ventricle (RV). At the service of it, the systolic function of the left ventricle (LV), which is necessarily performed according to the ejection of the LV ejection fraction (LV EF) in the case of normal or hyperdynamic, but it does not reflect those mechanical processes and properties of the LV that occur in IPAH. In separate studies, it has been shown that a decrease in left ventricular global longitudinal strain (LV GLS) is a common phenomenon in IPAH. In addition, ventricular septal shift (VSS) with LV pressure overload is a prognostic factor for consumption. We hypothesized that the presence interrelation LV GLS to VSS may be a sensitive marker of mechanical and hemodynamic changes in IPAH. Our aims was: (i) to evaluate and describe the changes in the mechanical function of the LV in LAH based on transverse and longitudinal movement; (ii) to determine the extent to which changes in LV GLS/VSS can be associated with changes in NT‐proBNP, distance according to the data of the 6‐min walk test, TAPSE, and LV GLS; (iii) whether there is a relationship with the data obtained with the help of right heart catheterization (RHC) and LV GLS/VSS ratio. Both groups were comparable in terms of gender and age. In the group of patients with ILAH, the average pressure in the pulmonary artery was 57.0 ± 11.4 mmHg, pulmonary vascular resistance (PVR) was 971.3 ± 451.9 dynes/s/cm⁵ and cardiac index (CI) 2, 4 ± 0.8 L/min/m². Compared with the control group, patients with IPAH had a higher rate of LV EF (64.9 ± 5.9 vs. 61.2 ± 3.7, *p* < 0.019), LV EF (37.7 ± 13.8 vs. 22.1 ± 5.0, *p* < 0.01) and a decrease in LV GLS (−17.5 ± 4.2% vs. −21.6 ± 2.1, *p* < 0.01). Correspondingly, the LV GLS/VSS ratio was reduced in patients with IPAH compared to controls (±0.53 ± 0.28 vs. 1.03 ± 0.32, *p* < 0.01). According to the data of the correlation analysis, the LV GLS/VSS ratio was reliably associated with the clinical characteristics of the patients: the distance covered according to the data of the 6‐min walk test (*r* = 0.48, *p* < 0.01); level of NT‐proBNP (*r* = −0.56, *p* < 0.01). Also, associations were found with hemodynamic indicators according to RHC: Cardiac output (CO) (*r* = 0.56, *p* < 0.01), CI (*r* = 0.51, *p* < 0.01). According to our study, the ratio of LV GLS/VSS significantly differed between the IPAH group and the control group; we found significant associations between this index and the level of NT‐proBNP, the distance traveled according to the data of the 6‐min walk test and the parameters of right heart catheterization—CO and CI. In the future, further studies on a larger sample of patients are possible, which will help to better reveal the above‐described indicators and integrate them into clinical practice.

## A027 P300/CBP INHIBITION: A DUAL STRATEGY TO COMBAT PAH LUNG AND RV REMODELING

Alice Bourgeois^1^, Sarah‐Eve Lemay^1^, Yann Grobs^1^, Charlotte Romanet^1^, Charlie Théberge^1^, Mélanie Sauvaget^1^, Sandra Martineau^1^, Sandra Breuils‐Bonnet^1^, François Potus^1^, Steeve Provencher^1^, Olivier Boucherat^1^, Pr Sebastien Bonnet^1^

^1^Quebec Heart And Lung Institute ‐ Laval University, Québec, Canada

Pulmonary arterial hypertension (PAH) is a vascular remodeling disease characterized by increased pulmonary artery (PA) pressure and vascular remodeling of distal PAs, partly due to excessive proliferation and survival of PA smooth muscle cells (PASMCs). Initially, RV hypertrophy allows adaptation (compensation) to maintain cardiac output. However, as the disease progresses, maladaptive remodeling (decompensation) inevitably occurs, leading to RV failure and premature death. Current therapies aim to promote vasodilation, but none of them directly targets lungs or RV pathological remodeling. The histone acetyltransferases P300/CBP have been identified as central players driving gene expression in various cellular processes such as proliferation/apoptosis and hypertrophy/fibrosis, all of which are critical features of pathological lung and RV remodeling in PAH. Given their role in controlling gene transcription programs, we hypothesized that P300/CBP contributes to maladaptive remodeling observed in PAH and that their inhibition could reverse this pathological phenotype. We show by western blot (WB) and immunofluorescence (IF) increased P300 expression in isolated PASMCs and distal PAs from PAH patients compared to controls (*p* < 0.01) as well as in monocrotaline (MCT) and sugen‐hypoxia rat models (*p* < 0.05). Similarly, P300 expression is upregulated in compensated and decompensated RV from PAH patients, MCT—and pulmonary artery banding (PAB)—subjected rats (WB, *p* < 0.05). In vitro, pharmacological (CCS‐1477) or molecular (siRNA) inhibition of P300/CBP reduces H3K27 acetylation (WB, *p* < 0.05) in PAH‐PASMCs and RV fibroblasts stimulated with TGF‐β. This effect was accompanied by a decrease in PAH‐PASMC proliferation and resistance to apoptosis (WB: PCNA, PLK1, Survivin; IF: Ki67, AnnexinV, *p* < 0.01). Similarly, inhibition of P300/CBP reduces TGF‐β‐induced proliferation (WB PCNA, Survivin, *p* < 0.05), activation and extracellular matrix production (WB: pSMAD2/3, αSMA, Fn, Col1, MMP2, *p* < 0.05) in isolated RV fibroblasts. P300/CBP inhibition prevents phenylephrine‐induced hypertrophy in isolated adult rat cardiomyocytes and H9C2 cells (IF: F‐actin, *p* < 0.05). Interestingly, adult rat cardiomyocytes isolated from MCT rats preserve their hypertrophied phenotype in vitro, which is reversed upon P300/CBP inhibition (IF: F‐actin, *p* < 0.05). In vivo, administration of CCS‐1477 significantly improves hemodynamics in MCT rat with established PAH (CO, RVSP, mPAP, *p* < 0.05), reduces pulmonary vascular remodeling (Elastica Van Gieson, *p* < 0.05) and RV fibrosis (Masson's Trichrome, *p* < 0.05). In the PAB rat model, P300/CBP inhibition attenuates the progression of RV failure (improved cardiac output, TAPSE, RVEDP, *p* < 0.01), diminishes RV fibrosis (Masson's Trichrome, *p* < 0.05) and RV cardiomyocyte surface area (H&E, *p* < 0.01). Our finding identified P300/CBP inhibition as a promising therapeutic avenue that could tackle both lung and RV maladaptive remodeling in PAH.

## A028 PRESCRIBING RIOCIGUAT FOR CTEPH PATIENTS: CAN WE EVALUATE PRACTICE FOR MORE SUSTAINABLE HEALTHCARE?

Rebecca Burney^1^, Frances Varian^1,2^, Ze Ming Goh^1,2^, Alexander Rothman^1,2^

^1^Sheffield Teaching Hospitals NHS Trust, Sheffield, UK, ^2^Department of Infection, Immunity and Cardiovascular Disease, University of Sheffield, Sheffield, UK

The World Health Organisation identified environmental factors such as climate change causing 12.6 million deaths globally in 2012, highlighting the need for global health initiatives. The NHS is responsible for 25 million tonnes of carbon emissions a year of which 20% is attributed to medicines and 5% patient travel. Evaluating local practices are key to achieving sustainable healthcare. This study aimed to assess feasibility in estimating the carbon cost of riociguat treatment for patients with CTEPH in a large specialist pulmonary hypertension centre (Sheffield Pulmonary Vascular Disease Unit) to guide sustainable practice initiatives. Retrospective review of the ASPIRE Registry (REC 16/YH/0352) was undertaken to identify patients with CTEPH commenced on riociguat between July 2015 and January 2023. The estimated carbon footprint was calculated for median distance travelled to appointments, medication wastage from unplanned cessation of therapy and outpatient appointments. Patient travel emissions were estimated based on the Gov.uk greenhouse gas conversion factors 2022. The impact of medication delivery was calculated from carbon emissions per parcel delivered by Royal Mail 2022–23 (218 kgCO2e) and carbon costings estimated using the Sustainable Healthcare Coalition (SHC) and Carbonfootprint calculator for travel. A total of 75 patients with a formal CTEPH diagnosis were prescribed riociguat: 34 male (45%) and 41 female (54%) with an average age of 75 years (IQR 63–80 years). The treatment duration was 2.2 years (IQR 4.3 months–5.2 years). Riociguat has an annual cost of £26,000, and therefore an average treatment cost of 10.3 tonnesCO2e. Excess medication incurs an estimated 0.39 tonnesCO2e per 1 month's supply. 72% of patients reached a maximum tolerated dose and 22 patients (29%, average 3/annum) stopped treatment due to ‘side effects’, resulting in a potential annual medication waste cost of 1.17 tonnesCO2e (0.39 tonnesCO2e per month's supply). Median travel distance from home to the hospital was 101 km (range 5–472 km). Average return car travel is estimated 0.04 tonnesCO2e (range 0–0.16 tonnesCO2e). Remote initiation and follow‐up remote appointments (*n* = 10) could save an average 0.08 tonnesCO2e/annum. The average annual carbon cost of treating a patient with riociguat including an annual outpatient appointment, car travel, and medication including delivery, is 4.94 tonnesCO2e per 12‐month treatment. The majority of carbon emissions are from medication and delivery (with one box supplying 14 days at £997.36). Calculators for carbon emissions are increasingly available. They reveal a significant environmental cost associated with medical therapy, even on a small scale. Areas to focus improvements include reducing medication waste and unnecessary travel. On a patient level, the environmental burden imposed from treatment makes achieving a low‐carbon lifestyle (typically 5–7 tonnes of CO2eq/cap/year) challenging. Estimates suggest patients on riociguat, receiving specialist care, would not be able to offset associated carbon costs even with modification of lifestyle to a vegan diet (reducing emissions by 1.55 tonnes of CO2eq/cap/year), not owning a car and not flying. Systematic service evaluation to identify and develop sustainable healthcare initiatives is recommended.

## A029 INVESTIGATING PULMONARY ARTERY VASCULAR CELL DYNAMICS AT PHYSIOLOGICAL AND PATHOLOGICAL OXYGEN TENSIONS

Mercè Rossinyol Boladeres^1^, Anika Miah^1^, Joose Kreutzer^2^, Veronica Carroll^1^

^1^St George's University of London, London, UK, ^2^BioGenium Microsystems Ltd, Tampere, Finland

Chronic hypoxia is thought to be a primary driver of vessel remodelling in the pulmonary vasculature in lung diseases which can lead to pulmonary hypertension (PH) and, ultimately, right heart failure. Phenotypic changes to pulmonary vascular cells that occur in hypoxia are mediated by the transcription factors, hypoxia‐inducible factors, HIF‐1 and HIF‐2. HIFs regulate genes involved in cellular processes important in vessel remodelling such as endothelial to mesenchymal transition, smooth muscle cell plasticity and fibrosis. However, the complexity of the regulation of vascular remodelling in PH is still poorly understood, particularly at the interface between physiological and pathological oxygen tensions and dependent on the differential stabilisation of HIF‐α subunits. To investigate pulmonary artery vascular cell dynamics, we are developing an ex vivo cell model that recapitulates lung oxygen levels using an Oxygenie portable hypoxic platform which is compatible with time‐lapse microscopy. Using this system, we can study pulmonary artery endothelial and smooth muscle cell motility, growth, and proliferation under different oxygen conditions by real‐time imaging. Experiments were performed in the absence and presence of TGF‐β and PDGF, which are established triggers of remodelling events in PH. This work aims to address the lack of cell models investigating vascular cell dynamics in conditions that mimic either physiological or pathological oxygen tensions to better understand abnormal vascular remodelling in PH.

## A030 EFFECTS OF PHYSICAL TRAINING ON ELECTRON TRANSPORT CHAIN IN A MOUSE MODEL OF PULMONARY ARTERIAL HYPERTENSION

Kelly Casós Vásquez^1^, Irene Gómez^1^, Olga Tura‐Ceide^1,2,3^, Joan Albert Barberà^1,2^, Isabel Blanco^1,2^, Victor Ivo Peinado^1,2,4^

^1^Department of Pulmonary Medicine, Servei de Pneumologia, Hospital Clínic‐Institut d'Investigacions Biomèdiques August Pi I Sunyer (IDIBAPS), University of Barcelona, Villarroel, Barcelona, Spain, ^2^Biomedical Research Networking Centre on Respiratory Diseases (CIBERES), Madrid, Spain, ^3^Department of Pulmonary Medicine, Josep Trueta Universitary Hospital de Girona, Santa Caterina Hospital de Salt and the Girona Biomedical Research Institute (IDIBGI), Parc Hospitalari Marti I Julià, Edifici M2, C/Castany s/n, Girona, Spain, ^4^Department of Experimental Pathology, Institut d'Investigacions Biomèdiques de Barcelona (IIBB), CSIC‐IDIBAPS, Barcelona, Spain

Pulmonary arterial hypertension (PAH) is a progressive disease characterized by elevated pulmonary vascular resistance leading to right heart failure. Exercise training has been shown to improve quality of life, exercise capacity, lower limb muscle strength, and possibly hemodynamics in PAH patients. Recent research has increasingly identified metabolic and bioenergetic alterations as consistent features of PAH, particularly with a greater association with increased oxidative stress, involving lipid oxidation. Importantly, considering that the main source of free radicals responsible for oxidative stress is mitochondrial respiration and given that exercise is a recognized stimulator of mitochondrial biogenesis in skeletal muscle, it is likely that compensatory mechanisms operate within the lung. In this context, we hypothesize that the benefits of exercise in the lungs could involve a partial inhibition of the electron transport chain (ETC) complexes (CI–CV), potentially leading to a shift in the phosphorylative phenotype of ATP production resulting in a lower lipid peroxidation. To investigate this hypothesis, we conducted experiments on a mouse model of PAH exposed to SU5416+hypoxia. Concomitantly, half of the animals were then subjected to a moderate exercise program (45 min daily). Therefore, our study included four groups: two control groups (exercise (CE) *n* = 6 and sedentary (CS) *n *= 6) and two PAH groups (exercise (HE) *n* = 6 and sedentary (HS) *n* = 6). Systolic pulmonary arterial pressure (sPAP) was determined by right heart catheterization using a pressure transducer (Millar). ETC complexes were simultaneously examined in lung, heart, and skeletal muscle homogenates through western blot analysis. At the end of the study, HE animals showed lower sPAP compared with HS (*p* = 0.005) without changes in body weight. In the lungs, we observed a decrease in the expression of Complex III (CIII) in the CE versus HE groups (*p* = 0.038) and in the HS versus HE groups (*p* = 0.034). Additionally, there was a decrease in Complex V (CV) expression when comparing CE and HE groups (*p* = 0.045) within the ETC. Similarly, in the heart, there was a decrease in all ETC complexes (CI–CV) when comparing the following groups: CI: CE versus HE (*p* = 0.015); CII: CE versus HE (*p* = 0.02); CIII: CE versus HE (*p* = 0.012) and HS versus HE (*p* = 0.045); CIV: CE versus HE (*p* = 0.006); and CV: CE versus HE (*p* = 0.034). Interestingly, in skeletal muscle, we observed a trend towards an increase in CV levels in the HE group, reaching levels like those of the CS group. Our results suggest that exercise may partially inhibit the ETC in the lungs and the heart, but not in the skeletal muscle, which has different energy demands. This inhibition in the lungs and heart could be beneficial in PAH as it would lead to reduced lipid accumulation in the mitochondrial matrix, resulting in lower generation of NADH and FADH2 cofactors in the Krebs cycle, aligning the ETC with the energy demands of PAH. However, further studies are needed to fully understand the role of exercise on mitochondrial function in the context of PAH.

## A031 MINIMAL IMPORTANT DIFFERENCE OF NT‐PROBNP IN PULMONARY ARTERIAL HYPERTENSION

Lucas Celant^1,2^, Lilian J Meijboom^2,3^, HJ Bogaard^1,2^, Frances S de Man^1,2^, Anton Vonk Noordegraaf^1,2^

^1^Department of Pulmonary Medicine, Amsterdam UMC location Vrije Universiteit Amsterdam, the Netherlands, ^2^Amsterdam Cardiovascular Sciences, Pulmonary Hypertension and Thrombosis, the Netherlands, ^3^Department of Radiology and Nuclear Medicine, Amsterdam UMC location Vrije Universiteit Amsterdam, the Netherlands

In pulmonary arterial hypertension (PAH), N‐terminal pro B‐type natriuretic peptide (NT‐proBNP) reflects right ventricular structure and function. Elevated levels indicate increased wall stress and have shown to harbor prognostic information. In clinical trials, NT‐proBNP is therefore often utilized as secondary endpoint, but the magnitude of a relevant change is unknown. What is a clinically relevant change of NTproBNP in PAH? 135 incident, treatment naïve PAH‐patients with available NT‐proBNP and cardiac magnetic resonance (CMR) imaging at baseline and follow up were included. Stroke volume (SV) was used as anchor to establish the minimal important difference (MID) of NT‐proBNP. Patients were divided in two groups based on their treatment response: (1) patients with an increase in SV of at least 10 mL; (2) patients whose SV remained stable or did not increase beyond 10 mL. Statistical approaches included: the optimal cutoff point on receiver operating curve (ROC) analysis, mean change in NT‐proBNP between groups and bivariate linear regression to correct for baseline covariates. Upon treatment, 52 patients had an increase in stroke volume of at least 10 mL (SV ≥ 10 patients) compared to 83 who failed to do so (SV < 10 patients). Groups were similar in terms of gender (70% female), age (53 yrs) and disease severity (PVR: 930 [604, 1182] vs. 633 [453, 903] dynes.s.cm^−5^, *p* > 0.05). Both groups were mostly started on oral combination therapy. Right ventricular functional impairment was most pronounced in SV ≥ 10 patients (32 ± 12% vs. 40 ± 12%, *p* < 0.001), but greatly improved to a similar level after treatment initiation (49 ± 11% vs. 45 ± 14%, pint  <0.001). Naturally, SV ≥ 10 patients had a greater increase in SV (17 mL/m^2^; 26 ± 8 to 43 ± 10 mL/m^2^) compared to SV < 10 patients (2 mL/m^2^; 32 ± 9 to 34 ± 9 mL/m^2^). NT‐proBNP levels at baseline were greater for SV ≥ 10 patients (1529 [432, 2847] vs. 603 [182, 1829] pg/mL). Overall, the MID range between methods is small and were as follows: ROC (−78%) and mean change difference (−81%). Multivariate adjustment showed that the MID did not differ by age, gender, PAH etiology, NYHA functional class and indexed right ventricular end diastolic volume (−73%). The MID of NT‐proBNP to identify patients with an increase of at least 10 mL in SV was approximately −80% using anchor‐based approaches.

## A032 NT‐PROBNP AS AN EARLY MARKER FOR FUTURE DISEASE PROGRESSION IN PULMONARY ARTERIAL HYPERTENSION PATIENTS

Lucas Celant^1,2^, Gabriel Stulnig^4^, Pavel Smirnov^4^, Lilian Meijboom^2,3^, Denys Wahl^4^, HJ Bogaard^1,2^, Frances de Man^1,2^, Anton Vonk Noordegraaf^1,2^

^1^Department of Pulmonary Medicine, AmsterdamUMC, Location Vumc, Amsterdam, The Netherlands, ^2^Amsterdam Cardiovascular Sciences, Pulmonary Hypertension and Thrombosis, The Netherlands, ^3^Department of Radiology and Nuclear Medicine, Amsterdam UMC location VUmc, The Netherlands, ^4^Janssen Pharmaceutica NV, Beerse, Belgium

A subset of PAH‐patients exists that are long‐term survivors (>5 years survival without lung transplantation (LungTx)). In these patients, it is often difficult to predict clinical worsening and detect early signs of disease progression. N‐terminal pro–brain‐type natriuretic peptide (NT‐proBNP) is a validated biomarker that reflects right ventricular (RV) structure and harbors prognostic information in PAH. The prognostic value of NT‐proBNP in long‐term survivors is currently unknown. We hypothesize that NT‐proBNP can detect early signs of future disease progression in PAH patients. Incident, treatment naïve PAH‐patients with available NT‐proBNP and cardiac magnetic resonance (CMR) imaging at baseline and follow‐up (FU) were included. True or false clinical stability was defined by the occurrence of death or lungTx after a minimal observational time >5years. We identified 60 patients, of which *N* = 16 deceased or received lungTx (false stable), while *N* = 44 patients did not (true stable). FU time for false stable patients was 7 years [IQR:6.4–7.8] and 8.5 years [IQR:6.5–10.5] for true stable patients. False stable patients were older (61 ± 15 vs. 45 ± 16 years) and more often male (50% vs. 23%), but displayed similar disease severity at baseline (pulmonary vascular resistance; 768 ± 315 vs. 669 ± 295 dyn·s·cm^−5^). At first FU, RV size between groups was similar (right ventricular end‐diastolic volume index: 77 ± 21 vs. 77 ± 27 mL/m^2^), but false stable patients had higher NT‐proBNP values (483 ng/L [IQR:234–777] vs. 132 ng/L [IQR:68–299], *p* < 0.01), lower stroke volume (SV) (32 ± 8 vs. 39 ± 11 mL/m^2^, *p* = 0.03) and lower 6‐min walking distance (6MWD) (428 ± 91 m vs. 536 ± 114 m, *p* < 0.01). Treatment regimens were similar, however, three true stable patients received upfront triple therapy (3 vs. 0). Univariate predictors (*p* < 0.10) by logistic regression analyses at first FU for future death/lungTx were: NT‐proBNP, age, gender, SV, right ventricular ejection fraction, and 6MWD. In multivariate logistic regression analyses, only gender, 6MWD, and SV remained significant (*p* < 0.05). NT‐proBNP levels in early disease course were different in false stable versus true stable PAH patients.

## A033 PULMONARY ENDOTHELIAL AK4: AN ESSENTIAL REGULATOR IN VASCULAR REMODELLING AND PULMONARY HYPERTENSION DEVELOPMENT?

Anis Čilić^1^, Vanessa Kleinhenn^1^, Edma Loku^1^, Astrid Weiss^1^, Natalia Bakalarska^2^, Friedrich Grimminger^1^, Ralph T Schermuly^1^, Hossein Ardeschir Ghofrani^1^, Natascha Sommer^1^, Stefan Hadžić^1^, Norbert Weissmann^1^, Christine Veith‐Berger^1^, Cheng‐Yu Wu^1^, Magdalena Wujak^2^

^1^Universities of Giessen and Marburg Lung Center (UGMLC), Member of the German Center for Lung Research (DZL), Excellence Cluster Cardio‐Pulmonary Institute (CPI), Justus‐Liebig University, Giessen, Germany, ^2^Department of Medicinal Chemistry, Collegium Medicum in Bydgoszcz, Faculty of Pharmacy, Nicolaus Copernicus University in Toruń, Bydgoszcz, Poland

Pulmonary hypertension (PH) is a life‐threatening and incurable disease affecting millions of people worldwide. A hallmark of PH involves alterations in the pulmonary vasculature, characterised by hyper‐proliferation of pulmonary arterial smooth muscle cells (PASMC) and dysfunction of endothelial cells manifested among others by uncontrolled proliferation and a glycolytic shift. These vascular remodelling processes lead to vessel lumen obliteration, which subsequently elevate pulmonary vascular resistance and pulmonary arterial pressure. Moreover, as PH advances, patients are at risk of developing cor pulmonale and potentially right heart failure. Previously, we reported increased adenylate kinase 4 (AK4) expression in the pulmonary vessels of patients with idiopathic pulmonary arterial hypertension (IPAH). In addition to PASMC, our preliminary data point toward the localisation of AK4 in endothelial cells of remodelled pulmonary vessels. Furthermore, AK4 expression was regulated by hypoxia‐inducible factor‐1 alpha (HIF‐1α) and may be responsible for the HIF‐1α‐dependent glycolytic shift in hypoxic PASMC. However, the role of AK4 in pulmonary endothelial cells remains poorly understood. Against this background, an in vitro model of PH was employed to study the AK4 expression regulation in pulmonary microvascular endothelial cells (PMVEC) challenged with hypoxia (1% O_2_) for different time points. The results showed that AK4 was upregulated under hypoxic conditions in a HIF‐1α‐dependent manner. Seahorse XF extracellular flux analyses demonstrated that AK4 was involved in endothelial glycolysis and mitochondrial respiration. Kinome profiling further revealed that AK4 silencing altered activities of Src family kinases (SFK), cyclin‐dependent kinases (CDKs), and mitogen‐activated protein kinases in hypoxic PMVEC. Strikingly, using a two‐dimensional co‐culture system, we found that AK4 loss‐of‐function in PMVEC reduced the proliferation of PASMC. In summary, our results suggest that AK4 in pulmonary endothelial cells plays a role in the regulation of glucose metabolism, protein kinase signalling pathways, and PMVEC‐PASMC crosstalk, potentially contributing to PH development. Further studies will focus on evaluating the impact of endothelial‐specific AK4 deletion using a mouse model of hypoxia‐induced PH, with the aim to propose a new therapeutic approach. This research was supported by: The German Research Foundation (DFG) (Project‐ID 268555672, SFB 1213: A06, A07, A08, A10, B04), The Excellence Cluster Cardio‐Pulmonary Institute (CPI) (EXC2026, Project ID:390649896) and partially by the National Science Centre (Poland) [grant No. 2021/05/X/NZ1/00657] and The Initiative of Excellence—Research University Programme [grant No. 150/2022/Grants4NCUStudents].

## A034 IMPROVEMENT OF PULMONARY ARTERIAL HYPERTENSION RISK ASSESSMENT MODEL USING CARDIAC MAGNETIC RESONANCE IMAGING VARIABLES

Priscilla Correa‐Jaque^1^, Yeming Lin^1^, Shili Lin^1^, Yongqi Liu^1^, Charles Fauvel^2^, Rebecca Vanderpool^1^, Manreet Kanwar^3^, Jidapa Kraisangka^4^, Adam Perer^5^, Allen Everett^6^, Samer Alabed^7^, Andrew Swift^7^, David Kiely^7^, Raymond Benza^8^

^1^The Ohio State University, Columbus, USA, ^2^Rouen University Hospital, Rouen, France, ^3^Allegheny Health Network, Pittsburgh, USA, ^4^Mahidol University, Salaya, Thailand, ^5^Carnegie Mellon University, Pittsburgh, USA, ^6^The John Hopkins University, Baltimore, USA, ^7^University of Sheffield Medical School, Broomhall, UK, ^8^Icahn School of Medicine at Mount Sinai, New York City, USA

Pulmonary arterial hypertension (PAH) remains a deadly disease without cure. Periodic formalized risk stratification allows personalized therapeutic adjustments based on individual risk that optimizes drug utilization with the promise of mitigating morbid and mortal outcomes. Contemporary risk scores, like REVEAL 2.0, are recommended for use in PAH guidelines but only offer good discrimination (C‐Indexes 0.7–0.8). The PHORA project's goals are to create risk models with excellent discrimination (C‐Index > 0.8), using modern statistical techniques and expanded variable pools including imaging and genomics. The AIM of this study was to demonstrate the improved performance with the addition of cardiac MRI variables. PAH patients from the ASPIRE (Assessing the Spectrum of Pulmonary Hypertension Identified at a Referral Center) cardiac MRI database were analyzed. We performed imaging variable selection using three types of machine learning methods: logistic regression, Lasso, and Random Forest. Clinicians' inputs were also solicited. Rankings of the imaging variables from these sources were aggregated to arrive at a consensus list. The selected imaging variables were added to the set of variables for deriving the REVEAL 2.0 composite score then built Bayesian networks (BN) to predict 1‐year survival based on the Tree‐Augmented naïve Bayes (TAN) algorithm. Five‐fold cross‐validation was performed to assess the improvement in survival prediction from adding the selected imaging variables. A total of 343 PAH prevalent subjects were included in this analysis. The rank aggregation algorithm identified several imaging variables, including LVSVI, RVCO, LVEDVI, and RVESVI, that were predictive of survival but not the REVEAL 2.0 composite score. Adding these imaging parameters to the REVEAL 2.0 variables, we build a BN model that depicts the nonlinear relationships among the predictors and 1‐year survival (Fig. 1). We obtained an average AUC of 0.83 over the five cross‐validation test sets, an improvement over the AUC of 0.78 using only the REVEAL 2.0 variables. A Mann–Whitney non‐parametric test shows that the improvement is statistically significant at the 0.1 level. Using advanced statical models that include cardiac imaging variables improves the performance of PAH risk assessment models.

## A035 RISK STRATIFICATION IN PULMONARY VENO‐OCCLUSIVE DISEASE

Alejandro Cruz‐Utrilla^1^, Carmen Pérez‐Olivares Delgado^2^, Carmen Jiménez López‐Guarch^1^, Andrés Quezada‐Loaiza^1^, Pedro Bedate^3^, Amaya Martínez‐Meñaca^4^, Manuel López Meseguer^5^, Fernando Arribas‐Ynsaurriaga^1^, Pilar Escribano‐Subias^1^

^1^Pulmonary Hypertension Unit, Cardiology Department, Hospital Universitario 12 de Octubre, Madrid, Spain. Instituto de Investigación Hospital 12 de Octubre (i+12), Madrid, Spain. ERN‐LUNG., Madrid, Spain, ^2^Department of Cardiology, Hospital Del Henares, Coslada, Spain, ^3^Department of Pulmonary Medicine, Hospital Universitario Central de Asturias, Oviedo, Spain, ^4^Department of Pneumology, Hospital Universitario Marqués de Valdecilla. Santander. Spain. ERN‐LUNG. Instituto de Investigación Valdecilla (IDIVAL), Santander, Spain, ^5^Department of Pulmonary Medicine, Hospital Universitario Vall d'Hebron, Barcelona, Spain. ERN‐LUNG. Centro de Investigación Biomédica en Red de Enfermedades Respiratorias (CIBERES), Instituto de Salud Carlos III, Madrid, Spain, Barcelona, Spain

Pulmonary arterial hypertension (PAH) is a rare and severe disease, leading to death if untreated. This risk of death is influenced by several variables. European and American Guidelines for PAH recommend the use of multiparametric models for risk stratification. Pulmonary veno‐occlusive disease (PVOD) is a particularly infrequent form of PAH, having an especially low survival. There is no evidence confirming the applicability of risk scores used for PAH specifically in PVOD. Our aim in this study was to describe the applicability of the baseline ESC/ERS and the REVEAL Lite 2 scores at baseline in patients with PVOD. We considered patients with PVOD included in the Spanish registry of PAH (REHAP) between 2011 and 2022. The diagnosis of heritable PVOD required a homozygous variant in EIF2AK4. Patients with sporadic PVOD had a definitive pathological diagnosis or the presence of 3 over 3 radiological signs of PVOD (septal lines, ground glass opacities, and mediastinal lymphadenopathy). All cases required a diagnostic right heart catheterization (RHC). Both the REVEAL Lite 2 and the ESC/ERS scores were applied in this population at baseline. Goodness‐of‐fit and calibration of the models were evaluated. All participants signed informed consent before entering the REHAP registry. The study was conducted following the Declaration of Helsinki and after approval by the local Ethics Committee. In the study period up to 32 cases of heritable PVOD in 23 unrelated families were collected. Six cases of sporadic PVOD were confirmed histologically, and other 6 cases were diagnosed as probable PVOD by means of the radiological findings. After a median follow‐up of 22.1 months, up to 18 patients with PVOD needed lung transplantation (40.9%), and 12 patients died (27.3%). The observed rate of death at 1, 3, and 5 years in PVOD was 5.3%, 22.9%, and 50.5%, respectively. Most of the included population had a high risk of mortality after the application of the REVEAL Lite 2 (63.6%). Those patients at intermediate risk had a hazard ratio of 2.97 (0.27–33.03, *p* = 0.376) when compared with patients at low risk, and those patients at high risk had a hazard ratio of 8.49 (1.02–79.89, *p* = 0.048) when compared with patients at low risk. The application of the score at diagnosis in patients with PVOD demonstrated a good global prognostic capacity (C Index of 0.694), and the calibration of the model in this population was modest. On the other hand, the intermediate‐risk stratum was the most frequent one after the application of the European model (20 cases, 62.5% of the cohort), and only one case (3.1%) was categorized as having high‐risk. There was also a good global prognostic capacity (C Index of 0.707). Intermediate‐risk patients had a hazard ratio of 9.53 (2.15–42.17, *p* = 0.003) when compared with patients at low risk, and patients at high‐risk had a hazard ratio of 9.49 (0.86–104.69, *p* = 0.066) when compared with patients at intermediate risk. REVEAL Lite 2 and ESC/ERS models could be equally useful in PVOD. Further multicentre studies are needed in this population.

## A036 CLASSIFICATION, DIAGNOSIS AND TREATMENT STATUS OF PULMONARY HYPERTENSION FROM 2012 TO 2019: A SINGLE CENTER STUDY IN YUNNAN PROVINCE

Hai‐long Dai^1^, Xiao‐Lan Feng^1^, Yi‐Bing Lu^1^, Dong Yang^1^, Qiang Xue^1^, Ji‐Lei Zhang^1^, Chun‐Rong Lin^1^, Pin Gan^1^, Wei‐Hua Zhang^1^, Xue‐Feng Guang^1^

^1^Yan'an Affiliated Hospital Of Kunming Medical University, Kunming, China

To analyze the classification, diagnosis, and treatment status of patients with pulmonary hypertension (PH) in Yunnan province. This was a retrospective study. Hospitalized patients with PH at Yan'an Affiliated Hospital of Kunming Medical University from January 2012 to December 2019 were enrolled. The composition ratio of PH, diagnosis, and treatment were analyzed. A total of 13 590 patients with PH were enrolled, accounting for 3.09% (13 590/440 056) of the total number of hospitalizations during the same period. The composition of PH was predominantly pulmonary arterial hypertension (PAH) (55.50% (7542/13 590)), followed by pulmonary hypertension (PH) caused by left heart disease (24.16% (3284/13 590)). Among them, PAH could be subdivided into four types: idiopathic pulmonary arterial hypertension (IPAH), PAH associated with connective tissue disease, PAH associated with portal hypertension, and PAH associated with congenital heart disease (CHD‐PAH), with CHD‐PAH as the predominating type (98.09% (7398/7542). Patients with PAH were predominantly adolescents. In hospitalized patients with PH, from 2012 to 2019, the proportion of children and adolescents showed a decreasing trend from year to year, and the proportion of middle‐aged and older adults showed a significant increasing trend, and the proportion of female patients showed a gradual decreasing trend, and the proportion of patients with comorbid hypertension, diabetes mellitus, coronary artery disease, arrhythmia, and pneumonia showed an increasing trend. A total of 1034 patients (7.61% (1034/13 590)) underwent right heart catheterization. The concordance rate between echocardiographic and right heart catheterization findings was (86.98% (875/1006)). A total of 2574 (18.94%) of PH patients were treated with PAH‐targeted drugs, of which 58.16% (1497/2574) were treated with monotherapy. Among the PH patients treated with PAH‐targeted drugs, the majority of patients were PAH patients (86.44% (2225/2574)), and 83.53% (2150/2574) patients treated with PAH‐targeted drugs were CHD‐PAH. Hospitalized PH patients in our center between 2012 and 2019 are predominantly CHD‐PAH, and the proportion of patients receiving right heart catheterization and targeted drug therapy is relatively low. The percentage of middle‐aged and elderly PH patients shows an increasing trend from year to year, as well as the percentage of those with concomitant comorbidities.

## A037 LUNG CAPILLARY ENDOTHELIUM TO ARTERIAL ENDOTHELIUM TRANSITION IN PULMONARY ARTERIAL HYPERTENSION

Bin Liu, Dan Yi, Karina Ramirez, Xiaomei Xia, Hongxu Ding, Zhiyu Dai^1^

^1^University Of Arizona, Phoenix, USA

Pulmonary arterial hypertension (PAH) is characterized by a progressive increase of pulmonary vascular resistance and obliterative pulmonary vascular remodeling that result in right heart hypertrophy, failure, and premature death. The underlying mechanisms of loss of distal capillary endothelial cells (ECs) and obliterative vascular lesion formation remain unclear. Our recent single‐cell RNA sequencing, spatial transcriptomics analysis, RNASCOPE, and immunostaining analysis showed that arterial ECs accumulation and loss of capillary ECs were evident in human PAH patients and pulmonary hypertension (PH) rodents. Pseudotime trajectory analysis of the single‐cell RNA sequencing data suggest that lung capillary ECs transit to arterial ECs during the development of PH. Our study also identified CXCL12 as the marker for arterial ECs in PH. Capillary EC lineage tracing approach using capillary specific‐Dre;Tdtomato reporter mice demonstrated that capillary ECs gave rise to arterial ECs during PH development. Genetic deletion of HIF‐2a or pharmacological inhibition of Mediator kinase Cdk19 normalized the arterial programming in PH. In conclusion, our study demonstrates that capillary endothelium transits to arterial endothelium through the HIF‐2a‐Cdk19 pathway during the development of PAH. Thus, targeting arterial EC transition might be a novel approach for treating PAH patients.

## A038 MODELING HERITABLE PULMONARY ARTERIAL HYPERTENSION: GENERATION OF A BMPR2 HETEROZYGOUS SHEEP USING CRISPR

Sanjeev A. Datar^1^, Austin Brown^2^, Devon Fitzpatrick^2^, Rachel Hutchings^1^, Elena Amin^1^, Eric Johnson^2^, Jessica Morgan^2^, Hythem Nawaytou^1^, Omar Gonzales Viera^2^, Thomas Bishop^2^, Tara Urbino^2^, Bret McNabb^2^, Eric Austin^3^, Jeffrey Fineman^1^, Alison Van Eenennaam^2^

^1^University of California, San Francisco, San Francisco, USA, ^2^University of California, Davis, Davis, USA, ^3^Vanderbilt University, Nashville, USA

Monoallelic mutations in BMPR2, a serine/threonine kinase receptor in the TGF‐β superfamily, are the most common genetic risk factor for PAH, however BMPR2's role in the disorder's progression remains incompletely understood due in part to the lack of appropriate preclinical genetic models. Homozygous null mutations in BMPR2 are embryonic lethal; to overcome this barrier, a single guide RNA (sgRNA) CRISPR/Cas9 approach was used to edit sheep zygotes to create a monoallelic mutation in exon 3 of the ovine BMPR2 gene. Five to six edited blastocysts were transferred to each of eight recipient ewes resulting in seven pregnancies, one of which subsequently failed. Eight fetuses (two sets of twins and four singletons) were confirmed viable via ultrasound at 9.5 weeks, past the embryonic lethality period, with expected due dates in late July 2023. One male was stillborn 4 weeks prematurely, and one set of twins and one singleton died during labor. Four lambs, one male and three females, were born alive, and genomic sequence analysis of peripheral blood mononuclear cells confirmed that each lamb had one mutant allele for BMPR2 with an out of frame disruption of exon 3. By 3 weeks of age, one of the three females had developed right ventricular hypertrophy and septal flattening by echocardiography; a right heart catheterization at 5 weeks of age showed near systemic mean pulmonary artery pressure with a calculated pulmonary vascular resistance 130% of age‐matched controls. This lamb also had a patent ductus arteriosus (PDA), resulting in a Qp:Qs of 1.1 in 21% FiO_2_ and 1.7 in 100% FiO_2_. CT angiography (CTA) at 8 weeks of age confirmed cardiomegaly, and distended pulmonary arteries with a PA:Ao ratio of 1 (normal <0.8). CTA also identified cardiomegaly, PDA, and a PA:Ao ratio of 1 and 1.3 in the second and third females, respectively, who had normal findings by echocardiography. There was no radiographic evidence of peripheral pulmonary vascular pruning or pulmonary parenchymal changes. Tissue was collected from each heterozygote animal to determine if truncated BMPR2 transcript and/or protein was made. One female lamb was born with arthrogryposis of the forelimbs and mandibular brachygnathia and was eventually euthanized at 7 weeks of age due to osteomyelitis and pathologic fractures of the distal phalanx. Another female lamb developed progressive neuromuscular weakness by 6 weeks of age that was most pronounced in the hind limbs, and she was euthanized by 9 weeks of age. Tissue from each was sent for comprehensive histologic analysis. Upon reaching sexual maturity, semen from the male heterozygote will be collected and the sperm will be genotyped and tested for viability, and he will be bred with wildtype ewes to establish a reliable supply of BMPR2 +/− lambs for future studies. This innovative large animal model will facilitate sophisticated developmental and physiologic testing that includes advanced imaging, invasive cardiovascular measurements, and exercise testing. It will serve as a vital platform for mechanistic molecular studies that advance the field and will also provide a much‐needed preclinical model for extensive treatment evaluations.

## A039 PROTECTIVE EFFECTS OF NOXO1 DELETION IN LUNG CELL TYPES ON CIGARETTE SMOKE‐INDUCED PULMONARY HYPERTENSION AND EMPHYSEMA

Siddartha Doswada^1,2,3,4^, Cheng Yu Wu^1,2,3,4^, Edma Loku^1,2,3,4^, Friedrich Grimminger^1,2,3,4^, Hossein A. Ghofrani^1,2,3,4^, Ralph T. Schermuly^1,2,3,4^, Werner Seeger^1,2,3,4,7,8^, Baktybek Kojonazarov^1,2,3,4,5^, Ralf P. Brandes^6^, Norbert Weissmann^1,2,3,4^, rer.nat. Stefan Hadzic^1,2,3,4^

^1^Excellence Cluster Cardio‐Pulmonary Institute (CPI), Giessen, Germany, ^2^Universities of Giessen and Marburg Lung Center (UGMLC), Giessen, Germany, ^3^Member of the German Center for Lung Research (DZL), Giessen, Germany, ^4^Justus‐Liebig‐University‐Giessen, Giessen, Germany, ^5^Institute for Lung Health (ILH), Giessen, Germany, ^6^Institute for Cardiovascular Physiology, Goethe University, Frankfurt, Germany, ^7^Max‐Planck Institute for Heart and Lung Research, Bad Nauheim, Germany, ^8^Member of the German Center for Lung Research (DZL), Bad Nauheim, Germany

Chronic obstructive pulmonary disease (COPD) patients often suffer from at least mild pulmonary hypertension (PH) due to significant remodeling of the pulmonary vasculature. Chronic exposure to cigarette smoke (CS) is a known risk factor for the development of COPD‐PH. Moreover, it appears that cellular molecular alterations in the pulmonary vasculature with elevated nitrosative stress and oxidative stress precede the development of pulmonary emphysema in COPD. Our previous study identified NADPH oxidase organizer 1 (Noxo1) as the main source of oxidative stress during the development of CS‐induced emphysema and PH in the mouse COPD model. In this context, NOXO1‐dependent superoxide upregulation in the vascular and alveolar compartment could combine to trigger the peroxynitrite formation proposed to underlie PH and emphysema development. In our study, we investigate the protective effects of Noxo1 deletion on emphysema and PH development, specifically in three distinct cell populations within the lung: Tie2+ cells, CCSP+ cells, and Acta2+ cells. Noxo1flox/flox transgenic mice with ERT2Cre recombinase inserted under Acta2, Tie2, or CCSP promoter were used in this study. Noxo1 was deleted in the respective cell type before CS exposure. We exposed Wild type (Wt) and cell‐specific Noxo1 knock‐out animals to CS for 3 (PH development) or 8 (emphysema development) months. PH was diagnosed by noninvasive echocardiography and invasive hemodynamic measurements, while pulmonary emphysema was assessed by in vivo lung function tests and confirmed by histological analysis. To investigate the inflammatory mediators associated with CS‐induced PH, a cytokine assay was performed in bronchoalveolar lavage fluid (BALF) and lung homogenate of experimental animals. Mechanistic investigations reveal that Noxo1 deletion in Tie2+ or CCSP+ cells exerts protective effects against CS‐induced increase in right ventricular systolic pressure (RVSP) and right ventricular hypertrophy but not against CS‐induced emphysema. However, mice with Noxo1 deletion in Acta2+ cells were protected against CS‐induced emphysema but developed signs of PH similar to Wt mice. Our results collectively suggest that, during CS‐induced lung injury, NOXO1 in Tie2+ (mainly endothelial cells) or CCSP+ (epithelial cells) contributes to CS‐induced PH development and NOXO1 in Acta2+ (mainly smooth muscle cells) cells contributes to CS‐induced emphysema development. These findings highlight the potential of NOXO1 as a promising therapeutic target for the treatment of PH and underscore the importance of cell‐specific interventions. Further research is needed to delineate the molecular mechanisms of NOXO1‐mediated development of CS‐induced PH.

## A040 EXERCISE HAEMODYNAMIC SURROGATES OF RIGHT VENTRICULAR TO PULMONARY ARTERIAL (RV‐PA) UNCOUPLING AND THEIR CLINICAL RELEVANCE

Philipp Douschan^1,2^, Bruno Brita da Rocha^2^, Athiththan Yogeswaran^2^, Zvonimir Rako^2^, Nils Kremer^2^, Hossein A. Ghofrani^2,3,4^, Werner Seeger^2^, Manuel Richter^2^, Khodr Tello^2^

^1^Medical University of Graz and Ludwig Boltzmann Institute for Lung Vascular Research, Graz, Austria, ^2^Universities of Giessen and Marburg Lung Center (UGMLC), Member of the German Center for Lung Research (DZL), Institute for Lung Health (ILH), Excellence Cluster Cardio‐Pulmonary Institute (CPI), Justus‐Liebig‐University, Giessen, Germany, ^3^Department of Pneumology, Kerckhoff Heart, Rheuma and Thoracic Center, Bad Nauheim, Germany, ^4^Department of Medicine, Imperial College London, London, UK

Pathological pulmonary exercise haemodynamics are characteristics and prognostic markers of early pulmonary vascular disease (PVD). However, no data exists regarding the association of pulmonary exercise haemodynamics with right ventricular function and their clinical relevance in patients with manifest PH. We hypothesized that pulmonary exercise haemodynamics may be significantly associated with RV‐PA‐coupling at rest and during exercise. Moreover, they may serve as additional prognostic markers in patients with severe PH. In the EXERTION study (NCT04663217), we aimed to prospectively assess the association between pulmonary haemodynamics with right ventricular function at rest and during exercise in a continuum of patients with different stages of PVD. All patients underwent right heart catheterization (RHC) and pressure‐volume RHC at rest and during symptom‐limited exercise. Pressure‐volume curves were analysed to obtain Ees, Ea, and RV‐PA‐coupling (Ees/Ea). RV‐PA‐uncoupling was defined as Ees/Ea < 0.8. PH was defined as a mPAP > 20 mmHg. Exercise PH (EPH) was defined as an mPAP/CO‐slope > 3WU. Clinical worsening was obtained from all patients. Between 2020 and 2023, 65 patients without postcapillary PH were prospectively included (43 female, age 67 [57–74], mPAP 33 ± 15 mmHg, CI 2.7 [2.3–3.1], Ees/Ea 1.2 ± 0.4). The median follow‐up time was 22 [16–26] months with 21 events. Forty‐six patients fulfilled criteria for precapillary PH. Forty‐nine patients fulfilled the criteria for EPH. The best haemodynamic predictors for RV‐PA‐uncoupling at rest were PVRbase (AUC 0.83), RAP/CO‐slope (AUC 0.8) and mPAP/CO‐slope (AUC 0.8). The best predictors for RV‐PA‐uncoupling during exercise were RAP/CO‐slope (AUC 0.83), COpeak (AUC 0.83), and ΔRAP/CO‐ratio (AUC 0.79). PVRbase, COpeak and ΔRAP/CO‐ratio turned out as age and sex independent predictors of clinical worsening. To further assess the utility of these haemodynamic parameters to identify patients at additional risk for clinical worsening in an unbiased manner, tertiles for PVRbase (1st‐tertile: up to 224 dyn, 2nd‐tertile:224 dyn to 509 dyn, 3rd‐tertile: above 509 dyn), COpeak (1st‐tertile: up to 6.2 L/min, 2nd‐tertile:6.2 L/min to 8.8 L/min, 3rd‐tertile: above 8.8 L/min) and ΔRAP/CO‐ratio (1st‐tertile: up to 0.93 mmHg/L/min, 2nd‐tertile: 0.93 mmHg/L/min to 2.04 mmHg/L/min, 3rd‐tertile: above 2.04 mmHg/L/min) were generated. Only ΔRAP/CO‐ratio was able to identify a cluster of patients with manifest PH and significantly impaired outcome (1st‐ and 2nd‐tertile vs. 3rd‐tertile [*p* = 0.003]). There was a significant decline of Ees/Eapeak (1.36[IQR:1.15–1.78] vs. 1.26[IQR:1.05–1.46] vs. 0.79[IQR:0.59–0.98], *p* = <0.001), ΔEes (0.18[IQR:0.01–0.59] vs. 0.35[IQR:0.27–0.41] vs. 0.13[IQR:−0.1 to 0.35], *p* = 0.079), performance in watt/kg (0.52[IQR:0.31–0.67] vs. 0.31[IQR:0.21–0.48] vs. 0.21[IQR:0.1–0.36], *p* = 0.001) and peakVO2 (1.35 L/min[IQR:1.22–1.75] vs. 1.21 L/min[IQR:0.84–1.44] vs. 0.77 L/min[IQR:0.74–0.93], *p* = 0.002) from the 1st‐ to the 3rd‐tertile of the ΔRAP/CO‐ratio. In contrast, there was a significant increase in BNP (16 pg/mL[QR:10–40] vs. 67 pg/mL[IQR:27–110] vs. 247 pg/mL[118–441], *p* ≤ 0.001), REVEAL‐lite 2.0 score (4[IQR:4–5] vs. 6[IQR5–7] vs. 8[IQR:7–9], *p* < 0.001) and ESC‐risk‐score (1.4[IQR:1.2–1.5] vs. 1.6[IQR:1.5–1.8] vs. 2.1[IQR:1.9–2.4], *p* ≤ 0.001) dependent on ΔRAP/CO‐ratio. In this prospective study pulmonary exercise hemodynamics turned out as important surrogates of RV‐PA‐uncoupling at rest and during exercise. A ΔRAP/CO‐ratio greater than 2.04 mmHg/L/min was able to identify patients with manifest PH with lower exercise capacity, higher Reveal‐Lite 2.0‐ and the ESC‐risk‐score and worse prognosis. ΔRAP/CO‐ratio may serve as an additional prognostic parameter in patients with manifest PH at rest.

## A040 CT IMAGING FEATURES OF PULMONARY HYPERTENSION AND CHRONIC THROMBOEMBOLIC PULMONARY DISEASE PREDICT THE DEVELOPMENT OF CHRONIC THROMBOEMBOLIC PULMONARY HYPERTENSION IN PATIENTS PRESENTING WITH ACUTE PE: RESULTS FROM THE ASPIRE REGISTRY

Charlotte Durrington^1^, Judith Hurdman, Charlie Elliot, Rhona Maclean, Joost Van Veen, Giorgia Sacculloo, Duneesha De‐Foneska, Andrew Swift, Smitha Rajaram, Catherine Hill, Steven Thomas, Krit Dwivedi, Sameer Alabed, Jim Wild, Athanoasios Charlampopoulos, Abdul Hameed, Alexander Rothman, Lisa Watson, Neil Hamilton, AA Roger Thompson, Robin Condliffe, David Kiely

^1^Sheffield Teaching Hosptials NHS FT,  Sheffield, UK

Diagnostic rates and risk factors for the subsequent development of chronic thromboembolic pulmonary hypertension (CTEPH) following pulmonary embolism (PE) are not well defined. CTEPH may be present at the time of initial presentation but there is only limited data on the radiographic features at the time of acute PE in patients with a subsequent diagnosis of CTEPH. Over a 10‐year period (2010–2020), consecutive patients attending a PE follow‐up clinic in Sheffield (population 554,600) were included. CTPAs were reviewed from patients subsequently diagnosed with CTEPH and compared to a randomly selected group who did not develop CTEPH. Where all three CT features of pulmonary hypertension (PH) were present; pulmonary artery (PA) size ≥30 mm, right ventricular outflow hypertrophy (RVOTH) ≥6 mm and right ventricular:left ventricular ratio (RV:LV) ≥1 and at least two of four features of chronic thromboembolic pulmonary disease (CTEPD) (dilated bronchial arteries, arterial webs or bands, attenuated or occluded vessels and mosaic parenchymal perfusion pattern) were present, the patient was defined as having CTEPH at the time of the initial presentation. During the study period 20,494 patients from Sheffield underwent CTPA for suspected acute PE, 3289 patients (16%) were diagnosed with PE and 1956 (59%) of these patients were seen in the PE clinic 3–6 months following a diagnosis of acute PE. Forty‐one patients were subsequently diagnosed with CTEPH with a cumulative incidence of 2.10% and CTPAs were available for review in 36 patients. The presence of three CT features of pulmonary hypertension in combination with ≥2 of four features of chronic thromboembolic pulmonary disease (CTEPD) at the index PE was present in 19% of patients who developed CTEPH and in 0% of patients who did not. Whereas enlargement of the PA and an increase in RV:LV ratio were seen in 67% and 78% of patients who developed CTEPH compared to 31% and 36% of patients who did not develop CTEPH, respectively, RVOTH and all 3 features of PH were present in 44% and 36% of patients who developed CTEPH and in only 8% and 6% of patients who did not, suggesting that the presence of RVOTH and all 3 features of PH may aid identification of patients at significantly increased risk of developing CTEPH. CT features of PH and CTEPD are risk factors for a subsequent diagnosis of CTEPH. CT features of PH are easier to appreciate by the nonspecialist radiologist and physician compared to those of CTEPD and in contrast to features of CTEPD are also amenable to automated analysis using Artificial Intelligence approaches. This study highlights the potential for systematic analysis of the CTPA at the time of acute PE to aid early diagnosis and identifies CT features of PH as an important risk factor for CTEPH.

## A041 SOPHA‐STUDY: A PROSPECTIVE, RANDOMIZED, CONTROLLED TRIAL TO ASSESS THE EFFECT OF LONG‐TERM OXYGEN THERAPY ON CLINICAL PARAMETERS IN PATIENTS WITH PULMONARY ARTERIAL HYPERTENSION AND CHRONIC THROMBOEMBOLIC PULMONARY HYPERTENSION

Ishan Echampati^1,2^, Panagiota Xanthouli^1,2,3,4^, Satenik Harutyunova^1,2,3^, Christina Eichstaedt^1,2,5^, Benjamin Egenlauf^1,2,3^, Silvia Ulrich^5^, Ekkehard Grünig^1,2^, Nicola Benjamin^1,2,3^

^1^Centre for Pulmonary Hypertension, Thoraxklinik Heidelberg gGmbH, Heidelberg, Germany, ^2^Translational Lung Research Center Heidelberg (TLRC), Member of the German Center for Lung Research (DZL), Heidelberg, Germany, ^3^Department of Pneumology and Critical Care Medicine, Thoraxklinik Heidelberg gGmbH at Heidelberg University Hospital, Heidelberg, Germany, ^4^Department of Internal Medicine V: Hematology, Oncology and Rheumatology, University Hospital Heidelberg, Heidelberg, Germany, ^5^Laboratory for Molecular Genetic Diagnostics, Institute of Human Genetics, Heidelberg University, Heidelberg, Germany, ^6^Pulmonary Division and Sleep Disorders Center, University Hospital of Zurich, Zurich, Switzerland

Current guidelines recommend oxygen (O_2_) supplementation in patients with pulmonary hypertension (PH), despite scarce data on long‐term O_2_ administration (LTOT). The aim of this study was to investigate the effect of LTOT in patients with precapillary PH on exercise capacity, clinical parameters and hemodynamics. In this prospective, randomized, controlled trial, patients with precapillary PH under stable targeted therapy, experiencing O_2_ desaturations at rest and/or during exercise were randomly assigned to receive LTOT (≥16 h/day) or standard of care (SoC) for 12 weeks. To patients receiving SoC, LTOT was offered after 12 weeks (secondary intervention group). The primary endpoint was analysed in a hierarchical testing strategy of (1) the change of the distance walked in 6 min (6MWD) after 12 weeks of treatment in the primary and secondary intervention group; (2) the comparison of changes in the 6MWD baseline to 12 weeks in the intervention versus control group. Secondary endpoints included changes in clinical, laboratory and functional parameters, quality of life, and hemodynamics. A total of 20 patients (O_2_ *n* = 10 vs. SoC *n* = 10) were randomized (women *n* = 14, age 67 ± 11.4 years, mean pulmonary arterial pressure 39.7 ± 12.5 mmHg, 70% WHO functional class III). After LTOT treatment, a significant improvement in 6MWD of 42.2 ± 34.20 m (*p* = 0.003) at week 12 was measured compared to baseline. The intervention group significantly improved in 6MWD (38.9 ± 33.87 m) compared to the control group (−12.3 ± 21.83 m, *p* = 0.015). No consistent differences between groups in other clinical parameters were found. Two patients died during the study, one patient due to SARS‐CoV2 pneumonia in the O_2_ arm and one due to right heart failure in the SoC arm. These events were not associated with the study medication. Long‐term O_2_ therapy was well tolerated and resulted in a significant improvement of the primary endpoint 6MWD. The effect of LTOT should be further investigated in larger controlled‐trials.

## A042 EFFECT OF TREATMENT WITH AMBRISENTAN IN PATIENTS WITH SYSTEMIC SCLEROSIS AND MILD PULMONARY ARTERIAL HYPERTENSION: LONG‐TERM FOLLOW‐UP DATA FROM EDITA STUDY

Paul Uesbeck^1,2^, Nicola Benjamin^1,2,3^, Hanns‐Martin Lorenz^4^, Christina A. Eichstaedt^1^, Satenik Harutyunova^1,2,3^, Benjamin Egenlauf^1,2,3^, Ekkehard Grünig^1,2,3^, Panagiota Xanthouli^1,2,3,4^

^1^Thoraxklinik at Heidelberg University Hospital, Heidelberg, Germany, ^2^Translational Lung Research Centre Heidelberg (TLRC), Member of the German Centre for Lung Research (DZL), Heidelberg, Germany, ^3^Department of Pneumology and Critical Care Medicine, Thoraxklinik Heidelberg gGmbH at Heidelberg University Hospital, Heidelberg, Germany, ^4^Department of Internal Medicine V: Hematology, Oncology and Rheumatology, University Hospital Heidelberg, Heidelberg, Germany, ^5^Laboratory for Molecular Genetic Diagnostics, Institute of Human Genetics, Heidelberg University, Heidelberg, Germany

In the EDITA trial, patients with systemic sclerosis (SSc) and mild pulmonary arterial hypertension (PAH) treated with ambrisentan had a significant decline of pulmonary vascular resistance (PVR) but not of mean pulmonary arterial pressure (mPAP) versus placebo after 6 months. In the current study, we aimed to assess the long‐term effects of continued therapy with ambrisentan versus standard of care (SoC). Patients who participated in the EDITA study and received regular follow‐up clinical assessments in our centre were included in this study. Clinical, echocardiographic, laboratory, exercise and hemodynamic parameters after the termination of study participation were analysed. The primary endpoint was to assess whether continued treatment with ambrisentan versus SoC prevented the development of PAH according to the new definition. From 38 SSc‐patients participating in the EDITA study 4 were lost to follow‐up. Of the 34 remaining patients (age 55 ± years, 82.1% females), 19 received ambrisentan after the termination of the blinded phase and 15 continued with SoC. The mean follow‐up time was 2.25 ± 1.49 years, during which 29 patients underwent right heart catheterization. There was a significant improvement of mPAP in the group receiving ambrisentan versus SoC (−1.53 ± 2.53 vs 1.91 ± 2.98 mmHg, *p* = 0.003). In the SoC group five patients newly developed PAH with mPAP >20 mmHg in contrast to none of the patients receiving ambrisentan (*p* = 0.0084). Under continued targeted PAH therapy significantly more SSc patients were protected from deterioration of hemodynamics compared to patients receiving standard of care. Thus, early treatment and close follow‐up could be beneficial in this risk group for impeding PH/PAH development. Future trials in this field are needed to confirm these results.

## A043 FLUID RESTRICTION IN PATIENTS WITH PULMONARY ARTERIAL HYPERTENSION AND RIGHT HEART FAILURE

Carolin Resag^1,2,3^, Nicola Benjamin^1,2,3^, Panagiota Xanthouli^1,2,3,4^, Ishan Echampati^1,2^, Satenik Harutyunova^1,2,3^, Christina A. Eichstaedt^1,2,5^, Benjamin Egenlauf^1,2,3^, Ekkehard Grünig^1,2^

^1^Thoraxklinik at Heidelberg University Hospital, Heidelberg, Germany, ^2^Translational Lung Research Center Heidelberg (TLRC), Member of the German Center for Lung Research (DZL), Heidelberg, Germany, ^3^Department of Pneumology and Critical Care Medicine, Thoraxklinik Heidelberg gGmbH at Heidelberg University Hospital, Heidelberg, Germany, ^4^Department of Internal Medicine V: Hematology, Oncology and Rheumatology, University Hospital Heidelberg, Heidelberg, Germany, ^5^Laboratory for Molecular Genetic Diagnostics, Institute of Human Genetics, Heidelberg University, Heidelberg, Germany

In pulmonary arterial hypertension (PAH) right heart (RH) failure is associated with high mortality and poor prognosis. The objective of this study was to assess, whether reduction of fluid intake has an impact on RH size and clinical outcome in patients with PAH. In this study fluid‐uptake and clinical parameters were assessed at baseline and during 8.4 ± 5.3 months follow‐up in patients with invasively diagnosed PAH and signs of RH failure. All patients were advised to reduce their fluid uptake to <2 L/day. Hydropic decompensation was defined when patients were hospitalized and needed iv‐diuretics. Time to clinical worsening included death due to right heart failure, lung transplantation and hospitalization due to PAH. Clinical parameters were compared between groups by student's *t*‐test. Cox regression and log‐rank tests were performed to analyse survival, time to clinical worsening and their determining factors. Out of 66 patients with signs of fluid retention at baseline (normal fluid intake <2 L/day, *n* = 16; high fluid intake ≥2 L/d, *n* = 50), 21 presented with hydropic decompensation, which was significantly associated with worse survival (*p* = 0.004) and time to clinical worsening (*p* < 0.001). During follow‐up patients who reduced fluid intake <2 L/day improved their RV area (trend *p* = 0.051) and time to clinical worsening (*p* = 0.007). Hydropic decompensation and fluid intake during follow‐up were independent predictors of time to clinical worsening. The results of this study suggest that most patients with PAH who present with signs of right heart failure drink too much (more than >2 L/day). Fluid restriction was highly effective to improve right ventricular size and function and time to clinical worsening. Larger prospective studies should be performed for further evaluation.

## A044 DNMT3A DEPLETION IN HEMATOPOIETIC STEM CELLS PROMOTES PULMONARY ARTERIAL HYPERTENSION AND RIGHT VENTRICULAR DYSFUNCTION IN MICE

Isaac Emon^1^, Ruaa Al‐Qazazi^1^, Ashley Martin^1^, Patricia Lima^1^, Kuang‐Hueih Chen^1^, Danchen Wu^1^, Asish Das Gupta^1^, Mrs. Brooke Ring^1^, Curtis Noordhof^1^, Lindsay Jefferson^1^, Charles Hindmarch^1,2^, Michael Rauh^3^, Stephen Archer^1,2^

^1^Department of Medicine, Queen's University, Kingston, Canada, ^2^Queen's CardioPulmonary Unit, Queen's University, Kingston, Canada, ^3^Department of Pathology and Molecular Medicine, Queen's University, Kingston, Canada

Pulmonary Arterial Hypertension (PAH) is a fatal cardiopulmonary disorder due to the remodelling and stiffening of small pulmonary arteries. Increasing evidence suggests that inflammation plays a significant role in PAH pathogenesis. Mutations in TET2, a driver of clonal hematopoiesis of indeterminate potential (CHIP), predispose to developing an inflammatory form of PAH in patients. Preliminary human genetic sequencing data suggest that mutations in DNMT3A, the most mutated gene associated with CHIP, may also be linked to PAH development. To assess the biological plausibility of our human data, we study the effects of hematopoietic Dnmt3a depletion, which simulates loss‐of‐function DNMT3A CHIP mutations, on the development of PAH and inflammation in mice. We produced a conditional, Vav‐Cre driven hematopoietic Dnmt3a knockout mouse model [control (DNMT3Af/f), heterozygous (DNMT3A+/−), knockout (DNMT3A−/−)]. A group of 3‐month‐old male and female mice were exposed to a second hit (3 weeks of hypoxia) to accelerate PAH development, followed by 3 weeks of normoxia before conducting endpoint studies. Pulmonary artery acceleration time (PAAT) and tricuspid annular plane systolic excursion (TAPSE) were assessed using echocardiography. Right ventricular systolic and end‐diastolic pressures (RVSP, RVEDP) were evaluated using right‐heart catheterization (*n* = 6–9/group). Percent pulmonary artery medial wall thickness (%MT) was assessed using hematoxylin and eosin (H&E) staining. RV fibrosis was investigated using picrosirius red staining. Immune cell infiltration of the lung was measured via immunofluorescence, and specific leukocyte populations (macrophages, neutrophils, T‐cells and Bcells) were investigated using flow cytometry. Thirty‐two plasma cytokines were measured using the Luminex 200 system (Eve Technologies). Normoxic Dnmt3a−/− mice had significantly elevated RVSP (mean = 29.9 ± 2.8 mmHg, *p*= 0.0034), reduced PAAT (mean = 21.2 ± 0.7 ms, *p*= 0.0065) and decreased TAPSE (mean = 0.72 ± 0.02 mm, *p*= 0.0303), with a modest trend towards increased RVEDP (mean = 1.3 ± 0.3 mmHg, *p*=0.7885), compared to normoxic Dnmt3af/f mice (mean RVSP: 20.4 ± 0.7 mmHg, RVEDP: 0.7 ± 0.1 mmHg, PAAT: 25.0 ± 0.7 ms, TAPSE: 0.87 ± 0.02 mm). Further, hypoxic Dnmt3a−/− mice had evidence of more severe PAH with elevated RVSP (mean = 34.7 ± 1.6 mmHg, *p*=0.0003) and RVEDP (mean = 2.3 ± 0.7 mmHg, *p*= 0.0239), reduced PAAT (mean = 19.4 ± 0.7 ms, *p*=0.0247) and decreased TAPSE (mean = 0.69 ± 0.04 mm, *p*= 0.0232) in comparison to hypoxic Dnmt3af/f mice (mean RVSP: 23.1 ± 1.8 mmHg, RVEDP: 0.7 ± 0.2 mmHg, PAAT: 22.4 ± 0.4 ms, TAPSE: 0.81 ± 0.03 mm). Heterozygous mice developed an intermediate level of PAH in both normoxic (RVSP: 25.7 ± 1.5 mmHg, RVEDP: 0.7 ± 0.1 mmHg, PAAT: 21.6 ± 0.5 ms, TAPSE: *p*= 0.75 ± 0.04 mm) and hypoxic (RVSP: 34.2 ± 1.2 mmHg, RVEDP: 1.3 ± 0.4 mmHg, PAAT: 23.0 ± 1.8 ms, TAPSE: 0.70 ± 0.04 mm) conditions relative to their respective controls. There is an increase in %MT in hypoxic Dnmt3a−/− mice compared to hypoxic controls (57.83 ± 3.9% Dnmt3a−/−, 49.73 ± 2.8% Dnmt3af/f; *p*= 0.002, *n* = 3/group) as well as a trend towards an increase in RV fibrosis (*n* = 2/group). There was a significant increase in CD45+ leukocyte infiltration in the lungs of Dnmt3a−/− mice (*p*=0.0466), with a trend towards increased macrophages (*p *= 0.1897) and B cells (*p *= 0.2674). Interleukin 13 (IL‐13), which has been reported to be increased in patients with PAH associated with systemic sclerosis, was significantly increased in the plasma of normoxic Dnmt3a−/− mice compared to normoxic Dnmt3af/f controls (*p *= 0.001). Depleting Dnmt3a in the hemopoietic system is sufficient to provoke PAH in mice by increasing inflammation. This mouse model supports our human data that suggests DNMT3A mutations may be associated with PAH in patients.

## A045 ENGINEERED PULMONARY ARTERY TISSUES (EPATS)—A NOVEL TECHNIQUE TO ASSESS VASCULAR CONTRACTILITY

Adam Fellows^1^, Katie Quigley^1^, Harry Barnett^1^, David Miller^1^, Alex Ainscough^1^, Beata Wojciak‐Stothard^1^

^1^Imperial College London, London, UK

Conventional monolayer culture of vascular smooth muscle cells suppresses their contractile phenotype, a property crucial to cardiovascular function and disease. Therefore, we developed a novel in vitro three‐dimensional culture platform using hydrogels containing human pulmonary artery smooth muscle cells—‘Engineered Pulmonary Artery Tissues’ (EPATs). To allow rapid and direct measurement of vasoreactivity in vitro. EPATs were produced using custom‐made racks fabricated from polydimethylsiloxane (PDMS) using a 3D‐printed resin mould. Primary human pulmonary artery smooth muscle cells were suspended with fibrinogen and seeded between pairs of posts hanging from the PDMS racks. 3D constructs were formed after fibrin polymerisation by thrombin. Auxotonic stretch exerted by the PDMS posts is designed to re‐establish contractility within smooth muscle cells. Crucially, EPATs were able to mimic both vasoconstriction and vasodilation in response to various vasoactive reagents, indicated by flexion of the posts and subsequent measurements of changes in EPAT length using time‐lapse microscopy. Contractility was apparent 7 days after fabrication and changes were robust and reproducible over multiple experiments. Importantly, EPATs are viable for over 3 weeks enabling both long‐term treatments and repeated measurements over time. Finally, concentration‐response curves were generated for endothelin‐1 and imatinib, which are both highly relevant for the modelling and treatment of pulmonary arterial hypertension. The EPAT methodology provides the unique ability to reproduce SMC vasoactivity in vitro and has enormous potential to address the clinical need for better vasodilatory therapies for diseases such as pulmonary arterial hypertension.

## A046 PH‐PF IS LINKED TO A LOSS OF ENDOTHELIAL CELL HOMEOSTASIS

Elisabeth Fließer^1^, Katharina Jandl^2^, Anna Birnhuber^3^, Francesco Valzano^1^, MSc Thomas Lins^1^, Dagmar Kolb^4^, Vasile Foris^5^, Matthias Evermann^6^, Konrad Hoetzenecker^6^, Michael Kreuter^7^, Leigh M Marsh^1^, Malgorzata Wygrecka^8^, Grazyna Kwapiszewska^1^

^1^Ludwig Boltzmann Institute For Lung Vascular Research, Graz, Austria, ^2^Pharmacology, Medical University of Graz, Graz, Austria, ^3^Division of Physiology, Medical University of Graz, Graz, Austria, ^4^Core Facility Ultrastructure Analysis, Center for Medical Research, Medical University of Graz, Graz, Austria, ^5^Division of Pulmonology, Department of Internal Medicine, Medical University of Graz, Graz, Austria, ^6^Department of Thoracic Surgery, Medical University of Vienna, Vienna, Vienna, Austria, ^7^Mainz Center for Pulmonary Medicine, Departments of Pneumology, Mainz University Medical Center, Mainz, Germany, ^8^Center for Infection and Genomics of the Lung (CIGL), Universities of Giessen and Marburg Lung Center (UGMLC), Giessen, Germany

Pulmonary hypertension leads to worsen the outcome of patients with pulmonary fibrosis. In line, pronounced vascular remodelling and inflammation, abnormal alveolar‐capillary permeability and aberrant coagulation and vessel density are common features observed in the fibrotic lung. However, a comprehensive analysis of endothelial integrity/homeostasis (e.g., CD31, VE‐Cadherin, Thrombomodulin), as well as activity markers (e.g., von‐Willebrand‐Factor/vWF, P‐selectin, vascular cell adhesion molecule 1/VCAM‐1), is lacking so far. Endothelial integrity and activation have been measured in human lung and plasma samples by immunohistochemistry, transmission electron microscopy, qPCR, ELISA as well as based on functional in‐vitro assays. The immunohistochemical analysis revealed a pronounced alteration in endothelial cell morphology in fibrotic lungs. Marked structural changes were further supported by ultrastructural analysis based on transmission electron microscopy. A significantly elevated abundance of the activation marker von‐Willebrand‐Factor (vWF) in PH‐PF lung tissue indicated, that the pulmonary endothelium was hyper‐activated in the diseased state. Concordantly, the gene expression of activity marker VCAM‐1 was significantly upregulated. On the other hand, barrier markers ICAM‐2, CD31, and VE‐Cadherin were significantly decreased in the lungs of PF patients. The potential hyper‐activation was going hand in hand with increased counts of infiltrating immune cells in fibrotic lungs and a stronger adhesion of immune cells to ECs isolated from fibrosis lungs in vitro. Moreover, ECs isolated from fibrotic lungs had a reduced capability to establish a proper barrier in vitro as compared to donor ECs. The changes of endothelial integrity and activation were represented in PF patients' plasma. While integrity markers ICAM‐2, CD31, VE‐Cadherin, and Thrombomodulin were decreased, activation markers vWF, P‐selectin, and IL‐8 were increased. Our thorough analysis of endothelial integrity and activation markers in human lung tissue and plasma samples revealed a pronounced dysregulation of the endothelium in PF. Our data support the existence of an important link between functional endothelial changes, inflammation, and fibrogenesis in PH‐PF.

## A047 INTRAPULMONARY T CELLS ARE SUFFICIENT FOR SCHISTOSOMA‐INDUCED PULMONARY HYPERTENSION

Dara Fonseca Balladares^1^, Rahul Kumar, Claudia Mickael, Biruk Kassa, Ari Molofsky Elina Wells, Michael Lee, Kevin Nolan, Rubin Tuder, Brian Graham

^1^University Of California, San Francisco Ucsf Pulmonary Division, San Francisco, USA

Schistosomiasis is a parasitic infection that can cause pulmonary hypertension (PH), a disease of thickened lung blood vessels. We previously found Th2 CD4 T cells are required for Schistosoma‐PH in mice. However, it is unknown if CD4 T cells need to be located in the lung to initiate the localized inflammation that leads to vascular remodeling. Here, we tested the hypothesis that Schistosoma‐PH requires activated Th2 CD4 T cells to migrate to the lung parenchyma by administering FTY720, which blocks lymphocyte egress from lymph nodes. Female C57Bl6 mice (Jackson Labs) were sensitized with 240 *S. mansoni* eggs/gram intraperitoneally (IP) and were challenged intravenously (IV) 2 weeks later with 175 eggs/gram by tail vein injection. Daily FTY720 (0.5 mg/kg IP) or PBS (equivalent volume IP) was started the day before IV egg administration. Three days after IV eggs, *N* = 6–7 mice per group were killed, and the lungs were digested for flow cytometry. Seven days after IV eggs, *N* = 6–7/group underwent right heart catheterization to measure right ventricle systolic pressure (RVSP). FTY720 treatment decreased the number of circulating CD3 and CD4 T cells in FTY720‐treated mice compared to vehicle‐treated mice. In the mediastinal lymph nodes, there were more CD3 and CD4 T cells, whereas, in the lungs, there were fewer CD3 and CD4 T cells in the FTY720‐treated mice. The percentage of CD44+CD69+ (i.e., educated and activated) CD4 T cells was greater in the lungs of the FTY720‐treated mice, but the absolute number was decreased. FTY720 treatment caused no change in RVSP and or any change in right ventricle hypertrophy compared to vehicle‐treated mice. Conclusions: Blocking T cell migration into the lungs was insufficient to suppress PH following IV Schistosoma egg challenge. Pre‐positioned CD4 T cells in the lung may be sufficient to cause Type 2 inflammation and PH.

## A048 COVID‐19 AND PULMONARY ARTERIAL HYPERTENSION: A SWORD WITH TWO EDGES AFFECTING PULMONARY ENDOTHELIUM

Frantzeska Frantzeskaki^1^, Dimitrios Konstantonis^1^, Michail Rizos^1^, Vasileios Kitsinelis^1^, Iraklis Tsangaris^1^

^1^Attikon University Hospital, 2nd Department of Critical Care, Pulmonary Hypertension Clinic, Athens, Greece

COVID‐19 pneumonia caused by SARS‐CoV‐2 coronovirus is characterized by pulmonary endothelial dysfunction. Pulmonary hypertension (PH) is associated with pulmonary vascular remodelling and endothelial dysfunction. There are limited reports on the incidence and outcomes of COVID‐19 pneumonia in PH patients. To describe the incidence and outcomes of patients with PAH and COVID‐19, in a Greek PH expert centre of a tertiary university hospital. We retrospectively recorded the demographic characteristics, laboratory tests, management and outcome of PH patients of Group 1 and Group 4, who developed COVID‐19, between February 2020 and September 2023. Functional and hemodynamic data of PH patients before COVID‐19 disease were retrieved from the respective medical files. During the study period, 25 prevalent PH patients (mean age 51 years old) developed COVID‐19, confirmed by PCR (20 patients suffering from pulmonary arterial hypertension and 5 from chronic thromboembolic pulmonary hypertension). The hospitalization rate was 52%. All patients were under double or triple PAH‐targeted therapy, which was resumed during COVID‐19. Hospitalized patients received remdesivir, corticosteroids, antibiotics and oxygen therapy, while two patients required high flow oxygen therapy. None of the patients was intubated. Eight patients (32%) died from COVID‐19. Nonservivors were older than survivors, while they suffered from comorbidities and severe PH with right ventricular impairment. The mean duration of hospitalization was 15 days. After hospital discharge, patients were at the same functional class, as before COVID‐19. COVID‐19 in PH patients is associated with high hospitalization rate. Older age and comorbidities are associated with a higher risk of death.

## A049 SPATIALLY RESOLVED TRANSCRIPTOMICS IN ALVEOLAR CAPILLARY DYSPLASIA IDENTIFY SYSTEMIC, PULMONARY VENOUS AND LYMPHATIC VESSEL SPECIFIC GENE NETWORK OF DYSPLASTIC CAPILLARIES AND REDUCED NUMBER OF MATURE ALVEOLAR CAPILLARIES

Csaba Galambos^1^, Aasta Khatiwada^2^, Gregory Seedorf^1^, Rachel Blumhagen^2^, Steven Abman^1^

^1^University of Colorado, Aurora, USA, ^2^National Jewish Health, Denver, USA

Alveolar capillary dysplasia (ACD), a severe developmental lung disease is associated with intractable pulmonary hypertension (PH). FOXF1 gene abnormalities have been strongly linked to ACD, however signaling mechanisms that lead to PH are unknown. We have identified the presence of an expanded systemic (bronchial) vascular network characterized by the presence of open intrapulmonary bronchopulmonary anastomoses (IBAs) in ACD. IBAs are precapillary vascular connections between pulmonary and bronchial circulations that allow blood flow to bypass the pulmonary bed creating a right‐to‐left‐shunt leading to severe hypoxia. In addition to IBAs, many malpositioned dilated and congested microvessel (best known as dysplastic capillaries) are present in the distal ACD lung. However, it is unknown if the dysplastic capillaries are related to systemic circulation and how they contribute to ACD and PH. We aimed to study the endothelial cell (EC) genetic signature of dysplastic capillaries using GeoMx digital spatial profiler (DSP). GeoMx‐DSP is a novel platform that enables spatially resolved, high‐plex (10 s–10,000 s) quantitation of mRNA in formalin‐fixed paraffin‐embedded (FFPE) tissue. As opposed to single‐cell methods, GeoMx‐DSP allows concurrent identification of diseased vessels and vessel cell‐specific transcriptomic signatures. We obtained lung biopsies from four ACD patients (two males and two females) and four controls (three females and one male) and performed morphologic identification of vessel and vessel cell types by immunofluorescence staining (CD31‐EC, SMA594‐smooth muscle cells, and Syto82‐cell nuclei), followed by regions of interest selection. Following QC, normalization, and background correction, we used a linear mixed effects model to test for differential gene expression where genes with an adjusted *p*‐value < 0.05 were considered significant. A total of 53 samples, 27 unique ROI (28 CD31/EC, 25 SMA/smooth muscle cells) were obtained across eight subjects following QC. We compared EC gene transcriptomics in ACD dysplastic capillaries to those of alveolar capillaries in controls in the CD31 regions. We identified increased expression of CC14, PVLPV, ACKR1, SELE and PDE2A among the top 12 genes, suggesting that dysplastic capillaries are of systemic, pulmonary venous and lymphatic origin. TMEM100, expressed mainly by mature alveolar capillaries was significantly decreased in ACD patients compared to controls, suggesting a reduced mature alveolar capillary network in ACD. The finding of downregulated SEMAC among the top 12 genes corroborates recent data showing perturbed Semaforin genes in ACD patients and further suggests that ECs of dysplastic capillaries are immature. Using Col15A1 immunohistochemical staining (IHC) along with electron microscopic studies (EM), we identified increased numbers of systemic EC in ACD. Specific lymphatic IHC marker D2‐40 confirmed increased distal lymphatic vessels, while decreased TMEM100 IHC confirmed reduced number of mature alveolar capillaries in ACD lungs. Based on the combined data from GeoMx‐DSP, histology, IHC and EM we propose that the dysplastic capillary network in ACD is an extension of diverse types of vessels including systemic, pulmonary venous and lymphatics in the distal ACD lung, and that there is an attenuated mature alveolar capillary network. Further studies are needed to investigate whether targeting these genes induces capillary maturation and PH reduction in ACD patients.

## A050 TARGETING ENAMPT IN PAH‐ASSOCIATED RIGHT VENTRICULAR DYSFUNCTION: PRECLINICAL RAT PAH STUDIES AND NAMPT GWAS ANALYSES

Joe GN Garcia^1,2^, Mohamed Ahmed^3^, Akash Gupta^3^, Nancy G. Casanova^1^, Franz Rischard^3^, Heidi Erickson^3^, Michael W. Pauciul^4^, Tae‐Hwi L. Schwantes‐An^5^, William C. Nichols^4^, Jason Yuan^6^, Ankit Desai^3^

^1^University Of Florida, Jupiter, USA, ^2^Aqualung Therapeutics, Corp, Juno Beach, USA, ^3^University of Arizona, Tucson, USA, ^4^Cincinnati Children's Hospital Medical Center, Cincinnati, USA, ^5^Indiana University School of Medicine, Indianapolis, USA, ^6^University of California, San Diego, USA

Dysregulated innate immunity‐driven inflammatory pathways play critical causal roles in the pathogenesis of pulmonary vascular remodeling, right ventricular dysfunction, and pulmonary arterial hypertension (PAH). Damage‐associated molecular pattern molecules (DAMPs) are actively secreted or passively released by damaged cells to amplify inflammatory cascades via pattern recognition receptors (PRRs) including Toll‐Like Receptors 2 (TLRs) and the Receptor for Advanced Glycation End Products (RAGE). For example, high‐mobility box group 1 (HMGB1) is a dually functioning cytozyme implicated in PAH that intracellularlly functions as a non‐histone nucleoprotein. However, when secreted or released from dying cells, HMGB1 exhibits strong DAMP properties via binding to TLR2, TLR4, and RAGE. Similarly, our genomic‐intensive approaches identified a novel cytozyme, eNAMPT (extracellular nicotinamide phosphoribosyl‐transferase), as a highly evolutionarily‐conserved DAMP and master regulator of inflammatory cascades via ligation of TLR4. Intracellular NAMPT is a major NAD‐synthesizing enzyme with strong antiapoptotic properties via SIRT1. However, in response to multiple cellular stressors (such as virus, bacteria, mechanical stress, and hypoxia), eNAMPT is released into the circulation with plasma eNAMPT levels linked to disease severity/mortality in severe acute inflammatory lung disorders (including ARDS and sepsis). Our published work and preliminary studies support the speculation that activation of the eNAMPT/TLR4 inflammatory cascade is a key contributor to endothelial‐smooth muscle activation, vascular remodeling, and right ventricular dysfunction in human PH pathobiology. First, NAMPT RNA and protein expression are significantly increased in PBMCs and in remodeled lung vessels from PAH patients. Secondly, plasma eNAMPT levels are elevated in PAH subjects with levels correlating with an index of cardiac catheterization‐proven RV dysfunction. Third, a PAH BioBank GWAS study (674 iPAH subjects, 250 controls) identified three NAMPT SNPs (rs61330082, rs9770242, and rs59744560), previously linked to ARDS mortality, to be highly significantly associated with cardiac catheterization‐proven indices of RV dysfunction and PAH severity, including cardiac output, peripheral vascular resistance (PVR), and right ventricular stroke work index (RVSWi). Fourth, the targeting of the eNAMPT/TLR4 pathway in preclinical PAH monocrotaline and hypoxia/Sugen rat studies demonstrate that eNAMPT is a highly druggable target as the subcutaneous delivery of humanized eNAMPT‐neutralizing mAb, ALT‐100, profoundly attenuated PAH vascular remodeling and indices of RV dysfunction in rats with established PH (>35%–40% reductions in PAH severity in each model). Lastly, genomic studies validated ALT‐100 targeting of the eNAMPT/TLR4 pathway in rat lung tissues and human PAH PBMCs as the mechanism by which ALT‐100 mAb rescues PAH severity and halts PAH progression. These results strongly validate eNAMPT as a novel PAH therapeutic candidate. Given that the eNAMPT‐neutralizing ALT‐100 mAb is currently in Phase 2A clinical trials for the indication of moderate to severe ARDS and exhibits a strong safety profile, ALT‐100 mAb may represent a potential PAH therapy to address the unmet need for strategies to improve RV dysfunction/failure and PAH survival. In addition, future PAH clinical trials designed to assess eNAMPT as a novel PAH therapeutic target may benefit from subject stratification strategies utilizing plasma eNAMPT levels and/or NAMPT SNPs to enhance the likelihood of therapeutic success.

## A051 ALDOSTERONE INHIBITION ALLEVIATES TYPE 2 PULMONARY HYPERTENSION BY PHENOTYPIC SHAPING OF MONOCYTE SUBSETS

Jonathan Gillan^1,2^, Niklas Hegemann^1,2^, Willem Bintig^1^, Wolfgang Kuebler^1,2^, Jana Grune^1,2^

^1^Institute of Physiology, Charité‐Universitätsmedizin Berlin, Berlin, Germany, ^2^DZHK (German Centre for Cardiovascular Research), Berlin, Germany

Aldosterone (ALDO) is the final mediator of the renin‐angiotensin‐aldosterone system, RAAS, and a driver of hypertension and inflammation. RAAS overactivation leads to left heart disease (LHD) which is commonly associated with secondary pulmonary hypertension (Type 2 PH). Dysregulated RAAS components and elevated ALDO levels have been described in patients with Type I pulmonary hypertension (PH), even in the absence of LHD. Hence, ALDO excess may be an unrecognized contributor to PH, potentially via immune cell dysregulation in hearts and lungs. We induced myocardial infarction (MI) in 8‐week‐old male C57BL/6 mice. After 6 weeks, MI‐mice presented with LHD and Type 2 PH, evidenced by reduced ejection fraction, lung edema, and right ventricular (RV) hypertrophy compared to naïve controls. Flow‐cytometric analyses revealed a phenotypic switch of reparative Ly6Clo monocytes towards inflammatory Ly6Chi monocytes in the blood and lungs of MI mice. To test for the role of ALDO in PH‐LHD, we performed bilateral adrenalectomy (ADX) 1 week before MI induction, resulting in an absence of systemic ALDO. ADX rescued the MI‐induced shift towards Ly6Chi monocytes and resulted in greater numbers of reparative Ly6Clo monocytes compared to MI mice with regular ALDO plasma levels, demonstrating ALDO‐directed programming of monocyte subsets. In line, treatment with the ALDO antagonist spironolactone (SPIRO) resulted in higher Ly6Clo monocyte counts in blood and lungs of MI‐mice compared to vehicle‐treated controls. Moreover, SPIRO treatment alleviated histologically assessed lung vascular maladaptation, lung edema, and RV hypertrophy compared to controls, while the underlying LHD was comparable between both groups. To assess the relevance of this phenotype in a clinical setting, we are currently investigating circulating immune profiles of a cohort of patients with primary aldosteronism. A particular focus of this project will be on circulating monocyte phenotype and function in such patients, and how these change following aldosterone‐reducing treatment, either with ADX or ALDO antagonists. ALDO has been demonstrated to upregulate cellular adhesion molecules, which facilitate extravasation and recruitment of monocytes to the site of injury leading to vascular inflammation and remodeling. Hence, we measured soluble vascular cell adhesion molecule 1 (VCAM‐1) and intercellular adhesion molecule 1 (ICAM‐1) in serum samples from Type I PH patients and healthy donors. We found both VCAM‐1 and ICAM‐1 to be increased in serum samples from PH patients compared to healthy donors. Viewed together, our data suggest that ALDO‐directed phenotypic programming of immune cells, particularly monocytes, is a so far unrecognized driver of lung vascular inflammation that may mediate ALDO‐dependent lung vascular remodeling and RV dysfunction. Our data provide evidence that ALDO blockage presents a potential strategy for therapeutic intervention in PH.

## A051 CATHEPSIN D—A PROTEASE MOONLIGHTING AS A PATHOLOGICAL MECHANISM IN COPD‐ASSOCIATED PULMONARY HYPERTENSION

Ann‐Kathrin Fluethmann^1^, Vinita Sharma^1^, Oleg Pak^1^, Ralph Schermuly^1^, Ardeschir Ghofrani^1,2^, Werner Seeger^1,3,4^, Natascha Sommer^1^, Norbert Weissmann^1^, Marija Gredic^1^

^1^Cardio‐Pulmonary Institute (CPI), Universities of Giessen and Marburg Lung Center (UGMLC), Member of the German Center for Lung Research (DZL), Justus‐Liebig‐University (JLU), Giessen, Germany, ^2^Department of Medicine, Imperial College London, London, UK, ^3^Institute for lung health (ILH), Giessen, Germany, ^4^Max Planck Institute for Heart and Lung Research, Member of the DZL, Bad Nauheim, Germany

Chronic obstructive pulmonary disease (COPD) is an incurable condition with increasing prevalence and the third most common cause of death worldwide. In addition to the respiratory tract pathology, which includes chronic bronchitis and emphysema, the majority of COPD patients have pulmonary vascular abnormalities and pulmonary hypertension (PH). Even mild to moderate elevation of pulmonary arterial pressure (PAP) is associated with an increased risk of exacerbations and higher mortality in COPD patients. Moreover, current evidence suggests that pulmonary vascular alterations precede emphysema, possibly contributing to its development and/or progression. We recently demonstrated that a cross‐talk between interstitial “M2‐like” macrophages and adjacent pulmonary artery smooth muscle cells (PASMCs) drives pulmonary vascular remodeling in COPD and that the mechanism is largely hypoxia‐independent. Importantly, our results implicated inducible nitric oxide synthase (iNOS) activity in macrophages and activation of extracellular signal‐regulated kinase (ERK)‐1/2 in PASMCs as crucial events in these dysregulated cellular mechanisms. Intriguingly, our data suggested that iNOS exerts its role through the nitration of certain signaling proteins that have yet to be identified. Cathepsin D (Cts‐D) is a ubiquitously expressed lysosomal aspartic protease, upregulated in macrophages acquiring an “M2‐like phenotype,” whose activity can be regulated via protein nitration. In pathological conditions such as cancer, this enzyme is secreted in its inactive form into the intercellular environment, where it triggers the proliferation and migration of cancer cells and stromal fibroblasts. Therefore, we hypothesized that Cts‐D may play a role in the cross‐talk of “M2‐like” macrophages and PASMCs, leading to PASMC proliferation and pulmonary vascular remodeling in COPD. To test this hypothesis, we determined Cts‐D expression in pulmonary vasculature of smoke‐exposed mice and COPD patients, and investigated the effects of Cts‐D modulation on proliferation of PASMCs in vitro. Interestingly, our data demonstrated that Cts‐D is strongly upregulated in the pulmonary vascular compartment of COPD lungs, and lungs of cigarette smoke‐exposed, but not chronic hypoxia‐exposed mice. Furthermore, in accordance with the literature, we found that Cts‐D expression is upregulated in bone marrow‐derived macrophages acquiring M2 phenotype in vitro. Of note, our data suggested that M2‐polarized macrophages may not be (the only) source of Cts‐D in co‐cultures with PASMCs. Namely, the increase of Cts‐D level in the co‐culture medium was accompanied by a decrease of the intracellular level in PASMCs, suggesting the secretion of Cts‐D from these cells in response to signals from macrophages. Finally, we tested whether modulation of the Cts‐D concentration affects the proliferation of PASMCs. We observed that the addition of recombinant pro‐Cts‐D augments the proliferation of cultured PASMCs in a concentration‐dependent manner, whereas the knockdown of this protein exerts the opposite effect. Importantly, addition of recombinant Cts‐D to the PASMC culture induced phosphorylation of ERK‐1/2, a kinase previously shown to be involved in the pro‐proliferative cross‐talk of PASMCs with adjacent macrophages and resulting pulmonary vascular remodeling. Overall, our data demonstrate that Cts‐D expression is increased in the pulmonary vasculature in COPD lungs, and suggest an important role of this protein for PASMC proliferation underlying pulmonary vascular remodeling.

## A052 DOES OXIDATION OF PROTEIN KINASE A REGULATORY SUBUNIT PKARIΑ PLAY A ROLE IN PULMONARY HYPERTENSION?

Hannah Green^1^, Anthony Rasetta^1^, Manpreet Kaur^1^, Philip Eaton^2^, Olena Rudyk^1^

^1^School of Cardiovascular and Metabolic Medicine & Sciences, King's College London, London, UK, ^2^William Harvey Research Institute, Barts & The London School of Medicine & Dentistry, Queen Mary University of London, London, UK

There is an unmet need for new treatments to reverse vascular remodeling and alleviate pulmonary arterial pressure in patients with pulmonary hypertension (PH). In the vasculature, protein kinase A (PKA) regulates smooth muscle relaxation, proliferation and angiogenesis, the perturbation of which have all been implicated in the vascular remodeling associated with PH. PKA is classically activated by cyclic AMP; however, PKA isoforms containing the PKARIα regulatory subunit can also be activated by oxidants such as H₂O₂. Oxidation of redox‐active cysteine residues within the PKARIα subunit results in the formation of an interprotein disulfide homodimer which transduces oxidant signals into phosphorylation‐mediated events. Using wire myography and blood pressure telemetry in Cys17Ser PKARIα ‘redox‐dead’ knock‐in (KI) mice, in which the PKARIα disulfide dimer cannot be formed, a role for disulfide‐PKARIα in systemic blood pressure regulation has been identified. Isolated aortic and mesenteric vessels from KI mice displayed impaired H₂O₂‐induced vasodilation and increased vasoconstriction in response to phenylephrine or angiotensin II compared to those from wildtype (WT) littermates. After chronic angiotensin II treatment, exacerbated hypertension and cardiac hypertrophy were apparent in KI mice compared to WT mice, indicating a beneficial, pressor‐limiting role for disulfide‐PKARIα in the systemic vasculature. It was a logical possibility that a similar mechanism operates in pulmonary arteries and therefore it was intuitive to reason that oxidant‐activated PKARIα may play a functional role in PH, which is associated with alterations in cellular metabolism and redox balance. Notably, in pulmonary arteries and smooth muscle cells isolated from idiopathic pulmonary arterial hypertension patients, a decrease in the abundance of disulfide‐PKARIα was observed compared to donors. Similarly, a decrease in disulfide‐PKARIα was apparent in lung tissue from mice subjected to a Sugen‐hypoxia model of pulmonary arterial hypertension. Given its contribution to vasodilation, we hypothesized that disulfide‐PKARIα may play a beneficial role in PH. To begin investigating this, male WT and KI mice were subjected to a chronic hypoxia‐induced (10% O₂) PH model for 3 weeks. Interestingly, the hypoxia‐induced elevation of right ventricle systolic pressure (RVSP), right ventricle (RV) hypertrophy and pulmonary vascular resistance (PVR) occurred to a lesser extent in KI mice than WT littermates, suggesting an unexpected detrimental role of disulfide‐PKARIα in PH, which perhaps counterbalanced its possible protective signaling role in vasodilation. Consistent with the mild‐to‐moderate course of a chronic hypoxia PH model, there was no evidence of cardiac dysfunction or failure in either WT or KI mice. Preliminary analysis of α‐smooth muscle actin staining of lung tissue indicated a similar extent of pulmonary vessel muscularization in WT and KI mice following exposure to hypoxia. Furthermore, preliminary data from a 6‐week chronic hypoxia study suggest possible sex differences in the role of disulfide‐PKARIα in PH, as male KI mice showed less severe increases in RVSP, RV hypertrophy and PVR compared to WT mice in hypoxia, whereas the opposite trend was apparent in female mice. Ongoing work investigates whether a contribution of disulfide‐PKARIα to endothelial or smooth muscle cell proliferation and/or angiogenesis may contribute to PH disease progression.

## A053 UNDERSTANDING PULMONARY ARTERIAL HYPERTENSION THROUGH PATIENT IPSCS AND ORGANOIDS: ADVANCING MECHANISTIC INSIGHTS AND THERAPEUTIC INNOVATIONS

Mingxia Gu^1^, Silin Sa^2^, Michele Donato^2^, Minzhe Guo^1^, Neil Wary^1^, Yifei Miao^1^, Rebecca Harper^2^, Lingli Wang^2^, Purvesh Khatri^2^, Joseph Wu^2^, Marlene Rabinovitch^2^

^1^Cincinnati Children's Hospital Medical Center, Cincinnati, USA, ^2^Stanford University, Palo Alto, USA

Pulmonary arterial hypertension (PAH) is a progressive disorder characterized by occlusive vascular remodeling. The central role of endothelial cell (EC) dysfunction in PAH pathophysiology necessitates a tailored approach to therapy due to the heterogeneous causal mechanisms involved. To address this challenge, we harnessed patient‐specific induced pluripotent stem cells (iPSCs) from individuals with PAH, directing their differentiation into ECs and vessel organoids, thereby establishing a human‐specific disease modeling platform. Our investigation unveiled significant impairments within PAH iPSC‐derived ECs, notably compromised cell adhesion, migration, and viability under serum‐deprived conditions. Concomitantly, a reduction in BMPR2 expression, along with downstream signaling and collagen IV levels, underscored the dysregulated molecular landscape. Recognizing the intricate cell–cell interactions prevailing in the pulmonary vasculature, we advanced our study by generating 3D vessel organoids comprising both ECs and pericytes. Beyond the previously observed EC dysfunctions, these organoids exhibited augmented endothelial‐to‐mesenchymal transition, aberrant angiogenic responses, and disrupted EC‐pericytes crosstalk. Leveraging this model, we further performed a high‐throughput phenotypic drug screening in six PAH iPSC‐EC lines. These cells were exposed to a library of 4500 compounds, followed by evaluation of improved cell survival under serum withdrawal. Through phenotypic evaluation and computational analyses of publicly available transcriptomic datasets, we identified a promising lead compound, AG1296. AG1296 countered PAH phenotypes by enhancing EC survival, suppressing proliferation, and improving BMPR2 signaling. AG1296 also induced regression of neointimal lesions and PA occlusive changes in the Sugen/hypoxia rat model, reducing right ventricular pressure. The efficacy of the compound was validated by improved vascular function and BMPR2 signaling, aligned with the upregulation of the anti‐PAH gene signature. Through transcriptomic analysis, AG1296 upregulated SMAD 1/5 coactivators, CREB3 and CREB5, therefore promoting downstream genes that regulate EC functions. In conclusion, our study demonstrates the feasibility of investigating PAH cellular phenotypes and mechanisms in a dish, while also highlighting the accelerated potential of PAH drug discovery through the integration of phenotypic screening and computational analyses of publicly accessible datasets.

## A054 CHROMATIN REMODELING FACTOR ARID1A AS A NOVEL THERAPEUTIC TARGET IN THE PATHOGENESIS OF PULMONARY ARTERIAL HYPERTENSION

Kate Jankowski^1^, Irene Turnbull^1^, Pr Sebastien Bonnet, Malik Bisserier, Lahouaria Hadri^1^

^1^Icahn School Of Medicine At Mount Sinai, New York, USA

Pulmonary arterial hypertension (PAH), a rare and severe cardiopulmonary vascular disease, is characterized by the remodeling of pulmonary arterioles that leads to increased vascular resistance, resulting in right ventricular failure and death. We and others have shown that the dysregulation of epigenetic mechanisms plays a critical role in PAH pathogenesis. Thus, we contend that targeting chromatin‐remodeling factor and epigenetic regulators to reduce PASMC overgrowth, will improve pulmonary vascular function and structure. This will have a major clinical impact as a viable approach to treating PAH. The SWItch/Sucrose Non‐Fermentable (SWI/SNF) chromatin remodeling complexes control the accessibility of chromatin to transcriptional and coregulatory machinery. The AT‐rich interactive domain‐containing protein 1a (ARID1a), a subunit of the SWI/SNF chromatin‐remodeling complex, plays key roles in normal physiology and diseases. We hypothesized that the chromatin‐remodeling factor ARID1a plays a key role in the landscape of chromatin accessibility and in epigenetic modifications of target genes whose expression is responsible for PASMC overgrowth and dysfunction. By qRT‐PCR and western blot analysis we showed that ARID1a is significantly decreased in PAH human lung samples, while the enhancer of zest homolog 2 (EZH2) expression is increased. In hPASMC, our results showed that ARID1a gain‐and loss‐of‐function modulate hPASMC proliferation and downregulate EZH2 expression. Furthermore, we found that depletion of ARID1a in vivo using lenti.shARID1a significantly increased pulmonary vascular and increased right ventricle systolic pressure and RV remodeling. Altogether, this study unveiled the role of ARID1a in pulmonary hypertension and identified a novel molecular mechanism involved in the regulation of EZH2 by ARID1a in PAH.

## A055 EXPLORING FGF10‐MEDIATED REVERSAL OF PULMONARY HYPERTENSION IN COPD: INSIGHTS INTO CELLULAR MECHANISMS AND THERAPEUTIC POTENTIAL

Stefan Hadzic^1^, Edma Loku^1^, Marija Gredic^1^, Oleg Pak^1^, Mareike Gierhardt^2^, Simone Kraut^1^, Jochen Wilhelm^1,3^, Baktybek Kojonazarov^1,3^, Natascha Sommer^1^, Hossein A. Ghofrani^1^, Ralph T. Schermuly^1^, Werner Seeger^1,4^, Elie El Agha^1,3^, Saverio Bellusci^1^, Norbert Weissmann^1^

^1^Excellence Cluster Cardio‐Pulmonary Institute (CPI), Universities of Giessen and Marburg Lung Center (UGMLC), Member of the German Center for Lung Research (DZL), Justus‐Liebig‐University, Giessen, Germany, ^2^Max‐Planck Laboratory for Heart & Lung Research, Biomedicine Research Institute of Buenos Aires (IBioBA) ‐ CONICET ‐ Partner Institute of the Max Planck Society, Buenos Aires, Argentina, ^3^Institute for Lung Health (ILH), Justus‐Liebig‐University, Giessen, Germany, ^4^Max‐Planck Institute for Heart and Lung Research, Bad Nauheim, Germany

Pulmonary hypertension (PH) is often associated with chronic obstructive pulmonary disease (COPD) due to remodelling of the pulmonary vasculature. Moreover, emerging evidence suggests that early molecular and cellular alterations in the pulmonary vasculature precede and potentially contribute to emphysema development in COPD lungs. We previously showed that mice with impaired fibroblast growth factor (FGF) 10 signalling spontaneously develop PH and emphysema. In our transgenic animal model, PH was characterized by pulmonary vascular pruning and remodelling. FGF10 overexpression effectively reversed established CS‐ and elastase‐induced experimental PH and emphysema in mice. Notably, the regression of PH preceded the reversal of emphysema. However, the precise molecular and cellular mechanisms governing FGF10‐mediated repair of adult pulmonary vascular and alveolar structures remained elusive. Histological analysis of lung sections revealed that FGF10 treatment reversed CS‐induced vascular remodelling, which was evident through reduced pulmonary vessel muscularization. Transcriptomic analysis in the dissected pulmonary vascular compartment revealed the prominent downregulation of transcription factor TOX High Mobility Group Box Family Member 2 (Tox2) upon CS exposure, followed by upregulation upon FGF10 overexpression. Additionally, analysis of available single‐cell RNA sequencing data pinpointed Tox2 expression in pulmonary arterial endothelial cells, where the vascular remodelling leading to PH development occurs. Furthermore, increased TOX2 expression was verified in lung sections of COPD patients with diagnosed PH compared to lungs with healthy vasculature. In the dissected distal lung parenchyma, the transcriptomic analysis revealed that FGF10 treatment reversed the CS‐induced decrease in the expression of endothelial cell markers. The histological assessment confirmed the expansion of endothelial cells in the distal lung parenchyma following FGF10 overexpression, implying capillary network repair and vascular pruning reversal in utilized COPD animal models. Remarkably, the impact on the capillary network preceded the FGF10‐induced reversion of emphysema, indicating that the expansion of endothelial cells in the distal lung parenchyma may influence alveolar septal repair. These findings were further corroborated by experiments on cultured human COPD precision‐cut lung slices treated with recombinant FGF10. Ongoing investigations employing cell‐specific knockout mice aim to elucidate the precise cellular mechanisms underlying FGF10‐mediated lung repair upon injury. Our study sheds light on the promising role of FGF10 in reversing pulmonary hypertension and emphysema, offering insights into novel therapeutic approaches for these debilitating conditions.

## A056 MICROFLUIDIC PLATFORM FOR MODELLING OF ALVEOLAR‐VASCULAR CELL INTERACTIONS IN PULMONARY HYPERTENSION (PH) ASSOCIATED WITH CHRONIC OBSTRUCTIVE PULMONARY DISEASE (COPD)

Maike Haensel^1^, Vanessa Ho^1^, Joshua Edel^1^, Darryl Overby^1^, Clare Lloyd^1^, Beata Wojciak‐Stothard^1^

^1^Imperial College London, London, UK

Pulmonary hypertension (PH) is a severe disease characterized by progressive thickening of the small intrapulmonary arterioles, which restricts blood oxygenation and leads to heart failure. PH is a common complication of lung diseases such as COPD. To better understand PH development, it is important to simultaneously monitor changes happening in different cell types in the peripheral lung, in contact with pulmonary vasculature. At present, there is no cure for the disease, as conventional cell culture methods and animal models do not accurately mimic the human physiology, which leads to high rates of drug failure. Organs‐on‐chips enable key structural and functional features of human organs to be mimicked for in‐vitro disease modelling. To develop a novel organ‐on‐a‐chip device for co‐culture of human pulmonary artery endothelial cells with small airway epithelial cells, smooth muscle cells, pericytes, and fibroblasts under basal and disease conditions induced by hypoxia, inflammation, and pollution. (REspiratory‐and‐VAScular)‐on‐a‐chip (REVAS) was designed and fabricated using photolithography. Top and bottom polymer microfluidic channels were overlayed hosting alveolar and vascular cells, separated by a porous membrane, and perfused using a custom‐built flow system. In silico simulation of flow and pressure within the REVAS circuit was performed with COMSOL Multiphysics. To establish a COPD model, human small airway epithelial cells were incubated with 5, 10, and 20 µM of hydrogen peroxide under flow to induce oxidative stress condition. Cell morphology, differentiation markers, permeability, and cell function were studied for all primary cell types that were co‐cultured. Simulation of air flow (1 dyne/cm^2^) through respiratory channels and media flow (4 dynes/cm^2^) through vascular channels confirmed physiological range of pressure, flow pattern, and wall shear stress. Extracellular matrix and co‐culture medium components were optimised to support adhesion and growth of the different cell types. Flow‐stimulated endothelial cells grown in top channels showed threefold increase in cell alignment, compared with static controls (*n* = 3). Primary epithelial, endothelial, smooth muscle cells, fibroblasts, and pericytes expressed tissue‐specific differentiation markers in long‐term (>12 days) culture. Interactions of endothelial cells with other vascular cell types significantly enhanced endothelial barrier function under basal conditions and following stimulation with thrombin (1 U/mL, 1 h). Hydrogen peroxide exposure led to a significant increased epithelial permeability under medium and airflow and resulted in cell retraction and gap formation (*n* = 3). REVAS incorporates multiple cell types from the respiratory and vascular systems. Future steps include proteomic analysis of cells exposed to oxidative stress grown within the REVAS device to determine if the presence of vascular cell types affect endothelial response to alveolar damage. This 3D co‐culture platform, validated under flow and static conditions, can potentially be used for in vitro disease modelling and towards personalized medicine.

## A057 QUANTITATIVE INTERSTITIAL ABNORMALITIES IN SMOKERS ARE ASSOCIATED WITH INCREASED BURDEN OF CT‐BASED MEASURES OF PULMONARY HYPERTENSION

Eileen Harder^1^, Pietro Nardelli^2^, Carrie Pistenmaa^1^, Alejandro Diaz^1^, Ruben San José Estépar^2^, George Washko^1^, Bina Choi^1^, Raul San José Estépar^2^, Farbod Rahaghi^1^

^1^Brigham And Women's Hospital, Division of Pulmonary and Critical Care Medicine, Boston, USA, ^2^Brigham and Women's Hospital, Division of Radiology, Boston, USA

Pulmonary hypertension (PH) is a frequent complication of parenchymal lung disease associated with distal arterial loss on chest computed tomography (CT)1. Quantitative interstitial abnormalities (QIAs) are CT‐based high attenuation areas that may represent early interstitial lung disease and in ever‐smokers correlate with lower lung function, reduced exercise capacity, and increased mortality2,3. We hypothesized that QIA percentage would be associated with more extensive distal pulmonary arterial pruning on CT in ever‐smokers with emphysema in the Genetic Epidemiology of COPD (COPDGene) Study. Using local histogram density analysis, QIA burden was quantified on baseline CT chest scans of smokers in COPDGene, as previously described2–5. The vasculature was automatically segmented and reconstructed, and pulmonary arterial volume <5 mm^2^ in cross‐sectional area (aBV5) was normalized to total arterial vessel volumes (aTBV), as previously described1,6. Distal arterial pruning in pulmonary hypertension is reflected by reduced aBV5/aTBV volume1,6. The relationship of QIA percentage with vascular pruning was examined with Spearman's rank correlation and multivariable linear regression adjusted for age, sex, race, body mass index, local histogram‐quantified percent emphysema, total lung capacity on CT, presence of COPD (defined by post‐bronchodilator FEV1/FVC < 0.7), FEV1, and self‐reported congestive heart failure, rheumatoid arthritis, and sleep apnea. There was an inverse correlation between QIA percentage and aBV5/aTBV (*n* = 5172; *ρ* −0.35, *p* < 0.001), reflective of greater arterial pruning in patients with a higher QIA burden. In multivariable linear regression, greater QIA continued to be associated with a significant reduction in aBV5/aTBV (−0.034 aBV5/aTBV for each 5% increase in QIA, 95% CI −0.037 to −0.032; *p* < 0.001). Among the subset of smokers with obstructive lung disease, the association between increased QIA and greater distal arterial pruning persisted. QIA burden, a proposed marker of early ILD, was associated with a greater degree of CT‐measured distal arterial pruning in smokers within the COPDGene cohort. Given the co‐existence of destructive and fibrotic parenchymal diseases in this population, patients with both emphysema and QIAs are likely at high risk of PH development and should be monitored closely this potential cardiovascular complication.

## A058 VASCULAR PRUNING IN PATIENTS MEETING CRITERIA FOR IDIOPATHIC PULMONARY FIBROSIS AND ABNORMAL INVASIVE HEMODYNAMICS

Eileen Harder^1^, Pietro Nardelli^3^, Badar Patel^4^, Sirus Jesudasen^2^, Ruben San José Estépar^3^, Aaron Waxman^1^, George Washko^1^, Raúl San José Estépar^3^, Sydney Montesi^2^, Farbod Rahaghi^1^

^1^Brigham And Women's Hospital, Division of Pulmonary and Critical Care, Boston, USA, ^2^Massachusetts General Hospital, Division of Pulmonary and Critical Care Medicine, Boston, USA, ^3^Brigham and Women's Hospital, Division of Radiology, Boston, USA, ^4^Beth Israel Deaconess Hospital, Division of Cardiovascular Medicine, Boston, USA

Pulmonary hypertension (PH) in Idiopathic Pulmonary Fibrosis (IPF) is known to be associated with poor outcomes. Vascular pruning has been described in various forms of pulmonary vascular disease including patients with chronic lung disease associated PH. In this study, we sought to study small vessel arterial pruning within IPF patients who underwent right heart catheterization (RHC). Using the Mass General Brigham (MGB) Research Patient Data Repository, we identified patients with IPF who underwent both chest CT imaging with section thickness of ≤1.25 mm and concurrent RHC within a 1 year period. Medical records were reviewed to confirm the diagnosis of IPF by ILD experts. The presence of PH was defined by an elevated mean pulmonary artery pressure (mPAP) > 20 mmHg. Precapillary PH was defined as mPAP >20 mmHg with pulmonary vascular resistance of ≥3 Wood Units (WU). Post‐transplant CT and RHC data were excluded. CT imaging was processed using the Chest Imaging platform (www.chestimagingplatform.org). The vascular volume in vessels less than 5 mm^2^ of cross‐section (BV5) was divided by the total volume (TBV) to yield a fraction BV5/TBV, which has been used previously to measure relative loss of small vessels to total vascular volume and validated in multiple settings of pulmonary vascular disease. Categorical variables were assessed using Chi‐squared, continuous variables using Wilcoxon Rank‐Sum. Data is represented as median and interquartile ranges. One hundred sixty‐nine IPF patients with RHC and CT imaging were identified within the MGB system. Of these, 113 patients had mPAP >20 mmHg and 76 had elevated PVR > 3WU. There were 71 patients who met the criteria for precapillary PH with both mPAP >20 mmHg and PVR > 3WU. BV5/TBV was higher in subjects without PH (0.40 [interquartile range, 0.35–0.51]) compared to those with PH (0.35 [0.27–0.47], *p* = 0.006). Similarly, in those who met precapillary PH criteria with both elevated mPAP and PVR, there was a decrease in BV5/TBV (0.33 [0.28–0.46], *p* = 0.005). When focusing on the circulatory phases, the arterial BV5/TBV was significantly reduced in both PH and precapillary PH groups compared to normal controls (aBV5/TBV of 0.37 [0.28–0.49] and 0.37 [0.27–0.47] vs. 0.43 [0.35–0.52], *p*‐values of 0.005 and 0.003, respectively). Conversely, only a trend toward differences between venous BV5/TBV was observed (PH 0.34 [0.24–0.45] and precapillary PH 0.32 [0.25–0.45] vs. normal controls 0.36 [0.30–0.48], *p*‐values of 0.06 and 0.06, respectively). In this retrospective study of patients with IPF who underwent contemporary CT imaging and RHC, the presence of an elevated mPAP alone and the separate presence of precapillary PH with elevated mPAP and PVR were associated with increased global vascular pruning. These measures, which can be automatically extracted from CT imaging, may serve as a noninvasive screening method for pulmonary vascular disease in this cohort.

## A059 RIGHT VENTRICULAR MYOCARDIUM REMODELING IN PULMONARY ARTERIAL HYPERTENSION IS SEX SPECIFIC AND OVARIAN HORMONE DEPENDENT

Becky Hardie^1^, Jessica Huberts^1^, Daniela Valdez‐Jasso^1^

^1^University Of California, San Diego, La Jolla, USA

Pulmonary arterial hypertension (PAH) a progressive vasculopathy that manifests as sustained elevated arterial pressures and irreversible structural remodeling. Increased pulmonary vascular resistance leads to, and later exacerbates, pressure overload on the right ventricle (RV). Previous studies have shown that elevated RV pressures are associated with increased myocardial diastolic stiffness. PAH is significantly more predominant in women; however, despite the 2–4 times higher incidence in women, premenopausal women maintain better cardiac function. Due to the less severe phenotype seen in premenopausal women, we hypothesize that the RV myocardium remodels in a sex‐ and estrogen‐dependent manner in PAH. PAH was induced in male, intact female, and ovariectomized female (OVX) Sprague‐Dawley rats by a 20 mg/kg injection of Sugen 5416, followed by 3 weeks of hypoxia and 1, 5, or 9 weeks of normoxia. Immediately after hemodynamic measurements to confirm hypertension, the heart was harvested, flushed, and the RV free wall excised. A square sample aligned along the apex‐to‐outflow (AOT) direction was cut from the mid‐wall. RV samples were subjected to quasi‐static displacement‐controlled biaxial testing and the resulting stress‐strain data were fit by a Fung‐type exponential strain energy function. Stress‐strain relations were compared using two‐factor and three‐factor analysis of covariance (ANCOVA, α = 0.05) with a log transformation of the stress in JMP. Total collagen content in the RV tissue surrounding the biaxially‐tested sample was quantified with a hydroxyproline assay. While RV end‐systolic and end‐diastolic pressures increase in PAH by similar amounts in male, intact female, and OVX rats, SuHx‐treatment impacts tissue‐level mechanical properties in a sex‐specific and ovarian hormone‐dependent manner. At baseline, female RVs are slightly stiffer than males, and OVX female RVs in particular have a higher collagen content. By 4 weeks post‐disease induction, the RV has significantly stiffened in all groups (*p* < 0.0001). At 8 weeks post‐induction, male RVs have continued to stiffen (*p* < 0.0001), unlike the RVs from intact females and OVX females. Collagen content increases in all groups, with the largest increase from baseline observed in males and the smallest in OVX. OVX female RVs experience an additional increase in stiffness by 12 weeks post‐disease induction (*p* < 0.0001), while intact female RVs continue to maintain their stiffness and male RVs experience a slight decrease from their peak stiffness. This study suggests that the organ‐level chamber stiffening seen in the overloaded RV is at least in part due to passive myocardial tissue stiffening. Increased male RV stiffness in PAH has previously been attributed to a stiffer collagen extracellular matrix, which is supported by the larger amount of collagen present in the SuHx‐treated RVs in all groups. The lack of progressive stiffening in the intact female RVs after the initial remodeling due to pressure overload may explain why premenopausal women are better able to preserve cardiac function in PAH, compared with OVX females and males which both experience a secondary increase in RV stiffness. The delayed additional stiffening in OVX females compared to males may suggest independent contributions from both sex and ovarian hormones in RV remodeling in PAH.

## A060 MANAGEMENT OF PULMONARY HYPERTENSION IN PREGNANCY—EXPERIENCE FROM CHINA

Hong Gu^1^

^1^Beijing Anzhen Hospital, Beijing, China

Pulmonary hypertension is known to complicate pregnancy and historically been associated with unacceptably high maternal mortality. Normal physiological changes during pregnancy, such as an increase in plasma volume, heart rate, stroke volume, cardiac output, hypercoagulability, and inferior vena cava compression by the uterus, as well as a decrease in blood pressure and systemic vascular resistance, are not well tolerated in patients with PH and can result in right ventricular failure. Cardiovascular risk assessment tools for pregnant patients, including Modified World Health Organization classification and Cardiac Disease in Pregnancy CARPREG II Risk, categorize patients with PH as high‐risk for cardiovascular complications, which indicates the risk of maternal cardiovascular complications is extremely high. Thus, pregnancy is contraindicated in PAH patients. If pregnancy occurs, termination should be discussed. Notably, patients with Eisenmenger syndrome require special consideration for high maternal mortality (20%–50%). The PAH‐directed therapy during pregnancy is tricky. Parenteral prostaglandins can be combined with oral PDE‐5 inhibitors in pregnancy and Endothelin receptor blockers and soluble guanylate cyclase stimulator are pregnancy category X and should not be used in pregnancy. Also, delivery is generally completed in consultation with a multidisciplinary team, including PAH specialists in pulmonology, cardiology, maternal fetal medicine, and cardiac and/or obstetric anesthesiology, the nursing team and additional ECMO team, neonatologists, cardiac intensive care unit. Previous studies described the clinical characteristics and outcomes of pregnancy in women with PH. The results showed high mortality even with the application of PAH‐targeted therapy. Different from European and American countries, PAH associated with congenital heart disease (PAH‐CHD) is the most common type PH in adult patients in China. As a referral tertiary medical center for high risk pregnancy and fetal health, the number of pregnant women with CHD is increasing in our hospital. Patients with eisenmenger syndrome took a large proportion and showed higher mortality according to our previous study. And now, there is an ongoing multinational registry study of PH with pregnancy in China to standardize the diagnosis and treatment process and optimize the prognosis of these patients.

## A061 MANAGING PULMONARY HYPERTENSION IN CHILDREN—EXPERIENCE FROM CHINA

Hong Gu^1^

^1^Beijing Anzhen Hospital, Beijing, China

Pulmonary arterial hypertension associated with congenital heart disease (PAH‐CHD) and idiopathic/heritable pulmonary arterial hypertension (IPAH/HPAH) are the two most common types of PAH in Chinese pediatric patients. The prognosis of children with PAH was extremely poor in China before the era of targeted therapy. In recent years, targeted therapy for pediatric PAH has made remarkable progress to effectively delay the progression of disease and improve patient's survival and the overall survival rate of Chinese children with PAH has improved to resemble Europe and America. However, there are still challenges in the treatment of pediatric PAH: (1) late diagnosis and treatment; (2) off‐label use is very common, and only one targeted drug has pediatric indication in China; (3) unstandardized follow‐up; (4) the drugs are costly and the proportion of combination therapies is low. Previous clinical studies from China showed the use rate of targeted drugs in children with PAH is inadequate, and the proportion of combination therapies is low. Facing these challenges in China, there's lot of developments in diagnosis and treatment of pediatric PH in China. Guidelines and consensuses on PH in children have been released successively from 2011 to 2022 in China. With continuous exploration, there are fruitful academic achievements from multiple approaches: (1) Beijing Anzhen Hospital has the largest pediatric cohort of right heart catheterization in China and data over the past 15 years was recently reviewed. We explored the acute effect of inhaled Iloprost in Children with PAH associated with simple CHD and summarized our experience of pulmonary hypertension crisis in children with PAH during catheterization; (2) the genetic factors in Chinese children with IPAH/HPAH and persistent pulmonary hypertension in Neonates were also investigated; (3) Chinese PAH patients is featured with postoperative PAH because there is a gap between China and developed countries for the surgical indication. However, the prognosis of postoperative PAH is similar with patients with Eisenmenger syndrome according to our previous study; (4) besides PAH‐targeted medications, surgical procedures, including Potts shunt and lung transplantation, are performed in certain pediatric centers. Despite the huge progress in China recently, multi‐center studies and long term follow up are needed to optimize the prognosis of pediatric PAH patients.

## A062 TREPROSTINIL EFFECTIVENESS IN HIGHER‐RISK PEDIATRIC PATIENTS WITH IDIOPATHIC AND HERITABLE PULMONARY ARTERIAL HYPERTENSION

Yuan He^1^, Qiangqiang Li^1^, Chen Zhang^1^, Bradley B Keller^2^, Hong Gu^1^

^1^Beijing Anzhen Hospital, Beijing, China, ^2^Cincinnati Children's Heart Institute, Cincinnati Children's Hospital Medical Center, OH, USA

Little is known about the effectiveness of treprostinil in higher‐risk pediatric patients with various PAH genotypes. This study was designed to investigate the prognosis of higher‐risk pediatric patients with IPAH and HPAH (IPAH/HPAH) following treprostinil therapy. Children with IPAH/HPAH who were stratified as higher‐risk and treated with treprostinil in our center were included as study cohort. Meanwhile, those who only received oral medications were included as reference cohort. All patients in the study cohort received PAH‐related genotyping. Survival was defined as no death. Event‐free survival was defined as no death, Potts shunt or atrial septostomy. Forty‐nine children (median age 7.7 (IQR 4.2, 11.5) years, female 65.3%) were included in the study cohort and forty‐eight children were included in the reference cohort. 84% of the study cohort had genetic disorders after genetic testing with a dominance of BMPR2 and ACVRL1 mutations. After a median therapy duration of 5.56 (IQR 2.66, 11.12) months, all patients were alive with significant improvements in clinical characteristics. One‐, two‐ and 3‐year survival rate were 91%, 84%, and 69%, respectively with a median follow‐up duration of 19.17 (IQR 9.7, 29.79) months, which was significant superior to the reference cohort (*p* = 0.038). Multivariate cox regression analysis identified WHO‐FC after therapy as a predictor for survival. There was no significant difference in survival among patients with different genotypes. Treprostinil can significantly improve the prognosis in children with IPAH/HPAH who are in higher‐risk despite genetic backgrounds.

## A063 WHAT CAUSES THIS PATIENT TO HAVE PULMONARY HYPERTENSION: CHRONIC MYELOGENOUS LEUKEMIA OR DASATINIB?

Yingxin He^1^

^1^The Second Affiliated Hospital Of Harbin Medical University, Harbin, China

Pulmonary hypertension (PH) is a major complication of several haematological disorders. Chronic myeloproliferative diseases (CMPDs) associated with pulmonary hypertension have been included in group five of the clinical classification for pulmonary hypertension, corresponding to pulmonary hypertension for which the aetiology is unclear and/or multifactorial. Precapillary PH is usually diagnosed late in the course of the haematological disease, while CTEPH is usually diagnosed earlier and may even be concurrent with the haematological diagnosis. Dasatinib, which is used for the treatment of CML, is considered to be a probable cause of PAH. High haematocrit levels with hyperviscosity, thrombocytosis and splenectomy may contribute, among other mechanisms, to the increased rate of thrombotic events in patients with CMPDs, especially polycythaemia vera. PAH‐like disease associated with CMPD was found to be related to myeloid metaplasia, suggesting that pulmonary myeloid infiltration and pulmonary capillary obstruction by megakaryocytes with stasis and secondary microthrombosis may contribute to the pulmonary vascular disease. When PH is diagnosed, secondary causes which may contribute to the elevated pulmonary pressure, such as anaemia and heart failure, should be recognised and treated. However, when PH persists, treatment is not yet established. Anticoagulant drugs should be administered carefully because of the potential risk of haemorrhagic complications. Cytoreductive treatment should be used in association with symptomatic treatment of PH, such as oxygen and diuretics. There are no data on the effectiveness of specific PAH therapies in these patients and randomised control trials are needed. The prognosis of PH associated with CMPDs remains poor. However, pulmonary endarterectomy is the treatment of choice in eligible patients with proximal CTEPH. What is the reason why a 27‐year‐old girl with CML who has used dasatinib gets better after taking targeted drugs and stopping dasatinib?

## A064 TRANSCRIPTOMIC SIGNATURE OF THE RIGHT VENTRICLE FOLLOWING SUPRACORONARY AORTIC BANDING (SAB) IN THE RAT; BNP AND ANP CORRELATE WITH BOTH HAEMODYNAMICS AND GENE EXPRESSION

Charles Hindmarch^1^, Rachel Bentley, Ping Xiong, Lian Tian, Stephen Archer

^1^Queen's University, Kingston, Canada

Pulmonary Hypertension (PH) is divided into five groups based on clinical observation. Group 2 (GP) PH is classified as secondary to left heart disease and is the most prevalent form of PH with an incidence of 250 cases per 100,000 population. Supracoronary aortic banding (SAB) is a model of Group 2 PH that is created when a titanium clip is placed distal to the aortic valve ascending aorta of rats to induce diastolic dysfunction. We wanted to establish the impact of GP2 PH on the molecular signature of the right ventricle (RV) of SAB (*n* = 8; trans banding gradient 129.4 ± 9.47 mmHg) and sham (*n* = 8) rats. While there is a significant elevation in left ventricular end diastolic pressure (LVEDP; 36.1 ± 4.94 mmHg), left ventricular systolic pressure (LVSP 225.6 mmHg ± 12.7), right ventricular end diastolic pressure (RVEDP; 5.2 ± 1.33 mmHg), and right ventricular systolic pressure (RVSP; 47.5 ± 8.1 mmHg) relative to the shams, we observed high level of variability in the SAB haemodynamics. Libraries of mRNA were constructed from snap‐frozen RVs from each animal, and RNAsequencing at the 3' end of each transcript allowed the transcriptome of each condition to be identified. Because only 89 differentially regulated genes were revealed (corrected *p*‐value < 0.1), we hypothesised that the range of haemodynamics in the SAB animals may be responsible for increased noise in our RNAsequencing data, which in turn impacted the false discovery rate of the data. Circulating levels of atrial natriuretic peptide (ANP), and B‐type natriuretic peptide (BNP) have both diagnostic, and prognostic value in heart disease. When we stratified our samples according to the results of an independent qPCR analysis of the genes that encode these peptides (NPPA and NPPB respectively), we identified that both genes correlate with each other. We stratified our SAB and identified 4×SAB samples with higher expression, and 4×SAB samples with lower expression. Reanalysis of our RNAsequencing data revealed in animals with highest NPPA/NPPB RV expression compared to shams, 817 genes were significantly differentially regulated (with corrected *p*‐value < 0.1). When we compared the 4×SAB samples with lower NPPA/NPPB RV expression. Only 8 genes were significantly differentially regulated between those RV with lower NPPA/NPPB RV expression and shams. Functional analysis reveals multiple, overlapping biological pathways, including those that underpin mitochondria. These data demonstrate that NPPA/NPPB gene expression is a useful method to stratify SAB samples and reduce the noise in sensitive omic experiments like RNAseq.

## A065 PULMONARY HYPERTENSION ASSOCIATED WITH BRONCHOPULMONARY DYSPLASIA IN ADULTS

Stephanie Hon^1^, Harrison Farber^1^

^1^Tufts Medical Center, Boston, USA

Bronchopulmonary dysplasia (BPD), neonatal lung injury associated with prematurity and low birth weight, leads to chronic pulmonary disease into adulthood. Pulmonary hypertension(PH) occurs in 21%–42% of BPD patients and has ~38% mortality at 1 year. In most survivors, PH improves but only resolves in 20%. Although BPD‐PH is well‐studied in neonatology and pediatrics, there is little literature on BPD‐associated PH(BPD‐PH) in adults. We present a series of three adult patients with BPD‐PH and describe their clinical course and response to pulmonary vasodilators. Case 1: 38‐year‐old female with BPD‐PH at birth that resolved was admitted for acute hypoxic respiratory failure in the setting of pneumonia. After resolution of the pneumonia, dyspnea on exertion persisted. Right heart catheterization (RHC) revealed mean pulmonary artery pressure(mPAP) 42 mmHg, pulmonary capillary wedge pressure (PCWP) 11 mmHg, cardiac index (CI) 3.34 L/min and pulmonary vascular resistance (PVR) 5.5 WU: moderate pre‐capillary PH, thought to be related to underlying BPD. She was treated with Ambrisentan and Tadalafil; on surveillance RHC, PVR decreased to 3.7 WU; functional class (FC) had also improved from FCIII to FCII. Case 2: 33‐year‐old male born 4 weeks premature with BPD, persistent fetal circulation and required tracheostomy for chronic respiratory failure until age 7. He had abnormal echocardiography throughout childhood but was formally diagnosed with PH at age 15; RHC demonstrated mPAP 55 mmHg, normal PCWP, and markedly elevated PVR. He was started on Bosentan monotherapy with improvement in mPAP to 37 mmHg and PVR to 3.3 WU with hemodynamic stability on surveillance RHCs. Bosentan was changed to Macitentan for convenience, but 15 years later, echocardiography and hemodynamics worsened. Tadalafil was added with improvement in exertional capacity and normalization of RV by echocardiography. Case 3: 38‐year‐old male with BPD requiring oxygen supplementation until age 2 and emphysema was admitted for COPD exacerbation; echocardiography was abnormal. RHC showed mPAP 27 mmHg, PCWP 7 mmHg, CI 3.2 L/min and PVR 5.1 WU. He was treated with Ambrisentan and Tadalafil. On surveillance RHC, PVR decreased to 2.4WU; FC had improved from FCIII to FCII. With advances in neonatal care and improved viability of extremely premature infants, caring for patients with chronic lung disease including BPD‐PH, will become increasingly common for adult pulmonologists. Treatment of BPD‐PH is individualized, with little data in children or adults. This case series is the first to use pulmonary vasodilators for treatment of BPD‐PH. While initial results are promising, further studies are needed to fully evaluate treatment for adult BPD‐PH patients.

## A066 EFFECTIVENESS OF MACITENTAN‐BASED DUAL COMBINATION THERAPY IN PULMONARY ARTERIAL HYPERTENSION IN CHINESE PATIENTS: THE SUBGROUP ANALYSIS FROM A RETROSPECTIVE REAL‐WORLD STUDY

Hong Gu^1^, Yu‐Cheng Chen^2^, Hai‐Long Dai^3^, Chun‐Li Liu^4^, Qiu‐Shang Ji^5^, Yun‐Shan Cao^6^, Jiang Li^7^, Jing Xiao^8^, Xin‐Chao Luo^9^, Jian‐Min Zhuo^9^

^1^Beijing Anzhen Hospital, Beijing, China, ^2^West China Hospital of Sichuan University, Chengdu, China, ^3^Yan'an Hospital Affiliated to Kunming Medical University, Kunming, China, ^4^The First Affiliated Hospital of Guangzhou Medical University, Guangzhou, China, ^5^Qilu Hospital of Shandong University, Jinan, China, ^6^Gansu Provincial Hospital, Lanzhou, China, ^7^The Second Xiangya Hospital of Central South University, Changsha, China, ^8^Xi'an Janssen Pharmaceutical Ltd., Beijing, China, ^9^Janssen China R&D, China

This subgroup analysis aims to evaluate the effectiveness of macitentan‐based dual therapy in a real‐world clinical setting in China. We retrospectively collected medical records data among pulmonary arterial hypertension (PAH) patients who received macitentan‐based dual therapy from seven top centers in China. Effectiveness was evaluated via World Health Organization functional class (WHO‐FC), 6‐min walk distance (6MWD), and N‐terminal pro‐brain natriuretic peptide (NT‐proBNP) by measuring changes from baseline to follow‐up within 3–7 months after macitentan initiation. McNemar‐Bowker test was used to compare the baseline and follow‐up visit for WHO‐FC, while Wilcoxon signed‐rank test was used for 6MWD and NT‐proBNP. A total of 46 patients received macitentan‐based combination therapy. 32 (69.6%) patients who treated with macitentan + PDE‐5i and 14 (30.4%) patients received macitentan + selexipag. Improvement of patient proportion was found in both groups in WHO‐FC I and II among who performed this assessment at baseline/follow‐up: 78.2%/92.9% for macitentan + PDE‐5i; 50.0%/81.8% for macitentan + selexipag. The 6MWD improved in both groups with the mean (SD) change being 39.3 (56.4) m for macitentan + PDE‐5i (*p* < 0.001), and 46.4 (63.2) meters for macitentan + selexipag (*p* = 0.017). Reduction was observed in NT‐proBNP in macitentan + PDE‐5i: −542 (974.6) ng/L (*p* = 0.001), while increased in macitentan + selexipag: 188 (870.5) ng/L without significance. PAH patients treated with macitentan‐based dual therapies (macitentan + PDE‐5i or macitentan + selexipag) showed clinical benefit in Chinese PAH patients. This analysis is supplementary evidence of effectiveness of macitentan‐based dual therapy for Chinese PAH patients.

## A067 INTEGRATIVE MULTIOMICS IN THE LUNG REVEALS A PROTECTIVE ROLE OF ASPORIN IN PULMONARY ARTERIAL HYPERTENSION

Jason Hong^1^

^1^Ucla, Los Angeles, California, USA

Integrative multiomics can elucidate pulmonary arterial hypertension (PAH) pathobiology but procuring human PAH lung samples is rare. Here, we leverage transcriptomic profiling and deep phenotyping of the largest multicenter PAH lung biobank to date (96 disease and 52 control) by integration with clinicopathologic data, genome‐wide association studies, Bayesian regulatory networks, single‐cell transcriptomics, and pharmacotranscriptomics. We identify two potentially protective gene network modules associated with vascular cells, and we validated ASPN, coding for asporin, as a key hub gene that is upregulated as compensatory response to counteract PAH. Specifically, we find that asporin is upregulated in lungs and plasma of multiple independent PAH cohorts and correlates with reduced PAH severity. We show asporin inhibits proliferation and TGF‐β/pSMAD2/3 signaling in pulmonary artery smooth muscle cells from PAH lungs. Finally, we demonstrate in Sugen‐hypoxia rats that ASPN knockdown exacerbated PAH while recombinant asporin attenuated PAH. Our integrative systems biology approach to dissect the PAH lung transcriptome uncovered asporin as a novel protective target with therapeutic potential in PAH.

## A068 CONSISTENCY OF THE EFFICACY AND SAFETY PROFILE OF SOTATERCEPT ACROSS 2022 ESC/ERS RISK STRATA: A POST HOC ANALYSIS OF THE STELLAR STUDY

Marc HUMBERT^1^, David Badesch^2^, H. Ardeschir Ghofrani^3^, J. Simon Gibbs^4^, Mardi Gomberg‐Maitland^5^, Vallerie McLaughlin^6^, Ioana Preston^7^, Rogerio Souza^8^, Aaron Waxman^9^, Ekkehard Grünig^10^, Grzegorz Kopeć^11^, Gisela Meyer^12^, Stephan Rosenkranz^13^, Marc Humbert^14^, Jianxin Lin^15^, Amy Johnson‐Levonas^15^, Maria Loureiro^15^, Samuel Kim^15^, Marius Hoeper^1^

^1^Hannover Medical School and the German Center for Lung Research, Hannover, Germany, ^2^University of Colorado, Anschutz Medical Campus, Aurora, USA, ^3^Department of Internal Medicine, Justus‐Leibig‐University Giessen, Universities of Giessen and Marburg Lung Center (UGMLC), Member of the German Center for Lung Research (DZL), Giessen, Germany, ^4^National Heart and Lung Institute, Imperial College London, London, UK, ^5^George Washington University, Washington, USA, ^6^University of Michigan, Ann Arbor, USA, ^7^Tufts Medical Center, Boston, USA, ^8^Instituto do Coracao, Hospital das Clinicas da Faculdade de Medicina da Universidade de Sao Paulo, Sao Paulo, Brazil, ^9^Brigham and Women's Hospital, Boston, USA, ^10^Thoraxklinik‐Heidelberg and the German Center for Lung Research, Heidelberg, Germany, ^11^The Pulmonary Circulation Center, Department of Cardiac and Vascular Diseases, Jagiellonian University Medical College, John Paul II Hospital in Krakow, Krakow, Poland, ^12^Irmandade da Santa Casa de Misericordia de Porto Alegre, Porto Alegre, Brazil, ^13^Department of Cardiology, and Cologne Cardiovascular Research Center (CCRC), Heart Center, University Hospital Cologne, Cologne, Germany, ^14^Universite Paris‐Saclay, INSERM Unite Mixte de Recherche en Sante 999, Hopital Bicetre (Assistance Publique‐Hopitaux de Paris), Le Kremlin‐Bicetre, France, ^15^Merck & Co., Inc., Rahway, USA

Risk stratification plays an important role in the clinical management of patients with pulmonary arterial hypertension (PAH). Sotatercept is an activin signaling inhibitor under investigation for PAH. Aims: This post hoc analysis compared the efficacy and safety profile of sotatercept versus placebo across the 2022 European Society of Cardiology/European Respiratory Society (ESC/ERS) risk strata. Participants in STELLAR, a phase 3 trial (NCT04576988), were randomized 1:1 to sotatercept (0.3 up to 0.7 mg/kg once every 3 weeks) or placebo added to background therapies. Eligible participants had PAH and World Health Organization functional class (WHO‐FC) II‐III, pulmonary vascular resistance (PVR) ≥ 400 dyn·s·cm^−5^, and 6‐min walk distance (6MWD) 150–550 m. Participants were stratified based on ESC/ERS 2022 risk categories at baseline (low, intermediate‐low, intermediate‐high, high). Continuous endpoints were analyzed by aligned‐rank stratified Wilcoxon with WHO‐FC and background therapy as strata. Hodges–Lehmann (HL) location‐shift estimates for between‐group difference in change from baseline (95% CI), *p*‐value were calculated. Categorial endpoints were analyzed by Cochran–Mantel–Haenszel. Time to death or nonfatal clinical worsening event was analyzed by log‐rank, stratified by randomization factors. Hazard ratios (HR) were derived from a Cox proportional model with treatment group as covariate, stratified by randomization factors. Cumulative safety up to data cut‐off was descriptively summarized across subgroups. Analyses were not adjusted for multiplicity. In STELLAR, 95 (29.6%), 142 (44.2%), 81 (25.2%), and 3 (0.9%) participants were in the low, intermediate‐low, intermediate‐high and high‐risk strata at baseline, respectively. The high‐risk subgroup was not analyzed due to small number of participants (*n* = 3 placebo, *n* = 0 sotatercept). The treatment effects generally appeared more pronounced in the higher versus lower risk strata. The between‐group HL shift estimates for changes from baseline in 6MWD (range from low to intermediate‐high: 12.1 [NS], 42.0 [*p* < 0.001], and 83.3 m [*p* < 0.001]), PVR (range: −154.5, −231.7, and −373.2 dyn·s·cm^−5^ [*p* < 0.001 for all]) and NT‐proBNP (range: −125.4, −422.8, and −1746.5 pg/mL [*p* < 0.001 for all]) favored sotatercept treatment. The proportions of participants who showed improvement in WHO‐FC or ESC/ERS risk status at week 24 were significantly higher with sotatercept versus placebo in the intermediate‐low and intermediate‐high risk subgroups (*p* ≤ 0.037; p = NS for the low‐risk subgroup). Prolonged event‐free survival was seen with sotatercept versus placebo in the intermediate‐low and intermediate‐high subgroups (HRs 0.112 and 0.268, respectively; *p* ≤ 0.010 for both), as well as the low‐risk subgroup (HR 0.000; *p* = 0.010). The overall incidences of adverse events (AEs), treatment‐related AEs, and serious AEs with sotatercept versus placebo across the various subgroups were comparable to that observed in the overall trial population. Significant improvements in exercise capacity, hemodynamics, functional parameters, goal attainment and outcomes were seen in the intermediate‐low and intermediate‐high risk subgroups following sotatercept treatment with acceptable safety/tolerability profile. The low‐risk subgroup demonstrated improvements in PVR and NT‐proBNP at week 24 as well as prolonged event‐free survival. These findings demonstrate the potential therapeutic benefit of sotatercept in patients with PAH across a wide range of risk strata. The role of sotatercept in patients at high risk is currently being evaluated in ongoing trials.

## A069 LDHA‐MEDIATED LACTATE OVERPRODUCTION PROMOTES PULMONARY VASCULAR REMODELING AND PULMONARY HYPERTENSION THROUGH SMOOTH MUSCLE‐SPECIFIC LACTYLATION OF TOP1 AND EMILIN1

Lifeng Jiang^1^, Iryna Zhyvylo^1^, Dmitry Goncharov^1^, Leyla Teos^1^, Derek Lin^1^, Lisa Franzi^1^, Aisha Saiyed^1^, Sanjana Neeli^1^, Nicholas Kenyon, Paul Wolters^2^, Horace Delisser^3^, Tatiana Kudryashova^4^, Elena Goncharova^1^

^1^University of California, Davis, Davis, USA, ^2^University of California, San Francisco, San Francisco, USA, ^3^University of Pennsylvania, Philadelphia, USA ^4^University of Pittsburgh, Pittsburgh, USA

Pulmonary arterial hypertension (PAH) is a complex and progressive disease characterized by the remodeling of small pulmonary arteries (PAs). Pulmonary vascular remodeling occurs due to abnormal proliferation and survival of resident pulmonary vascular cells, including PA smooth muscle cells (PASMCs), endothelial cells (PAECs), and adventitial fibroblasts (PAAFs). PASMCs in PAH have a metabolic shift to glycolysis to support PAH‐specific hyper‐proliferative, apoptosis‐resistant phenotype. This leads to overproduction of lactate, the role of which in PAH remains unclear. We found that lactate dehydrogenase A (LDHA), an enzyme that converts pyruvate into lactate, was overexpressed in the smooth muscle (SM) α‐actin (SMA)‐positive areas of small muscular PAs in lungs of patients with PAH and rats and mice with SU5416/hypoxia (SuHx)‐induced PH compared to respective controls. Early‐passage distal human PAH PASMCs, but not PAECs and PAAFs, had significantly higher mRNA and protein levels of LDHA compared to control cells, which was associated with significant increase of intracellular lactate levels and unstimulated hyper‐proliferation. In vitro, reduction of the lactate production by siRNA‐induced depletion of LDHA suppressed proliferation, and induced apoptosis in human PAH PASMCs, which effects were prevented by the lactate supplementation. In vivo, mice with smooth muscle‐specific Ldha knockout (SM22α‐Ldha−/−) were protected from SuHx‐induced pulmonary vascular remodeling, PH, and right ventricular (RV) hypertrophy, suggesting that dysregulated LDHA‐lactate signaling facilitates PASMC hyper‐proliferation and survival, pulmonary vascular remodeling, and PH. Lactate modulates cellular responses by providing a substrate for the posttranslational modification lysine lactylation (Kla) of histone and non‐histone proteins. Comparative analysis of pulmonary vascular cells from non‐diseased and PAH human lungs demonstrated that Kla is markedly higher in PAH PASMCs, but not PAECs or PAAFs, than in respective non‐diseased controls. Proteomic analysis of anti‐Kla antibody precipitates identified 12 non‐histone proteins that were hyper‐lactylated in human PAH PASMCs compared to cells from non‐diseased subjects. Further validation revealed that hyper‐lactylation triggered over‐accumulation of DNA topoisomerase 1 (TOP1) and a deficiency of elastin microfibril interfacer 1 (EMILIN1) in PASMCs from PAH lungs, which was required for increased cell proliferation and for upregulation of pro‐proliferative/pro‐survival Yap/Taz, Akt‐mTOR, and TGFβ1. Additional observations revealed that human PAH PASMCs had significantly elevated lactate secretion; and exogenous lactate, in turn, induced significant proliferation of PAECs and PAAFs. Treatment with LDHA inhibitor oxamate significantly reduced cell proliferation and enhanced apoptosis of human PAH PASMCs in vitro, and reversed upregulation of TOP1 and Akt in small muscular PAs, pulmonary vascular remodeling, PH, and RV hypertrophy in mice with SuHx‐induced PH. Taken together, our data demonstrate that an overproduction of lactate driven by LDHA overexpression promotes proliferative, apoptosis‐resistant PASMC phenotype, pulmonary vascular remodeling, and PH via hyper‐lactylation and over‐accumulation of TOP1, deficiency of EMILIN1, and subsequent activation of Yap/Taz, Akt/mTOR, and TGFβ1 signaling pathways. Our findings also suggest that targeting the LDHA‐lactate‐TOP1/EMILIN1 axis could represent potentially attractive therapeutic strategy to treat established pulmonary vascular remodeling and PAH.

## A070 RESISTIN REGULATES NLP3 INFLAMMASOME IN PULMONARY HYPERTENSION

Roger Johns^1^, Udeshika Kariyawasam^1^, John Skinner, Paul Hassoun, Qing Lin

^1^Johns Hopkins University School of Medicine, Baltimore, Maryland, USA 

Human resistin (Hresistin) and its rodent homologue resistin‐like molecule (RELM)‐α has been identified to play a central role in inflammatory mechanisms of a wide array of autoinflammatory diseases including pulmonary hypertension (PH). However, it is unclear how Hresistin/RELMα's diverse inflammatory effects are integrated or how they amplify and sustain inflammation to induce the disease progression. We reported Bruton's Tyrosine Kinase (BTK) as a binding partner of RELMα/Hresistin and the mediator of chemokine activities of RELMα/Hresistin in myeloid cells. We also reported that Hresistin/RELMα activates high‐mobility group box‐1 (HMGB1) in PH. Both BTK and HMGB1 are regulators of the NLRP3 inflammasome that was recently suggested to be central to the vascular inflammation in PH. Here, we show novel understanding of Hresistin/RELM‐ α as a critical regulator of NLRP3 inflammasome in both priming and activation pathways by using both in‐vitro and in‐vivo studies. Human macrophages were stimulated with recombinant Hresistin protein with or without inhibitors of Hresistin, HMGB1, BTK and NLRP3. The NLRP3 inflammasome priming and activation pathways were determined in Hresistin treated immune cells. To determine which type of immune cells, express the highest level of NLRP3 in lungs, performing immunohistology staining was done with NLRP3 and or MAC2 (macrophage), MPO (neutrophils) and CD79b (B cell) markers using lung sections from mice after 4 days of hypoxia and from PH patients. Furthermore, co‐localization of NLRP3 and BTK was checked in lung sections of WT and RELMα‐K/O mice kept in 4 days of hypoxic condition. To link NLRP3 inflammasome activation and PV‐SMC proliferation, we measured proliferative responses of macrophage co‐cultured PV‐SMC. Hresistin upregulated the expression and secretion of HMGB1 in human macrophages, leading to the expression of NLRP3, pro‐capsase‐1, and pro‐IL‐1β in macrophages. This was prevented by the Hresistin blocking antibody or HMGB1 antagonist Box‐A. Hresistin further bound to BTK causing its autophosphorylation and the subsequent phosphorylation and activation of NLRP3, leading to the cleavage and activation of capsase‐1, IL‐1β, and IL‐18. Blocking BTK, NLRP3 or Hresistin prevented this activation pathway. To confirm Hresistin/RELMα priming and activation of NLRP3 in in vivo inflammatory disease, we studied rodent and human pulmonary hypertension (PH). Chronic hypoxia‐induced PH showed RELMα‐dependent upregulation of BTK and NLRP3 in the mouse lung tissues and was linked to vascular remodeling pathways in vivo. Further, co‐localization of Hresistin/RELM‐α, NLRP3 and BTK was increased in PH patients' lungs. Hresistin/RELM‐α regulates NLRP3 inflammasome in macrophages, and the secreted IL‐1β and IL‐18 mediate the post‐injury proliferative responses, which may be the mechanism of how Hresistin/RELMα amplifies and sustains inflammation to induce vascular remodeling over time for PH development. Our data indicate a novel concept that Hresistin/RELMα is a critical part of both the priming (via HMGB‐1) and activation (via BTK phosphorylation) of the NLRP3 inflammasome and may be essential to the regulation of NLRP3 inflammasome in a wide array of autoinflammatory diseases. We show Hresistin is a potential therapeutic target for NLRP3‐driven inflammatory disease.

## A071 CLUSTER ANALYSIS IDENTIFIES NOVEL LUNG DISEASE‐PULMONARY HYPERTENSION SUB‐PHENOTYPES: IMPLICATIONS FOR TREATMENT RESPONSE

Shelsey Johnson^1^

^1^Brigham And Women's Hospital, Boston, Massachusetts, USA

Patients with pulmonary hypertension associated with chronic lung disease or hypoxia (Group 3 PH) experience a mortality risk surpassing those with pulmonary arterial hypertension (PAH) by fivefold. Currently, treatment options are limited for these patients as clinical trials repurposing PAH therapies to Group 3 PH patients have by‐and‐large failed to demonstrate consistent benefit. However, the clinical heterogeneity of Group 3 PH suggests robust phenotyping may be utilized to detect treatment responsive subgroups. We hypothesized that cluster analysis would identify sub‐phenotypes with differential responses to oral PAH therapy. Two k‐means analyses were performed on a national cohort of U.S. Veterans with Group 3 PH; an ‘inclusive model’ (I) of all treated patients (*n* = 196) and a ‘hemodynamic model’ (H) limited to patients with right heart catheterization (RHC) before starting PAH therapy (*n* = 112). Survival analysis was performed to assess the primary composite outcome of select organ failures or death by cluster. Untreated Group 3 PH patients (*n* = 196) were matched to treated clusters to evaluate within cluster treatment effects. Sensitivity analyses were performed with Fine‐Gray sub‐distribution hazard models for respiratory failure with death as a competing risk. In a cohort of 392 Veterans (97.7% male) with Group 3 PH, RHC was performed in *n* = 137 (34.9%) patients; *n* = 112 (57.1%) treated and *n* = 25 (12.8%) untreated. Three distinct clusters of Group 3 PH patients were identified. In the ‘inclusive model’ (C1I = 43, 21.9%; C2I = 102, 52.0%; C3I = 51, 26.0%) chronic lung disease and pulmonary function test parameters were most important in determining cluster assignment. Echocardiographic features of PH including elevated right ventricular systolic pressure estimates were present across Cluster 1‐3I, but not significantly different across clusters. In the ‘hemodynamic model’, underlying lung disease distributed more evenly across clusters and RHC data was particularly important to determining cluster assignment (C1H = 44, 39.3%; C2H = 43, 38.4%; C3H = 25, 22.3%). Patients were followed for 3.4 ± 2.6 years after the start of PAH therapy and experienced significantly different survival trajectories. In the ‘hemodynamic model’, C1H experienced the greatest hazard for respiratory failure or death compared to C3H (HR 6.1 [95% CI 3.2, 11.8]). Cluster assignment significantly determined treatment response (*p* = 0.006). Treated patients in C1H and C2H had worse outcomes compared to untreated patients, whereas the effect of treatment on C3H was equivocal (HR 3.2 [95% CI 2.1–5.0]; HR 1.6 [95% CI 1.1, 2.5]; HR 1.2 [95% CI 0.6, 2.3]). In competing risk sensitivity analyses, the hazard associated with treatment was equivocal across all clusters, both for the ‘inclusive’ and ‘hemodynamic’ models. Cluster analysis successfully identifies novel sub‐phenotypes of Group 3 PH patients with distinct clinical trajectories and responses to treatment. However, despite agnostic methods, we did not identify a sub‐phenotype with a favorable response to PAH therapy. In the absence of definitive prospective data documenting efficacy, these results reinforce concern for off‐label use of repurposed therapies. Additionally, these results raise the possibility that the application of similar methodologies to prospective randomized clinical trial populations, unbiased by treatment indication, may be better‐positioned to expose clinical profiles that inform treatment response.

## A072 CASE REPORT: EXOME 2P 13.3 NFU1 ALTERATION IN AN INFANF WITH PULMONARY ARTERIAL HYPERTENSION AND POOR OUTCOME

Ernesto JUANEDA^1^, Juan Diaz, Alejandro Peirone, Leonardo Meinarde, Laura Roganti, Romina Rinaudo, Lucrecia Bardossy

^1^Private University Hospital Cordoba, Córdoba, Argentina

Pulmonary arterial hypertension in children is defined as mean pulmonary artery pressure >20 mmHg at cardiac catheterization. Screening includes physical examination, laboratory, multimodality imaging, cardiac catheterization, and genetic study. Describe genetic result in an infant with pulmonary arterial hypertension and letal outcome despite specific treatment. Idiopatic pulmonary arterial hypertension diagnosis was performed at 3 months of age in a preterm female. Her twin sister died at 2 months of age due to refractory to inhaled nitric oxide (NO) pulmonary arterial hypertension, associated to partial anomalous pulmonary vein drainage and right aortic arch. On admisión she was on heart faillure and pro BNP 1790 pg/mL. When stabilized, cardiac catheterization was performed: mean pulmonary artery pressure (mPAP) 47 mmHg, wedge pressure 11 mmHg, pulmonary vascular resistance (PVR) 12.63 U/m^2^, RP:RS 0.75; acute vasoreactivity test NO 40 ppm + O_2_ 40%: mPAP 37 mmHg, PVR 6.56 U/m^2^, RP:RS 0.67. Genetic study was performed through exome molecular analysis clinically oriented. Results: Genetic test depicted exome 2p 13.3 NFU1 heterocigote c.622G>T alteration through aminoacid substitution (p. Gly208Cys) pulmonary arterial hypertension specific treatment encluded phosphodiesterase‐5 inhibitors, endothelin receptor antagonist and parenteral treprostinil. Her follow‐up encluded recovery with intravenous treprostinil and poor response when switched to subcutaneous pump. After 3 months in intensive care unit, patient died from multiorgan faillure. This genetic variant has being described as pathogenic since affects protein synthesis (PMID:314161310) and cause multiple mithocondrial dysfunction syndrome. Althoug rare, this genetic abnormality has being described in central nervous system disorders and pulmonary hipertensión. Pulmonary arterial hypertension in an infant due to exome 2p 13.3 NFU1 alteration may preclude poor outcome.

## A073 THE IMPACT OF PULMONARY ARTERIAL HYPERTENSION ON MATERNAL HEALTH AFTER SUCCESSFUL PREGNANCY—CLINICAL COURSE AND OUTCOMES

Shine Kumar^1^, Raman Krishna Kumar^2^

^1^Pulmonary Hypertension Clinic, Dept Of Pediatric Cardiology, Amrita Hospital, Amrita Vishwa Vidyapeetham University, Kochi, India, ^2^Department Of Pediatric Cardiology, Amrita Hospital, Amrita Vishwa Vidyapeetham University, Kochi, India

Pregnancy marks an important event in the life cycle of a woman. Pregnancy associated with pulmonary arterial hypertension (PAH) poses significant maternal mortality and morbidity. The maternal outcomes beyond peripartum period after successful pregnancy are scarcely reported in this subset. To describe the intermediate‐term maternal outcomes after successful pregnancy associated with PAH. We analyzed prospectively collected data of pregnancy associated with PAH from 2014 to 2022. We identified 18 successful pregnancies, predominantly primigravida (88.8%) with mean age of 28.3 ± 4.9 years and mean weight of 55.4 ± 11.9 kg. The diagnoses were Eisenmenger syndrome (8, 44.4%), residual PAH after correction of congenital heart disease (CHD) (6, 33.3%), idiopathic pulmonary arterial hypertension (2, 11.1%) and systemic lupus erythematosus (SLE) (2, 11.1%). The majority had severe PAH (15, 83.3%), while the rest had moderate PAH. All were in WHO functional class (FC) II (16, 88.8%) or I (2, 11.1%) pre pregnancy. At discharge after delivery majority were in WHO FC II (13, 72.2%), four (22.2%) in FC III and one (5.6%) in FC I with median Tricuspid Annular Plane Systolic Excursion (TAPSE) of 20.3 mm (range 13.6–33) and median N terminal pro Brain Natriuretic Peptide (Ntpro‐BNP) level of 199 picograms (pg)/ml (27–1522), upper limit of normal being 177 pg/mL. All patients except one (residual PAH after CHD correction) regained the pre‐pregnancy FC within the postpartum period of 6 weeks. After median follow‐up for 48.5 months (9–92), two patients (11.1%) expired, and the remaining were in FC II (15, 93.7%) and I (1, 6.3%) [mean 6‐min walk distance—394.3 ± 66.7 m), Ntpro‐BNP level—median 198.5 (25–1962 pg/mL), TAPSE—median 20.5 (15–28 mm)]. Among the survivors, nine patients (56.2%) were on monotherapy and seven (43.7%) on dual therapy with pulmonary vasodilators. They had five clinical worsening events (events requiring in hospital management), which included acute respiratory tract infections, right heart failure requiring up titration of pulmonary vasodilators and flaring up of SLE. The two expired patients had severe residual PAH after correction of CHD, one expired 81 months postpartum from disease progression and the other 15 months postpartum. The latter succumbed from progressive worsening after delivery, never attaining the pre‐pregnancy FC. The intermediate‐term follow‐up after successful pregnancy in well‐preserved selected women with PAH tends to follow the natural course of primary illness. Majority tolerated the pregnancy well and regained the pre‐pregnancy FC within the postpartum period. Those with severe residual PAH after correction of CHD are likely a more vulnerable high‐risk subset for pregnancy as reflected in accordance with their natural history.

## A074 BETA ADRENERGIC THERAPY IN GROUP 3 PULMONARY HYPERTENSION, FRIEND OR FOE?

Wang Wei^1^, Scott Collum^2^, Gautam Sikka^2^, Harry Karmouty Quintana^2^

^1^Wuhan University, Wuhan, China, ^2^Uthealth Houston, Houston, USA

Group 3 pulmonary hypertension (PH) is observed in patients with chronic lung diseases such as chronic obstructive pulmonary disease or idiopathic pulmonary fibrosis. PH develops as a result of extensive pulmonary vascular remodelling and resultant changes in vascular tone that can lead to right ventricle hypertrophy. This eventually leads to right heart failure, which is the leading indicator of mortality in patients with idiopathic pulmonary fibrosis. Treatments for group 3 PH are not available. Intriguingly, alternative polyadenylation (APA) is a process that has been recently associated with the development of PH and lung fibrosis. APA can result in transcripts with shortened or lengthened 3'untranslated regions (UTR). Alterations to the 3'UTR landscape can have important effects on the stability and translation efficiency of mRNA. Studies in cancer, PH and lung fibrosis have demonstrated that 3'UTR shortening results in increased cell proliferation and matrix deposition, important processes in vascular remodelling. β‐adrenergic blockers are one of the most widely prescribed drug classes worldwide, specifically for left‐side heart failure. However, their use in right‐side heart failure has been contra‐indicated. Yet a recent clinical trial in patients with PAH has demonstrated that the dual β1/β2 adrenergic receptor (AR) antagonist, carvedilol, was safe and improved cardiovascular outcomes. In this experiment we tested the capacity of carvedilol and clenbuterol (a selective β2AR‐agonist) in a model of bleomycin (BLM)‐induced lung fibrosis and pulmonary hypertension (PH). Our result show that structural alterations in the right heart, such as right ventricular wall hypertrophy, occurred as early as day 14 of BLM and that similar increases in right ventricle chamber size were seen between days 21 and 28. These structural changes were correlated with decreases in the systolic function of the right ventricle and right ventricular cardiac output, which also occurred between the same time points. Remarkably, our results revealed that these carvedilol and clenbuterol improved right‐heart function without worsening lung function. Specifically, both agents reduced right ventricle systolic pressure and altered heart function and structure measures by echo. Intriguingly, pulmonary artery smooth muscle cells (PASMC) treated with either carvedilol or clenbuterol exhibited a distinct 3'UTR landscapes. These changes in the 3'UTR profile may reflect previously unknown mechanisms for beta adrenergic function.

Taken together, these results demonstrate that despite the anti‐fibrotic effect of β2AR‐agonists, β‐blockers do not worsen lung fibrosis. It is also noteworthy that drugs with opposing pharmacology were both able to improve right‐heart function.

## A075 STUDY DESIGN OF A REAL‐WORLD PATIENT REGISTRY IN GROUP 3 PULMONARY HYPERTENSION DUE TO INTERSTITIAL LUNG DISEASE (PH‐ILD)

Sarah Kaspari^1^, Eric Shen^1^, Meredith Broderick^1^, Kevin Maher^1^

^1^United Therapeutics Corporation, Research Triangle Park, USA

Pulmonary hypertension (PH) is a common sequelae of chronic lung diseases such as interstitial lung disease (ILD). Regardless of disease severity, PH‐ILD negatively impacts symptoms and survival. PH‐ILD is also associated with increased rates of hospitalization and worse quality of life. Since limited data exist regarding disease background, risk factors, and disease progression, real‐world data is necessary to understand the epidemiological landscape and outcomes associated with PH‐ILD. This real‐world patient registry in Group 3 PH‐ILD is a prospective, non‐interventional, multicenter study that will enroll approximately 1000 patients across the United States. To enroll, patients must have a diagnosis of PH confirmed by right heart catheterization (RHC) and a diagnosis of ILD by computed tomography (CT). Patients with connective tissue disease must have a baseline forced vital capacity of <70%. Once enrolled, patient information will be retrospectively collected and recorded into a chosen electronic data capture system. Retrospectively collected information will include diagnostic values and imaging as well as other assessments of interest as available. Assessments collected at 6‐month intervals include spirometry (including diffusing capacity of the lungs for carbon monoxide), concomitant medication use (including supplemental oxygen requirements), WHO/NYHA functional class, transplant status, vital signs, healthcare resource utilization, echocardiogram, serum NT‐proBNP, patient reported outcomes questionnaires, 6‐min walk test and Borg dyspnea score. High‐resolution CT will be collected every 12 months and RHC will be collected as available from standard of care visits. All treatment decisions are to be made at the discretion of the patient's healthcare provider and will not be influenced by study participation. Patients who enroll will be followed for up to 5 years. Following study completion, demographic and epidemiological characteristics of patients with PH‐ILD will be analyzed and characterized. This registry aims to observe and capture demographic characteristics, treatment patterns, and clinical outcomes of interest for patients with PH‐ILD to further clinicians' understanding of the epidemiological landscape and outcomes of the disease.

## A076 EXPRESSION OF BMP LIGANDS IN BMPR2‐ AND TGFB1‐SILENCED HUMAN PULMONARY MICROVASCULAR ENDOTHELIAL CELLS

Nikolaos S. Lotsios^1^, Chrysi Keskinidou^1^, Alice G. Vassiliou^1^, Ioanna Dimopoulou^1^, Anastasia Kotanidou^1^, David Langleben^2^, Stylianos E. Orfanos^1^

^1^Department of Critical Care Medicine and Pulmonary Services, School of Medicine, National & Kapodistrian University of Athens, “Evangelismos” Hospital, Athens, Greece, ^2^Center for Pulmonary Vascular Disease, Division of Cardiology, Azrieli Heart Center, and Lady Davis Institute for Medical Research, Jewish General Hospital, McGill University, Montreal, Canada

Pulmonary arterial hypertension (PAH) is a chronic disease characterised by a progressive increase in mean pulmonary arterial pressure (1). Bone morphogenetic proteins (BMPs) are a group of signalling molecules, which belong to the transforming growth factor‐β (TGF‐β) superfamily of proteins (2). Bone morphogenetic protein type II receptor (BMPR2) is highly expressed in endothelial cells and is directly implicated in PAH progression. Different BMP ligands, including BMP9 and BMP10, bind with high affinity to the receptor complex, resulting in the activation of the downstream SMAD (Mothers Against Decapentaplegic Homolog) transcription factors (3). A switch between BMPR2‐SMAD1/5/8 to TGF‐β‐SMAD2/3 signalling appears to occur in PAH onset or development (4). Moreover, administration of BMP9 has shown to reverse established PAH, suggesting the promise of direct enhancement of endothelial BMP signalling as a new therapeutic strategy for PAH (5). Even though, mutations in the gene encoding BMPR2 are the most common cause of familial PAH in humans, only a small subset of individuals carrying BMPR2 mutations develops PAH. Thus, suggesting different genetic drivers of BMPR2 function could promote PAH onset and progression (6). Taking into account the involvement of the above‐mentioned molecules in PAH onset or development, we examined how BMP9 and BMP10 participate in the pathophysiology of PAH and investigated their possible interaction with other members of the TGF‐β superfamily, specifically TGF‐β1. To this end, we silenced the gene expression of BMPR2 and TGFB1 in separate experiments, in the HPMEC‐ST1.6R cell line. We then proceeded to examine mRNA and protein expression levels of both BMP9 and BMP10 in comparison to siRNA negative control (siRNA ‐ve). BMP9 mRNA expression levels presented a significant increase in BMPR2‐ and TGFB1‐silenced cells (*p* < 0.05). However, only in the BMPR2‐silenced cells did the protein levels of BMP9 increase (*p* < 0.05). Regarding BMP10, mRNA expression levels were found elevated in both conditions (*p* < 0.05), in contrast to protein levels, which remained unaltered. Our findings seem to support the potential of BMP9 as a therapeutic agent in PAH, since in our BMPR2‐silenced PAH‐mimicking model, both mRNA and protein levels of BMP9 and BMP10 increased, suggesting an endogenous attempt to counterbalance this effect. TGFB1‐silencing seemed to have the same effect.

## A077 UNRAVELING THE ENIGMA: FACTORS UNDERLYING DETERIORATION IN A PATIENT WITH PULMONARY ARTERIAL HYPERTENSION

Burcak Kilickiran Avci^1^, Utku Raimoglu, Damla Raimoglu, Cem Sulu, Sukran Nur Sanli, Ezgi Deniz Gokce

^1^Istanbul University Cerrahpasa, Cerrahpasa Faculty of Medicine, Department of Cardiology, Istanbul, Turkey

Pulmonary Arterial Hypertension poses a challenging clinical landscape. This case report delves into the complex diagnostic and management challenges encountered in a 44‐year‐old female patient who has been under our center's care for over a decade with a confirmed diagnosis of idiopathic pulmonary arterial hypertension (IPAH). In 2012, following her initial diagnosis, the patient initiated treatment with dual therapy comprising ambrisentan and tadalafil. This therapeutic regimen initially provided some relief; however, as her condition evolved, adjustments were made to her treatment plan. Iloprost was incorporated into her therapy, and in 2020, selexipag was introduced. Nevertheless, her clinical course was not without its challenges. Atrial flutter (AF) emerged as a complicating factor, necessitating the initiation of amiodarone therapy. Unfortunately, this course of treatment was short‐lived due to the recurrence of AF and the onset of hypothyroidism, leading to the discontinuation of amiodarone treatment. Subsequently, she underwent successfull AF ablation therapy. In early 2022, deteriorating risk parameters necessitated a transition to intravenous (IV) epoprostenol therapy (maximum dose 48 ng/kg/min). Remarkably, her condition improved, resulting in a shift from WHO functional class III to II. Additionally, there was a notable reduction in proBNP levels (from 4605 to 1105 pg/mL) and a significant improvement in the 6‐min walking distance (from 400 to 580 m). These positive developments enabled her to return to work. However, the patient's journey took an unexpected turn merely a year later. She was readmitted to hospital with symptoms including muscle pain, nausea, diarrhea, loss of appetite, and intermittent hypotension. The epoprostenol dose was gradually reduced in pursuit of a potential dose‐related etiology. While diarhea abated, the other symptoms persisted. Further evaluation unearthed low thyroid‐stimulating hormone (TSH) levels, which prompted the initiation of thyromazol therapy. Despite these interventions, her clinical status continued to deteriorate, manifesting as profound cachexia, further functional impairment, and worsening right heart failure with hypotension (72/38 mmHg). The possibility of an advanced right heart failure and hypoperfusion scenario loomed large. She was hospitalized, dopamine and dobutamine infusions were introduced. During this challenging period, renal function remained stable, urine output adequate, and consciousness unaltered. Further examination revealed a rare complication—an isolated adrenocorticotropic hormone (ACTH) deficiency. Hydrocortisone therapy was promptly initiated, leading to a rapid resolution of nausea, the return of appetite, the disappearance of weakness, and an increase in blood pressure increase to 92/50 mmHg. Over a 3‐month period she gained 8 kg, and she now resides in functional class II under the combined treatment of epoprostenol, ambrisentan, tadalafil and hydrocotisone. This case emphasizes the importance of considering adrenal insufficiency as a potential complication in patients receiving epoprostenol, as its clinical presentation often mimics drug‐related side effects and worsening right heart failure. Heightened awareness of this rare side effect can facilitate early diagnosis and timely intervention, ultimately improving patient outcomes.

## A078 IMPROVED ECHOCARDIOGRAPHIC MEASURES OF LEFT VENTRICULAR DIASTOLIC FUNCTION AFTER 6 MONTHS OF A MEDICALLY SUPERVISED KETOGENIC DIET

Darlene Kim^1^, NP Dthia Kalkwarf, Maria Saucedo Garcia, Nurse Practitioner Vera Pillitteri, Mohammad Dalabih, Zulma Yunt, Kristen Holm, M. Patricia George

^1^National Jewish Health, Denver, USA

Pulmonary hypertension associated with left heart disease (PH‐LHD) is the most common form of PH yet has few treatment options [1]. A growing subgroup of PH‐LHD is heart failure with preserved ejection fraction (HFpEF) associated with obesity and metabolic syndrome (MetS). We previously presented our work demonstrating meaningful improvements in obesity, quality of life, exercise tolerance, and metabolic parameters in response to a 6‐month medically supervised ketogenic diet (MSKD). In this update, we present echocardiographic evidence of improved diastolic function in this cohort. We prospectively enrolled subjects with PH‐LHD due to MetS‐associated HFpEF with mean pulmonary artery pressure >25 mmHg and mean pulmonary capillary wedge pressure >15 mmHg. Baseline testing included echocardiogram, bloodwork, exercise and quality of life assessments. Subjects were enrolled in a MSKD intervention for 6 months and were monitored with daily serum β‐hydroxybutyrate ketone and glucose levels. Regular safety checks were performed through bloodwork, physical exams, and daily symptom surveys. Repeat testing included echocardiogram at 6 months. Of the 11 subjects who completed the intervention, 9 completed the echocardiogram at 6 months. Similar to our previous report, subjects demonstrated improvements in a wide range of parameters, including weight (mean weight loss of 10.4 kg), abdominal circumference (mean decrease 9.5 cm), quality of life as measured by the Minnesota Living with Heart Failure Questionnaire, and exercise parameters. After 6 months, there was a mean increase in both septal and lateral e’ (0.39, 1.29 cm/s), an increase in E/A (0.22), a decrease in septal, lateral, and averaged E/e’ (−1.12, −1.90, −1.51), and a decrease in maximal tricuspid regurgitation velocity (−0.06 m/s). There was an increase in average NT‐proBNP from baseline to 6 months of 130 pg/mL, which may be a result of significant weight reduction and a correction in the “natriuretic handicap” seen in obesity, as has been reported in obese patients after gastric bypass and gastric sleeve surgeries, and after weight loss due to comprehensive lifestyle changes [2,3,4]. A MSKD in subjects with PH‐LHD due to MetS and HFpEF improved echocardiographic measures of left ventricular diastolic function. A concomitant increase in NT‐proBNP may be due to significant weight loss.

## A079 MACHINE LEARNING TO DETECT PULMONARY HYPERTENSION AND PULMONARY HEMODYNAMICS USING X‐RAY IMAGES

Tarik Kivrak^1^, Mehmet Ali Gelen, Hilal Erken, Turker Tuncer, Sengul Dogan

^1^Firat Üniversitesi, Elazig, Türkiye, ^2^Etlik State Hospital, Ankara, Turkey, ^3^Firat University Digital Forensics, Elazig, Turkey

Cardiac disorders contribute to significant mortality rates, emphasizing the importance of their early detection. Machine learning models, especially those with deep feature extraction capabilities, have been instrumental in diagnosing these disorders. This study uses X‐ray images with an advanced feature extraction model to detect cardiac tension. We collected seven datasets for this research, all pertaining to cardiac tension. We utilized these datasets to demonstrate the general classification ability of our proposed model, which is based on MobileNetV2. Our model, named ExMobileNet, integrates an exemplar feature extraction strategy. ExMobileNet comprises four main phases: (i) Features are generated from two layers of the pretrained MobileNetV2: the global average pooling (GAP) and the fully connected (FC) layers. Using the exemplar feature extraction strategy features from the raw X‐ray image and fixed‐size patches are extracted. Furthermore, a third feature vector is obtained by merging the vectors generated by the FC and GAP layers. (ii) The three generated feature vectors are used as input for the neighborhood component analysis (NCA) and Chi2 feature selectors. This phase culminates in the creation of six selected feature vectors. (iii) k‐nearest neighbor (kNN) and support vector machine (SVM) classifiers are applied to the generated feature vectors, yielding 12 distinct results (3 feature vectors × 2 selectors × 2 classifiers). The aforementioned 12 results serve as input for iterative majority voting (IMV). IMV produces ten voted results. The highest classification accuracy outcome is chosen from the collective 22 results (12 from classification + 10 from voting). At this juncture, ExMobileNet operates as a self‐organized model. Our proposed model achieved classification accuracies exceeding 90% across all seven datasets. Additionally, we analyzed the classification performance of the used models and identified the optimal one. The results underscore the efficacy of the proposed ExMobileNet, establishing it as a potent image classification model for detecting tension in X‐ray images.

## A080 CELL‐SPECIFIC KNOCKOUT OF TRANSCRIPTION FACTOR RBPJ PARTIALLY PROTECTS MICE FROM CHRONIC‐HYPOXIA‐INDUCED PULMONARY HYPERTENSION

Vanessa Kleinhenn^1^, Oleg Pak^1^, Stefan Hadžić^1^, Akylbek Sydykov^1^, MSc Anis Čilić^1^, Cheng‐Yu Wu^1^, Natascha Sommer^1^, Norbert Weissmann^1^, Tilman Borggrefe^2^, Christine Veith^1^

^1^Universities of Giessen and Marburg Lung Center (UGMLC), Member of the German Center for Lung Research (DZL), Excellence Cluster Cardio‐Pulmonary Institute (CPI), Justus‐Liebig University, Giessen, Germany, ^2^Institute of Biochemistry, Justus‐Liebig University, Giessen, Germany

Pulmonary hypertension (PH) is a life‐threatening disease characterised by an elevated pulmonary arterial pressure due to pulmonary vascular remodelling. If left untreated, PH progresses to right heart failure and eventually death. Pulmonary vascular remodelling can be caused by chronic alveolar hypoxia triggering an uncontrolled proliferation of pulmonary arterial smooth muscle cells (PASMC) and dysfunction of pulmonary endothelial cells (EC). Despite the already characterised involvement of Notch receptors in PH pathogenesis, the involvement of downstream signalling is poorly understood. In this regard, we aim to assess the role of recombination signal‐sequence‐binding protein J (RBPJ; also known as CSL or CBF1), the central transcription factor in the Notch pathway, in vascular cell dysregulation and PH pathogenesis. Loss‐of‐function experiments were performed in vitro to unravel the functional role of RBPJ in vascular cell dysregulation in PH. The in vivo relevance of RBPJ for disease development was assessed by using tamoxifen‐inducible STOCK RbpJ tm1Hon Tg(Acta2‐cre/ERT2)12Pcn (smooth‐muscle specific) and STOCK RbpJ tm1Hon Tg(Tek‐cre/ERT2)1Soff (endothelial cell‐specific) mice in a model of chronic hypoxia‐induced PH. Our data revealed a hypoxia‐dependent regulation of RBPJ in primary human vascular cells. Furthermore, the hypoxia‐induced increase in PASMC proliferation was reversed to baseline conditions following RBPJ silencing. In vivo data in inducible cell‐specific Rbpj‐knockout mice presume a role of RBPJ in chronic hypoxia‐induced PH, by affecting right ventricular systolic pressure, right heart hypertrophy/function, and pulmonary vascular remodelling. In conclusion, our data provide the first evidence for a role of RBPJ.

## A081 INFLUENCE OF CARDIOMETABOLIC COMORBIDITIES ON THE EFFICACY AND SAFETY PROFILE OF SOTATERCEPT IN PATIENTS WITH PAH: A POST HOC ANALYSIS OF THE STELLAR STUDY

Grzegorz Kopeć^1^, Marius Hoeper^2^, David Badesch^3^, H. Ardeschir Ghofrani^4^, J. Simon Gibbs^5^, Mardi Gomberg‐Maitland^6^, Vallerie McLaughlin^7^, Ioana Preston^8^, Rogerio Souza^9^, Aaron Waxman^10^, Ekkehard Grünig^11^, Gisela Meyer^12^, Karen Olsson^2^, Irene Aschenbach^13^, Aiwen Xing^14^, Amy Johnson‐Levonas^14^, Madhavi Reddy^14^, Ryan Frieler^14^, Michela Brambatti^14^, Stephan Rosenkranz^15^

^1^The Pulmonary Circulation Center, Department of Cardiac and Vascular Diseases, Jagiellonian University Medical College, John Paul II Hospital in Krakow, Krakow, Poland, ^2^Hannover Medical School and the German Center for Lung Research, Hannover, Germany, ^3^University of Colorado, Anshutz Medical Campus, Aurora, USA, ^4^Department of Internal Medicine, Justus‐Liebig‐University Giessen, Universities of Giessen and Marburg Lung Center (UGMLC), Member of the German Center for Lung Research (DZL), Giessen, Germany, ^5^National Heart and Lung Institute, Imperial College London, London, UK, ^6^George Washington University, Washington, USA, ^7^University of Michigan, Ann Arbor, USA, ^8^Tufts Medical Center, Boston, USA, ^9^Instituto do Coracao, Hospital das Clinicas da Faculdade de Medicina da Universidade de Sao Paulo, Sao Paulo, Brazil, ^10^Brigham and Women's Hospital, Boston, USA, ^11^Thoraxklinik‐Heidelberg and the German Center for Lung Research, Heidelberg, Germany, ^12^Irmandade da Santa Casa de Misericordia de Porto Alegre, Porto Alegre, Brazil, ^13^Universite Paris‐Saclay, INSERM Unite Mixte de Recherche en Sante 999, Hopital Bicetre (Assistance Publique‐Hopitaux de Paris), Le Kremlin‐Bicetre, France, ^14^Merck & Co., Inc., Rahway, USA, ^15^Department of Cardiology, and Cologne Cardiovascular Research Center (CCRC), Heart Center, University Hospital Cologne, Cologne, Germany

In patients with pulmonary arterial hypertension (PAH), the presence of cardiometabolic comorbidities may worsen disease progression and complicate management. Sotatercept is an activin signaling inhibitor under investigation for PAH. This post hoc analysis compared the efficacy and safety of sotatercept versus placebo across subgroups defined by baseline comorbidity type (yes/no: coronary heart disease [CHD], diabetes, hypertension, obesity) or number (0, 1–2, ≥3). Methods: Participants in STELLAR, a phase 3 trial (NCT04576988), were randomized 1:1 to sotatercept (0.3 up to 0.7 mg/kg once every 3 weeks) or placebo added to background PAH therapies. Eligible participants had a diagnosis of PAH, World Health Organization functional class (WHO‐FC) II‐III, pulmonary vascular resistance (PVR) ≥ 400 dyn·s·cm^−5^, and 6‐min walk distance (6MWD) 150–550 m. Participants were categorized by baseline medical history using MeDRA preferred terms as well as body mass index ≥30 kg/m² for the obesity subgroup. Participants also were classified by number of comorbidities present at baseline. Continuous endpoints were analyzed by aligned‐rank stratified Wilcoxon with WHO‐FC and background therapy as strata. Hodges‐Lehmann (HL) location‐shift estimates for between‐group difference in change from baseline (95% CI), p‐value were calculated. Categorial endpoints were analyzed by Cochran‐Mantel‐Haenszel. Time to death or non‐fatal clinical worsening event was analyzed by log‐rank, stratified by randomization factors. Hazard ratios (HR) were derived from a Cox proportional model with treatment group as covariate, stratified by randomization factors. Cumulative safety up to data cut‐off was descriptively summarized. Analyses were not adjusted for multiplicity. Analysis of the ≥3 comorbidity subgroup was hampered due to the small number of participants (*n* = 5 placebo, *n* = 7 sotatercept). Treatment effects were generally consistent across comorbidity type (i.e., yes/no: CHD [*n* = 24 and 299, respectively], diabetes [*n* = 24 and 299, respectively], hypertension [*n* = 64 and 259, respectively], obesity [*n* = 75 and 248, respectively]) and number (i.e., 0 [*n* = 192] and 1–2 [*n* = 119]). At week 24, between‐group HL shift estimates for changes from baseline in 6MWD (range: 37.0 to 71.7 meters [*p* ≤ 0.021 for all]), PVR (range: −220.5 to −346.4 dyn·s·cm^−5^ or 2.8 to 4.4 Wood Units [*p* ≤ 0.013 for all]), and NT‐proBNP (range: −273.3 to −725.2 pg/mL [*p* ≤ 0.036 for all]) favored sotatercept treatment across subgroups. The proportions of participants in each of the subgroups who showed improvement in WHO‐FC or ESC/ERS risk at week 24 were significantly higher with sotatercept versus placebo (*p* ≤ 0.036; WHO‐FC improvement p = NS for the obesity and no comorbidity subgroups). Prolonged event‐free survival was seen with sotatercept versus placebo in participants without comorbidities (HR 0.110; *p* < 0.001) and those with 1–2 comorbidities (HR 0.214; *p* = 0.029). The incidences of adverse events (AEs), treatment‐related AEs, and serious AEs with sotatercept versus placebo across the subgroups were comparable to that observed in the overall trial population.

The efficacy and safety profile of sotatercept was generally consistent across the comorbidity subgroups. These findings underscore the potential therapeutic benefits of sotatercept in a population of patients with PAH, including those with multiple or different cardiometabolic comorbidities.

## A082 DELETION OF NOXO1 AFFECTS COPD‐ASSOCIATED PH, CHRONIC HYPOXIA‐INDUCED PH AND PRESSURE OVERLOAD‐INDUCED RIGHT VENTRICULAR HYPERTROPHY IN A DIFFERENT WAY

Simone Kraut^1^, Akylbek Sydykov^1^, Fenja Knoepp^1^, Kathrin Malkmus^1^, Stefan Hadzic^1^, Cheng‐Yu Wu^1^, Lisa Schaffelhoffer^1^, Jo Meister^1^, Hossain A. Ghofrani^1^, Ralf P. Brandes^2^, Norbert Weissmann^1^, Christine Veith^1^

^1^Excellence Cluster Cardio‐Pulmonary Institute (CPI), University of Giessen and Marburg Lung Center (UGMLC), Member of the German Center for Lung Research (DZL), Justus‐Liebig‐University, Giessen, Germany, ^2^Institute for Cardiovascular Physiology, Goethe University, Frankfurt, Germany

Pulmonary hypertension (PH) is a life‐threatening disease that affects a wide range of people worldwide. It is characterized by pulmonary vascular remodeling, resulting in right ventricular hypertrophy and ultimately right heart failure. Recently, we identified the non‐phagocytic NADPH oxidase organizer 1 (Noxo1) as crucial in the development of cigarette smoke‐induced PH in mice. However, the role of Noxo1 in chronic hypoxia‐induced PH or pressure overload‐induced right ventricular hypertrophy has not been investigated yet. Thus, we subjected congenital Noxo1−/− and corresponding wild‐type (WT) mice to either chronic hypoxia (10% O₂) for 28 days or to pulmonary arterial banding‐induced chronic pressure overload for 21 days. Afterwards, mice were analyzed by echocardiographic, hemodynamic and histological measurements. Although Noxo1−/− mice are protected against cigarette smoke‐induced PH, both the degree of PH as well as right ventricular remodeling and function was unchanged between banded Noxo1−/− and WT mice. Interestingly, chronic hypoxia‐exposed Noxo1−/− mice were partially protected against PH, as indicated by a diminished right ventricular systolic pressure and wall thickness, whereas right ventricular function and lung vessel muscularisation was not altered between the two genotypes. Our data suggests, that Noxo1 plays a different role in COPD‐associated and chronic hypoxia‐induced PH in mice and is not involved in the control of adverse remodeling in the biomechanical stressed right ventricle.

## A083 HOMEODOMAIN‐INTERACTING PROTEIN KINASE 2 SUPPORTS INCREASED PROLIFERATION AND SURVIVAL OF HUMAN PULMONARY ARTERIAL SMOOTH MUSCLE CELLS IN PULMONARY ARTERIAL HYPERTENSION

Lifeng Jiang^1^, Dmitry Goncharov^1^, Yuanjun Shen^1^, Leyla Teos^1^, Derek Lin^1^, Aisha Saiyed^1^, Iryna Zhyvylo^1^, Theodore Avolio^2^, Elena A Goncharova^1^, Tatiana Kudryashova^2^

^1^University of California Davis, Davis, USA, ^2^University of Pittsburgh, Pittsburgh, USA

Pulmonary arterial hypertension (PAH) is a progressive deadly disease with no cure. Increased proliferation and survival of pulmonary arterial smooth muscle cells (PASMC), coupled with metabolic reprograming are key components of pulmonary vascular remodeling, a major pathophysiological feature of PAH, the mechanisms of which are not fully understood. We recently demonstrated that transcriptional coregulator homeodomain‐interacting protein kinase‐2 (HIPK2) is upregulated and supports pro‐proliferative/pro‐survival phenotype of PAH PASMC, and might serve as a potential target for therapeutic intervention to stop or reverse pulmonary vascular remodeling in PAH. However, the mechanisms of HIPK2 regulation and function in PAH remain understudied. Pharmacological inhibition of HIPK2 (tBID) in human PAH PASMC significantly decreased growth, proliferation (Ki67), induced apoptosis (TUNEL), and significantly decreased protein levels of pro‐proliferative/pro‐survival transcriptional co‐activator YAP/TAZ and regulator of cytokinesis centrosomal protein 55 (CEP55). RNAseq analysis of human PAH PASMC treated with tBID revealed significant down‐ and upregulation of 279 and 189 genes correspondently. KEGG enrichment analysis revealed deregulation of multiple genes associated with cell cycle, apoptosis, senescence, FoxO and p53 signaling pathways and glutathione metabolism. GO enrichment analysis revealed significant deregulation in genes associated with chromosome segregation, nuclear division, mitotic spindle and regulation of cell cycle transition. Immunocytochemical/immunoblot analyses demonstrated over‐accumulation of YAP and CEP55 in human PAH PASMC, and its depletion inhibited pro‐proliferative/pro‐survival protein kinase AKT, reduced cell proliferation (Ki67), and induced apoptosis (TUNEL), suggesting that HIPK2 promotes increased proliferation and survival of PAH PASMC through YAP and CEP55. Importantly, tBID‐induced YAP cytoplasmic retention in PAH PASMC and pharmacological (verteporfin) or siRNA‐induced YAP downregulation decreased HIPK2/CEP55 axis in PAH PASMC, suggesting that bi‐directional YAP‐HIPK2 crosstalk exists to support HIPK2/YAP/CEP55 upregulation and hyperproliferation in PAH. Human PAH PASMC demonstrate upregulation of key lipogenic enzymes (ACLY, ACC) as well as unrestricted growth and maintenance of intracellular lipid content (detected by BODIPY) in lipid‐free media, which were suppressed by AKT inhibition. Notably, inhibition of HIPK2 or siRNA‐induced depletion of CEP55 downregulated ACLY and ACC in human PAH PASMC, suggesting that HIPK2 supports increased lipogenesis and could act as a coordinator of cell cycle and cell metabolism in PAH PASMC. Immunoblot analysis revealed that HIPK2 downregulation by tBID significantly decreased the overproduction of major extracellular proteins Fibronectin and Collagen 1A in PAH PASMC. Moreover, control PASMC plated on the decellularized matrices, produced by PAH PASMC, had significantly increased levels of HIPK2, CEP55, YAP/TAZ, and elevated cell growth compared to cells grown on the matrices produced by control PASMC, suggesting that HIPK2 signaling is regulated by extracellular matrix composition. Importantly, pharmacological targeting of HIPK2 by tBID attenuated SU5416/Hypoxia‐induced PH in mice as evidenced by significant decrease of systolic right ventricular pressure (sRVP) compared to the vehicle‐treated group. HIPK2 supports pro‐proliferative/pro‐survival PASMC phenotype in PAH via up‐ regulating YAP/TAZ, CEP55, lipogenesis and extracellular matrix production. HIPK2 pharmacological inhibition attenuates pulmonary modeling and experimental PH in vivo. Further studies are needed to thoroughly evaluate benefits of HIPK2 targeting as a novel anti‐remodeling therapy for PAH. Funded by NIH/NHLBI R01HL166932 (TVK).

## A084 SEX‐DEPENDENT CHANGES TO THE RV MYOCYTE UNDERLIE SEX DIFFERENCES IN RIGHT VENTRICULAR CHAMBER ELASTANCE IN A RAT MODEL OF PULMONARY ARTERIAL HYPERTENSION

Ethan Kwan^1^, Tsui‐Min Wang^1^, Hao Mu^1^, Kristen Garcia^1^, Becky Hardie^1^, Daniela Valdez‐Jasso^1^

^1^University Of California San Diego, La Jolla, USA

Right ventricular (RV) function is an important prognostic indicator in pulmonary arterial hypertension (PAH), a progressive vasculopathy which stimulates RV hypertrophy and chamber remodeling and often results in intractable heart failure. While PAH predominantly develops in women, pre‐menopausal women show improved outcomes compared to post‐menopausal women or men1. We investigated sex differences in systolic and diastolic chamber function in a rat model of PAH by combining RV hemodynamic and morphologic measurements with a mathematical model of RV biomechanics. Age‐matched male and female (ovary‐intact and ovariectomized) rats were treated with vascular endothelial growth factor inhibitor and subjected to chronic hypoxia to induce PAH, following established methods (Sugen‐hypoxia/SuHx). RV blood pressure‐volume (PV) measurements, myocardial mechanics, and morphology were related using a RV biomechanics model2. Model analysis distinguished the relative contributions of chamber volume, geometry, and myocardial material properties. Myocyte shortening and calcium transients were measured to validate model predictions of myofiber contractility recruitment. SuHx‐treatment resulted in similar pulmonary arterial pressures, end‐systolic RV pressures, RV hypertrophy, and ejection fraction in all groups, with increases to end‐systolic chamber elastance being explained by RV hypertrophic wall thickening. However, SuHx‐induced remodeling of myocardium mechanical properties varied by sex and ovarian‐hormone presence. In SuHx male rats, RV end‐diastolic volume was maintained by passive myocardial stiffening. The smallest reductions in ejection fraction were seen in SuHx female rats, which showed only small increases in stiffening and no changes in myocyte shortening or calcium transients. SuHx‐treatment increased RV chamber volumes in ovariectomized female rats with increases in sarcomere shortening and peak intracellular calcium. This study demonstrates myocardial adaptation to PAH varies depending on sex and ovarian‐hormone presence and proposes mechanisms that would explain sex‐dependent outcomes. Diastolic chamber PV remodeling in male rats was explained by myocardial passive stiffening, matching previous clinical reports3. Model predictions of active material properties were validated with measurements of RV myocyte contractility and intracellular calcium, while diastolic stiffening was matched by biaxial testing of excised myocardium. Planned experiments aim to identify the cellular mechanism underlying calcium transient remodeling and to investigate passive myocyte stiffness. While the incidence of PAH is influenced by sex, several cohort studies have reported similar pulmonary vascular hemodynamics between men and women, which aligns with our findings. Previous studies have demonstrated that the relationship between sex and hemodynamics is not solely influenced by menopause, but also confounded by age and age‐related comorbidities. Further research is necessary to clarify the complex relationship between sex, age, and RV performance in pulmonary hypertension.

## A085 NON FLOW‐DRIVEN PHARMACOLOGICALLY‐MEDIATED PULMONARY CAPILLARY RECRUITMENT: A NOVEL MECHANISM FOR EXPANSION OF THE PERFUSED CAPILLARY BED

David Langleben^1^, ORFANOS STYLIANOS^2^, Ali Abualsaud^1^, Carlos Guerrero^1^, Judith Therrien^1^, Michele Giovinazzo^1^, Benjamin D Fox^3^, Nadira Ramrup^1^, Magali Kaddis^1^, Catherine Lagace^1^, Angie Spiropoulos^1^, John D Catravas^4^

^1^Jewish General Hospital, McGill University, Montreal, Canada, ^2^Evangelismos Hospital, University of Athens, Athens, Greece, ^3^Yitzchak Shamir Hospital, Tel Aviv University, Tzrifin, Israel, ^4^Old Dominion University, Norfolk, USA

Each normal human lung has a capillary surface area of a tennis court. At rest, the normal pulmonary microcirculation has many non‐concomitantly perfused capillaries. When pulmonary blood flow rises, such as during exercise, it is accommodated by expansion in the number of perfused capillaries, in a process known as recruitment. This results in an increase of measurable functional capillary surface area (FCSA). The degree of resting precapillary vascular constrictor tone, and the effects of a pulmonary vasodilator on capillary flow distribution in normals, are unknown. Patients born with a single functional cardiac ventricle must undergo corrective surgery that isolates deoxygenated blood flow into the lungs, from oxygenated blood feeding the body. The standard procedure is a Fontan correction, which connects systemic venous blood return directly into the pulmonary arterial system, without a right ventricle. This system functions as long as central venous pressure is adequate, pulmonary vascular resistance (PVR) is low, and pulmonary venous pressure is low. Lung blood flow in Fontan patients is continuous and nonpulsatile. The shear stress of pulsatile flow is known to stimulate production of endogenous pulmonary vasodilators, such as nitric oxide (NO). We hypothesized that there might be an element of vascular tone from NO deficiency, that would be detectable in nonpulsatile‐flow Fontan patients. We explored the contribution of PVR and the capillary flow distribution in 8 Fontan patients pre and during the administration of the pulmonary‐specific vasodilator, inhaled NO (40 ppm, 15 min). FCSA was assessed by measuring the first‐pass instantaneous percent metabolism (%M) and hydrolysis (v) of the radiolabelled substrate ³H‐benzoyl‐Phe‐Ala‐Pro, which interacts with the pulmonary capillary angiotensin‐converting‐1 ectoenzyme as the blood passes through the capillaries. Paired t‐test was used to compare baseline and NO. Results: PVR was <1.5 Wood units in all subjects at baseline, but decreased significantly by 20% or more in 6/8 subjects after NO. Mean PAP fell slightly but significantly. There was no significant change in cardiac output or PAWP. Total pulmonary gradient (TPG) also fell slightly but significantly. FCSA/BSA increased by at least 10% in 6/8 subjects, and the higher the baseline FCSA/BSA, the greater the % increase with NO. 4/8 subjects had large, unexpected, increases in v. Conclusion: Inhaled NO reduced PVR and PAP in Fontan patients, but without an increase in pulmonary blood flow. The clinical significance of these changes is unknown. However, FCSA also increased, and v increased in 4 patients, with no or little change in flow. This phenomenon, never previously described, is consistent with non flow‐mediated capillary recruitment, likely from a vasorelaxant effect of inhaled NO on contractile elements (smooth muscle and pericytes) in the lung microcirculation. FCSA rises because of an increased enzyme mass available for substrate interaction, and the rise in v indicates a longer capillary transit time for the blood, as the total lung blood flow distributes more widely. Capillary recruitment can occur physiologically (increased blood flow) or pharmacologically (vasodilator). Drs. Langleben and Orfanos contributed equally to this work and are both considered as presenting authors.

## A086 HYPOXIA ACTIVATES CASPASE‐1 IN PULMONARY EPITHELIUM, INDUCING CASPASE‐1‐DEPENDENT PULMONARY INFLAMMATION IN THE DEVELOPMENT OF PULMONARY HYPERTENSION

Camilla Udjus^1^, Ole Henning Skjønsberg^1^, Karl‐Otto Larsen^1^

^1^Oslo University Hospital, Oslo, Norway

Alveolar hypoxia (AH) occurring in lung diseases, may lead to pulmonary hypertension (PHT). Activation of caspase‐1, inducing inflammation, seems to influence the pathogenesis of PHT, mediating pro‐inflammatory cytokines like interleukin (IL)‐18. Elevated levels of IL‐18 are documented in the lungs after AH. Whether AH induces inflammation in other organs, is not known. Objectives: To explore whether lungs are the main site of caspase‐1‐related inflammation in AH. Methods: Wild type (WT) mice were exposed to AH or room‐air, and lungs and other organs were harvested. Right ventricular (RV) catheterization was performed in mice transplanted with WT or caspase‐1−/− bone marrow after chronic hypoxia. AH induced leukocyte infiltration and caspase‐1 activation in the lungs, while no leukocyte infiltration or changes in caspase‐1 were present in the brain, heart, liver, kidney, spleen or small intestine. Development of PHT was not influenced by depletion of caspase‐1 in leukocytes, determined by RV catheterization in mice transplanted with WT or caspase‐1−/− bone marrow. Thus, resident pulmonary cells can be of importance, and positive caspase‐1 and IL‐18 immunostaining were detected in bronchial epithelium from bronchi to the respiratory bronchioles. Hypoxia induced IL‐18 secretion from bronchial epithelial cells, and IL‐18 stimulation generated IL‐6 mRNA in monocytes. AH induced phosphorylated STAT3 in lungs, not in other organs. AH induces caspase‐1 activation and leukocyte infiltration specific to the lungs, not in other organs. IL‐18 from bronchial epithelial cells might contribute to hypoxia‐induced inflammation, possibly by IL‐6‐STAT3 signaling, leading to PHT.

## A087 EXPLORING HYPUSINE SIGNALING AS A NOVEL THERAPEUTIC TARGET IN PULMONARY ARTERIAL HYPERTENSION

Sarah‐Eve Lemay^1^, Yann Grobs^1^, Charlotte Romanet^1^, Sandra Martineau^1^, Mabrouka Salem^1^, Tsukasa Shimauchi^1^, Sandra Breuils‐Bonnet^1^, Alice Bourgeois^1^, Charlie Théberge^1^, François Potus^1^, Steeve Provencher^1,2^, Sébastien Bonnet^1,2^, Olivier Boucherat^1,2^

^1^Pulmonary Hypertension Research Group, CRIUCPQ‐UL, Quebec, Canada, ^2^Departement of medicine, Laval University, Quebec, Canada

Pulmonary Arterial Hypertension (PAH) is characterized by progressive pulmonary arteries (PAs) obstruction leading to heart failure and death. PA smooth muscle cells (PASMCs) of PAH patients display a “cancer‐like” phenotype that contributes to PA remodeling. Appreciation of the pivotal role of translational control in hyperproliferating diseases is steadily increasing. In this regard, eukaryotic translation initiation factor 5A (eIF5A) was shown to provide cancer cells with a competitive advantage by increasing the translation of mRNAs with oncogenic proprieties. Strikingly, eIF5A is the only protein containing the unique spermidine‐derived amino acid hypusine required for its function. Hypusine formation is catalyzed by the sequential actions of two enzymes dedicated to this pathway, deoxyhypusine synthase (DHPS) and deoxyhypusine hydrolase (DOHH). We hypothesized that increased hypusinated eIF5A in PAH‐PASMCs is required to promote translational efficiency of a set of factors conferring a higher survival and fibroproliferative capacity, leading to pulmonary vascular remodeling. Data derived from a comparative proteomic analysis (LC‐MSMS) between normal and PAH‐PASMCs and confirmed by Western blot indicate that DHPS and DOHH are overexpressed in PAH‐PASMCs compared to controls. Consistently, both total and hypusinated forms of eIF5A were found upregulated in dissected PAs, isolated PASMCs and failing right ventricles from PAH patients and animal models (monocrotaline and Sugen/hypoxia rats). In vitro, pharmacological inhibition of DHPS and DOHH, using GC7 and ciclopirox, respectively, as well as molecular inhibition using an adenovirus encoding for a short hairpin RNA that targets DHPS significantly attenuates PAH‐PASMCs survival (Western blot Survivin; Immunofluorescence Annexin V and terminal deoxynucleotidyl transferase dUTP nick end (TUNEL) labeling) and proliferation (Western blot MCM2, PLK1; Immunofluorescence Ki67 labeling and EdU incorporation). Mechanistically, Hypusine signaling promoted the expression of a broad array of proteins involved in oxidative phosphorylation, supporting the bioenergetic requirements of cell survival and proliferation (LC‐MSMS, Western blot, Seahorse assay). Mitochondrial translation elongation factor Tu (TUFM), an essential factor for the enzymatic activities of mitochondrial respiratory complexes, was identified as a potential direct translational target of eIF5A (Western blot, qPCR and RNA immunoprecipitation). The decreased expression of TUFM upon DHPS inhibition may, in part, explain its effects on cell proliferation and survival. In human right ventricular fibroblasts, pharmacological inhibition of DHPS prevents TGFβ1‐induced activation (Western blot collagen 1 and 3, fibronectin and αSMA). In vivo, although biallelic inactivation of Dhps targeted to smooth muscle cells was incompatible with extrauterine life, we found that its haploinsufficiency was sufficient to confer partial protection against the development of Sugen/hypoxia (Su/Hx)‐induced PH in mice. Moreover, pharmacological inhibition of DHPS using GC7 in monocrotaline‐treated and Su/Hx‐exposed rats with established PAH improved hemodynamics (right ventricular systolic pressure, mean pulmonary arterial pressure and cardiac output), vascular remodeling (Elastica Van Gieson staining) and right ventricular fibrosis (Masson's trichrome staining). We showed for the first time that hypusine signaling is implicated in PAH development and represents a new promising therapeutic target to improve pulmonary vascular remodeling.

## A088 IDENTIFICATION OF AURKB AS A THERAPEUTIC TARGET TO COUNTER PULMONARY VASCULAR REMODELING IN PAH

Sarah‐Eve Lemay^1^, Mélanie Sauvaget^1^, Manon Mougin^1^, Reem El Kabbout^1^, Sandra Martineau^1^, Sandra Breuils‐Bonnet^1^, Alice Bourgeois^1^, Mabrouka Salem^1^, Charlie Théberge^1^, François Potus^1^, Steeve Provencher^1,2^, Sébastien Bonnet^1,2^, Olivier Boucherat^1,2^

^1^Pulmonary hypertension research group, CRIUCPQ‐UL, Quebec, Canada, ^2^Department of Medicine, Laval University, Quebec, Canada

Pulmonary arterial Hypertension (PAH) is characterized by vasoconstriction and remodeling of pulmonary arteries (PAs), causing right ventricular (RV) failure, and premature death. Extensive proliferation of PA smooth muscle cells (PASMCs) is perhaps the most prominent feature of PAH accounting for the histopathological changes seen in this disease. With the recognition of this, direct targeting of vascular remodeling by anti‐proliferative approaches provides a promising strategy which holds the potential to reverse the remodeling process. To discover novel actionable target implicated in vascular remodeling, we performed a comparative RNA‐sequencing analysis between control and PAH‐PASMCs. To extract reliable targets, we next enriched our own experiment with two publicly available datasets conducted on comparable cell lines. After merging the three datasets, 136 genes were found to overlap, of which 126 and 10 were up‐ and downregulated in PAH‐PASMCs, respectively. A connectivity map analysis using SigCom Library of Integrated Network‐based Cellular Signatures was performed on the commonly upregulated genes and identified GSK1070916, an ATP‐competitive inhibitor of AURKB/C as the top reverser drug of PAH‐PASMCs gene signature. In support of this, aurora kinase B (AURKB), known to play a critical role in the onset and progression of mitosis, was present among the overlapping upregulated genes in PAH‐PASMCs. Increased expression of AURKB was confirmed in isolated PASMCs from PAH patients and in dissected PAs from monocrotaline (MCT)‐treated and Sugen/Hypoxia (Su/Hx)‐exposed rats compared to controls (Western blot). Additionally, further experiments using loss‐ and gain‐of‐function approaches revealed that FOXM1 positively regulates AURKB. Pharmacological (Barasertib) and molecular (siAURKB) inhibition of AURKB reversed the PAH‐PASMCs gene signature (RNA sequencing) and significantly reduced PAH‐PASMC proliferation (EdU incorporation & Ki67 labeling, PLK1 expression) and survival (Annexin V labeling and Survivin expression). These effects were associated with mitotic defects, including chromosome misalignment and multipolar spindles, resulting in cell cycle arrest in G2/M phase and formation of multinucleated cells (flow cytometry and αTubulin labeling). Additionally, the acquisition of a senescence‐like phenotype was observed in PAH‐PASMCs that escape to apoptosis following AURKB treatment (beta‐galactosidase assay, RNA sequencing and p21, p53, lamin B1, IL1b, IL6, IL8, GDF15, MMP3 expression). In vivo, treatment with Barasertib significantly reduced pulmonary vascular remodeling, improved hemodynamics and decreased RV hypertrophy in monocrotaline and Su/Hx rats with established PH. Consistent with our in vitro study, a marked increase of p21 was observed in the distal PAs of Barasertib‐treated animals (Immunofluorescence). We demonstrated that upregulation of AURKB contributes to vascular remodeling in PAH and represents a new therapeutic target. Current experiments aim at evaluating whether the combination of Barasertib with different classes of senotherapeutic agents is more effective in improving PAH than either drug alone.

## A089 TARGETING THE FIBRONECTIN‐BINDING INTEGRINS SYSTEM: AN APPROACH TO COUNTERACT PULMONARY VASCULAR AND RIGHT VENTRICULAR MALADAPTIVE REMODELING IN PULMONARY ARTERIAL HYPERTENSION

Sarah‐Eve Lemay^1^, Mónica S Montesinos^2^, Yann Grobs^1^, Tetsuro Yokokawa^1^, Tsukasa Shimauchi^1^, Sandra Breuils‐Bonnet^1^, Sandra Martineau^1^, Mabrouka Salem^1^, Alice Bourgeois^1^, Charlotte Romanet^1^, Reem El Kabbout^1^, Mélanie Sauvaget^1^, Charlie Théberge^1^, Xinqiang Huang^2^, James E Dowling^2^, Min Lu^2^, Adrian S Ray^2^, François Potus^1^, Steeve Provencher^1,3^, Olivier Boucherat^1,3^, Sébastien Bonnet^1,3^

^1^Pulmonary hypertension research group, CRIUCPQ‐UL, Quebec, Canada, ^2^Morphic Therapeutic, Waltham, USA, ^3^Department of Medicine, Laval University, Quebec, Canada

Pulmonary arterial hypertension (PAH) is characterized by progressive obstruction and decreased compliance of pulmonary arteries (PA), leading to right ventricular (RV) failure and premature death. It is recognized that, like cancer cells, PA smooth muscle cells (PASMCs) exhibit exaggerated proliferation and resistance to apoptosis in response to increased PA stiffness caused by extracellular matrix (ECM) remodeling. Integrins, members of the cell adhesion receptors superfamily, are known to promote cell proliferation, survival, hypertrophic growth and fibrosis, which are key elements leading to the progression of PAH. Therefore, we hypothesized that integrins signaling could promote PAH‐PASMC proliferation and resistance to apoptosis contributing to PAs vascular remodeling, and RV maladaptive hypertrophy and fibrosis, leading to RV failure. Using NanoString, we found that members of the fibronectin‐binding integrins (FnBIs) family were the most abundantly expressed in PASMCs and RV fibroblasts (RVFbs) isolated from PAH patients. By western blot (WB), we showed that FnBIs expression is significantly changed in distal PAs, PASMCs, PA endothelial cells and decompensated RV from PAH patients compared to controls. Similarly, increased expression was found in compensated and decompensated RV isolated from monocrotaline (MCT) and pulmonary artery banding (PAB) rats. Pharmacological inhibition of FnBIs decreases PAH‐PASMCs proliferation (Western blot PLK1, MCM2 and Ki67 labeling) and resistance to apoptosis (Western blot Survivin and Annexin V labeling). These effects were associated with a decreased activation of FnBIs downstream signaling pathways FAK and ILK. Pharmacological inhibition of FnBIs downregulates genes involved in mitotic cell cycle (RNA sequencing) and seems to impair centrosome formation and separation in early mitosis (Immunofluorescence, α‐tubulin and pericentrin labeling). In adult rat cardiomyocytes, FnBIs inhibition decreased phenylephrine‐induced hypertrophy and reversed established hypertrophy induced by monocrotaline treatment (f‐actin labeling). In human RVFbs, FnBIs inhibition prevented TGFβ1‐induced activation of CTRL‐RVFbs and decreased proliferation and activation of PAH‐RVFbs (Western blot PCNA, αSMA, COL1; Immunofluorescence Ki67). In vivo, in both moncrotaline and Sugen/hypoxia rats with established PAH, pharmacological inhibition of FnBIs alone or in combination with macitentan and tadalafil improves hemodynamics (mean pulmonary arterial pressure, cardiac output (CO), tricuspid annular plane systolic excursion (TAPSE), RV fractional area change (RVFAC)), vascular remodeling (Elastica Van Gieson staining), RV hypertrophy (Hematoxylin‐Eosin staning) and RV fibrosis (Masson's trichrome staning). In the PAB rat model, inhibition of FnBIs attenuates RV dysfunction (CO, TAPSE, RV end‐diastolic pressure, RVFAC). Ex vivo, preliminary results suggest that pharmacological inhibition of FnBIs may improve vascular remodeling in precision‐cut lung slices isolated from PAH patients (Elastica Van Gieson). Pharmacological inhibition of FnBIs improves hemodynamics and vascular remodeling in two complementary animal models of PAH and attenuates RV dysfunction in the PA banding rat model. Our results suggest that integrins play a key role in pathological lung and RV remodeling in PAH.

## A090 ROLE OF THE MITOCHONDRIAL COMPLEX III PROTEIN UQCRH IN HYPOXIC PULMONARY VASOCONSTRICTION

Muchen Li^1^, Oleg Pak^1^, Julia Schaeffer^1^, Stefan Hadzic^1^, Fenja Knoepp^1^, Werner Seeger^2^, Ralph T Schermuly^1^, Norbert Weissmann^1^, Natascha Sommer^1^

^1^Excellence Cluster Cardio‐Pulmonary Institute, University of Giessen and Marburg Lung Center, Member of the German Center for Lung Research, Giessen, Germany, ^2^Excellence Cluster Cardio‐Pulmonary Institute, University of Giessen and Marburg Lung Center, Institute for Lung Health, Member of the German Center for Lung Research, Giessen, Germany

Hypoxic pulmonary vasoconstriction (HPV) is essential for redirecting blood from poorly to better ventilated areas of the lung during acute local hypoxia, thereby preventing systemic hypoxemia. Chronic hypoxia additionally leads to pulmonary vascular remodelling and pulmonary hypertension (PH). HPV is triggered by an increase in mitochondrial superoxide release from complex III, which is regulated by a specific subunit of complex IV (cytochrome c oxidase subunit 4 isoform 2, Cox4i2). Cox4i2 expression is increased in mice deficient for ubiquinol‐cytochrome c reductase hinge protein (Uqcrh), which is an integral protein of complex III. Therefore, we hypothesized that Uqcrh will be involved in acute oxygen sensing in the pulmonary vasculature triggering HPV and development of PH. To investigate the role of Uqcrh for acute oxygen sensing, HPV was quantified in isolated perfused and ventilated mouse lungs from mice with a global deletion of Uqcrh (Uqcrh‐/‐) and wild‐type (WT) mice. To test its effect on chronic hypoxic signalling, Hif1α expression and cellular proliferation were compared in mouse pulmonary arterial smooth muscle cells (mPASMCs) of WT and Uqcrh−/− mice after chronic hypoxic exposure (1%O_2_ for 24 h or 72 h, respectively). Additionally, Uqcrh mRNA and protein expression was determined in WT mPASMCs after exposure to chronic hypoxia (1%O_2_ for 24 h, 48 h, and 72 h) as well as in lung tissues from donors and patients with idiopathic pulmonary arterial hypertension (IPAH). HPV was completely inhibited in mouse lungs isolated of Uqcrh−/− mice, while the response to a hypoxia‐independent stimulus with the thromboxane analogue U46619 was preserved. Hif1α protein expression and proliferation were increased to similar levels in WT and Uqcrh−/− mPASMCs after chronic hypoxic exposure. Uqcrh mRNA and protein expression were not significantly regulated in WT mPASMCs after chronic hypoxic exposure or lung tissues from IPAH patients. Our results show that the presence of Uqcrh is essential for the acute, but not chronic response of pulmonary vasculature to hypoxia.

## A091 THE EFFECT OF HIGH ALTITUDE (2500M) ON INCREMENTAL CYCLING EXERCISE IN PATIENTS WITH PULMONARY ARTERIAL HYPERTENSION AND CHRONIC THROMBOEMBOLIC PULMONARY HYPERTENSION—A RANDOMIZED CONTROLLED CROSS‐OVER TRIAL

Julian Müller^1^, Mona Lichtblau^1^, Anna Titz^1^, Simon R Schneider^1^, Meret Bauer^1^, Laura Mayer^1^, Leo Lüönd^1^, Tanja Ulrich^1^, Michael Furian^1^, Aglaia Forrer^1^, Esther I Schwarz^1^, Konrad E Bloch, Silvia Ulrich^1^

^1^University Hospital Zürich, Zürich, Switzerland

Physical activity at high altitude, including hiking or skiing, is increasingly desirable also for stable patients with precapillary pulmonary hypertension (PH) due to the pulmonary vascular diseases (PVD) pulmonary arterial and chronic thromboembolic PH (PAH/CTEPH). We investigated the effect of high altitude on cycling exercise performance in patients with stable PVD. In an RCT, stable patients with PAH or distal CTEPH without hypoxemia at low altitude performed two incremental exercise tests to exhaustion, one in low‐ (470 m) and one in high altitude (2500 m). The main outcome was maximal work rate. In 27 patients with PVD (44% women, 61 ± 14 y), maximal work rate was 110 ± 64 watts versus 123 ± 64 watts at 2500 m versus 470 m (95%CI: −16 to −11, *p* < 0.001). SpO2 and PaO2 at the end of exercise were 83 ± 6% versus 91 ± 6% and 6.1 ± 1.9 kPa versus 8.6 ± 1.9 kPa (−8%; −29%; both *p* < 0.001) at 2500 m versus 470 m, respectively. Maximal oxygen uptake (V'O2) at HA was 17.8 ± 7.5 L/min/kg versus 20 ± 7.4 L/min/kg (−11%) at low altitude (*p* < 0.001). The ventilatory equivalent for CO2 (V'E/V'CO2) was significantly increased at end‐exercise at high altitude by 3.8 (95%CI: 1.5–6.0; *p* = 0.002). No adverse events occurred during or after exercise. In patients with stable PVD, cycling exercise at 2500 m was well tolerated, but exercise capacity was lower compared to 470 m, associated with lower blood oxygenation and decreased ventilatory efficiency.

## A092 THE PREVALENCE OF PULMONARY HYPERTENSION AFTER SUCCESSFUL TUBERCULOSIS TREATMENT IN A COMMUNITY SAMPLE OF ADULT PATIENTS

Elizabeth Louw^1^, Nicola Baines^1^, Gerald Maarman^1^, Muhammad Osman^1,2^, Lovemore Sigwadhi^1,3^, Elivs Irusen^1^, Coenraad Koegelenberg^1^, Anton Doubell^1^, Steven Nathan^4^, Richard Channick^5^, Brian Allwood^1^

^1^Stellenbosch University, Cape Town, South Africa, ^2^School of Human Sciences, University of Greenwich, London, UK, ^3^Biomedical Research and Training Institute, Zimbabwe, ^4^Inova Fairfax Hospital, Falls Church, USA, ^5^David Geffen School of Medicine, Los Angeles, USA

There are an estimated 155 million survivors of tuberculosis (TB). Clinical experience suggests that post‐pulmonary tuberculosis lung disease (PTLD) is an important cause of group 3 pulmonary hypertension (PH). However, TB is not listed as a cause of PH in most guidelines. A cross‐sectional, community‐based study was conducted in non‐health care‐seeking adults who had successfully completed TB treatment between 1 and 5 years prior. The aim of the study was to determine PH prevalence. Subjects underwent questionnaires, spirometry, a 6‐min walk distance test (6MWD), and transthoracic echocardiography (TTE). Screen probable PH was defined on TTE as an estimated pulmonary artery peak systolic pressure (PASP) of ≥40 mmHg. One hundred adults (71 males) were enrolled, with a mean age of 42 years (SD 13.8 years) and a median of one TB episode (IQR: 1–2). Seventy‐two were current smokers (72%) and 12 previous smokers (12%), with a combined median of 8 pack years (IQR 3–22). Co‐morbidities included hypertension (21%), diabetes (16%), HIV (10%), and asthma/COPD (5%). Only 25% had no residual symptoms after TB. Probable PH was found in 9%, while 7% had borderline raised PASP values (PASP 35–40 mmHg). An association was found between PH and the number of previous TB episodes, with each additional episode of TB increasing the odds of PH‐post‐TB 2.13‐fold (CI 1.17–3.88; *p* = 0.013). All of those found to have PH were smokers or ex‐smokers, yielding an unadjusted odds ratio for PH‐post‐TB of 3.67 (95%CI 0.77–17.46). There was no statistical difference in spirometry or 6MWD, between those with and without PH. Neither symptoms nor co‐morbidities demonstrated significant association with PH. Interpretation of PH after TB was a common finding in this community‐based population. Further research is needed to confirm and determine the significance of these findings.

## A093 DISCOVERY OF A NOVEL AND SELECTIVE ACTIVATOR OF ADENYLYL CYCLASE ISOFORM 6: A POTENTIAL TREATMENT FOR PULMONARY HYPERTENSION

Saeid Maghsoudi, Alexander Pritchard, Vikram Bhatia, Martha Hinton, Prashen Chelikani, John Sorensen, Shyamala Dakshinamurti

^1^Biology of Breathing Group, Children's Hospital Research Institute of Manitoba; Department of Physiology, University of Manitoba, Winnipeg, Canada, ^2^Department of Chemistry, University of Manitoba, Winnipeg, Canada, ^3^Biology of Breathing Group, Children's Hospital Research Institute of Manitoba, University of Manitoba, Winnipeg, Canada, ^4^Biology of Breathing Group, Children's Hospital Research Institute of Manitoba; Department of Physiology and Pathophysiology, University of Manitoba, Winnipeg, Canada, ^5^Department of Oral Biology, University of Manitoba, Winnipeg, Canada, ^6^Department of Chemistry, University of Manitoba, Winnipeg, Canada, ^7^Biology of Breathing Group, Children's Hospital Research Institute of Manitoba; Departments of Physiology and Pediatrics, University of Manitoba, Winnipeg, Canada

Persistent pulmonary hypertension of the newborn (PPHN) is a disorder of the normal pulmonary vascular relaxation after birth, featuring hypoxemia and pulmonary vasoconstriction. Failure of first‐line nitric oxide (NO) therapy can result in death. In addition to the NO‐guanylate cyclase pathway, receptors coupled to G‐alpha‐s (Gαs) and adenylyl cyclase (AC) mediate vasorelaxation and are crucial drug targets. AC isoforms 3, 6, 7, and 9 are expressed in neonatal pulmonary arteries (PA), but AC6 is predominant. AC6 is considered cardioprotective, and its content upregulated in hypoxia. However, we have reported AC enzyme inhibition due to S‐nitrosylation in PPHN PA, and in PA myocytes exposed to hypoxia. By mutational analysis of S‐nitrosylatable cysteines, we identified C1004, at the interface of AC6 with Gαs, as crucial to initiate AC6 activity. To rescue AC6 activity, we synthesized and analyzed the activity of a series of novel forskolin derivatives against AC isoforms 3, 5, 6, 7, and 9. HEK cells stably overexpressing AC isoforms (AC 3, 5, 6, 7, 9) as well as cysteine‐to‐alanine mutants (AC6_C1004A, AC6_C1145A or AC6_C447A) were cultured in normoxia (21% O_2_) or hypoxia (10% O_2_) for 72 h, or challenged with nitroso donor S‐nitrosocysteine (CysNO). Cells from all treatment groups were lysed for measurement of real‐time AC catalytic activity, and detection of protein S‐nitrosylation by Biotin Switch Method. Forskolin‐dependent real‐time cAMP generation was measured using a live‐cell cAMP biosensor cADDis assay. A library of forskolin derivatives was synthesized and screened against all AC isoforms. dose–response testing was performed for compounds showing AC6 selectivity. Among AC isoforms studied, only AC6 catalytic activity is inhibited by hypoxia and CysNO, impairing cAMP production; this correlates with increased cysteine nitrosylation of hypoxic AC6, but not of other AC isoforms. C1004 is conserved among AC isoforms. However, alanine substitution of C1004, but not of other exposed cysteines, prevents nitrosylation of hypoxic AC6; AC activity and cAMP accumulation are decreased in AC6_C1004A compared to AC6 WT. Among forskolin derivatives synthesized, forskolin 1α,9α‐carbonate offered relatively selective activation of AC6. We conclude that among AC isoforms, only AC6 is S‐nitrosylated in hypoxia and after NO exposure, uniquely inhibiting its activity and decreasing cAMP generation. This impairs efficacy of PPHN treatment. We propose that nitrosylation at C1004 may inhibit AC6 interaction with Gαs; and that selective AC6 reactivation may be a feasible therapeutic target. Novel forskolin derivatives designed to selectively target allosteric activation of AC6 may hold promise in treating hypoxic PPHN. The C1 forskolin carbonate derivative of forskolin may interact selectively with the AC6 FSK binding site.

## A094 OSTEOPONTIN IN EXPERIMENTAL RIGHT VENTRICULAR REMODELING

Argen Mamazhakypov^1^, Norbert Weissmann^1^, Ardeschir Hossein Ghofrani^1^, Ralph Schermuly^1^, Akylbek Sydykov^1^

^1^Cardio‐Pulmonary Institute, Justus‐liebig‐universität Gießen, Gießen, Germany

Right ventricular (RV) failure is a critical determinant of survival and prognosis in patients with pulmonary hypertension. Circulating levels of osteopontin (OPN) are increased in these patients and associated with RV remodeling and dysfunction. We hypothesized that OPN‐knockout (OPN‐KO) mice would be protected from adverse RV remodeling and the reconstitution of OPN using recombinant OPN (rOPN) would mitigate adverse RV phenotypes caused by OPN deficiency in a mouse model of RV failure. We performed sham or pulmonary artery banding (PAB) surgery on OPN‐KO and wild‐type (OPN‐WT) mice and administered rOPN for 3 weeks. Cardiac phenotypes were evaluated with echocardiography and catheterization. PAB surgery caused a significant increase in RV systolic pressure and RV remodeling and dysfunction in both OPN‐KO and OPN‐WT mice with no differences between them. Treatment with rOPN had no effect on the functional and structural parameters of the RV in either OPN‐KO or OPN‐WT mice. Interestingly, OPN‐KO mice exhibited larger right atrium (RA) dilatation compared to OPN‐WT mice, which was attenuated with rOPN treatment. Despite a similar degree of RV remodeling, PAB OPN‐KO mice displayed a slightly higher mortality than OPN‐WT mice, although this difference was not statistically significant. Treatment with rOPN slightly but not significantly improved the survival of these mice. The lack of OPN did not affect RV remodeling in mice subjected to PAB. However, OPN deficiency was associated with a larger RA dilatation and a higher mortality in PAB mice, which were attenuated with OPN reconstitution. These results suggest that rOPN may have therapeutic potential in treating RV failure.

## A095 THE ROLE OF THE TRANSCRIPTION FACTOR FOXO3 IN RIGHT VENTRICLE HYPERTROPHY AND REMODELING

Giovanni Maroli^1^, Sreenath Nayakanti^1^, Argen Mamazhakypov^2^, Golnaz Hesami^1^, Sandra Medrano^1^, Fatemeh Khassafi^1^, Baktybek Kojonazarov^2^, Werner Seeger^1,2^, Rajkumar Savai^1^, Soni Pullamsetti^1,2^

^1^Max Planck Institute For Heart And Lung Research, Bad Nauheim, Germany, ^2^Justus Liebig University, Giessen, Germany

The Forkhead box O (Foxo) transcription factor family plays important roles in metabolism and adaptation to stress in distinct tissues. However, its role in right ventricle (RV) hypertrophy and remodeling is unknown. Here we show that the Foxo family member Foxo3 is transcriptionally upregulated upon compensated RV hypertrophy in pulmonary hypertension (PH) patients. Foxo3 is dephosphorylated in RVs from pulmonary artery banding (PAB)‐treated mice, suggesting transcriptional activation upon RV remodeling. Fibroblast‐specific ablation of Foxo3 leads to aberrant RV remodeling with increased cardiomyocyte hypertrophy and worsened RV function upon PAB. Transcriptomic analysis showed that several genes involved in extracellular matrix composition, angiogenesis and cardiac remodeling are drastically upregulated in wild type RVs upon PAB, but remain at basal levels in PAB‐treated Foxo3 mutant RVs. In contrast, genes involved in innate immune response, antigen presentation and T cell biology are significantly upregulated in the RVs of sham‐treated Foxo3 mutant mice compared to wild type controls. Mechanistically, we found that Foxo3 ablation in fibroblasts results in the accumulation of antigen‐presenting macrophages and T cells in the myocardium, which in turn may perturb normal pressure overload‐induced RV remodeling. In conclusion, we describe a novel function for Foxo transcription factors in RV hypertrophy and remodeling, with important implications for PH‐induced cardiac disease.

## A096 ROLE OF CARDIOPULMONARY EXERCISE TESTING IN THE NON‐INVASIVE DIAGNOSIS OF MILD PULMONARY HYPERTENSION IN PATIENTS WITH EXERTIONAL SYMPTOMS AFTER A PULMONARY EMBOLISM AND PERSISTENCE OF THROMBOTIC MATERIAL IN THE PULMONARY ARTERIES

Irene Martin de Miguel^1^, Sergio Huertas Nieto, Dra. Teresa Segura de la Cal^1^, Dra. Carmen Jiménez López‐Guarch^1^, Dra. Maite Velázquez Martín^1^, Fernando Sarnago Cebada^1^, ALEJANDRO CRUZ UTRILLA^1^, Nicolás Maneiro Melón^1^, Claudio Manuel Rivadulla Varela^1^, Fernando Arribas Ynsaurriaga^1^, Dra. Pilar Escribano Subias^1^

^1^Hospital Universitario 12 de Octubre, Madrid, Spain

Exercise limitation after a pulmonary embolism (PE) is common and multifactorial; among its causes is the persistence of thrombotic material in the pulmonary arteries with secondary pulmonary vascular disease. However, in the absence of relevant resting pulmonary hypertension (PH), non‐invasive diagnosis is limited by the low sensitivity of non‐invasive techniques. Therefore, the aim of this study is to establish whether in patients with dyspnea and persistent thrombosis after an acute PE cardiopulmonary exercise testing (CPET) has diagnostic utility to identify patients with mild PH. Methods: Symptomatic patients with discordant perfusion defects in lung ventilation/perfusion scintigraphy and computed tomographic confirmation of pulmonary artery obstructions despite optimal anticoagulation for at least 3 months after an acute PE and baseline right heart catheterization (RHC) with mean pulmonary artery pressure (mPAP) < 25 mmHg, pulmonary vascular resistance (PVR) ≤ 3 Wood units (Wu), pulmonary arterial wedge pressure (PAWP) < 15 mmHg undergoing CPET and exercise RHC were included. Comparison of CPET parameters between patients with baseline RHC with mild PH (mPAP 21–25 mmHg and PVR 2–3 Wu according to the new criteria from the European Society of Cardiology Guidelines) and patients with normal baseline hemodynamics (mPAP ≤ 20 mmHg, PVR ≤ 2 Wu) was performed. Exercise PH was defined as a mPAP/cardiac output slope >3 mmHg/L/min. Thiry‐nine patients (52.6 ± 14.6 years; 33.3% women) were included. Nine (23.1%) had mild resting PH. Compared with patients with normal baseline hemodynamics, patients with mild resting PH presented a lower percentage of the theoretical peak oxygen consumption and peak oxygen pulse (65 ± 17.1 vs 79.8 ± 13.4%, *p* = 0.04 and 75.9 ± 11.7 vs 91 ± 14.9%, *p* = 0.006, respectively), a lower percentage of the theoretical oxygen uptake efficiency slope (71.5 [17.8; 76.8] vs 81 [72; 97] %, *p* = 0.03); as well as greater ventilatory inefficiency (lower end‐tidal CO_2_ partial pressure at the second ventilatory threshold [32.2 ± 2.9 vs 35 ± 3.8 mmHg, *p* = 0.03] and a lower oxygen saturation at peak effort [90 ± 4.0 vs 93.4 ± 2.8%, *p* = 0.049]). Eighteen patients (46.2%) developed exercise PH. Patients with mild resting PH more frequently developed exercise PH than those with normal baseline hemodynamics (77.8% vs 36.7%, *p* = 0.05). Patients with persistent exertional dyspnea and thrombosis after an acute PE with mild resting PH have a lower functional capacity and greater ventilatory inefficiency in CPET. In this regard, CPET could represent a useful tool for the noninvasive diagnostic stratification of this subgroup of patients. Larger studies are warranted to further investigate these metrics and to establish additional noninvasive diagnostic and prognostic tools that ultimately lead to the development of optimal diagnostic and treatment protocols for symptomatic patients with chronic thromboembolism, mild pulmonary vascular disease without significant resting PH and impaired right ventricular and pulmonary vascular reserve during exercise.

## A097 PROGNOSTIC IMPLICATIONS OF ELEVATED LEFT ATRIAL VOLUME INDEX IN PATIENTS WITH HEART FAILURE WITH REDUCED EJECTION FRACTION AND PULMONARY HYPERTENSION

Mamotabo Matshela^1^

^1^SACIDF, Durban, South Africa

Left atrial volume index (LAVI) is a known marker indicative of the severity of raised left atrial pressure associated with left ventricular (LV) diastolic dysfunction. However, the prognostic implications of LAVI in pulmonary hypertension (PH) secondary to heart failure with reduced ejection fraction (HFrEF) has not been fully evaluated. To evaluate the prognostic implications of LAVI in HFrEF and PH. A total of 151 consecutive patients who had transthoracic echocardiography, documented HFrEF and PH were evaluated/included. There were 66 documented cardiac events during a median follow‐up 1.5 ± 0.6 years. There were no significant differences in LV end‐diastolic dimensions or ejection fraction between patients who did or did not have cardiac events. The LAVI values were significantly higher in patients with documented events (58 ± 23 vs 42 ± 18 mL/m^2^, *p* < 0.001). The study demonstrated an increase in risk of cardiac events with each unit increment of LAVI category, and LAVI strongly correlated with the highest risk of cardiac events. Multivariate Cox proportional hazard analysis demonstrated significantly higher LAVI as an independent predictor for cardiac events compared with other standard echocardiographic parameters including diastology and LV strain (*p* < 0.001). The LAVI proved to be a useful prognostic parameter in patients with documented HFrEF and PH, might be a useful echocardiographic parameter for risk stratification of patients with HFrEF and PH.

## A098 PROGNOSTIC IMPLICATIONS OF RIGHT VENTRICULAR SYSTOLIC AND DIASTOLIC STRAIN IN HEART FAILURE WITH REDUCED EJECTION FRACTION AND PULMONARY HYPERTENSION

Mamotabo Matshela^1^

^1^SACIDF, Durban, South Africa

The 2D‐ speckle‐tracking echocardiography (2D‐STE) is regarded as a modern echocardiographic technique recently used to assess subclinical myocardial mechanical dysfunction. However, its values in heart failure with reduced ejection fraction (HFrEF) and pulmonary hypertension has not been specifically focusing on the right ventricular (RV) longitudinal systolic function but the left ventricle. Main Aim: To evaluated RV strain parameters and it's associated with clinical outcome in patients with HFrEF and PH. Of the initial 121 HFrEF and PH patients were screened for this study and were eligible, then retrospectively enrolled. The 2D‐STE strain parameters were assessed on the 2D‐TTE images focusing on the RV mechanics. The patients were prospectively followed for development of new cardiac events which included hospitalization for acute HF and CVS‐related death. The RV longitudinal strain parameters (RVLS, RVLD) were assessed using the 2D‐STE and averaged in all segments (apical 4CV, global RVLS and RVLD) and also averaged free‐wall, as appropriate. Of the 121 patients at baseline, 64 had 43 new events during a mean follow‐up of 1.3 ± 0.6 years. RV free wall and global RV systolic, elevated NT Pro‐BNP, RV fractional area change, low TAPSE, LV end‐diastolic volume and PASP > 40 mmHg were independently predictive of combined outcomes (*p* <0.001). Overall, the best performance parameters predictive of cardiovascular events were for RV Free Wall systolic (AUC: 0.86), global RV systolic (AUC: 0.82) and RV fractional area change (AUC: 0.72). The strongest association between the degree of RV dysfunction and the risk of cardiovascular events was only evident for RV strain. Patients with HFrEF and PH, RV strain parameters are stronger predictors of outcome than other parameters and should be considered for regular clinical assessment and prognostic stratification.

## A099 25‐YEAR EXPERIENCE WITH PREGNANCY IN PULMONARY ARTERIAL HYPERTENSION IN A MULTICULTURAL AND RESOURCE‐LIMITED ENVIRONMENT

Tanya McWilliams^1^, Marie Mata^1^, Sasi Sithamparanathan^1^

^1^Auckland City Hospital, Auckland, New Zealand

Previous guidelines recommended against pregnancy in women with pulmonary arterial hypertension (PAH) due to high maternal mortality. This has been a topic for debate in recent literature. The Hannover group published their midterm outcomes from pregnancies in a cohort of patients with PAH advocating individualized advice to those who wish to become pregnant. The 2022 ERS guidelines support this approach. New Zealand has limited funding for PAH‐specific therapy. Auckland is the largest city with a multicultural population including Indigenous Māori and Pacifica people. The PAH service has managed planned pregnancies, for women presenting with PAH in pregnancy and post‐partum. At Auckland City Hospital (ACH), the multidisciplinary PAH, obstetric, and maternal fetal medicine team (MFM) have managed eighteen pregnancies in 16 women over 25 years in a multicultural and resource‐limited environment with excellent outcomes. Methods: Retrospective case review with data on age, ethnicity, classification, and if this was a planned pregnancy, PAH specific therapy used, delivery and outcome were retrieved. Results: Median age was 32.5 (23–39) years with one Asian, two Maori, three Pacifica, four Indian, and six European women. All but one had Nice Group 1 PAH (4 IPAH, 4 APAH‐CHD, 3 APAH‐CTD, 2 drugs associated, and one each PVOD and PoPH). Two pregnancies were unplanned, both had severe PAH and the women were counselled to terminate the pregnancy with no adverse outcomes. There were five planned pregnancies: two women had two planned pregnancies with 4 live births one further woman had severe PAH and despite advice not become pregnant had one live birth. Seven women presented with PAH during pregnancy two in the second trimester who elected to continue pregnancy. All the babies were delivered by planned caesarean section. Three women presented critically unwell immediately post‐partum. One needed urgent lung transplantation. There was only one death of both mother and baby. This mother presented with severe PAH due to metastatic malignant obstruction of the pulmonary circulation at 30 weeks pregnant (Nice 4.2.2) the baby delivered spontaneously and died 1 day post‐delivery. Two of the women had no antenatal care before presenting with PAH in pregnancy. Sildenafil and iloprost were used in pregnancy with epoprostenol once funded. All the other babies survived with no complications. ACH has a unique experience over many years where late or post‐partum presentation with PAH is not unusual. This experience has enabled us to support women who choose to become pregnant fully informed of the associated risks. The positive outcomes from our experience demonstrate successful pregnancies in PAH are feasible with careful counselling of the patient and MDT approach.

## A100 PROGNOSTIC PERFORMANCE OF RIGHT VENTRICULAR EVALUATION BY NON‐EXPERT PHYSICIANS USING POCKET ULTRASOUND IN PATIENTS WITH PULMONARY HYPERTENSION

Pamela Mercado Velázquez^1^, José Luis Hernández Oropeza^1^, Ivette Buendía Roldan^2^, Tatiana Sofia Rodríguez Reyna^1^, Consuelo Orihuela Sandoval^1^, Adrián Soto Mota^1^

^1^Salvador Zubirán National Institute of Health Sciences and Nutrition (INCMNSZ), Mexico City, Mexico, ^2^National Institute of Respiratory Diseases (INER), Mexico City, Mexico

Transthoracic Echocardiography (TTE) plays an important role in the clinical follow‐up of patients with PH (Pulmonary Hypertension)1. However, TTE is frequently inaccessible during follow‐up consultations and can only be performed by Echograpy‐specialised cardiologists. While many echocardiographic variables have been associated with mortality and survival2,3, Tricuspid Annular Plane Systolic Excursion (TAPSE) stands out because of its strong correlation with the right ventricular ejection fraction and its high reproducibility between observers3. To compare the predictive accuracy for disease progression of TAPSE measurements obtained with a pocket ultrasound (POCUS) device by trained non‐expert physicians with those acquired with echocardiography and with other well‐established disease progression predictors such as pericardial effusion and dilated right atrium. We prospectively enrolled 63 consecutive patients with PH groups 1 and 4. All patients underwent two‐dimensional (2D) echocardiography, cardiac ultrasound, physical examination, and 6MWT, at baseline and after 3 and 6 months. All echocardiographic measurements were analyzed by an expert echocardiographist and POCUS measurements were made by both observers. All researchers were blinded to the clinical characteristics of patients. The predictive performance for disease progression (defined as the occurrence of any of the following: worsening of symptoms of right heart failure, decrease in the number of meters walked during the 6MWT, need for hospitalizations, initiation of intravenous therapy or inhaled prostacyclin, need for additional therapy, septostomy or death), of each measurement‐observer pair, was compared with the AIC, BIC, Adjusted R2, and RMSE of their respective univariate logistic regression models. We included 63 participants, 83% (*n* = 52) female, with a mean age of 52 ± 13 years. Seventy‐one percent of our participants had group 1 PH (Pulmonary Arterial Hypertension), of these 44% were associated with connective tissue diseases, 12% were secondary to portal pulmonary syndrome, and 8% were idiopathic. Twenty‐eight percent (*n* = 18) of subjects were classified as group 4. At baseline, the interobserver agreement of having a TAPSE < 17 mm and pericardial effusion were 0.78 and 0.75, respectively. All TAPSE measurement‐observer pairs demonstrated comparable predictive performance for disease progression. Moreover, altered TAPSE (< 17 mm) by a nonexpert physician demonstrated a comparable predictive performance as finding pericardial effusion or a dilated right atrium with echocardiography.

These results suggest that POCUS TAPSE measurements made by trained nonexpert physicians can be a useful clinical follow‐up tool as they are accessible, reliable, and have comparable disease progression predictive accuracy as well‐established echocardiographic measurements.

## A101 INFLUENCE OF BACKGROUND PAH THERAPY ON THE EFFICACY AND SAFETY OF SOTATERCEPT IN PATIENTS WITH PAH: A POST HOC ANALYSIS OF THE STELLAR STUDY

Gisela Meyer^1^, Marius Hoeper^2^, David Badesch^3^, H. Ardeschir Ghofrani^4^, J. Simon Gibbs^5^, Vallerie McLaughlin^6^, Ioana Preston^7^, Rogerio Souza^8^, Aaron Waxman^9^, Ekkehard Grünig^10^, Grzegorz Kopeć^11^, Karen Olsson^2^, Stephan Rosenkranz^12^, Marc Humbert^13^, Jianxin Lin^14^, Amy Johnson‐Levonas^14^, Madhavi Reddy^14^, Ryan Frieler^14^, Janethe de Oliveira Pena^14^, Mardi Gomberg‐Maitland^15^

^1^Irmandade De Santa Casa De Misercordia De Porto Alegre, Porto Alegre, Brazil, ^2^Hannover Medical school and the German Center for Lung Research, Hannover, Germany, ^3^University of Colorado, Anschutz Medical Campus, Aurora, USA, ^4^Department of Internal Medicine, Justus‐leibig‐University Giessen Universities of Giessen and Marburg Lung Center (UGMLC), Member of the German Center for Lung Research (DZL), Giessen, Germany, ^5^National Heart and Lung Institute, Imperial College London, London, UK, ^6^University of Michigan, Ann Arbor, USA, ^7^Tufts Medical Center, Boston, USA, ^8^Instituto do Coracao, Hospital das Clinicas da Faculdade de Medicina da Universidade de Sao Paulo, Sao Paulo, Brazil, ^9^Brigham and Women's Hospital, Boston, USA, ^10^Thoraxklinik‐Heidelberg and the German Center for Lung Research, Heidelberg, Germany, ^11^The Pulmonary Circulation Center, Department of Cardiac and Vascular Diseases, Jagiellonian University Medical College, John Paul II Hospital in Krakow, Krakow, Poland, ^12^Department of Cardiology, and Cologne Cardiovascular Research Center (CCRC), Heart Center, University Hospital Cologne, Cologne, Germany, ^13^Universite Paris‐Saclay, INSERM Unite Mixte de Recherche en Sante 999, Hopital Bicetre (Assistance Publique‐Hospitaux de Paris), Le Kremlin‐Bicetre, France, ^14^Merck & Co., Inc., Rahway, USA, ^15^George Washington University, Washington, USA

Pulmonary arterial hypertension (PAH) remains a progressive debilitating disease despite existing therapies. The 2022 ESC/ERS guidelines recommend combination therapy with different agents to manage PAH. Sotatercept is an activin signaling inhibitor in clinical trials for PAH. This post hoc analysis evaluated the influence of background combination therapy (monotherapy, dual therapy, triple therapy) and prostacyclin infusion use (yes, no) on the efficacy and safety of sotatercept in patients with PAH. Participants in STELLAR, a phase 3 trial (NCT04576988), were randomized 1:1 to sotatercept (0.3 up to 0.7 mg/kg once every 3 weeks) or placebo added to background PAH therapies. Eligible participants had a diagnosis of PAH, World Health Organization functional class (WHO‐FC) II‐III, pulmonary vascular resistance (PVR) ≥ 400 dyn·s·cm^−5^, 6‐min walk distance (6MWD) 150–550 m. No new therapies could be added. Subgroups were defined by background therapy (monotherapy, dual, triple therapy) and prostacyclin infusion use (yes/no) at randomization. Continuous endpoints were analyzed by aligned‐rank stratified Wilcoxon with WHO‐FC and background therapy (prostacyclin subgroup, only) as strata. Hodges–Lehmann (HL) location‐shift estimates for between‐group difference in change from baseline (95% CI), *p*‐value was calculated. Categorial endpoints were analyzed by Cochran–Mantel–Haenszel. Time to death or nonfatal clinical worsening event was analyzed by log‐rank, stratified by randomization factors. Hazard ratios (HR) were derived from a Cox proportional‐hazards model with the treatment group as a covariate, stratified by randomization factors. Cumulative safety up to data cut‐off was descriptively summarized across subgroups. Analyses were not adjusted for multiplicity. In STELLAR, 112 (35%), 198 (61%), and 130 (40%) participants were on dual therapy, triple therapy, and prostacyclin infusion, respectively. The monotherapy subgroup was not analyzed due to small number of participants (*n* = 4 placebo, *n* = 9 sotatercept). Sotatercept treatment improved efficacy measures across dual, triple, and prostacyclin infusion use subgroups. The between‐group HL shift estimates for changes from baseline in 6MWD (range 38.4–43.3 m; *p* < 0.001), PVR (range −144.7 to −280.6 dyn·s·cm^−5^; *p* < 0.001) and NT‐proBNP (range −430.4 to −444.2 pg/mL; *p* < 0.001) were significant at week 24, favoring sotatercept across all subgroups. The proportions of participants who showed improvement in WHO‐FC or ERS/ESC risk status at week 24 were higher with sotatercept versus placebo, irrespective of background therapy (*p* ≤ 0.033 for all; except *p* = 0.411 for double therapy subgroup WHO‐FC). Prolonged event‐free survival was seen with sotatercept across all subgroups (HRs range: 0.151–0.209; *p* ≤ 0.008), especially pronounced in participants on triple background therapy. The overall incidences of adverse events (AEs), treatment‐related AEs, and serious AEs with sotatercept vs placebo across the various subgroups were comparable to that observed in the overall trial population. Sotatercept significantly improved exercise capacity, hemodynamics, functional parameters, risk score, and outcomes irrespective of dual or triple background therapy and parenteral prostacyclin. The overall safety profile of sotatercept versus placebo seen across the background therapy subgroups was comparable to the overall trial population in STELLAR. These findings underscore the potential therapeutic benefit of sotatercept when added to combination background PAH therapies including parenteral prostacyclin.

## A102 THE ROLE OF THE COMPLEMENT LECTIN PATHWAY IN THE PATHOGENESIS OF SCHISTOSOMA‐INDUCED PULMONARY HYPERTENSION

Claudia Mickael^1^, Linda Sanders^1^, Rahul Kumar^2^, Michael Lee^2^, Dara Fonseca‐Balladares^2^, Kurt Stenmark^3^, Rubin Tuder^1^, Brian B. Graham^2^

^1^University Of Colorado/Division of Pulmonary Sciences and Critical Care Medicine, Aurora, USA, ^2^University of California in San Francisco/Division of Pulmonary and Critical Care Medicine, Zuckerberg San Francisco General Hospital, San Francisco, USA, ^3^University of Colorado/Department of Pediatrics, Aurora, USA

Pulmonary hypertension (PH) is a pulmonary vascular disease characterized by perivascular inflammation resulting in vascular remodeling and increased mean pulmonary arterial pressures. This disease is fatal, and treatment options are limited. Complement activation has been recently reported as a significant contributor to PH pathogenesis; however, little is known about the role of complement in Schistosoma‐PH, a disease present in geographic regions where the parasite is endemic. Mannose‐binding lectin (MBL) is known to bind to parasite glycoproteins, activating the lectin complement pathway. In addition, Th2‐induced inflammation is a determinant in the pathogenesis of Schistosoma‐PH. Here, we tested the hypothesis that, as an innate trigger of adaptive immunity, complement activation through the lectin pathway is necessary for Type 2 inflammation, contributing to Schistosoma‐induced perivascular inflammation and PH. We used MBL−/− (mannose‐binding lectin) chimera mice, with bone marrow (BM) cells from MBL−/− donor mice transferred into lethally irradiated WT recipient mice. Reconstitution of WT BM into WT mice was used as controls. We intraperitoneally (IP) sensitized, and 2 weeks later, we intravenously (IV) challenged the mice with *Schistosoma mansoni* eggs. We also used the hypoxia sterile inflammation experimental model (as a non‐Th2 model), where the mice were challenged with 10% FiO2 hypoxia for 21 days. Endpoints included right ventricle systolic pressure (RVSP), right ventricle hypertrophy (RVH by Fulton Index), and analysis of the lung tissue. We observed that MBL−/− chimera mice had significantly lower RVSP and RVH compared to the WT chimeras (Fig. 1). Th2 inflammatory cytokines, IL4, and IL13 levels were mildly attenuated in the MBL−/− chimeras compared to the WT chimeras following Schistosoma‐challenge, which also presented a lower number of IL4 + CD4 + T cells. However, the peri‐egg granulomas had similar volume. In contrast, MBL−/− chimeras exposed to hypoxia showed increased RVSP and RVH. MBL−/− chimeras were protected from Schistosoma‐induced PH but not from hypoxic PH, suggesting that the pathogenic effect of the lectin pathway activation is specific to Schistosoma. The bone marrow compartment is the primary source of PH‐driving MBL.

## A103 GENETIC ASSOCIATIONS IN CHILDREN WITH PULMONARY ARTERIAL HYPERTENSION IN THE UK AND IRELAND

Cara Morgan^1^, Andrew Constantine, Shahin Moledina

^1^Great Ormond Street Hospital For Children, London, UK

Paediatric onset pulmonary arterial hypertension (PAH) is enriched with genetic associations. There is emerging evidence of genotype‐phenotype relations aiding diagnosis, tailored therapies, and predicting outcomes. We aim to describe through routine clinically collected data from the UK National Paediatric Pulmonary Hypertension Service (NPPHS) registry the prevalence of genetic factors, genotype‐phenotype associations, and outcomes in paediatric PAH. Retrospective cohort analysis of children with PAH classified as either idiopathic (both responder and nonresponder status), heritable, associated with co‐incident congenital heart disease, or pulmonary veno‐occlusive disease (PVOD) from inception (1 January 2001) to present day (1 October 2023). Genetic screening for PAH causative genes in this cohort of patients is routine in the NPPHS. However, due to the retrospective design of this study, there is variation in the genes tested for at the time of initiation of clinical screening. Children were additionally assessed by clinical geneticists in addition to relevant genetic testing initiated if there was a suspicion of a genetic disorder. Due to the novelty of recently discovered causative genes in PAH, particularly in children, those with a clear phenotype any variant of uncertain significance (VUS) were also included. Of the 187 children included in the study, 65 (35%) were identified to have a genetic disorder. Forty‐seven children (25%) had a known PAH gene mutation or variant, the most prevalent being TBX4 (*n* = 18, 10%) followed by BMPR2 (*n* = 14, 7%). Genetic syndromes (excluding TBX4 syndrome) were diagnosed in a further seventeen (9%) children with the majority having a documented association with PAH in the literature (Down's *n* = 6, Kabuki *n* = 2, CTNNB1 *n* = 1, incontinenti pigmenti *n* = 1). Of 117 children with no known genetic disorder, 10 (9%) were long‐term responders. Baseline characteristics showed that children with TBX syndrome were diagnosed younger compared to children with both other genetic associations and also children with no known genetic associations. Median duration of follow‐up from enrolment to the NPPHS was for the entire cohort was 3.2 [IQR: 0.7, 8.1] years. No children with sporadic PAH diagnosed as long‐term responders died or required either a Potts shunt or lung transplantation, termed composite outcome. Of children with a genetic disorder, those with TBX4 syndrome had the most favourable composite outcome. Children with PVOD had the worst composite outcome, followed by patients with SOX17 variant. This study demonstrates the wide range of genotype variation in paediatric PAH and highlights the important role of genotyping in predicting prognosis. The genotype‐phenotype of the patient should be considered in the management of children with PAH. Moreover, it is likely that future therapies may emerge to target gene‐specific PAH. However, the full extent of genotype‐phenotype associations in paediatric PAH is evolving, and further work is required in this field.

## A104 GENETIC ASSOCIATIONS IN CHILDREN WITH PULMONARY ARTERIAL HYPERTENSION: CLINICAL CHARACTERISATION AND OUTCOMES

Cara Morgan^1^, Andrew Constantine^2^, Shahin Moledina^1,3^

^1^Great Ormond Street Hospital For Children, London, UK, ^2^National Heart and Lung Institute, Imperial College, London, UK, ^3^Institute of Cardiovascular Sciences, University College London, London, UK

Paediatric‐onset pulmonary arterial hypertension (PAH) is rich with genetic associations. There is emerging evidence of genotype‐phenotype relations but scarce data on newly identified genes and genotype‐specific outcomes. Retrospective analysis of children with PAH diagnosed as either idiopathic (IPAH) or heritable (HPAH) disease, associated with small/coincidental congenital heart defects, pulmonary veno‐occlusive disease (PVOD), or pulmonary capillary haemangiomatosis (PCH) from 1 January 2001 to 1 October 2023. Patients underwent clinical genetic testing based on available testing panels and criteria. If there was a suspicion of a genetic disorder children were assessed by clinical geneticists, and relevant genetic testing was performed. Due to the novelty of recently discovered causative genes in PAH, particularly in children, those with a clear phenotype and variants of uncertain significance were also included. Survival analysis was performed using the Kaplan–Meier method with a composite outcome of death, lung transplant, or Potts shunt. Children were censored at the first occurrence of either transition to adult services, or emigration from UK. Of the 188 children included in the study, 68 (36%) were identified to have a genetic disorder. Fifty (27%) children had a known PAH gene mutation or variant, the most prevalent being TBX4 (*n* = 18, 10%), followed by BMPR2 (*n* = 16, 9%), ACVRL1 (*n* = 6, 3%), and SOX17 (*n* = 5, 3%). Genetic syndromes were diagnosed in a further 17 (9%) children, with the majority having a reported association with PAH (Trisomy = 6, Kabuki = 2, CTNNB1 = 1, Noonan's = 1, incontinenti pigmenti = 1). Ten of 58 children with HPAH (17%) had no known gene association. Of 115 children with sporadic PAH, 10 (9%) were long‐term responders. Seven (4%) children had a diagnosis of PVOD/PCH, two (33%) of whom had heterozygous variants in EIF2AK4. Children with TBX4‐syndrome were diagnosed younger compared to children with either sporadic PAH (2.6 ± 2.0 vs. 7.4 ± 4.9 years, *p* < 0.001) or those with BMPR2 mutation (2.6 ± 2.0 vs. 7.4 ± 4.6 years, *p* < 0.001) and had a higher prevalence of persistent pulmonary hypertension of the newborn (80% vs. 3% in all others, *p* < 0.001). Minor CHD lesions were prevalent across all groups (SOX17 (80%), other genetic association (61%), TBX4 (44%), ACVRL1 (33%), sporadic PAH (31%), BMPR2 (13%)). Those with SOX17 variant had the worst haemodynamic severity (mean mPAP 74 ± 25 mmHg, mean PVRi 33 ± 25 WU.m²). Of children with a genetic disorder, those with ACVRL1 had the most favourable composite outcome, 100% event‐free survival, followed by TBX4‐syndrome. Patients with SOX17 variant had the poorest outcome with BMPR2 and other genetic groups having intermediate outcome. Considering clinical phenotype, composite outcomes were worst in children with a PVOD/PCH phenotype, intermediate in sporadic PAH and best in long‐term responders, having 100% event‐free survival. This study demonstrates the wide range of genetic contribution to paediatric PAH. Clinical phenotype, disease severity, and outcome varied between genotypes. The full extent of genotype‐phenotype relations in paediatric PAH is evolving and further work is required to exploit these relations therapeutically.

## A105 DESIGNING AND UNDERSTANDING WHAT‐IF EXPLANATIONS IN AN INTERACTIVE CLINICAL DECISION‐SUPPORT TOOL FOR PULMONARY HYPERTENSION OUTCOME RISK ASSESSMENT AND TREATMENT GUIDANCE

Zexuan Li^1^, Katelyn Morrison^1^, Shuyi Han^2^, Jidapa Kraisangka^3^, Charles Fauvel^4^, Priscilla Correa‐Jaque^5^, Rebecca Vanderpool^5^, Yongqi Liu^5^, Shili Lin^5^, Adam Perer^1^, Allen Everett^6^, Manreet Kanwar^7^, Raymond Benza^8^

^1^Carnegie Mellon University, Pittsburgh, USA, ^2^University of California, San Diego, San Diego, USA, ^3^Mahidol University, Salaya, Thailand, ^4^Rouen University Hospital, Rouen, France, ^5^The Ohio State University, Columbus, USA, ^6^The Johns Hopkins University, Baltimore, USA, ^7^Allegheny General Hospital, Pittsburgh, USA, ^8^Mount Sinai Health System, New York, USA

Numerous graphical user interfaces and visualizations have been developed to aid clinicians in calculating the risk of patients with rare diseases, such as Pulmonary Arterial Hypertension (PAH). However, such current interfaces are simplistic and lack interactive designs to aid the clinician in comprehensibly understanding the progress of a patient's risk. Furthermore, they fail to provide clinicians with guidance on treatment plans. With the utilization of machine learning (ML) algorithms to augment clinicians' decision‐making workflows, interactive dashboards have the potential to provide insights that previously were not accessible. ML‐based interactive dashboards are starting to incorporate explanations, such as what‐if explanations, which allow clinicians to explore how different values for the model features impact the output (risk prediction). However, how clinicians want to interact with what‐if explanations and how they should be designed is unclear. Design a novel, interactive what‐if explanation visualization to help guide clinicians through machine learning‐based PAH risk stratification. We aimed to identify design goals for what‐if explanations that can be utilized while assessing the risk of and determining treatment plans for PAH patients. Based on the identified design goals, we designed and developed a clinical decision‐support tool with what‐if explanations for PAH risk assessment that clinicians can use in various contexts, such as one‐on‐one patient interaction and monthly PAH case meetings. We conducted semi‐structured need‐finding interviews and user studies with 28 PAH experts while iteratively prototyping different designs of a clinical decision‐support tool for PAH risk assessment. Prototyping stages consist of low‐fidelity, static to high‐fidelity, interactive prototypes, and a deployed, web‐based interactive dashboard. Interview transcripts were qualitatively analyzed to identify themes regarding the design of the dashboard and how clinicians would use the dashboard. Through rigorous qualitative analysis, we identified core design goals related to the what‐if explanations, the overall dashboard, and how the clinicians would adopt the what‐if explanations in various parts of their workflow, from risk assessment to identifying treatment plans. The primary ways that clinicians intended to use the what‐if explanations included (1) educating patients, caregivers, and less experienced clinicians about PAH; (2) motivating patients to improve their conditions by showing alternative negative and positive possibilities; (3) identifying the most appropriate treatment plans; and (4) understand how the underlying machine learning model works. Machine learning algorithms are increasingly being introduced to clinicians' decision‐making workflows, making it imperative to enhance the collaboration between the clinician and the predictive model. We designed several prototypes of what‐if explanations in a clinical decision‐support tool for Pulmonary Arterial Hypertension Risk Assessment Outcome, converging on one final design. Our interviews with 28 PAH experts suggest that clinicians will utilize the output from these predictive models in various ways (i.e., communicating with patients and determining treatment plans), making it necessary to design dashboards that prioritize safety.

## A106 “EVOLVING HAEMODYNAMIC PROFILES AND SURVIVAL TRENDS IN PATIENTS WITH PULMONARY HYPERTENSION: A SINGLE‐CENTRE STUDY”

Sofia Anastasia Mouratoglou^1^, Alexandra Arvanitaki^1^, Eleftherios Markidis^1^, Sophia‐Anastasia Mouratoglou^1^, Ioannis Farmakis^1^, Panagiotis Gourgiotis^1^, Thomas Chrysochoidis^1^, Christos Feloukidis^1^, Antonios Kouparanis^1^, Matthaios Didagelos^1^, Vasilios Grosomanidis^1^, Antonios Ziakas^1^, George Giannakoulas^1^

^1^AHEPA University General Hospital, Pulmonary Hypertension and Congenital Heart Disease Unit, Thessaloniki, Greece

Over the last 30 years, advances in the diagnosis and treatment of pulmonary hypertension (PH) significantly improved the disease course. This study aims to present the haemodynamic profile of PH patients at diagnosis in Greece and its temporal changes and investigate survival rates and potential prognostic factors. This retrospective cohort study included adult patients who underwent right heart catheterization (RHC) at Pulmonanry Hypertension Unit, AHEPA University Hospital of Thessaloniki, Greece, from January 2008 to June 2023 and were diagnosed with PH. Demographic and clinical data was collected at the time of RHC and survival data was collected from PH diagnosis till June 2023, including cause of death, when available. Patients were classified into PH groups and pulmonary arterial hypertension (PAH) subgroups based on clinical, haemodynamic, and laboratory data. The 3‐strata risk stratification model was applied to assess the mortality risk of PAH patients. Kaplan‐Meier curves were used to assess survival and cox‐regression analysis to identify prognostic factors. Among 294 patients who underwent RHC, 257 (87.4%) were diagnosed with PH (65% female, median age 63 years (IQR 23)), with group 1 being the most prevalent (46%). The diagnostic haemodynamic profile of patients within various PH groups was similar to previous registries. Overall, PH patients at diagnosis presented a median mean right atrial pressure (mRAP) of 7 mmHg (IQR 6), mean pulmonary artery pressure (mPAP) of 42 mmHg (IQR 18), pulmonary vascular resistance (PVR) of 6 WU (IQR 5), and cardiac index (CI) of 2.6 L/min/m2 (IQR 1). The corresponding data for PAH patients was 7 mmHg (IQR 6), 42 mmHg (IQR 18), 7 WU (IQR 6), and 2.6 L/min/m^2^ (IQR 1), respectively. Among PAH subgroups, connective tissue disease associated PAH (CTD‐PAH) and portopulmonary PH had the most favorable haemodynamics. The study, also, revealed temporal improvement in the diagnostic haemodynamic profile of PH patients, with a median mPAP and PVR of 56 mmHg and 13 WU between 2008 and 2013, 43 mmHg and 7 WU between 2013 and 2018, and 42 mmHg and 7 WU between 2018 and 2023, respectively. A similar improvement in mPAP and PVR was detected for PAH patients diagnosed after 2013 compared to those diagnosed between 2008 and 2013. Survival analysis demonstrated 5‐year survival rates of 65%, 59%, 39%, 77%, and 34% for groups 1 to 5 (p: 0.0039). The 5‐year survival rates for specific PAH subgroups were as follows: IPAH (90.3%), CTD‐PAH (58.7%), CHD‐PAH (81.3%), and portopulmonary PH (27%) (p: 0.0058). Notably, low‐risk PAH group presented similar 1‐year all‐cause mortality rates (12.4%) with high‐risk patients (12.8%) due to the majority of non‐PH‐related deaths in low‐risk patients (62%), while high‐risk patients died mostly due to PH (67%). Cox regression analysis identified only female gender as an independent factor for survival in PAH patients. This single‐center study contributes to our understanding of the evolving nature of PH care. The observed improvements in hemodynamic profiles highlight the importance of timely diagnosis and treatment, leading to a rather chronic disease with low‐risk patients dying mostly due to comorbidities.

## A107 LNCRNA16 AND LNCRNA19—MAJOR REGULATORS OF CELLULAR RESPONSES TO HYPOXIA IN THE LUNG

Zahraa Msheik^1,2^, Chanil Valasarajan^1,2^, Marek Bartkuhn^3,4^, Rajkumar Savai^1,2,4^, Werner Seeger^1,2,4^, Soni Savai Pullamsetti^1,2,4^

^1^Department of Lung Development and Remodeling, Max Planck Institute For Heart And Lung Research, Bad Nauheim, Germany, ^2^Department of Internal Medicine, Member of the German Center for Lung Research (DZL) and Cardio‐Pulmonary Institute (CPI), Justus Liebig University, Giessen, Germany, ^3^Biomedical Informatics and Systems Medicine, Justus Liebig University, Giessen, Germany, ^4^Institute for Lung Health (ILH), Justus Liebig University, Giessen, Germany

Hypoxia, a condition characterized by insufficient oxygen levels, plays a crucial role in various physiological and pathological events. The transcription factor HIFα, a key player in hypoxia, dysregulates multiple signaling pathways, inducing a pathological phenotype. Apart from the coding genes, recent studies have shown that several long noncoding RNAs (lncRNAs) are dysregulated under hypoxia. lncRNAs can regulate several signaling pathways by acting as RNA‐decoy, miRNA sponge, and splicing modulator. Notably, recent studies have shown that some of these lncRNAs can be further translated into micro‐peptides, which can be highly cell‐ and condition‐specific, opening up novel therapeutic options. In this project, we aim to delineate the role of high coding potential lncRNAs in modulating the hypoxia‐driven pathways in their RNA and micropeptide form and their contribution to the pathogenesis of chronic lung diseases. Screening for dysregulated lncRNAs in response to hypoxia was done by performing RNA‐seq on human pulmonary arterial smooth muscle cells (hPASMCs) exposed to 24 h of hypoxia (hox). The top ten most significantly upregulated candidates were further validated at different time points of hox exposure. Based on in silico analysis, three lncRNAs, LNC15, LNC16, and LNC19, were found to have high coding potential, suggesting their possible translation into micropeptides. Screening of the selected lncRNAs in different lung diseases revealed that LNC19 was significantly upregulated in both chronic obstructive lung disease (COPD) and idiopathic pulmonary fibrosis (IPF) while LNC19 displayed significant upregulation only in COPD. Interestingly, LNC16 and LNC19 were significantly upregulated in hPASMCs isolated from COPD‐PH patients compared to donor hPASMCs. Additionally, LNC19 exhibited significant upregulation in idiopathic pulmonary arterial hypertension (IPAH) isolated hPASMCs. Further investigations into the cellular localization of LNC16 and LNC19 revealed that both lncRNAs are predominantly expressed in the cytoplasm. To understand the upstream transcriptional regulation of these lncRNAs under hypoxia, their respective promoter regions were scrutinized for the presence of the hypoxia‐responsive element (HRE), the binding site for HIFα. Three HREs were found in the LNC16 promotor region, suggesting a HIFα based transcriptional regulation, whereas no HRE was found in the promoter region of LNC19. This was further validated by HIF1α knockdown experiments. Moreover, silencing LNC19 significantly induced antiproliferative effects in the hox‐exposed hPASMCs, whereas knocking down LNC16 showed induction of apoptotic resistant phenotype in the cells. Transcriptomic analysis revealed that silencing LNC16 and LNC19 dysregulates pathways associated with ribosomal biogenesis and translation in the hox‐exposed hPASMCs. Given the importance of cap‐independent translation, including the internal ribosome entry site (IRES) system, in driving the translation of hypoxia‐responsive genes to sustain the hypoxic response, the role of LNC16 and LNC19 in driving the IRES‐dependent translation under hypoxia and in COPD‐PH will be further examined.

## A108 REMODELING OF THE ENDOTHELIAL BASEMENT MEMBRANE IN PULMONARY HYPERTENSION DUE TO LEFT HEART DISEASE DRIVES INTIMAL SMOOTH MUSCLE CELL HYPERPLASIA

Netra Nambiar Veetil^1,2,3^, Tara Gransar^1,2^, Shao‐Fei Liu^1,3^, Robert Szulcek^1,3,4^, Volkmar Falk^2,3^, Mariya M. Kucherenko^1,2,3^, Wolfgang M. Kübler^1,3,5^, Christoph Knosalla^2,3^

^1^Institute Of Physiology ‐ Charité Universitätsmedizin Berlin, Berlin, Germany, ^2^Department of Cardiothoracic and Vascular Surgery, Deutsches Herzzentrum der Charité (DHZC), Berlin, Germany, ^3^DZHK (German Centre for Cardiovascular Research), partner site Berlin, Berlin, Germany, ^4^Department of Cardiac Anesthesiology and Intensive Care Medicine, Deutsches Herzzentrum der Charité (DHZC), Berlin, Germany, ^5^DZL (German Centre for Lung Research), partner site Berlin, Berlin, Germany

Pulmonary hypertension (PH) due to left heart disease (PH‐LHD) is first initiated by passive congestion of blood from the left heart into the pulmonary vasculature, but subsequently progresses by pulmonary arterial (PA) remodeling characterized by smooth muscle cell (SMC) hyperplasia. Yet, underlying pathomechanisms driving SMC hyperplasia in PH‐LHD are poorly understood and intervention strategies are lacking. Here, we addressed the role of the endothelial cell (EC)‐derived basement membrane (BM) in regulating SMC hyperplasia in PH‐LHD. PA samples were obtained from patients with end‐stage left‐heart disease (LHD) meeting clinical criteria of PH (PH‐LHD) or not (LHD w/o PH), or from healthy‐heart donors during orthotopic heart transplantation. Histological analyses revealed SMC hyperplasia in the PA intima as a hallmark of PH‐LHD that correlated with patients’ pulmonary hemodynamics, namely mean pulmonary arterial pressure and pulmonary vascular resistance. While SMC hyperplasia increased PH‐LHD PA wall thickness up to 10%, isolated SMC from PH‐LHD PAs showed increased cell migration and proliferation rates as compared to control SMC. Concomitantly, immunohistological detection of BM markers collagen IV and laminin identified endothelial BM remodeling as an early event in PA remodeling in LHD w/o PH patients before SMC hyperplasia and clinical PH. Based on these findings we hypothesized that early remodeling of the endothelial BM may enhance SMC migration and proliferation via extracellular matrix (ECM)‐to‐cell signaling. Consistent with this hypothesis, EC‐produced decellularized ECM (dECM) from PAs of LHD w/o PH patients increased proliferation and migration in control SMC, while control BM inhibited these responses in PH‐LHD SMC. We next considered an activation of mechanosensitive transcriptional co‐activator YAP‐1 as a mediator of BM effects on SMC. Indeed, culturing on dECM produced by LHD w/o PH or PH‐LHD EC increased nuclear (active) YAP‐1 abundance in control SMC, while culturing on dECM produced by control EC decreased nuclear YAP‐1 in PH‐LHD SMC. Inhibition of YAP‐1 by Verteporfin abolished the effects of LHD w/o PH and PH‐LHD endothelial dECM on proliferation and migration of human PA SMC cultured in vitro, and reduced PA SMC proliferation and migration in a rat model of PH‐LHD secondary to surgical aortic banding. Conclusion: Our findings identify remodeling of the endothelial BM (ECM) as an important pathomechanism driving SMC proliferation and migration in PH‐LHD via activation of YAP‐1. ECM remodeling and/or YAP‐1 activation may present promising therapeutic targets for preventing SMC hyperplasia in PH‐LHD. This research was supported by the German Center for Cardiovascular Research (DZHK), the State Ministry for Education and Research (BMBF), the German Foundation for Cardiac Research (DSHF), the German Society for Cardiology and Cardiovascular Research (DGK), and the German Research Foundation (DFG).

## A109 REMODELING OF THE ENDOTHELIAL BASEMENT MEMBRANE IN PULMONARY HYPERTENSION DUE TO LEFT HEART DISEASE DRIVES INTIMAL SMOOTH MUSCLE CELL HYPERPLASIA

Netra Nambiar Veetil^1,2,4,5^, Tara Gransar^1,2^, Shao‐Fei Liu^1,5^, Robert Szulcek^1,3,4,5,6^, Volkmar Falk^2,4^, Mariya Kucherenko^1,2,4,5^, Wolfgang Kübler^1,5,6^, Christoph Knosalla^2,4,5^

^1^Institute of Physiology, Charité – Universitätsmedizin Berlin, corporate member of Freie Universität Berlin and Humboldt‐Universität zu Berlin, Charitéplatz1, Berlin, Germany, ^2^Department of Cardiothoracic and Vascular Surgery, Deutsches Herzzentrum der Charité (DHZC), Augustenburger Platz 1, Berlin, Germany, ^3^Department of Cardiac Anesthesiology and Intensive Care Medicine, Deutsches Herzzentrum der Charité (DHZC). Augustenburger Platz 1, Berlin, Germany, ^4^Charité – Universitätsmedizin Berlin, corporate member of Freie Universität Berlin and Humboldt‐Universität zu Berlin, Charitéplatz 1, Berlin, Germany, ^5^DZHK (German Centre for Cardiovascular Research), partner site Berlin, Berlin, Germany, ^6^DZL (German Centre for Lung Research), partner site Berlin, Berlin, Germany

Pulmonary hypertension (PH) due to left heart disease (PH‐LHD) is first initiated by passive congestion of blood from the left heart into the pulmonary vasculature but subsequently progresses by pulmonary arterial (PA) remodeling characterized by smooth muscle cell (SMC) hyperplasia. Yet, underlying pathomechanisms driving SMC hyperplasia in PH‐LHD are poorly understood, and intervention strategies are lacking. Here, we addressed the role of the endothelial cell (EC)‐derived basement membrane (BM) in regulating SMC hyperplasia in PH‐LHD. PA samples were obtained from patients with end‐stage left‐heart disease (LHD) meeting clinical criteria of PH (PH‐LHD) or not (LHD w/o PH), or from healthy‐heart donors during orthotopic heart transplantation. Histological analyses revealed SMC hyperplasia in the PA intima as a hallmark of PH‐LHD that correlated with patients’ pulmonary hemodynamics, namely mean pulmonary arterial pressure and pulmonary vascular resistance. While SMC hyperplasia increased PH‐LHD PA wall thickness up to 10%, isolated SMC from PH‐LHD PAs showed increased cell migration and proliferation rates as compared to control SMC. Concomitantly, immunohistological detection of BM markers collagen IV and laminin identified endothelial BM remodeling as an early event in PA remodeling in LHD w/o PH patients before SMC hyperplasia and clinical PH. Based on these findings, we hypothesized that early remodeling of the endothelial BM may enhance SMC migration and proliferation via extracellular matrix (ECM)‐to‐cell signaling. Consistent with this hypothesis, EC‐produced decellularized ECM (dECM) from PAs of LHD w/o PH patients increased proliferation and migration in control SMC, while control BM inhibited these responses in PH‐LHD SMC. We next considered activation of mechanosensitive transcriptional co‐activator YAP‐1 as a mediator of BM effects on SMC. Indeed, culturing on dECM produced by LHD w/o PH or PH‐LHD EC increased nuclear (active) YAP‐1 abundance in control SMC, while culturing on dECM produced by control EC decreased nuclear YAP‐1 in PH‐LHD SMC. Inhibition of YAP‐1 by Verteporfin abolished the effects of LHD w/o PH and PH‐LHD endothelial dECM on proliferation and migration of human PA SMC cultured in vitro, and reduced PA SMC proliferation and migration in a rat model of PH‐LHD secondary to surgical aortic banding. Our findings identify remodeling of the endothelial BM (ECM) as an important pathomechanism driving SMC proliferation and migration in PH‐LHD via activation of YAP‐1. ECM remodeling and/or YAP‐1 activation may present promising therapeutic targets for preventing SMC hyperplasia in PH‐LHD. This research was supported by the German Center for Cardiovascular Research (DZHK), the State Ministry for Education and Research (BMBF), the German Foundation for Cardiac Research (DSHF), the German Society for Cardiology and Cardiovascular Research (DGK), and the German Research Foundation (DFG).

## A110 ASSESSMENT OF SUBMAXIMAL CARDIOPULMONARY HEMODYNAMICS IN PATIENTS WITH EXERCISE PULMONARY HYPERTENSION

Luiz Henrique Nascimento Campedelli^1^, Michael G. Risbano^1^

^1^University Of Pittsburgh, Pittsburgh, USA

There has been a renewed interest in investigating the cardiopulmonary hemodynamics of exercise pulmonary hypertension (ePH). Much of the work has focused on the hemodynamic definition of ePH1‐6, with the European Respiratory Society settling on a suitable definition for ePH and postcapillary ePH as a mean pulmonary artery pressure to CO slope >3.0 mmHg/L/min5. The characterization of submaximal physiological parameters in ePH patients, however, remains incompletely studied. The majority, if not all patients being evaluated for ePH have symptoms well before reaching peak exercise. Typically, after patients are diagnosed with ePH, peak exercise values are used to explain dyspnea during activities of daily living. We therefore sought to understand the hemodynamic responses for patients diagnosed with ePH at submaximal exercise. We performed a retrospective analysis of 25 patients who were diagnosed with ePH by invasive cardiopulmonary exercise testing (iCPET) and compared exercise results with 10 control subjects with normal iCPET results. All subjects had normal resting hemodynamics, particularly a mPAP < 25 mmHg. ePH was defined as the slope of mPAP/CO > 3 mmHg/L/min. We identified 50% of the peak V̇O2 achieved where the V̇O2 value sustained for 30 s (reported as 15 s average) as a submaximal midpoint. We then evaluated the associated cardiopulmonary exercise hemodynamics. Subjects were compared with a two‐sample *t*‐test. Data is expressed as mean ± SD. Demographics: The average age of the ePH group was 68.3 ± SD years; there were 18 (72%) females with a mean BMI of 31.4 kg/m^2^. The control group had an average age of 49.2 ± SD years, with five females (50%) and a BMI of 30.4 kg/m^2^. None of the control group patients had scleroderma or Raynaud's, though 5 ePH patients had these conditions. Similarly, five patients in the ePH group presented with interstitial lung disease, whereas no patient in the control group had this disease. Both the ePH and control groups had four patients each with a history of pulmonary embolism. Cardiopulmonary Hemodynamics: At rest, there were no differences between the two groups. At peak exercise we found differences in VCO2 (controls = 2213 ± 559.5; ePH = 1128 ± 336.5 mL/min, *p* < 0.001), oxygen consumption (V̇O2) (controls = 1928 ± 482.7; ePH = 972 ± 270.1 mL/min, *p* < 0.001), and ventilatory efficiency (V̇E/V̇CO2) (controls = 31.4 ± 3.3; ePH = 40.8 ± 6.3; *p* < 0.001). Significant differences were also noted at 50% V̇O2 peak, where V̇O2 (controls = 1040 ± 271; ePH = 549 ± 160 mL/min; *p* < 0.001), V̇CO2 (controls = 921 ± 269.8; ePH = 441 ± 119 mL/min; *p* < 0.001), RER (controls = 0.88 ± 0.07; ePH = 0.81 ± 0.07; *p* = 0.019), minute ventilation (controls = 27.6 ± 7.4; ePH = 17.9 ± 4.5 L/min; *p* = 0.002), V̇E/V̇CO2 (controls = 30.3 ± 2.7; ePH = 41.3 ± 6.3; *p* < 0.001) and end‐tidal CO2 (PETCO2) (controls = 37.5 ± 2.5; ePH = 32.3 ± 4.2; *p* < 0.001). Patients diagnosed with ePH often complain of exercise intolerance at submaximal levels of exercise, however, most of the initial studies on ePH have focused on peak exercise hemodynamics. Our results show lower oxygen consumption, reduced pulmonary perfusion, and evidence of ventilatory inefficiency at submaximal effort in patients with ePH compared to controls. Given that minute ventilation is lower in ePH at submaximal effort compared to controls, it is likely that the elevation in V̇E/V̇CO2 is due to increased dead space and not hyperventilation. These findings mirror submaximal values achieved in patients with resting pulmonary vascular disease7‐9.

## A111 THREE‐DIMENSIONAL (3D) RIGHT VENTRICULAR SURFACE STRAIN COMPUTED FROM 3D ECHOCARDIOGRAPHIC IMAGES REVEALS DIFFERENCES IN DEFORMATION BASED ON SEVERITY OF PULMONARY ARTERIAL HYPERTENSION

Hannah Oakland^1^, Lavanya Bellumkonda^2^, Lissa Sugeng^3^, Phillip Joseph^1^, Daniel Izzi^4^, Felicia Zalik^4^, Shannon McCabe^4^, Amjad Reza^4^ Ray Amendola^4^, Paul Heerdt^5^, Kendall Hunter^6^, Inderjit Singh^1^

^1^Yale University Section of Pulmonary, Critical Care, and Sleep Medicine, New Haven, USA, ^2^Yale University Section of Cardiovascular Medicine, New Haven, USA, ^3^Northwell Health, Manhasset, USA, ^4^Yale New Haven Hospital Heart and Vascular Center, New Haven, USA, ^5^Yale University Department of Anesthesiology, New Haven, USA, ^6^University of Colorado, Anschutz Medical Campus, Department of Bioengineering, Aurora, USA

Right ventricular (RV) adaptation to increased afterload is the single most important determinant of survival in pulmonary arterial hypertension (PAH). Despite shortcomings, echocardiography remains the mainstay of clinical cardiac imaging as it is noninvasive, portable, and inexpensive. Three‐dimensional (3D) echo‐based strain imaging describes the change in length of any portion of the heart as it moves through the cardiac cycle and has proven diagnostic value in left ventricular assessment. For the RV, however, commercially available technology is limited to the quantification of two‐dimensional (2D) strain solely in the longitudinal plane. Given the complexity of RV shape and motion, single‐planar 2D analysis of strain may prove progressively inadequate as disease states are linked to increasingly nuanced descriptions of 3D RV deformation. To address this issue, our team has developed a method to calculate maximum principal surface strain (PSMax) using data from bedside 3D echo that accounts for strain over the entire ventricular subendocardial surface. Image sequences are divided into approximately 900 small triangles, each of which is tracked as it changes shape and size throughout the cardiac cycle. As opposed to traditional strain metrics that require 5+ parameters to express surface deformation, PSMax analysis identifies vectors that represent the largest possible strains (and therefore maximally representative) over any given area, expressing each with only a magnitude and angle. Thus, using only two numbers, the dominant deformation of the ventricle is described, improving interpretability and providing concise representation of the dominant directionality of overall surface deformation. 3D echo‐based PSMax across the RV free wall was analyzed for 36 patients with PAH, of which 11 met the criteria for RV failure (right atrial pressure > 14 mmHg or cardiac index < 2 L/min/m^2^), and 23 control patients with normal right heart catheterization hemodynamics. PSMax was significantly different between PAH patients with RV failure (RV‐Fail) versus both non‐RV failure PAH patients (Non‐Fail), and controls (−21.0%, −25.9%, and −30.2%, respectively, *p*‐value < 0.05 for all pairwise comparisons). While circumferential strain was different between all groups, longitudinal strain, and 2D free wall strain were unable to differentiate RV‐Fail and Non‐Fail. Further, tricuspid annular plan systolic excursion (TAPSE) was only different between control and RV‐Fail. Finally, the angle of PSMax was oriented progressively longitudinally when comparing control, Non‐Fail, and RV‐Fail patients (40.7°, 38.1°, and 34.0°, respectively, *p*‐value < 0.05 for all comparisons). Study results demonstrate that PSMax elucidates differences in RV deformation across the spectrum of RV function in PAH. Although limited by sample size, this precise differentiation was not observed using currently established metrics of RV strain, including 2D free wall strain and TAPSE. We also show that PSMax has the additional benefit of measuring an aggregate angle of deformation that is distinct by disease severity. In health, RV myofibers are aligned in multiple directions, contributing to the complex motion of the RV. It has been shown in multiple studies that in PAH, these myofibers homogenize directions longitudinally; this effect has been shown to be pharmacologically modifiable, making it a potential therapeutic target.

## A112 THERAPEUTIC MODULATION OF MST1/2 KINASES REVERSE PULMONARY ARTERIAL HYPERTENSION VIA RHOA/ACTIN CYTOSKELETON DYNAMICS ALTERATION

Samuel Olapoju^1,2,3^, Eve Lemay S^4^, Prakash Chelladurai^1,2^, Dmitry A Goncharov^5^, Golnaz Hesami^1,2^, Rajkumar Savai^1,2^, Werner Seeger^1,2^, Babu Konda Kurakula^3^, Sebastian Bonnet^4^, Elena. A Goncharova^5^, Soni Savai Pullamsetti^1,2^

^1^Max Planck Institute For Heart and Lung Research, Bad Nauheim, Germany, ^2^Department of Internal Medicine, Justus Liebig University, Giessen, Germany, Giessen, Germany, ^3^Department of Physiology, Amsterdam University Medical Center, The Netherlands, ^4^Department of Medicine, Faculty of Medicine, Université Laval, Canada, ^5^Lung Center, Division of Pulmonary, University of California, Davis School of Medicine, USA

Pulmonary hypertension is a deadly disease characterized by hyperproliferation and reduced apoptotic ability of pulmonary resident cells. Although, current therapies focus on targeting vasoconstriction in other to slow the disease progression, which apparently remains ineffective. Thus, unravelling novel drugable signaling axis to be modulated as a therapeutic strategy is of upmost interest. The Mammalian STE20‐like protein kinases 1/2 (MST1/2) are core kinases of the HIPPO signaling axis involved in proliferation and survival, which we have previously reported as a driver of hyper proliferative and prosurvival phenotype in PAH. However, therapeutic modulation of MST1/2 to reverse the PAH‐like phenotype is unknown. Therefore, this study aims to decipher the molecular mechanism through which the modulation of MST1/2 kinases could be a novel therapeutic strategy in PAH. We profiled the expression of MST1/2 in human PAH patients versus healthy controls and in monocrotalin induced rat model of PAH. Interestingly, MST1/2 kinases were found to be upregulated in PASMCs isolated from our PAH patient cohort and also in Lung tissue homogenates obtained from monocrotalin induced rat model of PAH. Using MST1/2 knockout in mice as a genetic tool and XMU‐MP‐1 as MST1/2 specific inhibitor, our in vitro functional studies revealed that genetic ablation of MST1/2 in mice and Pharmacological treatment of human IPAH‐PASMCs with XMU‐MP‐1 caused phenotypic switch from pro‐PAH to anti‐PAH Phenotype. To elucidate the molecular mechanism through which MST1/2 inhibition reversed PAH‐like phenotype in PAH‐PASMCs, we subjected the inhibitor‐treated PAH‐PASMCs to RNAsequencing. KEEG and Reactome analysis showed RhoA/actin cytoskeleton dynamic was significantly altered upon treatment with XMU‐MP‐1 which was further confirmed with western blot analysis. In addition, daily intraperitoneal administration of XMU‐MP‐1 to monocrotaline‐induced PAH rat attenuated hemodynamic alterations and collagen deposition in Lungs of treated MCTinduced rats. Because right ventricular dysfunction is a common complication observed in PAH, we also investigated the effect of MST1/2 inhibitor treatment on RV structural alterations. We found that treated MCT‐induced PAH rats exhibited reduced RV collagen deposition, cardiomyocytes size, and improves vasculogenesis. It is important to mention that this study put both gender into consideration, making it one of the very few studies considering the possibility of gender‐driven differential response to treatment. Interestingly, both males and females of MCT‐induced rats showed ameliorated PAH pathology upon treatment with XMU‐MP‐1. From our current data, modulation of MST1/2 kinases could switch PAH‐like phenotype to antiPAH through RhoA‐dependent actin regulation. This is a novel mechanistic axis of modulating MST1/2 in PAH. Thus, a potential therapeutic mechanism. First author's contact: Samuel Olapoju (samuel. olapoju@mpi‐bn. mpg. de).

## A113 ALTERING MITOCHONDRIAL DYNAMICS IN ENDOTHELIAL CELLS RESULTS IN AGED‐DEPENDENT PULMONARY ARTERIAL HYPERTENSION

Bertha Garcia‐Leon^1^, Yolanda Sierra‐Palomares^1,2^, Daniel Lobato‐Alonso^1^, Susana F. Rocha^3^, Alvaro Macias^2,3^, Borja Ibanez^2,3,4^, Eduardo Oliver^1,2,3^

^1^Centro de Investigaciones Biologicas Margarita Salas (CIB), CSIC, Madrid, Spain, ^2^Centro Nacional de Investigaciones Cardiovasculares (CNIC), Madrid, Spain, ^3^Centro de Investigacion Biomedica en Red de Enfermedades Cardiovasculares (CIBERCV), Madrid, Spain, ^4^IIS‐Hospital Fundacion Jimenez Diaz, Madrid, Spain

Pulmonary Arterial Hypertension (PAH) is a rare disease with high mortality and morbidity. Among its characteristics, an endothelial dysfunction leading to vasoconstriction happens in the first stages of the disease. This is followed by an aberrant smooth muscle cell proliferation and endothelial‐mesenchymal transition leading to pulmonary vascular remodeling. These changes cause elevated pulmonary artery pressure and right ventricle hypertrophy, which evolves to failure and premature death very quickly. Interestingly, mitochondrial dysfunction has been previously related to be relevant in these pathological processes. Balanced mitochondrial dynamics is essential in a correctly functional cell. In a previous study, we described the ATP‐dependent metalloprotease YME1L to be an essential player in the regulation of mitochondrial fission/fusion balance and the specific lack of it within the cardiomyocyte has been reported to induced heart failure (Wai et al., Science 2015;350(6265):aad0116). In the current work, we hypothesize that the specific lack of Yme1l in the endothelial cell will alter mitochondrial dynamics, therefore disrupting cell homeostasis, which will cause endothelial dysfunction and PAH. To answer this hypothesis, we have developed a murine model of endothelial knockout Yme1l (eYKO) and have studied how the impaired mitochondrial fusion in the endothelial cells impacts the vascular health during aging. For the characterization of this novel murine model, we used different techniques of molecular biology, histology, and functional assays to elucidate whether or not eYKO mice developed PAH. Our results demonstrate that eYKO mice presented higher right ventricular systolic pressure and right ventricle hypertrophy along with vascular remodeling and endothelial dysfunction starting from a young age and worsening upon aging. Moreover, the exposure of eYKO to hypoxia exacerbates the PAH phenotype, thus highlighting the relevance of maintaining a correct mitochondrial wellness to prevent the development and evolution of the disease. In conclusion, we herein described a new animal model of PAH induced by altering mitochondrial dynamics in the endothelial cell. This model recapitulates some of the main pathophysiological characteristics of the disease; it begins early at a young age and keeps worsening during aging. Furthermore, eYKO mice stand as a perfect dual‐hit model to study the impact of a pre‐existing endothelial damage in the onset and evolution of pulmonary vascular diseases. Our results open new avenues for deeply understanding the disease and finding novel therapeutic approaches to treat PAH.

## A114 CLINICAL PHARMACOKINETICS OF AN EXTENDED‐RELEASE FORMULATION OF INHALED LIPOSOMAL TREPROSTINIL (L606) TO REDUCE DOSING FREQUENCY

Savan Patel^1^, Janet Tully^1^, Rajeev Saggar^1^, Jason Prabel^1^, Andreas Garcia^1^, Ko‐Chieh Chen^2^, Pei Kan^2^

^1^Liquidia Technologies, Morrisville, USA, ^2^Pharmosa Biopharm, Taipei, Taiwan

Treprostinil is a prostacyclin (PGI2) analog with a short half‐life used for the treatment of pulmonary arterial hypertension (PAH) and pulmonary hypertension associated with interstitial lung disease (PH‐ILD). Current inhaled therapies are immediate‐release formulations and require frequent dosing, up to every 4 h, due to the short half‐life. These immediate‐release formulations result in exposure only during the waking hours (14–16 h). Inhaled liposomal treprostinil (L606) is a novel extended‐release formulation designed to provide sustained plasma levels to reduce dosing frequency while extending daily exposure. L606 suspension is designed to control the release of the encapsulated treprostinil upon delivery to the lung, potentially reducing local respiratory tract irritation during treatment. A comparative bioavailability study in healthy volunteers was conducted to evaluate the pharmacokinetics of L606 (liposomal treprostinil). Subjects received L606 administered by a vibrating‐mesh nebulizer or a Tyvaso comparator. Blood samples were collected for 24 h after dosing and analyzed by LC‐MS‐MS. Systemic pharmacokinetics were evaluated for total exposure and dosing frequency. The systemic exposures of a single dose of L606, 51 µg, and Tyvaso, 54 µg, were compared. L606 resulted in a similar systemic exposure (AUCinf) compared to the equivalent dose of Tyvaso, with a significantly reduced peak plasma concentration (Cmax). The increased apparent half‐life (t1/2) of L606, combined with a plasma clearance rate comparable to that of Tyvaso, suggests that the liposomal formulation drives the controlled release of treprostinil after delivery to the lung. L606 demonstrates extended plasma concentrations up to 12 h after a single dose, supporting a reduction in dosing frequency to twice daily, or every 12 h. Peak and total exposure of treprostinil increased with increasing dose (data not shown). When evaluating the same total daily dose as Tyvaso, L606 provides sustained plasma concentrations with a similar total daily exposure. The extended‐release formulation results in a reduced peak‐to‐trough ratio to provide continuous coverage during waking and sleeping hours. The clinical pharmacokinetics of L606 in healthy volunteers demonstrate sustained plasma levels up to 12 h, supporting twice daily administration while maintaining exposure during sleeping hours. An open‐label study to assess safety of L606 in up to 60 patients with PAH and PH‐ILD patients transitioning from Tyvaso (neb or DPI), or PAH patients naïve to inhaled treprostinil is currently ongoing in the U.S.

## A115 PHYSIOLOGICAL AND CLINICAL OUTCOMES OF PATIENTS WITH IDIOPATHIC PAH AND A POSITIVE VASOREACTIVITY TEST: RESULTS FROM THE ASPIRE REGISTRY

Charlotte Pearson^1,2^, Frances Varian^1^, Jennifer T Middleton^2^, Sarah Binmahfooz^1^, Hamza Zafar^1^, Dharshan Neelam‐Naganathan^1^, Ze Ming Goh^2^, Christian Battersby^1^, Stefan Roman^2^, Jenna Ablott^2^, Neil Hamilton^2^, Iain Armstrong^2^, Judith Hurdman^2^, Abdul Hameed^2^, Athanasios Charalampopoulos^2^, Andrew J Swift^1^, A A Roger Thompson^2^, Robin Condliffe^2^, Charlie Elliot^2^, David G Kiely^2^, Alexander M K Rothman^2^

^1^Division of Clinical Medicine, University of Sheffield, Sheffield, UK, ^2^Sheffield Teaching Hospitals NHS Foundation Trust, Sheffield, UK

International guidelines recommend that patients with idiopathic pulmonary arterial hypertension (IPAH) and a positive vasoreactivity test be treated with calcium channel blocker therapy (CCB). We sought to evaluate the characteristics and outcomes of patients with a positive acute vasoreactivity test versus those with a negative test and compare clinical outcomes in long‐term responders to CCB (CCB‐LT) to non‐responders (based on clinical status at 1 year), including a cohort of patients implanted with pulmonary artery pressure and insertable rhythm monitors. Clinical data was collected from consecutive patients from the ASPIRE Registry (22/EE/0011) who had undergone acute vasoreactivity testing, between February 2001 and April 2023. Patients underwent clinical assessment including echocardiography, blood testing, exercise testing, lung function testing, multimodality imaging, and right heart catheterisation (RHC). Mortality data was collected from the NHS Personal Demographics Service. All patients on PAH therapies were followed up as part of the national service specification for patients with PH. Remote pulmonary haemodynamic data was also collected from a cohort of patients with IPAH enrolled in FIT‐PH (19/YH/0354) who were implanted with a pulmonary artery pressure monitor (CardioMems, Abbott) and insertable cardiac monitor (LinQ, Medtronic Inc.). Remote data was collected via regulatory‐approved online portals. Data analysis was undertaken in IBM SPSS, version 28.0. Patients with a positive acute vasoreactivity test were younger with less severe disease compared to patients with a negative test including significantly lower WHO FC (*p* < 0.001) and higher incremental shuttle walk (ISWT) distance (280 m vs 80 m, *p* < 0.001). These patients also had a significantly better median survival of 12.01 years versus 3.04 years, compared to non‐responders. Of 57 patients with an acute vasoreactive response, 24 patients were CCB‐LT responders. There was no significant difference in age or sex however, CCB‐LT responders had lower risk indicators (WHO FC I & II: 62.5% vs 30.0, *p* = 0.017), better quality of life outcome measures (Emphasis‐10: 28.00 vs 15.83) and less severe pulmonary haemodynamics (mRAP(mmHg): 6.04 vs 8.41), (PVR (dynes): 517 vs 702) compared to non‐responders. At 1‐year follow‐up, average ISWT significantly improved from 373 m at baseline to 537 m (*p* = <0.001) compared to no significant change in the non‐LT responders (270–280 m, *p* = 0.211). Five‐year survival in CCB‐LT responders was better compared to non‐responders at 98% compared to 70%, respectively. Six patients with a positive vasoreactivity test were enrolled in FIT‐PH. Remote monitored pulmonary artery pressure, cardiac output, and total pulmonary resistance were improved in patients with a CCB‐LT response (*n* = 3) compared with those who did not have a CCB‐LT response (*n* = 3) with changes identifiable at 20 days. Patients with IPAH with a positive acute vasoreactivity response have better functional outcomes and significantly better survival, compared to acute non‐responders. The survival of CCB‐LT responders is excellent. In addition, Implantable pulmonary artery pressure monitors are capable of identifying differences between CCB‐LT responders and non‐responders, much earlier than previously described.

## A116 COMPARATIVE ANALYSIS OF CIRCULATING ENDOTHELIAL PROGENITOR CELLS AND RESECTED TISSUE‐DERIVED ENDOTHELIAL CELLS IN CHRONIC THROMBOEMBOLIC PULMONARY HYPERTENSION (CTEPH)

Míriam Peracaula^1^, Cristina Rodríguez^1,2^, Ana M Ramírez^2^, Jeisson Osorio^2,3^, Montserrat Rigol^4,5^, Isabel Blanco^2,3^, Victor I Peinado^3,6^, Manel Castellà^7^, Joan Albert Barberà^2,3^, Olga Tura‐Ceide^1,2,3^

^1^Department of Pulmonary Medicine, Josep Trueta University Hospital de Girona, Santa Caterina Hospital de Salt and the Girona Biomedical Research Institute (IDIBGI), Girona, Spain, ^2^Department of Pulmonary Medicine, Servei de Pneumologia, Hospital Clínic‐Institut d'Investigacions Biomèdiques August Pi I Sunyer (IDIBAPS), University of Barcelona, Barcelona, Spain, ^3^Biomedical Research Networking Centre on Respiratory Diseases (CIBERES), Madrid, Spain, ^4^Cardiovascular Institute, Hospital Clínic de Barcelona‐Institut d'Investigacions Biomèdiques August Pi I Sunyer (IDIBAPS), University of Barcelona, Barcelona, Spain, ^5^Biomedical Research Networking Center on Cardiovascular Diseases (CIBERCV), Madrid, Spain, ^6^Department of Experimental Pathology, Institut d'Investigacions Biomèdiques de Barcelona (IIBB), CSIC‐IDIBAPS, Barcelona, Spain, ^7^Department of Cardiovascular Surgery, Cardiovascular Institute, Hospital Clínic, University of Barcelona, Barcelona, Spain

Endothelial dysfunction (ED) plays a crucial role in CTEPH pathogenesis. Previous studies have demonstrated that endothelial cells (ECs) within the occluding thromboembolic material exhibit phenotypic and functional alterations leading to impaired endothelial function. Nevertheless, systemic ED in CTEPH and its implications remain poorly understood. This study aims to investigate whether the observed ED in CTEPH patients is limited to the ECs derived from the affected thromboembolic tissue (CTEPH‐EC) or if also affects systemically circulating endothelial progenitor cells (CTEPH‐ECFC). Consequently, our objective is to ascertain whether distinctions exist in the extent of ED between CTEPH‐EC and CTEPH‐ECFC. We simultaneously isolated CTEPH‐EC and CTEPH‐ECFC from the same patients. Control samples were isolated from systemic circulation (CTL‐ECFC) and pulmonary artery (HPAE) of healthy individuals. We conducted phenotype analyses and functional assays, including assessments of proliferative capacity, reparative ability, angiogenic capacity, and senescence parameters. Additionally, we studied oxidative stress and mitochondrial membrane potential. CTEPH‐EC and CTEPH‐ECFC exhibit deregulated proliferation patterns estimated by double time population variable. Both groups showed lower division rate compared to their respective controls, CTEPH‐EC versus HPAE:(≈2 vs. 1 das, *p* < 0.01) CTEPH‐ECFC versus CTL‐ECFC:(≈6 vs. 1 days, *p* < 0.01). Wound healing ability, evaluated through migration assays, were notably decreased in both groups compared to controls: CTEPH‐EC (31.22% vs. 73.97%, *p* < 0.01) and CTEPH‐ECFC (52.63% vs. 94.24%, *p* < 0.01). Angiogenic ability was similarly diminished in both groups: CTEPH‐EC (177 vs. 272, *p* < 0.05) and CTEPH‐ECFC (223 vs. 110, *p* < 0.05). Furthermore, both patients groups exhibited a higher degree of mitochondrial membrane polarization, as indicated by JC‐1 dye, suggesting mitochondrial dysfunction: CTEPH‐EC (0.5383 vs. 1.319, *p* < 0.05) and CTEPH‐ECFC (0.2484 vs. 1.239, *p* < 0.05). Additionally, there was a higher percentage of senescent cells in both groups, as quantified by β‐galactosidase‐positive cells: CTEPH‐EC (17% vs. 5%, *p* < 0.01) and CTEPH‐ECFC (14% vs. 7%, *p* < 0.05). Elevated levels of oxidative stress were also observed in both CTEPH‐EC (20.28 vs. 13.35, *p* < 0.05) and CTEPH‐ECFC (22.50 vs. 6.75, *p* < 0.01). These preliminary findings provide unprecedented evidence of ED in paired patient samples of CTEPH‐EC and CTEPH‐ECFC. This demonstrates that the ED observed in CTEPH patients extends beyond endothelial cells derived solely from affected tissue, impacting circulating endothelial cells.

## A117 DYSREGULATED KLOTHO/FGF23 SIGNALING EXACERBATES PULMONARY ARTERIAL HYPERTENSION

Paul‐Lennard Perret^1,2,3^, Jonathan Lauryn^1,2^, Christiane Ott^2,4^, Willem Bintig^1^, Teresa C. Funk‐Hilsdorf^1,2,3^, Szandor Simmons^1,2^, Laura Michalick^1^, Philip Solymosi^1^, Gabor Kovacs^5,6^, Makoto Kuro‐O^7^, Jakob Voelkl^8^, Jana Grune^1,2,9^, Wolfgang Kuebler^1,2^

^1^Institute of Physiology, Charité ‐ Universitätsmedizin Berlin, Berlin, Germany, ^2^German Center for Cardiovascular Research (DZHK), Partner Site Berlin, Germany, ^3^Berlin Institute of Health (BIH), Berlin, Germany, ^4^Department of Molecular Toxicology, German Institute of Human Nutrition, Potsdam‐Rehbrücke, Germany, ^5^Medical University of Graz, Graz, Austria, ^6^Ludwig Boltzmann Institute for Lung Vascular Research, Graz, Austria, ^7^Division of Anti‐aging Medicine, Center for Molecular Medicine, Jichi Medical University, Shimotsuke, Japan, ^8^Institute for Physiology and Pathophysiology, Johannes Kepler University Linz, Linz, Austria, ^9^Department of Cardiothoracic and Vascular Surgery, Deutsches Herzzentrum Der Charité (DHZC), Berlin, Germany

While pulmonary arterial hypertension (PAH) was initially described as a disease that primarily affects young women, recent registries identified an increasing population of older patients suffering from PAH. Physiological aging is associated with a decrease in the renal “antiaging” transmembrane protein Klotho, which plays a critical role in multiple regulatory processes as a coreceptor for Fibroblast Growth Factor 23 (FGF23). In this study, we aimed to explore the role of Klotho and its downstream signaling pathways in the development of PAH. To this end, we exposed male C57BL/6 wildtype mice (WT) and klotho‐haploinsufficient (kl/+) mice to chronic hypoxia (10% O2) or normoxia for 14 days. Klotho deficiency aggravated the development of hypoxia‐induced PAH, as demonstrated by exacerbated right ventricular (RV) systolic pressures (RVSP) assessed by invasive right heart catheterization compared to WT littermates. Consistent with these data, noninvasive small animal echocardiography revealed lower pulmonary artery acceleration time‐to‐pulmonary artery ejection time ratios (PAT/PET), an indicator of PAH severity, in hypoxic kl/+ mice compared to hypoxic WT mice. Analogously, kl/+ mice hypoxic mice had higher peak pulmonary artery velocities, and more pronounced RV hypertrophy and dysfunction when compared to hypoxic WT controls, as indicated by Fulton's index and echocardiographically assessed tricuspid annular plane systolic excursion (TAPSE). H&E‐stained histological lung cross‐sections showed increased muscularization of small pulmonary arterioles in hypoxic kl/+ mice compared to hypoxic WT mice. ELISA‐based measurements revealed elevated FGF23 plasma levels in normoxic kl/+ mice as compared to WT, with even greater levels in hypoxic kl/+ mice. Similarly elevated levels of FGF23 were detected in plasma of IPAH patients relative to controls. In cell culture experiments, we found recombinant FGF23 to increase proliferation in pulmonary artery smooth muscle cells (PASMC) relative to vehicle‐treated cells, pointing towards a mechanistic involvement of FGF23 in pulmonary vascular remodeling and PAH. Our findings identify dysregulated Klotho/FGF23 signaling as a novel pathomechanism of lung vascular remodeling and PAH. This emerging adverse kidney‐lung interaction may play a critical role in PAH development in elderly and/or patients with chronic kidney disease. Supported by the Focus Area DynAge, the German Research Foundation (DFG) in the framework of CRC1470, subproject A04, and the German Centre for Cardiovascular Research (DZHK).

## A118 UNVEILING THE POWER OF BMPR2: AMPLIFYING PULMONARY HYPERTENSION AMIDST RIGHT PULMONARY ARTERY STENOSIS

Alban Todesco^1^, Julien Grynblat^2^, Firmin Akoumia^2^, Pr Damien Bonnet^3^, Pedro Mendes‐Ferreira^4^, Stephan Morisset^5^, Pr Denis Chemla^6^, Pr Marilyne Levy^3^, Mathilde Méot^3^, Sophie‐Guiti Malekzadeh‐Milani^3^, Carine Vastel^3^, Maria Rosa Ghigna^7^, Pr Marc Humbert^8^, Pr David Montani^8^, David Boulate^1^, Frederic Perros^9^

^1^Department of Thoracic Surgery‐Hôpital Nord‐APHM‐Aix‐Marseille University, Marseille, France, ^2^INSERM UMRS 999, Hôpital Marie Lannelongue, Le Plessis Robinson, France, ^3^Paris Cité University, Paris, France; Assistance Publique‐Hôpitaux de Paris, Necker‐Enfants malades Hospital, M3C, France, ^4^Department of Surgery and Physiology, Cardiovascular R&D Centre‐UnIC@RISE, Faculty of Medicine of the University of Porto, Porto, Portugal, ^5^Freelance Biostatistician, Pérouges, France, ^6^Service d'Explorations Fonctionnelles Multidisciplinaires Bi‐site, Hôpitaux Antoine Béclère‐Kremlin Bicêtre, Faculté de médecine‐Université Paris Saclay DMU‐CORREVE, AP‐HP, Le Kremlin‐Bicêtre, France, ^7^Department of Pathology, Institut Gustave Roussy, Villejuif, France, ^8^Université Paris‐Saclay, Faculté de Médecine, Le Kremlin‐Bicêtre, France; INSERM UMR_S 999 “Hypertension pulmonaire: Physiopathologie et Innovation Thérapeutique”, Hôpital Marie Lannelongue, Le Plessis‐Robinson, France; Assistance Publique ‐ Hôpitaux de Paris (AP‐HP), Service de Pneumologie et Soins Intensifs Respiratoires, Centre de Référence de l'Hypertension Pulmonaire, Hôpital Bicêtre, Le Kremlin‐Bicêtre, France, ^9^CarMeN Laboratory, INSERM U1060, INRAE U1397, Université Claude Bernard Lyon1, Pierre‐Bénite, France

The primary genetic risk factor for heritable pulmonary arterial hypertension (hPAH) is the presence of monoallelic mutations in the gene responsible for encoding bone morphogenetic protein receptor 2 (BMPR2). The incomplete penetrance of BMPR2 mutations implies that additional triggers are necessary for the occurrence of PAH. Pulmonary artery stenosis, whether congenital or acquired, can affect different levels of the pulmonary arterial tree. Significant stenosis of the lumen size in PA stenosis directly raises pulmonary artery pressure, while the redirection of blood flow to unobstructed arteries can lead to endothelial dysfunction and vascular remodeling. Consequently, this results in an increase in pulmonary vascular resistance (PVR) and the development of PH. Based on these observations, we hypothesized that right pulmonary artery stenosis (RPAS) could act as a trigger for the development of pulmonary hypertension (PH) in rats with Bmpr2 mutations. We report the clinical, functional, radiologic, and hemodynamic characteristics at diagnosis and outcomes of 10 patients with pulmonary artery stenosis and PH. Male and female rats with a 71 bp monoallelic deletion in exon 1 of Bmpr2 (Δ71 rats) and their wild‐type (WT) siblings underwent acute and chronic right pulmonary artery stenosis (RPAS). They were then subjected to full high‐fidelity hemodynamic characterization. We also examined how chronic RPAS can mimic the pulmonary gene expression pattern associated with installed PH. To achieve this, we conducted a comprehensive analysis using quantitative real‐time PCR, evaluating a panel of 90 genes associated with PH. All patients had significant functional impairment, with three patients in NYHA Functional Class II, three in NYHA Functional Class III, and four in NYHA Functional Class IV. All patients exhibited a precapillary component, with one having combined pre‐ and post‐capillary PH. No patients displayed positive acute vasoreactivity testing. RPAS‐induced PH in both male and female rats, both acutely and chronically. The unaltered left atrial pressure (LAP) signifies the precapillary origin of acute RPAS‐induced PH. Bmpr2 mutant and male rats manifested more severe PH compared to their counterparts. While WT rats adapted to RPAS, Bmpr2 mutant rats experienced heightened mortality. RPAS induced a decline in cardiac contractility index, particularly pronounced in male Bmpr2 rats. Chronic RPAS resulted in elevated pulmonary interleukin‐6 (IL‐6) expression and decreased Gdf2 expression (corrected *p*‐value < 0.05 and log2 fold change > 1). In this context, male rats expressed higher levels of endothelin‐1 and IL‐6 than females. Our novel two‐hits rat model presents a promising avenue for investigating the pathogenesis of precapillary PH, shedding light on pertinent sex and gene‐related effects.

## A119 ROLE OF CARBONIC ANHYDRASES 9 AND 12 IN PULMONARY HYPERTENSION

Aleksandar Petrovic^1^, Djuro Kosanovic^1,2^, Oleg Pak^1^, Akylbek Sydykov^1^, Norbert Weissmann^1^, Werner Seeger^1^, Ralph Theo Schermuly^1^

^1^University Of Giessen And Marburg Lung Center (UGMLC), Member of the German Center for Lung Research (DZL), Giessen, Germany, ^2^I.M. Sechenov First Moscow State Medical University (Sechenov University), Moscow, Russia

Carbonic anhydrases (CAs) are a family of enzymes that reversibly catalyze carbon dioxide hydration to bicarbonate and protons. Elevated expression of membrane‐bound CA9 and 12 isoforms has been reported in various types of cancer, and it was in many instances associated with disease progression and response to therapy. Pulmonary vascular remodeling as a hallmark of pulmonary hypertension (PH) has been characterized by several cancer‐like features, including dysregulated proliferation and migration of pulmonary vascular cells. Therefore, we hypothesize that CA9 and 12 play an important role in the pathogenesis of hypoxia and non‐hypoxia‐induced PH. Our initial investigation showed elevation of CA9 and 12 mRNA and protein expression profile in the lungs of patients with idiopathic pulmonary arterial hypertension (IPAH), compared to donors. Immunohistochemical analysis demonstrated that CA9 and 12 are indeed expressed in pulmonary vasculature. Furthermore, there was a significant increase in CA9 and 12 expression in human pulmonary artery smooth muscle cells (PASMCs) after hypoxia and non‐hypoxia stimulation. Additionally, using the small molecule inhibitor, we have shown the involvement of CA9 and 12 in the proliferation and migration of human PASMCs. Maintaining of optimal intracellular pH is a crucial event following the metabolic shift from oxidative phosphorylation to glycolysis, a feature shared in both cancer and PH. By pharmacological inhibition, we have demonstrated the important role of CA9 and 12 in human PASMCs intracellular pH homeostasis. In addition, CA9 and 12 inhibition lead to a significant decrease in acidification of PAMSCs extracellular milieu. Therefore, our data indicates CA9 and 12 elevated expression and an important role in the function of pulmonary vascular cells in pulmonary hypertension, highlighting carbonic anhydrases as potential players in the pathology of this severe and life‐threatening pulmonary vascular disease.

## A120 CARDIAC BAROREFLEX AND HEART RATE VARIABILITY IMPAIRMENT ARE ASSOCIATED WITH WORSENED EXERCISE CAPACITY AND QUALITY OF LIFE IN PULMONARY ARTERIAL HYPERTENSION

Michael Plunkett^1^, Ana Sayegh^1^, Tanya McWilliams^2^, Sasiharan Sithamparanathan^2^, Julian Paton^1^, Assoc. James Fisher^1^

^1^Manaaki Manawa – The Centre for Heart Research, Department of Physiology, Faculty of Medical and Health Sciences, University Of Auckland, Auckland, New Zealand, ^2^Respiratory Medicine, Te Toka Tumai Auckland, Te Whatu Ora Health New Zealand, Auckland, New Zealand

Autonomic control is abnormal in pulmonary arterial hypertension (PAH) with low cardiovagal tone occurring alongside sympathetic and adrenergic overactivation. This could impair exercise cardiovascular responses and play a role in exercise limitation, a symptom that most patients with PAH continue to suffer. We assessed cardiovascular autonomic function using heart rate variability (HRV), spontaneous cardiac baroreflex sensitivity (cBRS), and beat‐to‐beat blood pressure variability (BPV) and explored their relationship with exercise capacity, health‐related quality of life, and haemodynamic characteristics. Fourteen PAH patients (nine females, age 48 ± 13 years; mean ± SD) and 14 healthy controls (nine females, age 49 ± 14 years) were recruited. Haemodynamic right heart catheter (RHC) results and 6‐min walk distance (6MWD) were collected from clinical records. WHO functional class (WHO FC) and emPHasis‐10 (health‐related quality of life) were assessed. Participants underwent a 10‐min supine resting measurement of heart rate (HR; lead II electrocardiogram) and beat‐to‐beat blood pressure (BP; finger photoplethysmography) for HRV, cBRS (sequence technique), and BPV assessment. Resting baroreflex effectiveness index (BEI; proportion of systolic BP changes that lead to a corresponding HR change), and HRV parameters total power and root mean square of successive differences (RMSSD) were lower in PAH than controls (*p* = 0.006, *p* = 0.033, and *p* = 0.030, respectively). BPV measures were not different between PAH and healthy controls. Lower BEI correlated with poorer 6MWD (R2 = 0.509, *p* = 0.021), WHO FC (R2 = 0.440, *p* = 0.037) and emPHasis‐10 (R2 = 0.567, *p* = 0.012). Lower HRV total power and RMSSD were associated with poorer 6MWD (R2 = 0.634, *p* = 0.003; and R2 = 0.407, *p* = 0.035 respectively), WHO FC (R2 = 0.586, *p* = 0.006; and R2 = 0.421, *p* = 0.031 respectively) and emPHasis‐10 (R2 = 0.570, *p* = 0.007; and R2 = 0.501, *p* = 0.015 respectively). Baroreflex and HRV measures did not correlate with RHC haemodynamic measures. Collectively, these findings support the existence of cardiac autonomic dysfunction in PAH and further indicate that it is associated with reduced exercise capacity, worsened functional class, and poorer health‐related quality of life, independently of PAH haemodynamic severity.

## A121 LOW‐DOSE DOPAMINE LOWERS PERIPHERAL CHEMOREFLEX SENSITIVITY, IMPROVES VENTILATORY EFFICIENCY BUT NOT EXERCISE TOLERANCE IN PULMONARY ARTERIAL HYPERTENSION

Michael Plunkett^1^, Ana Sayegh^1^, Tanya McWilliams^2^, Sasiharan Sithamparanathan^2^, Julian Paton^1^, Assoc. James Fisher^1^

^1^Manaaki Manawa – The Centre for Heart Research, Department of Physiology, Faculty of Medical and Health Sciences, University Of Auckland, Auckland, New Zealand, ^2^Respiratory Medicine, Te Toka Tumai Auckland, Te Whatu Ora Health New Zealand, Auckland, New Zealand

Exercise limitation affects patients with pulmonary arterial hypertension (PAH) and worsens quality of life. Peripheral chemoreflex sensitivity (i.e., the hypoxic ventilatory response) is exaggerated in PAH, but whether this drives excess ventilation and limits exercise tolerance in PAH is unknown. Therefore, we tested the hypothesis that infusion of low‐dose dopamine, to inhibit the peripheral chemoreflex, would improve ventilatory efficiency, dyspnoea, and exercise performance in PAH. In a randomised, cross‐over, single‐blinded study, patients with PAH underwent submaximal constant work‐rate cycle exercise testing (75% of maximal work‐rate) with low‐dose dopamine infusion (2 μg∙kg^−1^∙min^−1^) and normal saline (control) infusion, until exhaustion. Under each condition, assessment of resting peripheral chemoreflex sensitivity (slope of ventilation to oxygen saturation) was performed using an isocapnic hypoxic rebreathing method targeting an end‐tidal O_2_ of 45 mmHg, with measurement of the ventilatory response. Exercise cardiorespiratory responses were compared between conditions at 25, 50, 75% and 100% of total exercise time using mixed model for repeated measures. In preliminary findings from five patients with PAH (three females, 47.8 ± 12.9 years; mean ± SD), peripheral chemoreflex sensitivity was reduced by 45% with dopamine compared to saline (0.61 ± 0.50 vs. 1.10 ± 0.71 L∙min^−1^ ∙ %−1 respectively; *p* = 0.123). V̇E/V̇CO2 ratio (i.e., at 50% exercise duration: 34.2 ± 3.3 vs. 36.5 ± 3.7 mmHg respectively; condition *p* = 0.035) and end‐tidal O2 (i.e., at 50% exercise duration: 116 ± 1 vs. 119 ± 3 mmHg respectively; condition *p* = 0.017) were also lower with dopamine compared to saline, while end‐tidal CO_2_ was increased (i.e., at 50% exercise duration: 32.9 ± 2.5 vs. 30.8 ± 2.5 mmHg respectively; condition *p* = 0.002). However, neither perception of dyspnoea (Borg 0–10 dyspnoea score; condition *p* = 0.828) nor exercise duration (771 ± 535 vs. 751 ± 478 s respectively; *p* = 0.932) were different between dopamine and saline. These preliminary findings indicate that peripheral chemoreflex inhibition improves ventilatory efficiency during submaximal exercise in PAH but does not improve dyspnoea or exercise tolerance.

## A122 NOVEL, OFF‐LABEL USE OF THE CARDIOMEMS™ REMOTE PULMONARY ARTERIAL PRESSURE MONITORING DEVICE IN MANAGEMENT OF IDIOPATHIC PULMONARY HYPERTENSION IN A PEDIATRIC PATIENT. CLINICAL REPORT AND LITERATURE REVIEW

Katarzyna Polak^1^, Inmaculada Guillén Rodríguez^1^, Maria Jesus Del Cerro, Mrs. Félix Félix Coserría Sánchez^1^, Nassib Salomon Fadul^1^

^1^Department of Paediatric Cardiology of Hospital Universitario Virgen del Rocío, Seville, Spain, ^2^Department of Paediatrics, Hospital Universitario Nuestra Señora la Candelaria, Santa Cruz de Tenerife, Spain, ^3^Department of Paediatric Cardiology of Hospital Rámon y Cajal, Madrid, Spain

Management of pulmonary arterial hypertension (PAH) precises close monitoring of pulmonary artery pressure (PAP). However, the risk associated with catheterization and general anesthesia, precludes frequent monitoring. The CardioMEMS™ HF System is an implantable monitor capable of measuring PAP, approved by FDA for adult patients with heart failure. It has been proven to provide useful information to monitor PAH therapy in adults, and demonstrated short‐ and long‐term safety. We present a male patient who underwent annual follow‐up visits in pediatric cardiology consult since birth due to the family history of a brother who died at 15 months of age because of PAH. The annual echocardiographic controls resulted normal until the age of 10, when the patient presented to consult referring episodes of presyncope and dyspnea, and echocardiography revealed signs of mild PAH, which was later confirmed in diagnostic catheterization (PAP 43/7/22 mmHg with normal pulmonary vascular resistance and negative vasoreactivity test). The guided interview revealed that 18 months prior, the patient was diagnosed with ADHD and started treatment with methylphenidate. Laboratory analyses for PAH workup including genetic tests resulted normal. The initial treatment included bosentan, sildenafil, and suspension of methylphenidate, with complete remission of symptoms during the period of 6 years. At 16 years of age, the patient began to present syncopes. The diagnostic catheterization (PAP 49/9/29 mmHg), ET, cardiac MR, and echocardiography did not reveal relevant changes with respect to previous studies. The symptoms have not been remitted regardless of the increase of bosentan and sildenafil doses and triple therapy with Selexipag as part of the clinical trial SALTO. Due to high clinical suspicion of PHA as an etiology of his symptoms, CardioMEMS™ device was implanted and permitted continuous, real‐time monitoring of PAP that demonstrated high variability of PAP throughout the day with peaks up to 92/72/46 mmHg, which had never been previously detected by catheterization or echocardiography. Despite a negative vasoreactivity test, calcium channel blockers were added to the therapy to prevent peaks of increased PAP that were most likely the cause of the syncopes. Real‐time recordings provided by the CardioMEMS™, permitted observing the effect of medication change and revealed lower variability of PAP curve and decreased PAP peaks (61/38/18 mmHg) with remission of the symptoms. Currently, at 17 years of age, the patient remains asymptomatic with elevated PAP, therefore the treatment will be further optimized to improve the prognosis. The literature review identified 4 articles reporting use of CardioMEMS™ in pediatric Fontan patients and patients with ventricular assist devices, indicating that the device may be safely utilized in the pediatric population. CardioMEMS™ device is a safe, reliable, and useful tool for the management of PAH, strongly facilitating close monitoring of patients, enabling medication titration, and anticipating the onset of symptoms. We believe that CardioMEMS™ will revolutionize the management of PAH, avoiding repeated invasive measurements and outweigh indirect measures, which until now have been the most accessible tools for the management of PAH and on which the majority of the indications of the current clinical guidelines are based.

## A123 POST‐COVID‐19 PATIENTS SHOW VASCULAR SEQUELAE THAT LAST UP TO 12 MONTHS

Paula Poyatos^1,3^, Neus Luque^1^, Gladis Sabater^2^, Saioa Eizaguirre^2^, Marc Bonnin^2^, Ramon Orriols^1,2,3,4^, Olga Tura‐Ceide^1,3,4^

^1^Girona Biomedical Research Institute (IDIBGI), Girona, Spain, ^2^Department of Pulmonary Medicine, Josep Trueta University Hospital de Girona, Santa Caterina Hospital de Salt, Girona, Spain, ^3^Department of Medical Sciences, Faculty of Medicine, University of Girona, Girona, Spain, ^4^Biomedical Research Networking Centre on Respiratory Diseases (CIBERES), Madrid, Spain

SARS‐CoV‐2 infection causes severe endothelial damage and endothelial dysfunction, an essential step for cardiovascular complications including coagulopathies and thromboembolisms. Endothelial progenitor cells (EPCs) could act as a biomarker of vascular damage, but its role in SARS‐CoV‐2 is unknown. The study aims to evaluate whether the number of EPCs and angiogenic biomarkers remains altered after 6 and 12 months postinfection and whether this alteration differs in COVID‐19 patients who suffered an acute pulmonary embolism (PE) from those who did not. Moreover, it will be evaluated whether this imbalance correlated with the presence of long‐COVID syndrome and other biological parameters measured. Seventy‐two patients were recruited in the study at different time points after overcoming COVID‐19 and 31 healthy controls. All subjects were matched for age, gender, BMI, and comorbidities. EPCs were obtained from peripheral blood mononuclear cells, cultured with specific medium and conditions. The appearance, number of colonies, and days of appearance were quantified. The results show an abnormal increase in the number of EPCs in post‐COVID‐19 patients compared to controls (82.8% vs. 48.4%, *p* < 0.01) that is maintained up to 6 months (87.0% vs. 48.4%, *p* < 0.01) and 12 months postinfection (85.0% vs. 48.4%, *p* < 0.01). There was no difference in EPCs production in post‐COVID‐19 patients who suffered an acute PE compared to those who did not. Interestingly, post‐COVID‐19 patients showed a significant downregulation of angiogenesis‐related proteins compared to controls indicating a clear endothelial injury. Troponin, NT‐proBNP, and ferritin levels, markers of cardiovascular risk and inflammation remained elevated up to 12 months postinfection. Patients with lower numbers of EPCs exhibited higher levels of inflammatory markers, such as ferritin, suggesting that EPCs may play a protective role. Additionally, the presence of long‐COVID syndrome was associated with higher ferritin levels and with female gender. The results confirm the presence of a long‐term vascular sequela in post‐COVID‐19 patients that persists up to 12 months after infection and point out the need for preventive measures and patient follow‐up.

## A124 PREDICTED DLCO, ADJUSTED FOR FERRITIN—FUTURE OF ANALYSIS OF GAS EXCHANGE IN PATIENTS WITH PULMONARY HYPERTENSION?

Serhii Progonov^1^, Olena Torbas^2^, Yuriy Sirenko^3^

^1^SI “NSC “Institute of Cardiology, Clinical and Regenerative Medicine n. a. Academician M.D. Strazhesko” of the National Academy of Medical Sciences of Ukraine, Kyiv, Ukraine, ^2^SI “NSC “Institute of Cardiology, Clinical and Regenerative Medicine n. a. Academician M.D. Strazhesko” of the National Academy of Medical Sciences of Ukraine, Kyiv, Ukraine, ^3^SI “NSC “Institute of Cardiology, Clinical and Regenerative Medicine n. a. Academician M.D. Strazhesko” of the National Academy of Medical Sciences of Ukraine, Kyiv, Ukraine

Diffusing capacity for carbon monoxide (DLCO) is a standard measurement in patients with pulmonary hypertension (PH) which allows the detection of pathological changes in transferring O2 and CO2. But this parameter depends on different other factors, including hemoglobin (Hb) level. That is why recent guidelines propose the use of DLCO standardized for Hb. Unfortunately, blood Hb can cause errors in the assessment of anemia in PH patients, therefore, we routinely determine the ferritin level. Consequently, our aim was to determine the lung function and DLCO changes in patients of 1 and 4 groups of PH without previously diagnosed lung disease and to evaluate whether the standardization of DLCO by blood ferritin has additional diagnostic value. We included 99 patients: 67 patients with diagnosed PH of 1 and 4 groups, 21 patients with arterial hypertension (AH), and 11 healthy controls. All groups were statistically comparable by age (PH group: 54.61 ± 1.78; AH group: 54.64 ± 3.03; control group: 49.73 ± 4.24), height (PH group: 16.66 ± 1.15; AH group: 172.41 ± 2.01; control group: 168.45 ± 2.04), weight (PH group: 83.39 ± 2.48; AH group: 95.36 ± 4.81; control group: 84.27 ± 6.45), and body mass index (PH group: 29.76 ± 0.92; AH group: 31.96 ± 1.26; control group: 29.44 ± 1.76). We did pulmonary function tests (PFTs), echocardiography, a 6‐min walking test (6MWT), and DLCO measurements. Right heart catheterization (RHC) was done only for PH patients. Calculation of DLCO, adjusted for Hb was done by standard formula. We used this by modifying the formula for DLCO, adjusted for ferritin. In the case of reducing the number of indicators that are defining obstructive changes (FEV1, MEF 75, MEF 50, MEF 25), we additionally performed a bronchodilatation test with inhaled salbutamol to further distinguish their reversibility or irreversibility. We used independent sample test t‐criteria to evaluate differences between groups and Spearman correlation analysis to evaluate associations. Patients with PH had lower parameters of pulmonary function compared to those with AH – lower VC (80.48 ± 2.6 vs 93.33 ± 4.35, *p* < 0,05), FEV1 (75.10 ± 2.38 vs 91.64 ± 4.44, *p* < 0.05), PEF (59.97 ± 2.64 vs 73.36 ± 5,98, *p* < 0,05), MEF 50 (64.39 ± 2.901 vs 83.68 ± 6.548, *p* < 0.05) and MEF 25 (68.25 ± 4.931 vs 97.53 ± 11.136, *p* < 0.05). DLCO was statistically lower for patients with PH than for patients with AH (49.43 ± 2.62 vs 76.65 ± 3.3, *p* < 0.001). Also, next parameters in healthy controls met our expectations and were significantly higher than in patients with PH – VC (103.09 ± 4.56 vs 80.48 ± 2.6, *p* < 0.001), FEV1 (98.09 ± 6.19 vs 75.10 ± 2.38, *p* < 0.05), MEF 25 (92.36 ± 7.71 vs 68.25 ± 4.931, *p* < 0.05), DLCO (89.0 ± 5.53 vs 49.43 ± 2.62, *p* < 0.001). There was no reliable difference of measurements obtained as result of PFTs after performed bronchodilatation tests with inhaled salbutamol. Levels of predicted DLCO, adjusted for ferritin were inversely correlated to levels of triscupid annular plane systolic excursion (TAPSE) measured during echocardiography (*r* = −0.410, *p* < 0.05) in group of patients with PH but no correlation of TAPSE with DLCO adjusted for hemoglobin was found. Other associations were not significant. Predicted DLCO, adjusted for ferritin, needs further investigation to clarify its potential significance for more accurate reflection of pathological changes in gas exchange processes of patients with pulmonary hypertension.

## A125 MOLECULAR CLASSIFICATION AND PROGNOSIS OF SYSTEMIC LUPUS ERYTHEMATOSUS‐ASSOCIATED PULMONARY ARTERIAL HYPERTENSION BASED ON RARE VARIANTS IN PAH RISK GENES

Junyan Qian^1^, Xianzhuang Yang^2^, Yufang Ding^1^, Qian Wang^1^, Jiuliang Zhao^1^, Liu Yongtai^3^, Zhuang Tian^3^, Yanhong Wang^4^, Mengtao Li^1^, Xiaojian Wang^5^, Xiaofeng Zeng^1^

^1^Department of Rheumatology and Clinical Immunology, Peking Union Medical College Hospital, Peking Union Medical College & Chinese Academy of Medical Sciences, National Clinical Research Center for Dermatologic and Immunologic Diseases, Ministry of Science & Technology, Key Laboratory of Rheumatology and Clinical Immunology, Ministry of Education, State Key Laboratory of Complex Severe and Rare Diseases, Ministry of Science & Technology, Beijing, China, ^2^Medical Research Center, State Key Laboratory of Complex Severe and Rare Diseases, Peking Union Medical College Hospital, Chinese Academy of Medical Sciences and Peking Union Medical College, Beijing, China, ^3^Department of Cardiology, Peking Union Medical College Hospital, Peking Union Medical College & Chinese Academy of Medical Sciences, Beijing, China, ^4^Department of Epidemiology and Bio‐Statistics, Institute of Basic Medical Sciences, China Academy of Medical Sciences & Peking Union Medical College, China, ^5^Key Laboratory of Pulmonary Vascular Medicine, State Key Laboratory of Cardiovascular Disease, Center for Respiratory and Pulmonary Vascular Diseases, National Center for Cardiovascular Diseases, Fuwai Hospital, Chinese Academy of Medical Sciences and Peking Union Medical College, Beijing, China

Pulmonary arterial hypertension (PAH) is severe complication of systemic lupus erythematosus (SLE). However, the effect of rare variants in PAH genes on clinical phenotype and outcomes of SLE‐associated PAH has not been studied. Two hundred thirty‐seven patients with SLE‐associated PAH confirmed by right heart catheterization from the multicenter prospective CSTAR‐PAH cohort were included and analyzed for whole‐exome sequencing. Deleterious rare variants among 28 PAH risk genes, including BMPR2, EIF2AK4, TBX4, ATP13A3, GDF2, SOX17, AQP1, ACVRL1, SMAD9, ENG, KCNK3, CAV1, SMAD4, SMAD1, KLF2, BMPR1B, KCNA5, KDR, FBLN2, PDGFD, PTGIS, BMP10, ABCA3, ABCC8, GGCX, KLK1, TopBP1, and SMAD5 were annotated. The primary outcome was all‐cause mortality. Reaching a low‐risk profile of PAH was the secondary outcome. Over all, PAH rare variants were detected in 51 (21.5%) patients with SLE‐associated PAH. The top identified rare variants including ABCA3 (seven patients, 2.95%), FBLN2 (six patients, 2.53%), TOPBP1 (five patients, 2.11%), ABCC8 (five patients, 2.11%), GGCX (five patients, 2.11%), and BMPR2 (four patients, 1.69%). SLE‐associated PAH patients carrying PAH rare variants had shorter PAH duration from SLE onset (3.0 ± 3.1 vs. 5.3 ± 5.2 years, *p* = 0.004), lower rate of serositis (11.8% vs. 29.6%, *p* = 0.010), lupus nephritis (17.6% vs. 34.4%, *p* = 0.022), and positive anti‐U1 RNP antibodies (45.1% vs. 65.6%, *p* = 0.008), and lower SLE disease activity index (2.31 ± 2.02 vs. 5.03 ± 2.81, *p* = 0.047). During a mean follow‐up of 2.65 ± 2.24 years, 15 (6.3%) deaths occurred, and 136 (57.4%) patients reached the low‐risk profile of PAH. After adjusting confounding factors (age, gender, and baseline PAH risk stratification), carrying PAH rare variants was identified as an independent prognostic factor of mortality (HR = 3.18, 95% CI, 1.12–9.02, *p* = 0.030) and reaching low‐risk profile of PAH (HR = 0.40, 95% CI, 0.28–0.90, *p* = 0.020). Furthermore, a significant interaction was found between carrying PAH rare variants and baseline serositis on the prognosis. Among patients without PAH rare variants, baseline serositis was identified as a protective prognostic factor of reaching low‐risk profile of PAH (HR = 1.55, 95% CI, 1.07–2.26, *p* = 0.021). This is the first study investigating deleterious rare variants of PAH risk genes in SLE‐associated PAH. Analyzing PAH rare variants helped the molecular classification of SLE‐associated PAH in distinguishing “vasculopathy”(carrier) and “vasculitis”(noncarrier). Carrying PAH rare variants also independently predicted the long‐term outcomes of SLE‐associated PAH.

## A126 THE INTRICACIES OF LUNG MICROCIRCULATION: UNRAVELING THE ROLE OF CAPILLARY ENDOTHELIAL CELLS IN PULMONARY HYPERTENSION

Olga Rafikova^1^, Joel James^1^, Aleksandr Dekan^1^, Ruslan Rafikov^1^

^1^Indiana University, Indianapolis, USA

Pulmonary hypertension (PH) is a complex and multifaceted condition that has been the subject of intense research. One less explored aspect of PH is lung microcirculation and capillary endothelial cells' role in its development and progression. This area is pivotal in ensuring optimal lung function and, as recent studies suggest, may be crucially implicated in the pathophysiology of PH. A significant leap in understanding the microcirculatory environment and cell‐to‐cell interactions within the lung was achieved by establishing single‐cell transcriptomics (scRNAseq). This technology allowed for identifying molecularly distinct aerocytes and general capillary (gCaps) endothelial cells. However, the lung capillary system's intrinsic complexity means there may still be numerous undiscovered cellular heterogeneities within this structure. In our endeavor to delve deeper into this system, we utilized scRNAseq to probe CD31‐enriched lung endothelial cells. Remarkably, we identified five novel gCaps populations, each with its unique molecular signature and functional role. Two of these populations express Scn7a(Na+) and Clic4(Cl−) ion transporters, forming the arterial‐to‐vein zonation and playing a crucial role in establishing the capillary barrier. Additionally, we unveiled the existence of mitotically‐active “root” cells, marked by Flot1+. Positioned strategically at the interface between the arterial, Scn7a+, and Clic4+ endothelium, these cells are pivotal in regenerating and repairing adjacent endothelial populations. We discovered that the transition of gCaps to a vein necessitates a specific venous‐capillary endothelium expressing Lingo2. Notably, gCaps detached from the zonation exhibited high levels of Fabp4 and other metabolically active genes, along with markers indicative of Tip cells, thereby suggesting a regulatory role in angiogenesis. To elucidate the changes each cellular population undergoes in the context of pulmonary hypertension (PH), we employed two pre‐clinical models—the metabolically driven PH induced by an NFU1 mutation and the Sugen/Hypoxia (Su/Hx) model. As expected, the changes in transcriptomic profiles in response to PH phenotype were diverse across the endothelial populations. However, both preclinical models demonstrated the onset of senescence and a pronounced loss of aerocytes. In the NFU1 model, the senescent phenotype was linked to mitochondrial dysfunction. Contrarily, in the Su/Hx model, aerocytes, which expressed higher levels of VEGFR2 compared to other endothelial populations, emerged as the primary targets for VEGFR2 inhibition by Sugen. Our findings highlight the intricate dynamics of the lung capillary system and emphasize the potential pivotal role of capillary cells in PH initiation and progression. Although understudied, capillary endothelium could represent the central player in the disease pathology.

## A127 THE PIVOTAL ROLE OF MITOCHONDRIAL ENZYMES IN THE ONSET OF PULMONARY ARTERIAL HYPERTENSION

Olga Rafikova^1^, Mathews Varghese^1^, Joel James^1^, Maki Niihori^1^, Ruslan Rafikov^1^

^1^Indiana University, Indianapolis, USA

Mitochondrial dysfunction (MD) is tightly associated with the pathobiology of pulmonary arterial hypertension (PAH), although the molecular mechanisms initiating are diverse. Previously, we discovered the severe in rats with a human NFU1G206C mutation, which compromises iron‐sulfur cluster stability and inhibits mitochondrial complex II (Cx‐II) and pyruvate dehydrogenase (PDH) activity, leading to spontaneous PAH phenotype and activation of the glycolytic pathway. Although this glycolytic shift is viewed as a compensatory mechanism, the increased glucose influx reported in the lungs of PAH patients could also initiate MD. However, the molecular determinants of this crosstalk between glycolytic switch and are not well established. We proposed that an increased glucose influx inhibits mitochondrial function and initiates PAH. To study the effect of an increased glucose uptake, we used the mice overexpressing glucose transporter GLUT4. The mice were characterized through assessment of pulmonary hemodynamics, histopathological examination, metabolomics, and lung protein expression profile. Isolated pulmonary artery smooth muscle cells (PASMCs) served to investigate the levels of glucose uptake and mitochondrial bioenergetics. Overexpression of human GLUT4 in mice was sufficient to spontaneously develop PAH phenotype (RVSP 24.98 ± 0.13 vs. 28.98 ± 0.16 mmHg, *p* < 0.001, and Fulton index 0.25 ± 0.03 vs. 0.32 ± 0.02, *p* < 0.0001 in Controls vs. GLUT4). GLUT4 overexpression has also increased glucose uptake and elevated levels of hexokinase‐1 and glucose‐6 phosphate compared to non‐transgenic controls. Metabolomics analysis of pulmonary tissue revealed dysregulation of the pentose phosphate pathway and fatty acid oxidation. Most importantly, GLUT4 mice showed a reduced expression of CX‐II and PDH subunits (SDHA and PDHA1) in the lungs, similar to the NFU1 model. Bioenergetic analysis of PASMCs confirmed the presence of significant in GLUT4 mice, characterized by decreased ATP production, basal respiration, and maximal respiration, and subsequent upregulation of proliferative effectors, such as Akt and PKC. Despite the different molecular mechanisms of induction in NFU1 and GLUT4 models, both exhibited a spontaneous PAH phenotype linked to the reduced expression or activity of mitochondrial enzymes Cx‐II and PDH. We conclude that the insufficiency of these enzymes is a crucial molecular determinant of PAH that bridges glycolytic shift with MD, triggering the onset of downstream proliferative signaling.

## A128 SMC‐DERIVED PCSK9 INDUCES PULMONARY VASCULAR DYSFUNCTION IN VITRO IN PULMONARY HYPERTENSION

Nabham Rai^1^, Baktybek Kojonazarov^2^, Ralph Schermuly^1^

^1^Excellence Cluster Cardio Pulmonary Insititute, Justus Liebig University, Giessen, Germany, ^2^Institute for lung health, Giessen, Germany

Proprotein convertase subtilisin/kexin type 9 (PCSK9) regulates LDL (low‐density lipoprotein)‐cholesterol via hepatic LDL‐receptor inhibition. PCSK9 also contributes directly to the pathogenesis of a number of diseases independent of the PCSK9‐LDLR axis. Pulmonary hypertension (PH)(PH) is a disease characterized by pulmonary vascular remodeling and increased pulmonary vascular load, leading to right ventricular hypertrophy and, ultimately, right heart failure. We sought to understand how PCSK9 interacts with pulmonary artery endothelial and smooth muscle cells to promote pulmonary vascular dysfunction in PH. Expression analysis in lungs by immunohistochemistry and in pulmonary arterial smooth muscle cells (PASMCs) from PAH patients was studied using RNA‐sequencing, Western blot analysis, and immunofluorescence. PCSK9 concentrations in cell culture supernatants were measured using the human PCSK9 ELISA kit. We demonstrated that the expression of PCSK9 was markedly upregulated in the lungs and pulmonary arterial smooth muscle cells of patients with clinical PH. Elevated PCSK9 gene expression is noted in PASMCs. Interestingly, it was observed that silencing PCSK9 in PASMCs downregulated the proliferative markers such as cyclin D1 and proliferating cell nuclear antigen (PCNA) but also suppressed the inflammatory response. Stimulation with pro‐proliferative growth factors such as Platelet‐derived growth factor (PDGF) as well as oxidative stress marker, ox‐LDL (oxidized low‐density lipoprotein) induced PCSK9 expression while its deletion prevented the proliferation of PASMCs. Ox‐LDL not only increased intracellular PCSK9 but also mediated its extracellular release as evidenced by PCSK9 ELISA. Next, the effect of PASMC‐released PCSK9 was studied on pulmonary artery endothelial cells (PAECs). PAECs cultured in SMC‐PCSK9‐conditioned medium demonstrated an active ERK/β‐catenin pathway as well as enhanced cell proliferation, whereas PCSK9‐depleted conditioned medium alleviated these effects. Similar effects were recapitulated when PAECs were incubated with recombinant human PCSK9 protein. Several studies have suggested LDL‐R‐independent effects of PCSK9 in cardiovascular disease. It is unclear whether these effects are caused by circulating PCSK9 originating from the liver or by PCSK9 acting locally in the cell types affected. Here, we propose that PASMC‐derived PCSK9 may contribute to the activation of quiescent endothelial cells into a hyper‐proliferative state. Thus, targeting intracellular vascular PCSK9 and its release might be an attractive future therapeutic strategy for PH.

## A129 METABOLOMIC ASSESSMENT OF CHRONIC PE COMPARED TO RESOLVED PE DURING REST AND PEAK EXERCISE

Michael Risbano^1,2,3^, Alyssa Kelder^1^, Luiz Campedelli^2^, Belinda Rivera‐Lebron^1,2^, Stephen Chan^1,3,4^

^1^Department of Internal Medicine, University of Pittsburgh School of Medicine and UPMC, Pittsburgh, USA, ^2^Division of Pulmonary, Allergy, and Critical Care Medicine, Department of Medicine, University of Pittsburgh School of Medicine and UPMC, Pittsburgh, USA, ^3^Center for Pulmonary Vascular Biology and Medicine, Pittsburgh Heart, Lung, Blood Vascular Medicine Institute, University of Pittsburgh School of Medicine and UPMC, Pittsburgh, USA, ^4^Division of Cardiology, Department of Medicine, University of Pittsburgh School of Medicine and UPMC, Pittsburgh, USA

Chronic pulmonary embolism (CPE) may occur after acute pulmonary embolism (PE)1,2. Often patients experience ongoing dyspnea after the diagnosis of PE. Evaluation with invasive cardiopulmonary exercise testing (iCPET) can identify etiologies of exercise intolerance including resting pulmonary hypertension (CTEPH), exercise pulmonary hypertension (ePH), or increased dead space during exercise (both CTEPD)3–5. The molecular drivers of these conditions, particularly metabolomics, are currently not well investigated. No studies have compared peak exercise metabolomics obtained during iCPET in patients with CPE to resolved PE (RPE) as a control group. The coupling of plasma metabolomics with invasive cardiopulmonary exercise testing (iCPET) physiology may produce a deeper physiological phenotyping of CPE. We hypothesized that the unbiased metabolomic analysis of CPE compared to RPE can identify molecular pathways unique to CPE and identify potential therapeutic molecular targets for this under‐studied disease. We performed a retrospective analysis of 44 patients diagnosed with acute PE by chest CT angiogram who underwent evaluation for persistent dyspnea on exertion with ventilation/perfusion (VQ) scan and referred for iCPET. Patients performed a peak exercise upright iCPET with a goal respiratory exchange ratio (RER) > 1.05 or peak heart rate >90% predicted6. Normal peak oxygen consumption (V̇O2peak) was defined as >80% predicted. Twenty‐eight CPE and 16 RPE patients underwent a peak exercise iCPET study. We performed unbiased metabolomic testing (Metabolon) on paired blood samples obtained from the right atrium at rest and peak exercise in all subjects, and compared rest to peak exercise samples between CPE and RPE subjects. Metabolomic analysis utilized multiple separation techniques by mass spectrometer identification of specific metabolites. Groups were compared by ANOVA to determine if the means of the two populations were different. Raw data was Log transformed. CPE rest to peak values demonstrated 70 metabolites that were significantly downregulated and 429 metabolites that were significantly upregulated. RPE rest to peak values demonstrated 50 metabolites that were significantly downregulated and 128 metabolites that were significantly upregulated. Interestingly, CPE had significantly elevated levels of fibrinopeptides at peak exercise (i.e., fibrinopeptide A) compared to RPE. Overall, we found that rest‐to‐peak exercise CPE patients have significantly different shifts in pathways related energetics (mild abnormalities in the Cori and Cahill cycle; branched chain amino acids). Furthermore, we identified consistent elevations in complex lipid metabolism (phosphatidyl‐choline, plasmalogen, and sphingomyelins) in CPE and RPE patients from rest to peak exercise. Circulating cholesterols were observed to have a similar trend, suggesting that the lipid changes are due to a change in lipoprotein composition in the blood, which can be associated with thrombotic events. This global metabolic profiling study was conducted to identify metabolic signatures associated with CPE and its interaction with exercise and response to exercise. These pathways are interconnected through a variety of means, such as systemic energy regulation, and may each play a distinct role in promoting persistent thrombus formation.

## A130 RIOCIGUAT IN PULMONARY ARTERIAL HYPERTENSION: APPLICATION OF THE 4‐STRATA COMPERA 2.0 RISK ASSESSMENT TOOL IN THE PATENT STUDIES

Stephan Rosenkranz^1^, Marius M. Hoeper^2^, David B. Badesch^3^, Marc Humbert^4^, David Langleben^5^, John W. McConnell^6^, Claudia Rahner^7^, Hossein‐Ardeschir Ghofrani^8^

^1^Department of Cardiology – Internal Medicine III, Cologne University Heart Center, Cologne, Germany; Cologne Cardiovascular Research Center (CCRC), Cologne University Heart Center, Cologne, Germany, ^2^Clinic for Respiratory Medicine, Hannover Medical School, member of the German Center for Lung Research (DZL), Hannover, Germany, ^3^School of Medicine, Anschutz Medical Campus, University of Colorado, Colorado, USA, ^4^Université Paris‐Saclay, Faculty of Medicine, Le Kremlin‐Bicêtre, France; INSERM UMR_S 999, Pulmonary Hypertension: Pathophysiology and Novel Therapies, Hôpital Marie Lannelongue, Le Plessis Robinson, France; AP‐HP, Department of Respiratory and Intensive Care Medicine, Pulmonary Hypertension National Referral Centre, Hôpital Bicêtre, DMU 5 Thorinno, Le Kremlin‐Bicêtre, France, ^5^Center for Pulmonary Vascular Disease, Division of Cardiology, Jewish General Hospital, McGill University, Montreal, Canada, ^6^Norton Health Care, Louisville, Kentucky, USA, ^7^CHRESTOS Concept GmbH & Co. KG, Essen, Germany, ^8^University of Giessen and Marburg Lung Center, member of DZL, Giessen, Germany; Department of Pneumology, Kerckhoff‐Klinik, Bad Nauheim, Germany; Department of Medicine, Imperial College London, London, UK

The 2022 ESC/ERS treatment guidelines for pulmonary arterial hypertension (PAH) recommend using a four‐strata risk assessment strategy at follow‐up. COMPERA 2.0 is a refined four‐strata risk assessment tool that subdivides patients at intermediate risk of mortality at 1 year into intermediate‐low and intermediate‐high risk groups for a more granular approach to risk prediction. Aim: This post hoc analysis applied COMPERA 2.0 to the PATENT studies of riociguat in patients with PAH. COMPERA 2.0 was applied to patients who were pretreated with PAH therapy at PATENT‐1 entry. Patients with missing data for COMPERA 2.0 variables were excluded. In PATENT‐1, risk strata were assessed at baseline and Week 12, and 6‐min walk distance (6MWD) at Week 12 was analyzed by risk strata at baseline. All pretreated patients entering the PATENT‐2 open‐label extension were included in Kaplan–Meier analyses to assess association of risk strata at PATENT‐1 baseline and Week 12 with clinical worsening and survival. At PATENT‐1 baseline (riociguat 2.5 mg *n* = 102; placebo *n* = 49), more patients were at intermediate‐low risk (riociguat 49%; placebo 49%) than intermediate‐high risk (riociguat 28%; placebo 33%) or low risk (riociguat 23%; placebo 18%). At PATENT‐1 Week 12, a higher proportion of patients were at low risk with riociguat (38%) vs placebo (27%). Also, at PATENT‐1 Week 12, 40% vs 45% were at intermediate‐low risk, 22% vs 27% were at intermediate‐high risk, and 0% vs 2% were at high risk with riociguat vs placebo, respectively. At PATENT‐1 Week 12, the following mean [SD] changes in 6MWD were seen in patients at intermediate‐low risk (riociguat 24 m [57 m], placebo 10 m [60 m]) and intermediate‐high risk (riociguat 34 m [56 m], placebo −2 m [70 m]) by COMPERA 2.0 at baseline. COMPERA 2.0 assessed at PATENT‐1 baseline, and Week 12 was able to discriminate between risk strata for clinical worsening and survival in PATENT‐2 (*n* = 167, log‐rank tests: *p* ≤ 0.001 for all analyses). This analysis confirmed the risk‐reduction benefits of riociguat in patients with PAH and externally validated the utility of COMPERA 2.0 in the long‐term risk assessment of patients from a clinical trial population.

## A131 THE IMPACT OF RIOCIGUAT IN PULMONARY ARTERIAL HYPERTENSION PATIENTS WITH COMORBIDITIES INCLUDED IN INTERVENTIONAL CLINICAL TRIALS

Stephan Rosenkranz^1^, Hossein‐Ardeschir Ghofrani^2^, Marius M. Hoeper^3^, Vallerie V. McLaughlin^4^, Claudia Rahner^5^, David Langleben^6^

^1^Department of Cardiology – Internal Medicine III, Cologne University Heart Center, Cologne, Germany; Cologne Cardiovascular Research Center (CCRC), Cologne University Heart Center, Cologne, Germany, ^2^University of Giessen and Marburg Lung Center, member of DZL, Giessen, Germany; Department of Pneumology, Kerckhoff‐Klinik, Bad Nauheim, Germany; Department of Medicine, Imperial College London, London, UK, ^3^Clinic for Respiratory Medicine, Hannover Medical School, member of the German Center for Lung Research (DZL), Hannover, Germany, ^4^Department of Internal Medicine, Division of Cardiology, Frankel Cardiovascular Center University of Michigan Medical School, Ann Arbor, Michigan, USA, ^5^CHRESTOS Concept GmbH & Co. KG, Essen, Germany, ^6^Center for Pulmonary Vascular Disease, Division of Cardiology, Jewish General Hospital, McGill University, Montreal, Canada

The number of pulmonary arterial hypertension (PAH) patients with comorbidities is increasing, however, there are few published data from these patients in PAH therapy clinical trials meaning that evidence‐based treatment recommendations have not been made. Aim: This post hoc analysis of pooled riociguat clinical trial data assessed the impact of riociguat in patients with PAH and comorbidities. All interventional studies of riociguat in patients with PAH (PATENT‐1, PATENT PLUS, RESPITE, MOTION, REPLACE, and the Phase II study) were included in the pooled analysis. The safety of riociguat and placebo was assessed in patients with ≥3 comorbidities vs <3 comorbidities. Safety was assessed by adverse events (AEs) and serious AEs (SAEs). Comorbidities included: age ≥65 years, body mass index ≥30 kg/m^2^, history of essential hypertension, diabetes mellitus, history of significant coronary artery disease, and atrial fibrillation. The 609 patients who received riociguat and 132 patients who received placebo in the main phase clinical trials of riociguat were included in the analysis. Of these, 113 patients (19%) in the riociguat group and 21 patients (16%) in the placebo group had ≥3 comorbidities. In both treatment groups, AE rates were higher in patients with ≥3 comorbidities vs <3 comorbidities (riociguat 93% vs 86%; placebo 95% vs 84%), with most AEs being mild or moderate. Study drug‐related AE rates were similar in both treatment groups and in patients with ≥3 comorbidities vs <3 comorbidities (riociguat 54% vs 56%; placebo 52% vs 52%). SAE rates were higher in riociguat‐treated patients with ≥3 comorbidities vs <3 comorbidities (25% vs 12%); of these, 4% and 3% were study drug‐related, respectively. In patients with ≥3 comorbidities, study drug‐related SAE rates were higher in those receiving placebo vs riociguat (10% vs 4%). AE rates leading to study drug discontinuation were higher in patients with ≥3 comorbidities vs <3 comorbidities (riociguat 6% vs 4%; placebo 10% vs 6%), whereas AE rates leading to death were similar in both groups (riociguat 2% vs 1%; placebo 0% vs 4%). These data suggest that riociguat had an acceptable tolerability profile in PAH patients with comorbidities with most AEs being mild or moderate.

## A132 A REDOX SWITCH IN CYCLIN d‐CDK4 INHIBITS KINASE ACTIVITY TO REGULATE PULMONARY VASCULAR CELL PROLIFERATION AND ALLEVIATE PULMONARY HYPERTENSION

Hannah Knight^1^, Giancarlo Abis^2^, Manpreet Kaur^1^, Hannah Green^1^, Susanne Krasemann^3^, Kristin Hartmann^3^, Steven Lynham^1^, James Clark^1^, Lan Zhao^4^, Clemens Ruppert^5^, Astrid Weiss^6^, Ralph Schermuly^6^, Philip Eaton^7^, Olena Rudyk^1^

^1^King's College London, School of Cardiovascular and Metabolic Medicine & Sciences, London, UK, ^2^University College London, Institute of Structural and Molecular Biology, Division of Biosciences, London, UK, ^3^University Medical Centre Hamburg‐Eppendorf, Institute of Neuropathology, Hamburg, Germany, ^4^Imperial College London, Faculty of Medicine, National Heart and Lung Institute, London, UK, ^5^ Justus‐Liebig‐University Giessen, Universities of Giessen & Marburg Lung Center Giessen Biobank, Giessen, Germany, ^6^Justus‐Liebig‐University Giessen, Member of the German Center for Lung Research (DZL), Department of Internal Medicine, Giessen, Germany, ^7^Queen Mary University of London, Barts & The London School of Medicine & Dentistry, William Harvey Research Institute, London, UK

Pulmonary vascular remodeling is the prominent structural hallmark of pulmonary hypertension (PH) that involves changes in all vessel wall layers. It is characterized, amongst other abnormalities, by hyperproliferative smooth muscle and endothelial cells and a perturbed cellular redox and metabolic balance. Oxidants halt cell proliferation through cell cycle arrest; however, little is known about the redox‐regulated effector proteins that mediate these processes. Oxidants such as hydrogen peroxide can post‐translationally regulate proteins that contain redox‐active cysteine residues, and many of these redox switches remain to be identified. Here, we report a novel kinase‐inhibitory disulfide bond in cyclin d‐CDK4 that forms in human pulmonary arterial smooth muscle cells in vitro and pulmonary arteries in vivo and investigate its functional role in vascular cell proliferation and PH. By employing cysteine mutants, cell‐based experiments, tandem mass‐spectrometry, in silico structural modelling tools, and in vitro kinase activity assays, we identified that this reversible disulfide bond forms between C7/8 cyclin D and C135 CDK4 to inhibit phosphorylation of the tumor suppressor protein, retinoblastoma, and induce cell cycle arrest. Importantly, this disulfide bond forms at a critical cysteine residue, meaning mutation of C135 CDK4 is sufficient to impair kinase activity and attenuate cell proliferation. As a result, CDK4 C135A mutation causes haploid HAP1, primary pulmonary arterial smooth muscle or endothelial cells to proliferate at slower rates, consistent with C135 being indispensable for kinase activity. Corroborating this finding, novel ‘redox‐dead’ constitutive cysteine to alanine mutant, C135A CDK4 knock‐in male and female mice develop a less severe PH phenotype and less pronounced pulmonary vascular muscularization compared with the wild‐type littermates in the experimental Sugen/hypoxia PH model. Furthermore, it was found that the abundance of cyclin d‐CDK4 disulfide is decreased in pulmonary arteries or cultured pulmonary arterial smooth muscle cells from patients with idiopathic pulmonary arterial hypertension (PAH). This is consistent with fully active CDK4 kinase contributing to disease pathogenesis and may account, at least partially, for the previously reported hyperactivity of CDK4 in PAH. Finally, a pharmacological thioredoxin reductase inhibitor, auranofin, was employed to investigate whether this kinase‐inhibitory cyclin d‐CDK4 disulfide offers protection in experimental PH. It was found that auranofin induced the cyclin d‐CDK4 disulfide accumulation in mouse pulmonary arteries and attenuated hemodynamic measures of disease severity (assessed by right ventricular pressure and hypertrophy and noninvasive echocardiographic imaging) and decreased vascular remodeling (measured by immunohistochemical staining of α‐smooth muscle actin) in three experimental models of PH. To conclude, a novel disulfide bond in cyclin d‐CDK4 represents a rapid redox switch evolved to inhibit kinase activity and halt cell proliferation. C135 CDK4 is a novel regulatory site in CDK4 that may provide the opportunity to selectively target kinase using a covalent inhibitor that is predicted to be beneficial in PH.

## A133 POTENTIAL PHYSIOLOGICAL AND PATHOLOGICAL ROLES FOR ENDOTHELIAL SENESCENCE IN PULMONARY ARTERIAL HYPERTENSION: IMPLICATIONS FOR TIMING OF SENOTHERAPIES

Elmira Safaie Qamsari^1,2^, Nicholas D Cober^1,2^, Duncan J Stewart^1,2^

^1^Ottawa Hospital Research Institute, Sinclair Centre for Regenerative Medicine Program, Ottawa, Canada, ^2^University of Ottawa, Faculty of Medicine, Department of Cellular and Molecular Medicine, Ottawa, Canada

Senescence is a cellular state characterized by irreversible cell cycle arrest and the secretion of pro‐inflammatory and tissue‐remodeling factors. Accumulation of senescent cells (SCs) has recently been implicated in the pathogenesis of pulmonary arterial hypertension (PAH), which is a chronic disease of the pre‐capillary pulmonary arterioles, characterised by endothelial cell (EC) injury, inflammation, and proliferative remodelling. Recent studies have implicated senescence in PAH; however, the specific role of senescent endothelial cells (SECs) in the context of both tissue repair and chronic inflammation remains poorly understood. Indeed, there is conflicting data whether eliminating SCs using senolytic drugs is beneficial or harmful in PAH models. Our hypothesis is that SCs play a dual role in PAH. In the early stages of the disease, they significantly contribute to vascular repair. However, in later stages, they promote inflammation and occlusive vascular remodeling. While targeting SCs with senotherapies may hold potential for reversing PAH, this approach would require optimal timing to strike the right balance between promoting adaptive vascular repair and mitigating potential harmful effects. We characterized the accumulation of SCs at various time points during the progression of PAH in Sugen‐hypoxia (SU/CH) rat model. In brief, rats received a single Subcutaneous injection of the VEGFR inhibitor, SU5416 (SU 20 mg) and were exposed to hypoxia (10% oxygen) for 3 weeks. Analysis of a single cell transcriptomic data set showed an increase in senescence markers appearing as early as 1 week and persisting to 8 weeks restricted to a population of ‘activated’ arterial endothelial cells that were highly localized to regions of arterial remodeling in PAH. Next, we performed flow cytometry at different timepoints to assess senescence‐associated ß‐galactosidase activity using the Senescence ß ‐Galactosidase Activity Assay commercial Kit, together with immunofluorescence (IF) staining for other senescent markers such as P53 and confirmed a significant increase in SECs in PAH in comparison of healthy lung tissues. We then studied the effect of a senolytic drug, ABT‐263, given at early or late stages of disease and saw benefit only with delivery after 3 weeks, consistent with the possibility that clearance of SECs soon after EC injury might hinder repair, while late administration may be beneficial by reducing the pro‐inflammatory and pro‐proliferative actions of SECs. PAH is lethal disease, and the impact of current therapies on disease progression and survival remains uncertain. In this study, we elucidated the specific role of SCs during PAH onset and progression in the SU/CH rat model, focusing mainly on the EC populations. We found evidence of EC senescence beginning surprisingly early in disease, consistent with an initial adaptive role of CS in endothelial repair. However, SECs persisted to the late stages of PAH, at which time the administration of senotherapies was effective in reducing the severity of disease. Finding the optimal window for implementing therapeutic interventions is fundamental in developing strategies to mitigate the adverse effects associated with SCs accumulation in PAH.

## A134 THE SULFATASE‐1‐NEUROPILIN‐1 AXIS UNDERLIES PULMONARY ARTERIAL HYPERTENSION PATHOBIOLOGY

Andriy Samokhin^1^, Progyaparamita Saha^1^, Bradley Maron^1^

^1^University Of Maryland, Baltimore, Baltimore, USA

Sulfatase‐1 (SULF1) is an extracellular enzyme that removes 6‐O‐sulfate (6‐O‐S) groups from heparan sulfate (HS). In turn, the removal of 6‐O‐S from cell surface HS‐proteoglycans leads to the release of growth factors, including VEGF‐A, that have been linked to pathogenetic cardiovascular effects. We demonstrated recently that SULF1 is upregulated in pulmonary vessels of patients with pulmonary arterial hypertension (PAH) and that molecular inhibition of SULF1 prevents severe pulmonary hypertension in two experimental disease rodent models in vivo. The proteoglycan Neuropilin‐1 (NRP‐1) is expressed on the pulmonary artery endothelial cell (PAEC) membrane, identified as a potential PAH biomarker, and functions as a co‐receptor of angiogenic VEGFR2. However, the molecular mechanisms that regulate NRP‐1 in PAH are not known. We hypothesized that in PAH, increased SULF1 controls pulmonary endothelial NRP1 by affecting the 6‐O‐S modification of its attached HS to induce a proangiogenic phenotype. Control donor HPAECs were analyzed by immuno‐electron microscopy (IEM) to study co‐localization of NRP1 with 6‐O‐S HS in vitro. In an inflammatory model of PAH, male rats were administered a single intraperitoneal injection of monocrotaline (MCT) (50 mg/kg). In an angioproliferative model of PAH, male rats were administered SU‐5416 (20 mg/kg) and exposed to hypoxia for 21 days. Lungs were analyzed by immunohistochemistry to quantitate SULF1, NRP1, the endothelial marker CD31, and 6‐O‐S HS. The proximity ligation assay was used to profile colocalization of NRP1 and 6‐O‐S HS. Human lung specimens were either isolated from patients with PAH at the time of explant or discarded lung tissue from donor controls without PAH, and analyzed similarly. Compared to controls, distal pulmonary arterioles from PAH patients expressed increased NRP1 (4.6 ± 1.2 vs 16 ± 7.3 arb. units, *N* = 5–8/condition, *p* < 0.05), which was co‐localized with CD31, suggesting NRP1 upregulation in HPAECs. Directionally similar results for NRP1 expression were observed compared to controls for MCT‐PAH and SU‐5416/Hypoxia‐PAH (17 ± 2.9 vs. 28 ± 7.3 vs. 48 ± 11 arb. units, *N* = 5/condition, *p* < 0.05). Furthermore, we observed that expression of 6‐O‐S HS was decreased significantly in the MCT‐ and SU‐5416/Hypoxia‐PAH (9.3 ± 2.6 vs. 3.0 ± 1.8 vs. 1.6 ± 0.9 arb. units, *N* = 5/condition, *p* < 0.05) compared to controls, as was the signal ratio of 6‐O‐S HS to Sulf1 (3.5 ± 0.3 vs. 1.8 ± 0.6 vs 1.2 ± 0.3 arb. units, *N* = 5/condition, *p* < 0.05). In HPAECs, IEM confirmed proximity of the signals for NRP1 and 6‐O‐S HS, which was supported further by proximity ligation assay experiments showing a 93% (*N* = 5, *p* < 0.01) and 93% (*N* = 5, *p* < 0.01) decrease in 6‐O‐S HS expression on NRP1 in MCT‐PAH and SU‐5416/Hypoxia‐PAH, respectively, compared to controls. Remodeled PAH pulmonary arterioles overexpress SULF1 and NRP1, which is associated with decreased 6‐O‐S HS on NRP1. These data implicate SULF1 in the pathogenesis of PAH through a molecular mechanism that involves pulmonary endothelial SULF1‐mediated 6‐O‐S removal from HS of NRP‐1. Further experiments are warranted to quantitate the functional effects of this molecular mechanism on the angioproliferative potential of pulmonary arterioles in PAH.

## A135 PERIVASCULAR RADIOMIC MARKERS FROM CT SCANS: A NOVEL APPROACH FOR CHARACTERIZING PULMONARY HYPERTENSION

Maria Rollán Martinez Herrera^1^, Pietro Nardelli^1^, Farbod Rahaghi^2^, Raul San Jose Estepar^1^

^1^Dept. of Radiology, Brigham And Women's Hospital, Harvard Medical School, Boston, USA, ^2^Division of Pulmonary and Critical Care, Brigham and Women's Hospital, Harvard Medical School, Boston, USA

Inflammation is increasingly recognized as a pivotal component of PH, playing a vital role in initiating and perpetuating vascular remodeling. Specifically, perivascular inflammation in PH patients comprises T and B lymphocytes, macrophages, dendritic cells, and mast cells (1). While CT imaging is effective for assessing vascular pruning and remodeling, there are no established methods to define and quantify peri‐vascular imaging markers. Our objective is to identify unique signatures characterizing the perivascular inflammatory process using radiomics on CT scans, potentially establishing novel imaging markers of PH associated with perivascular endotypes. Pulmonary arteries were extracted and classified from CT scans using the Chest Imaging Platform. The perivascular space was defined as a 2 mm thick concentric region around vessels with a cross‐sectional area less than 5 mm_2_. A total of 985 radiomic features were extracted from the perivascular space using PyRadiomics. In a case‐control design, we employed the Boruta method for supervised perivascular radiomic feature selection. The associations between perivascular radiomics markers and hemodynamics and imaging metrics of PH were explored using Spearman's rank correlations. A diagnostic machine learning model was subsequently trained using these features, with an XGBoost classifier and a 2/3 to 1/3 training‐to‐testing split. A cohort of 145 patients, referred for pulmonary vascular disease consultation and having chest CT scans from Brigham and Women's Hospital, was analyzed. The cohort consisted of 37 controls and 106 PH patients (45 Group 1, 40 Group 2, and 21 Group 4). Eight perivascular radiomic markers were selected, of which five were textural descriptors and three were first‐order moments. Top perivascular radiomics descriptors correlated with mPAP, PVR, PA:A, and BV5/TBV at coefficients of ‐0.48 (*p* < 0.00001), −0.48 (*p* < 0.0001), −0.56 (*p* < 0.0011), and 0.5 (*p* < 0.001) respectively. The PH classifier, developed using the selected perivascular markers, yielded an AUC of 0.81 (CI 95% 0.68–0.94). This study successfully demonstrated the potential of perivascular radiomic markers extracted from CT scans in characterizing pulmonary vascular disease, specifically pulmonary hypertension (PH). Among the 985 radiomic features considered, eight significant perivascular radiomic markers were identified, with strong correlations observed between top descriptors and critical hemodynamic and imaging metrics of PH. Importantly, the diagnostic machine learning model, constructed using these selected markers, achieved a high accuracy with an AUC of 0.81. These findings underscore the potential of leveraging radiomic features from the perivascular space in CT scans as noninvasive biomarkers for PH diagnosis and prognosis.

## A136 BMP9‐MEDIATED ET‐1 TRANSCRIPTION REQUIRES DUAL ACTIVATION OF SMAD1/5/9 AND SMAD2/3

Shreya Sangam^1^, Jana Bagarova^1^, Samuel Paskin‐ Flerlage^1^, Ivana Nikolic^1^, Fernando Rodriguez‐ Pascual^2^, Paul Yu^1^

^1^Division of Cardiology, Department of Medicine, Massachusetts General Hospital, Harvard Medical School, Boston, USA, ^2^Centro de Biología Molecular “Severo Ochoa” Consejo Superior de Investigaciones Cientificas/Universidad Autonoma de Madrid, Madrid, Spain

Dysregulated bone morphogenetic protein (BMP) and transforming growth factor‐β (TGF‐β) signaling are implicated in heritable pulmonary arterial hypertension (HPAH), based on loss‐of‐function mutations in BMPR2, ACVRL1/ALK1, ENG, and GDF2/BMP9. BMP9 is a circulating ligand recognized by the BMPR2/ALK1/ENG signaling complex with potent effects in modulating endothelial cell (EC) function. BMP9 activates both SMAD1/5/9 (BMP‐associated) and SMAD2/3 (TGFβ‐associated) signaling pathways, but it is unknown if its EC functions require engagement one or both pathways. Recent studies demonstrate a pathophysiologic role of BMP9 in experimental PH due in part to regulation of EDN1/ENDOTHELIN‐1 (ET‐1), a potent vasoconstrictive peptide that contributes to vascular tone, cell proliferation, and fibrotic remodeling in PAH. We investigated the transcriptional regulatory mechanisms of ET‐1 via BMP9 to ascertain whether its function requires activation of SMAD1/5/9, SMAD2/3, or both. Human primary cultured pulmonary artery endothelial cells (PAEC) and pulmonary microvascular endothelial cells (PMVEC), PMVECs from wild‐type, conditional Acvrl1 or Bmpr2 knockout (KO) mice, and bovine aortic endothelial cells (BAEC) were used for examining transcriptional regulation of ET‐1, using truncated and full‐length sequences of the ET‐1 promoter. Signaling was tested using selective small molecule inhibitors of BMP versus TGF‐β signaling, and siRNAs specific for various BMP and TGF‐β type I, II, and III receptors (ACVRL1, ALK5, ENG, BMPR2, and TGFBR2). SMAD1/5/9 and SMAD2/3 signaling were assessed by immunoblot, and gene expression was examined by reporter assays and ELISA. Both BMP9 and TGFβ1 potently upregulated ET‐1 mRNA and protein expression in human PMVEC and BAEC. BMP9‐induced activation of SMAD1/5/9 and SMAD2/3. Inhibition of either BMP or TGFβ type I receptor kinases in ECs using small molecule inhibitors LDN‐193189 or SB‐431542 abrogated BMP9‐induced ET‐1 transcription. SiRNA for BMPR2, ACVRL1, or ENG but not ALK5 or TGFBR2 suppressed BMP9‐mediated ET‐1 expression in ECs, as did siRNA for SMAD3, or SMAD3 inhibitor SIS3. Similarly, PMVEC isolated from Alk1 or Bmpr2 KO mice failed to activate SMAD1 or SMAD3, or express ET‐1 in response to BMP9. Analysis of the ET‐1 promoter revealed unique requirements for BMP9 activity beyond AP‐1, including SMAD3/4 binding sites previously shown to be required for TGFβ1‐mediated activity, an additional putative NF‐1 binding site conferring enhanced sensitivity to BMP9. We dissected the mechanism involving the transcriptional regulation of human ET‐1 promoter in response to BMP9. These data demonstrate that BMP9 requires the coordinated activation of BMP and TGF‐β signaling effectors, and in particular SMAD3 to regulate gene expression of ET‐1 in endothelium. The potent regulation of ET‐1 by BMP9 and SMAD3 signaling suggest that the pathogenetic effects of BMP9 are mediated by canonical TGFβ effector molecules, with potentially important consequences for efforts to balance BMP and TGFβ signaling therapeutically in PAH.

## A137 PULMONARY ARTERY ANEURISMS: EPIDEMIOLOGY AND INFLUENCE ON SURVIVAL IN PULMONARY ARTERIAL HYPERTENSION

Dra María Ascensión Sanroman Guerrero^1^, Dra Irene Martín de Miguel, Alejandro Cruz Utrilla, Álvaro Cantero Acedo, Dra Maria Teresa Velázquez Martín, Jorge Nuche, Dra Yolanda Revilla, Sergio Alonso Chanterina, Dra Maria Pilar Escribano Subías

^1^Hospital 12 Octubre, Madrid, Spain

Pulmonary arterial hypertension (PAH) is a rare and heterogeneous disease characterized by the remodelling of small pulmonary vessels, increasing the pulmonary vascular resistance, and rising the right ventricle overload. The introduction of novel therapies has notably increased the survival, converting this otherwise lethal disease into a chronic one. Pulmonary artery aneurism (PAA) is defined as a dilatation of the main pulmonary artery (PA) above 40 mm. The increase in the PA pressure is associated with its dilatation over time. Consequently, this condition is frequently present at diagnosis in PAH, being additionally a marker of chronicity. PAA diagnosis has increased parallelly to the better awareness of PAH, but specially with longer follow‐up periods in this rare disease. The aim of this study is: (1) To describe the prevalence and the associated aetiology of PAA. (2) To determine the influence of having a PAA in the global survival. We selected a cohort of 434 patients with PAH followed up in a single reference centre for pulmonary hypertension from December 2010 to December 2022. Patients were selected if, at least one high‐definition cardiac magnetic resonance or a computed tomography imaging in where pulmonary artery diameter could be measured was available. Baseline clinical and haemodynamical characteristics, as well as global survival, were compared between those cases in which a PAA was present with those patients without PAA. The cumulative incidence of mortality was estimated with the Kaplan‐Meier method according to the presence of PAA and the difference was assessed with the log‐rank test. A total of 434 patients were included (median age 46.0 ± 16.0 and Women 66.8%). We identified the presence of a PAA in 138 patients (31.8%). PAA was more frequent in patients with PAH related to congenital heart diseases (CHD) and in idiopathic forms (28.3% and 31.2%, respectively). In the PAA cohort, 59.0% of the sample was classified as intermediate risk, whereas 15.0% as high risk. We observed differences in the haemodynamic profile between both groups (mPAP in patients with PAA was 58.5 +/− 15.7 mmHg vs 51 +/− 15.0; *p* < 0.0005. PVR was 12.6 UW +/− 6.8 Vs 11.0 +/− 6.3; *p* < 0.0005). Twenty‐six patients (18.8%) with PAA suffered at least one complication associated with the aneurism, such as pulmonary artery dissection, thrombosis or left main coronary artery compression. In the survival analysis, we observed that patients with PAA had a better survival than patients without PAA (139 vs 118 months; Long Rank test *p* < 0.05). PAA is frequent in patients with PAH, especially in idiopathic PAH and PAH‐CHD. A higher haemodynamic severity was observed in those patients with PAA, without any difference according to the baseline European risk stratification system. PAA is related with a better global survival according to this observational study, which could be explained by a longer follow‐up (Immortal Time Bias). Further investigation is necessary to verify the real impact of PAA on survival, and to evaluate possible confounding factors influencing this outcome.

## A138 THE ROLE OF SMYD2/RUNX2 AXIS IN RIGHT VENTRICULAR HYPERTROPHY AND FAILURE

Vandna Sapehia^1^

^1^JLU Giessen, Giessen, Germany

Pulmonary hypertension (PH) is a life‐threatening disease, characterized by excessive pulmonary vascular remodeling, leading to elevated pulmonary arterial pressure and right ventricular hypertrophy (RVH). The RV is the major determinant of functional state and prognosis in PH. The RVH triggered by pressure overload is initially compensatory but ultimately leads to RV failure. The mechanisms underpinning the development of RV failure are still unexplored. Recent studies demonstrated that an osteogenic transcription factor Runt‐related transcription factor 2 (RUNX2) plays a pathogenic role in cardiac hypertrophy and failure, and also contributes to the development of PH. In this study, we hypothesize the existence of the interplay of the methyltransferase SMYD2 (SET and MYND domain‐containing protein 2) and the RUNX2 in RVH. The impact of gene silencing and overexpression studies of RUNX2 and SMYD2 over one another was analyzed by using primary cardiac fibroblasts (CFs) by employing adenovirus‐mediated and si‐RNA‐mediated overexpression and gene silencing approaches, respectively. In loss‐of‐function studies, RUNX2 silencing reduced CF proliferation and promoted the downregulation of mRNA expression of osteogenic genes as Col1α1, Col3α1, Cysteine‐rich acidic matrix‐associated protein (SPARC), alkaline phosphatase (ALP) and Cartilage intermediate layer protein (CILP). Notably, SMYD2 knockdown provided opposite effects in CFs. In our experiments, we observed the negative effects of SMYD2 ectopic expression on RUNX2 protein accumulation in CFs. Furthermore, SMYD2 significantly impeded the expression of osteogenic markers such as Col1α1, Col3α1, SPARC, ALP, and CILP both in human and rat CFs. We also discovered that SMYD2 negatively affects SMAD2/3 protein expression. Thus, the main conclusion of our study is that SMYD2 plays a negative role in osteogenesis by suppressing the osteogenic gene program and via inhibiting the expression of RUNX2. We will continue to explore the mechanisms by which SMYD2 might contribute to osteogenesis and investigate whether RUNX2 serves as a specific therapeutic target for RVH and failure.

## A139 MACROPHAGE‐SPECIFIC FOSL2: A NOVEL IMMUNOMODULATOR IN LUNG CANCER‐ASSOCIATED PULMONARY HYPERTENSION

Poonam Sarode^1,2^, Spyridoula Barboutsi^1,2^, Carsten Kuenne^2^, Stefan Günther^2^, Rajender Nandigama^1,2^, Clemens Ruppert^3^, Natascha Sommer^3^, Friedrich Grimminger^1,3^, Werner Seeger^1,2,3^, Soni Savai Pullamsetti^1,2,3^, Rajkumar Savai^1,2,3^

^1^Institute for Lung Health (ILH), Justus Liebig University, Germany, ^2^Max Planck Institute for Heart and Lung Research, Member of the German Center for Lung Research (DZL), Member of the Cardio‐Pulmonary Institute (CPI), Germany, ^3^Department of Internal Medicine, Member of the DZL, Member of CPI, Justus Liebig University, Germany

Tumor‐associated macrophages (TAMs) have been shown to contribute significantly to the pathogenesis of lung cancer‐associated pulmonary hypertension (LC‐PH). Multiplex immunofluorescence staining of lung sections from LC‐PH patients revealed that pulmonary vessel‐associated macrophages (PVAMs) have dual macrophage phenotypic properties (M1 ≈ M2). These findings were reproduced in a newly developed in vitro pulmonary vessel‐associated macrophages (PVAM) model and cellular functional assays (smooth muscle cells and fibroblasts’ proliferation, migration, and apoptosis using conditioned medium from in vitro trained M1‐/M2‐like TAMs, cytokine‐stimulated M1/M2 macrophages). Therefore, to investigate similarities between genome‐wide transcription factor (TF) binding kinetics in M1‐ and M2‐like macrophages, we performed an ATAC‐seq of in vitro‐trained TAMs, followed by TOBIAS analysis. Of the top 50 TFs, 28 were more bound in the genome of both macrophage phenotype. Based on the binding‐site overlap, TFs belong to five different TF clusters – BCL11A, CEBPA/G, DBP, FLI1, and FOSL2. Of these five TFs, mRNA and protein expression of FOSL2 was found to be significantly increased cytokine‐stimulated M1/M2 macrophages, in vitro trained M1‐like, M2‐like TAMs, and PVAFOSL2‐overexpressing mice develop pulmonary remodeling, although selective deletion of FOSL2 in structural cells has little effect on the pathogenic phenotype of vascular cells. Interestingly, the conditioned medium of FOSL2‐deficient macrophages reduced proliferation and migration and markedly increased apoptosis of both idiopathic pulmonary vascular cells and lung tumor cells. To identify macrophage‐specific FOSL2‐regulated molecular mechanisms, we performed siRNA‐mediated knockdown of FOSL2 in TAand PVAMs, followed by RNAseq. Knockdown of FOSL2 altered inflammation‐mediated cytokine and chemokine‐, apoptotic‐, CCKR‐ signaling, and T‐cell activation in macrophages associated with IPAH fibroblasts; EGFR, PDGF, FGF signaling in macrophages associated with IPAH smooth muscle cells; and PDGF, angiotensin‐II, and Wnt signaling in TANotably, these results showed that FOSL2 regulates inflammation, proliferation, extracellular matrix deposition, and thus remodeling, although its effect is context‐dependent. Together with members of the AP1 family, FOSL2 forms a broad network of homodimers and heterodimers that, when interacting with other TFs, can affect both redundant and differential expression of target genes. Therefore, we used TF‐COMB to investigate TFs coexisting with FOSL2 and binding grammar in the regulatory domain. TF‐COMB study of in vitro trained TAshowed that FOSL2 coexists with both common and unique TFs in macrophage phenotypes, suggesting that macrophage‐specific FOSL2 coexists different TFs in TAand PVAto control the microenvironment‐dependent immune response. Therefore, investigating the role of macrophage‐specific FOSL2 transcription in disease‐specific immunomodulation may pave a new way to precision medicine in the treatment of lung cancer‐associated pulmonary hypertension.

## A140 HS135, A NOVEL ACTIVIN AND GDF TRAP, IS HIGHLY EFFICACIOUS IN MODELS OF GROUP 1 AND GROUP 2 PULMONARY HYPERTENSION (PH)

Gauthier Schang^1^, Mathilde Poujol De Molliens^1^, Ariane Sours^1^, Emilie Brûlé^1^, Cristina Chauvet^1^, Jean‐Francois Denis^1^, Vann Ganesh^1^, Gilles Tremblay^1^, Julia Schoelermann^1^, Maureen O'Connor‐McCourt^1^

^1^35Pharma, Montreal, Canada

Pulmonary hypertension (PH) is associated with dysregulated activin and growth differentiation factor (GDF) signalling. The use of the unmodified activin receptor II A ectodomain fused to Fc (ActRIIA‐Fc, sotatercept) as a decoy trap to neutralize activins and GDFs has recently been clinically validated as a promising new therapeutic modality in Group 1 PH. Building on this validation, HS135 is an activin receptor‐based activin/GDF trap that was rationally designed to have an improved therapeutic window relative to ActRIIA‐Fc, and to achieve best‐in‐class in vivo target engagement and a differentiated efficacy profile. Importantly, HS135 was designed to avoid increases in hematocrit, which have been shown to be dose limiting for this class. Here, HS135 was explored in preclinical models of Group 1 and 2 PH. Efficacy in Group 1 and 2 PH was assessed using the rat monocrotaline (MCT) and the mouse transverse aortic constriction (TAC) models, respectively. In each case, HS135 was compared to ActRIIA‐Fc, and tissue remodelling in the heart and lungs was assessed by IHC and RNA‐seq. Administration of MCT caused dysregulation of pathways related to inflammation and energy balance in the right ventricle (RV), which were only modestly improved by ActRIIA‐Fc. In contrast, in HS135‐treated animals, these pathways were nearly normalized, i.e., were indistinguishable from naïve mice. In line with RV gene expression profiles, HS135 but not ActRIIA‐Fc completely rescued RV function. Similarly, in the lung, HS135 was more efficacious than ActRIIA‐Fc at improving inflammation markers and reducing vascular remodelling. In the TAC model of Group 2 PH, HS135, but not ActRIIA‐Fc, returned LV fibrosis and heart failure markers to baseline in a dose dependent fashion. Importantly, HS135 but not ActRII‐Fc completely reversed TAC‐induced lung remodelling including vein arterialization, a hallmark of Group 2 PH. The best‐in‐class target engagement profile of HS135 translates into superior and differentiated heart and lung efficacy in relevant Group 1 and 2 PH preclinical models, supporting its further development as a novel agent in PH.

## A141 THE IMPACT OF COMORBIDITIES ON INHALED TREPROSTINIL TREATMENT IN PATIENTS WITH PULMONARY ARTERIAL HYPERTENSION

Rahul Argula^1^, Karim El‐Kersh^2^, Shelley Shapiro^3^, Eric Shen^4^, Hyoshin Kim^5^, Meredith Broderick^4^, Vallerie McLaughlin^6^

^1^Medical University of South Carolina, Charleston, SC, USA ^2^University of Arizona College of Medicine, Phoenix, AZ, USA ^3^VA Greater Los Angeles Healthcare System and Division of Pulmonary Critical Care, David Geffen UCLA School of Medicine, Los Angeles, CA, USA ^4^United Therapeutics Corporation, Research Triangle Park, NC, USA ^5^North Carolina State University, Raleigh, NC, USA ^6^University of Michigan, Ann Arbor, MI, USA

Registry data show an increasing number of elderly patients with pulmonary arterial hypertension (PAH) with comorbidities, particularly hypertension, diabetes, coronary artery disease, and obesity. Effects of these comorbidities on response to PAH treatment are not well understood, and guidelines do not provide evidence‐based treatment recommendations in this population. The purpose of this post‐hoc analysis was to explore the effects of inhaled treprostinil (iTre) on PAH patients with cardiovascular comorbidities in the pivotal TRIUMPH study. Patients randomized in the TRIUMPH study (*n* = 235) were classified as either having 0, 1, or ≥2 cardiovascular comorbidities based on conditions listed in each patient's medical history. The comorbidities included were hypercholesterolemia, diabetes mellitus, coronary artery disease, atrial fibrillation, systemic hypertension, heart failure, smoking history, and obesity. The median difference in 6MWD change from baseline between iTre and placebo groups was determined by the Hodges‐Lehman estimate and the Wilcoxon rank sum test. No imputation was used. Adverse events (AEs) and study discontinuations due to AEs were summarized for each comorbidity group. There were 111, 68, and 56 patients with 0, 1, and ≥2 comorbidities, respectively. At Baseline, patients with comorbidities weighed more, had a lower 6MWD, and a higher NT‐proBNP compared to patients without comorbidities. In patients with 0 comorbidities, iTre had a placebo‐corrected change from baseline in 6MWD of 27.0 m (*p* = 0.005). In patients with 1 comorbidities, the placebo‐corrected change from baseline in 6MWD was 14.0 m (*p* = 0.300). In patients with ≥2 comorbidities, the placebo‐corrected change from baseline in 6MWD was 27.7 14 m (*p* = 0.043). Adverse events incidence was similar between groups, however, discontinuation of iTre due to AEs was more frequent in patients with comorbidities than those without comorbidities. The most common cardiopulmonary comorbidities in TRIUMPH were systemic hypertension, hypercholesterolemia, and diabetes mellitus. Although not all subgroups showed significant improvements in 6MWD, likely due to the small sample size, results suggest clinical benefit associated with inhaled treprostinil; 6MWD improvements in each subgroup were similar to the 20 m improvement seen in the original study. In conclusion, inhaled treprostinil improved exercise, irrespective of the presence of comorbidities.

## A142 MACHINE LEARNING MODEL FOR PREDICTION OF PULMONARY HYPERTENSION IN PATIENTS WITH IDIOPATHIC PULMONARY FIBROSIS

Toru Shirahata^1,2^, Pietro Nardelli^3^, Eileen Harder^4^, Sydney Montesi^5^, Sirus Jesudasen^5^, Badar Patel^6^, Raúl San José Estépar^1,3^, Farbod Rahaghi^1,4^

^1^Applied Chest Imaging Laboratory, Brigham and Women's Hospital, Harvard Medical School, Boston, USA, ^2^Department of Respiratory Medicine, Saitama Medical University Hospital, Iruma‐gun, Japan, ^3^Department of Radiology, Brigham and Women's Hospital, Harvard Medical School, Boston, USA, ^4^Division of Pulmonary and Critical Care Medicine, Department of Medicine, Brigham and Women's Hospital, Harvard Medical, Boston, USA, ^5^Department of Medicine, Massachusetts General Hospital, Harvard Medical School, Boston, USA, ^6^Department of Medicine, Beth Israel Deaconess Medical Center, Harvard Medical School, Boston, USA

Since pulmonary hypertension (PH) in patients with idiopathic pulmonary fibrosis (IPF) is associated with worse prognosis, early prediction of PH is critical for timely intervention and may improve prognosis. This study aims to establish and validate predictive machine learning model based on the patients' demographic and the three‐dimentional (3D) automated central computed tomography (CT) structures. We retrospectively studied 163 patients with IPF who underwent both thin‐sectioned chest CT imaging and right heart catheterization (RHC). A diagnosis of IPF was confirmed based on medical record, and CT scans were reviewed by a pulmonologist specialized in interstitial lung disease. 3D automated volumetry of cardiac chamber and pulmonary artery was obtained using the Chest Imaging Platform (www.chestimagingplatform.org). We also measured the pulmonary artery (PA) and ascending aorta (AA) diameters on chest CT. Random forest (RF) model and logistic regression (LR) model in Python (3.8.17) were created using 3D automated volumetry of cardiac chamber and pulmonary artery. The model's reliability was assessed by utilizing 10‐fold cross‐validation. We also utilized SHapley Additive exPlanation (SHAP) values to explain the prediction model. Out of 163 patients, 75 had PH (46.0%). The RF model achieved a higher AUC than the LR model (0.87 vs 0.82). SHAP value revealed pulmonary arterial volume and right atrial volume were important features. When the PA/A ratio was applied to the RF model instead of PA volume, the model's performance decreased (AUC; 0.87 vs 0.79). The RF model had a better discrimination ability of PH from non‐PH than the LR model. This automated 3D volume measurement has the potential to outperform conventional PA/A measurements in predicting PH. Since the model and segmentations are automated and thus can be incorporated into clinical practice.

## A143 POTENTIAL DIFFERENCES IN THE EXPRESSION OF EPIGENETIC WRITERS AND ERASERS IN THE PATHOBIOLOGY OF PULMONARY ARTERIAL HYPERTENSION BETWEEN ETHNIC GROUPS

Christina Signoretti^1^, Shun Matsumura, Samuel Fatehi, Sachin Gupte

^1^New York Medical College, Valhalla, USA

The loss of ten eleven translocation (TET2) methylcytosine deoxygenase expression contributes to the pathobiology of pulmonary arterial hypertension (PAH). However, whether the expression and activity of other TETs and DNA methyltransferases (DNMTs) is altered in PAH remains enigmatic. Therefore, we determined the expression of DNMT (1, 3a, and 3b) and TET (1, 2, and 3) and their total activity. We assessed the expression of DNMT and TET enzymes in leukocytes and their activity in extracellular vesicles (EVs). Expression of DNMT (1, 3a, and 3b), TET (2 and 3) in leukocytes, and total activity in EVs, from PAH patients was higher than in healthy controls. Additionally, we noticed there were differences in the expression of these epigenetic enzymes based on ethnicity and found higher DNMT1 and lower TET2/TET3 expression in Caucasian than Hispanic/African American (combined) patients. Since loss‐of‐function mutation(s) and downregulation of TET enzymes are associated with hematological malignancies and cytokine production, we determined the expression of genes that encode cytokines in samples of Caucasian and Hispanic/African American patients. Expression of IL6, CSF2, and CCL5 genes were higher in the leukocytes of Caucasian than Hispanic/African American patients, and CSF2 and CCL5 negatively correlated with the decreased expression of TET3. Further, Hispanic/African American patients having higher TET2/TET3 expression had higher pulmonary capillary wedge pressure. Recently, we have reported that DNMT and TET expression and activity are regulated by metabolic reprogramming in a glucose‐6‐phosphate dehydrogenase (G6PD)‐dependent manner. Therefore, to determine the mechanism through which G6PD induces expression and activity of epigenetic enzymes and determine the role of G6PD in mediating sugen‐5416 in DMSO/normoxia‐induced PAH, we recently developed two rat models of Mediterranean (S188F; G6PDS188F)‐ and African (N126D; G6PDN126D)‐specific loss‐of‐function polymorphism in G6PD that mimics the characteristics of human polymorphisms. Interestingly, the Mediterranean, but not the African, polymorphism increased TET and conversely decreased DNMT expression and reduced sugen‐5416/normoxia‐induced PAH. Conversely, the African polymorphism did not regulate epigenetic enzymes and intriguingly potentiated sugen‐5416‐induced PAH. In conclusion, our results revealed that ethnic G6PD variants potentially elicits differential expression and activity of epigenetic enzymes, and higher DNMT1 and lower TET2/TET3 in Caucasian than Hispanic/African American patients together potentially augmented genes encoding inflammation‐causing cytokines and the severity of PAH.

## A144 IMPACT OF ANESTHESIA ON ECHOCARDIOGRAPHIC ASSESSMENT OF RIGHT VENTRICULAR FUNCTION IN PEDIATRIC PATIENTS WITH PULMONARY ARTERIAL HYPERTENSION UNDERGOING CARDIAC CATHETERIZATION

Charles Simpkin^1^, Billy McElroy^2^, Mark Twite^2^, Gareth Morgan^1^, D. Dunbar Ivy^1^, Benjamin Frank^1^, Dale Burkett^1^

^1^Division of Cardiology, Department of Pediatrics, University of Colorado, Aurora, USA, ^2^Department of Anesthesiology, University of Colorado, Aurora, USA

One of the great diagnostic challenges for children with pulmonary arterial hypertension (PAH) is the need for general anesthesia during right heart catheterization (RHC). The risk of clinical decompensation during induction for these patients is high. Furthermore, positive pressure ventilation (PPV) and anesthetic medications are known to alter pulmonary vascular hemodynamics in ways that may make the results difficult to relate to the patient's unsedated state. Here, for the first time, we describe how anesthesia alters echocardiographically derived estimates right ventricular (RV) function and pulmonary pressures during RHC. Two echocardiograms were obtained in 12 pediatric patients with PAH: one prior (within 2 weeks) of routine RHC, and another obtained simultaneously during RHC under general anesthesia. Tricuspid annular plane systolic excursion (TAPSE), right ventricular end‐diastolic area (RVEDA), right ventricular fractional area change (RV FAC), Systolic Eccentricity Index (EIs), and the maximum velocity from the tricuspid regurgitation (TR Vmax) were measured. These were compared to invasively measured mean and systolic pulmonary artery pressure (mPAP, PASP), indexed pulmonary vascular resistance (PVRi), and the RV systolic pressure (RVSP). Systemic blood pressure was significantly lower in the post‐anesthesia condition (*p* < 0.005), while heart rate was unchanged. TAPSE, RVEDA, RV FAC, and EIs did not change significantly between conditions. TR Vmax was significantly lower in the post‐anesthesia condition (4.0 (3.2–4.8) m/s vs 3.23 (2.8–3.9) m/s, *Z* = −2.11, *p* < 0.05). Patients who had normal RV FAC pre‐anesthesia were noted to have a significant decrease in their RV FAC, whereas those with already abnormal function did not show a significant change in RV FAC (median change 12.98 (9.75–15.50) vs −4.46 (−9.44 to 5.20) *z* = −2.74 *p* < 0.001). The post‐anesthesia TR Vmax correlated well with invasively measured PASP (*r* = 0.921, *p* < 0.001), whereas the pre‐anesthesia TR Vmax did not (*r* = 0.504, *p* = 0.11). EIs in both conditions were strongly correlated to invasive hemodynamics (vs PVRi, *r* = 0.971 *p* < 0.001 and *r* = 0.951 *p* < 0.001). The hemodynamic implications of general anesthesia are complex—but are an important caveat to consider when interpreting the results of diagnostic RHC in children with PAH. Because obtaining invasive hemodynamics pre‐ and post‐anesthesia in children is not feasible, noninvasive techniques are needed to shed light on how anesthesia may impact the right ventricle. To our knowledge, this is the first study to do so. Our results suggest the myocardial depressant effects of PPV and anesthesia may be counterbalanced by a degree of pulmonary vasodilation—given that TAPSE and RV FAC did not change significantly, while TR Vmax decreased. There was no change in EIs between conditions, which is likely a reflection of significant decreases in the systemic vascular resistance, which can alter ventricular‐ventricular interactions. In a subgroup analysis, we noted patients with normal RV FAC before anesthesia had a significant decline in their function compared to those with already depressed function. This is similar to prior works in adults, but the exact mechanism for this phenomenon requires further study. Interestingly, EIs performed better than TR Vmax in the pre‐anesthesia condition at predicting invasively measure pulmonary artery pressure and resistance.

## A145 THE LIVER AS AN INFLAMMATORY MEDIATOR OF PULMONARY ARTERIAL HYPERTENSION

Navneet Singh^1^, Amy Princiotto^2^, David Silverberg^3^, Peng Zhang^1,2^, Corey Ventetuolo^1,4^, Elizabeth Harrington^1,2^

^1^Department of Medicine, Alpert Medical School at Brown University, Providence, USA, ^2^Vascular Research Laboratory, Providence Veterans Affairs Medical Center, Providence, USA, ^3^Department of Pathology and Laboratory Medicine, Alpert Medical School of Brown University, Providence, USA, ^4^Department of Health Services, Policy and Practice, Brown University, Providence, USA

The liver–lung axis remains poorly understood in pulmonary arterial hypertension. Inflammation is a long‐established hallmark of PAH including in subtypes characterized by chronic liver disease. Bone morphogenetic protein 9 (BMP9), encoded by growth differentiation factor 2 (GDF2) and primarily produced in the liver, is a critical ligand in PAH pathobiology and involved in systemic inflammation. BMP9 deficiency and GDF2 mutations that produce nonfunctional BMP9 have been observed in PAH. BMP9 repletion rescues experimental disease. We hypothesized that livers from a preclinical Sugen‐Hypoxia (SuHx) rat model of PAH have increased inflammatory infiltrate and decreased expression of GDF2 in comparison to controls. Livers were harvested from SuHx and control rats (*n* = 3 in each group) 7 weeks after PAH induction. The left lobe of the liver in each animal was separated, randomly sectioned, paraffin‐embedded, and stained with Hematoxylin and Eosin to assess for cellularity, Oil Red O for fatty deposition, and CD68 for M0 macrophages. The remainder of the liver (thus far, *n* = 1 in each group) was used to assess the mRNA levels of GDF2 and the protein levels of TNF‐α and IL‐6 via qPCR and ELISA approaches, respectively, per manufacturer protocol. Histology and immunohistochemistry showed increased average staining for CD68+ macrophages in SuHx livers (1.60% vs 0.93%, *p* = 0.003) with a nonsignificant trend toward increased fatty deposition (12.22% vs 4.86%, *p* = 0.31). SuHx liver homogenate had 4.18‐fold increased expression of GDF2 mRNA. TNF‐α expression was increased in SuHx liver homogenate compared to controls (363.4 pg/mL vs 190.77 pg/mL) but not IL‐6 (107.5 pg/mL vs 121.6 pg/mL). These data suggest an increased inflammatory infiltrate in SuHx rat livers. Increased hepatic GDF2 expression may be a compensatory response to decreased circulating functional BMP9, as has been observed in human PAH. Future directions will include increasing sample size to validate these observations, macrophage and cytokine phenotyping, quantifying BMP9 expression in the liver and plasma and performing protein activity assays. This work was completed with support from the National Institutes of Health T32HL134625 (NS), P20GM103652 (EOH), RO1HL141268 (CEV), and The CHEST Foundation (NS).

## A146 PULMONARY VASCULAR INVOLVEMENT IN POST‐COVID‐19 ASSOCIATED DYSPNEA—A MULTICENTER, OBSERVATIONAL STUDY

Khodr Tello^1^, Karsten Krüger^2^, Pascal Bauer^3^, Katrin Milger‐Kneidinger^4^, Christoph Tabeling^5^, Isabell Pink^6^, Gani Oruqaj^1^, None Kevin Lo^1^, Selin Yildiz^1^, Zvonimir Rako^1^, Matthias Hecker^1^, None Lea Laufer^2^, Ulrich Matt^7^, Susanne Herold^7^, None Maik Hahmann^8^, Martin Witzenrath^5^, Tobias Welte^6^, Werner Seeger^9^, Natascha Sommer^10^

^1^Medical Clinic II, Excellence Cluster Cardio‐Pulmonary Institute (CPI), Member of the German Center for Lung Research (DZL), Justus‐Liebig University of Giessen, Giessen, Germany, ^2^Department of Exercise Physiology and Sports Therapy, Institute of Sports Science, Justus‐Liebig University Giessen, Giessen, Germany, ^3^Department of Cardiology and Angiology, Justus‐Liebig University Giessen, Giessen, Germany, ^4^Department of Internal Medicine V, Ludwig‐Maximilian University Munich, Member of the German Center for Lung Research (DZL), Comprehensive Pneumology Center Munich (CPC‐M), Munich, Germany, ^5^Division of Pulmonary Inflammation, Department of Infectious Diseases and Respiratory Medicine, Charité ‐ Universitätsmedizin Berlin, Corporate Member of Freie Universität Berlin and Humboldt‐Universität zu Berlin, Berlin, Germany, ^6^Department of Pulmonary and Infectious Diseases, Hannover Medical School, BREATH German Center for Lung Research (DZL), Hannover University School, Hanover, Germany, ^7^Department of Medicine V‐Infectious Diseases, Excellence Cluster Cardio‐Pulmonary Institute (CPI), Member of the German Center for Lung Research (DZL), Justus‐Liebig University, Giessen, Germany, ^8^Coordination Center for Clinical Studies (KKS), Member of the German Center for Lung Research (DZL), Philipps‐University Marburg and Justus‐Liebig University Giessen, Marburg, Germany, ^9^Medical Clinic II, Excellence Cluster Cardio‐Pulmonary Institute (CPI), Member of the German Center for Lung Research (DZL), Institute for Lung Health (ILH), Justus‐Liebig University of Giessen, Giessen, Germany, ^10^Excellence Cluster Cardio‐Pulmonary Institute (CPI), Universities of Giessen and Marburg Lung Center (UGMLC), Member of the German Center for Lung Research (DZL), Justus‐Liebig University of Giessen, Giessen, Germany,

Currently, the causes of exertional dyspnea and exercise limitation in patients with post‐COVID‐19 syndrome remain unclear. Due to the vascular tropism of SARS‐CoV‐2 and high frequency of right heart insufficiency during acute COVID‐19, we hypothesized that post‐acute sequelae of pulmonary vascular and right heart dysfunction may contribute to post‐COVID‐19 symptoms. We performed 2D and 3D right heart echocardiography during exercise, as well as estimation of ventilation‐perfusion mismatch (V/Q mismatch) by the automatic lung parameter estimator (ALPE) system (Mermaid Care A/S, Denmark) in a cohort of 60 post‐COVID‐19 patients (approx. one year after acute infection). Lung functional and exercise parameters were additionally tested by body plethysmography and cardiopulmonary exercise testing (CPET). Patients showed mild limitations of peak oxygen uptake (VO2peak 86+/‐20% pred, mean+/‐SD) and diffusing capacity of the lungs for carbon monoxide (DLCO 74+/‐13% pred), however, normal CO transfer coefficient (DLCO/VA 84+/‐13/pred). Echocardiographically, we did not detect signs of right heart dysfunction or pulmonary hypertension (PH) at rest or during exercise. ALPE measurement and CPET indicated mild V/Q mismatch and ventilatory inefficiency, respectively. Low DLCO was associated with worse static and dynamic lung functional and cardiac parameters. High dyspnea index in the total cohort was associated with low VO2peak, high heart rate and low left ventricular function at rest. In patients reaching the predicted cardiopulmonary and/or metabolic range, high dyspnea index was associated with a low anaerobic threshold and diffusion capacity. V/Q inhomogeneities resulting in low diffusion capacity may contribute to exertional dyspnea in specific subgroups of post‐COVID‐19 patients. We did not detect signs of PH in our cohort. Heterogeneity of post‐COVID‐19 patients needs further phenotyping of larger cohorts.

## A147 MUTATIONS IN GCN2 CAUSE PULMONARY VASCULAR DISEASE VIA A DYSFUNCTIONAL INFLAMMATORY EFFECT

Max Schwiening^1^, Stephen Moore^1^, Alexi Crosby^1^, Mark Southwood^1^, Christopher Huang^1^, Roger Thompson^2^, Nicholas Morrell^1^, Stefan Marciniak^1^, Elaine Soon^1^

^1^University of Cambridge, Cambridge, UK, ^2^University of Sheffield, Sheffield, UK

Mutations in eukaryotic translation initiation factor 2 alpha kinase 4 (EIF2AK4, or General Control Nonderepressible 2 [GCN2]) cause deadly forms of pulmonary hypertension such as pulmonary veno‐occlusive disease (PVOD). GCN2 is a serine/threonine protein kinase, one of a family of four kinases that phosphorylate the α‐subunit of the translation initiation factor eIF2, which activates a common adaptive pathway known as the Integrated Stress Response. The only definitive treatment is a lung transplant, but these are rare opportunities due to the paucity of organ supply. Consequently, the median survival for these young patients is only 1.5 years post‐diagnosis, which is comparable to many cancers. We used mice homozygous for a large deletion in exon 12 of GCN2 (gcn2−/−). Initially, we phenotyped the gcn2−/− mouse at baseline and created two independent mouse models of PVOD—the first using an inflammatory driver (lipopolysaccharide) on a background of genetic gcn2 loss, and the second using mitomycin‐c (a chemotherapeutic agent which has an idiosyncratic side‐effect of causing PVOD in patients). GCN2 deficiency causes a spontaneous mild pulmonary hypertensive phenotype, with a right ventricular systolic pressure (RVSP) of 28.1(±3.4) mmHg in gcn2−/− mice versus 24.7(±3.7) mmHg in the wild‐type at baseline (*p* = 0.007). Exposure to acute LPS creates a higher level of IL‐6 and KC in the lungs of gcn2−/− mice. Chronic administration of LPS exaggerates the RVSP response in gcn2‐deficient mice (32.6 ± 4.3 mmHg) but not in the wild‐type (23.3 ± 3.4 mmHg, *p* = 0.002). Genetic ablation of IL‐6 ameliorated the development of pulmonary hypertension in both baseline and stressed states, with the gcn2−/−il6−/− mouse having a mean RVSP of 20.4 (±0.6) mmHg despite chronic exposure to LPS. Administration of mitomycin‐c raises the RVSP from 19.23(±4.3) to 26.6(±3.8) mmHg in wild‐type mice (*p* = 0.007) but not in the IL6−/− mouse. Single‐cell RNA sequencing of wild‐type and gcn2‐deficient mouse lungs has allowed us to identify the specific cell‐types responsible for the inflammatory phenotype. Serum samples from patients with GCN2‐mutation‐positive PVOD confirm increased IL‐6 levels compared to healthy volunteers. Proteomics studies have shown that there is a heightened inflammatory response in GCN2‐mutation‐positive PVOD compared to healthy volunteers and that aspects of this response may differ from other kinds of mutation‐driven pulmonary vascular disease. Mutations in GCN2 cause a hyper‐inflammatory phenotype and a baseline pulmonary hypertensive phenotype which is worsened by persistent inflammation. Genetic deletion of IL‐6 ameliorates the pulmonary vascular disease in two independent mouse models of disease. We propose that IL‐6 is a central pathway for the pathogenesis of heritable pulmonary veno‐occlusive disease and anti‐IL6 therapies may be clinically useful in treating PVOD patients.

## A148 UTILITY AND PITFALLS OF CARDIOPULMONARY EXERCISE TESTING DIAGNOSIS OF PULMONARY HYPERTENSION IN THE LONG COVID ERA

Johanna Squires^1^, Sarra Al‐Zayer^1^, David Systrom^1^

^1^Pulmonary and Critical Care Medicine, Brigham and Women's Hospital, Boston, USA

Precapillary pulmonary hypertension (PH) and Long Covid (LC) often present with dyspnea upon exertion. Ventilatory inefficiency, reflected by an elevated minute ventilation/carbon dioxide production slope (VE/VCO2 > 30, Mezzani et al. (2017)) is a feature of PH (Weatherald et al. (2021)) and LC (Singh et al. (2022). This study attempts to highlight exercise pathophysiologic differences between the two disorders using the ventilatory and hemodynamic results from invasive cardiopulmonary exercise testing (iCPET). Between January 2020 and July 2023, 960 patients underwent clinical invasive cardiopulmonary exercise tests (iCPETs) at Brigham and Women's Hospital in Boston, MA. Exclusion criteria included morbid obesity (BMI > 40 kg/m^2^), anemia (Hgb < 9.0), elite athletes (peak VO2 > 120% predicted), submaximum effort (RER < 1.05), a primary pulmonary mechanical limit (VE @ AT/MVV > 0.7), and comorbidities such as active/treated cancer, interstitial lung disease, or other respiratory related diseases. CPET results from 36 PH patients (all groups) and 57 LC patients were analyzed. Peak exercise VO2, cardiac output (Qc), arterial blood gases, dead space to tidal volume ratio (VD/VT), end‐tidal CO_2_ (PETCO2), mean pulmonary arterial pressure (mPAP), right atrial pressure (pRAP), and pulmonary capillary wedge pressure (PCWP) were compared between both groups. Both groups demonstrated elevated VE/VCO2 (PH: 38.8 ± 10.7, LC: 31.3 ± 6.0, *p* < 0.001), need *p*‐value compared to published normals and peak VO2 was similarly reduced (PH: 69.2 ± 19.0% predicted, LC: 75.8 ± 18.2% predicted, *p* = 0.095), and there were no significant differences seen between groups for ETCO2 or PaCO2 (*p* > 0.05). iCPET revealed significant differences in peak arterial pH (PH: 7.40 ± 0.04, LC: 7.37 ± 0.04, *p* < 0.05), PA‐aO2 (PH: 38.0 ± 20.4 mmHg, LC: 8.0 ± 10.2 mmHg, *p* < 0.001), VD/VT (PH: 0.32 ± 0.11, LC: 0.18 ± 0.08, *p* < 0.001), Qc (PH: 78.6 ± 24.4% predicted, LC: 88.8 ± 19.0%, *p* = 0.027), RAP (PH: 6.7 ± 5.5 mmHg, LC: 1.2 ± 3.2 mmHg, *p* < 0.001), PCWP (PH: 13.8 ± 8.2 mmHg, LC: 6.3 ± 4.5 mmHg, *p* < 0.001), and peak PVR (PH: 293.4 ± 156.2 mmHg*min/L, LC: 94.8 ± 32.8 mmHg*min/L, *p* < 0.001). Reduced aerobic capacity and ventilatory inefficiency are common to both PH and LC. Invasive CPET, however, reveals profoundly different pulmonary hemodynamics and gas exchange. Ventilatory inefficiency is driven by hyperventilation in both groups, but disproportionately in PH by dead space ventilation. This study highlights the utility and pitfalls of noninvasive CPET in screening for PH in the Long COVID era.

## A149 AI‐BASED QUANTIFICATION OF PULMONARY VASCULAR CHANGES AFTER BALLOON PULMONARY ANGIOPLASTY: “THE BP‐AI STUDY”

Diederik Staal^1^, Leticia Gallardo‐Estrella^2^, M C J Van Thor^1^, Sanne Boerman^1^, J P Charbonnier^1^, J J Mager^1^, H A W M Tiddens^2^, M C Post^1,3^

^1^St. Antonius Hospital, Nieuwegein, The Netherlands, ^2^Thirona b.v., Nijmegen, The Netherlands, ^3^University Medical Centre Utrecht, Utrecht, The Netherlands

Chronic thromboembolic pulmonary hypertension (CTEPH) and chronic thromboembolic pulmonary disease (CTEPD) are rare complications of pulmonary embolisms. Balloon pulmonary angioplasty (BPA) is a proven endovascular therapy for patients with inoperable CTEPH and, to a lesser extent, in CTEPD. Artificial intelligence‐based pulmonary artery‐vein phenotyping (AVX) provides in‐depth characterization of the pulmonary vasculature on chest computed tomography (CT). This study is the first to evaluate pulmonary artery‐vein volume redistribution in relation to clinical outcomes in CTEPH/CTEPD patients treated with BPA. This retrospective study analyzed CT scans of inoperable CTEPH/CTEPD patients treated with BPA. CTEPH/CTEPD diagnosis and BPA treatment were assessed by a multidisciplinary CTEPH team according to the available guidelines at the time. One patient was considered a poor responder and one a good responder to BPA at 12‐month follow‐up following evaluation of clinical parameters. Following each BPA sessions, a chest CT was performed and analyzed using LungQ‐AVX (Thirona, Nijmegen, The Netherlands) to assess pulmonary arterial volume distribution partitioned into small arteries volume (artery diameter ≤2 mm, AVXSA) and large arteries volume (artery diameter >2 mm, AVXLA) in line with previous findings. The changes in vascular volume distribution were correlated with clinical outcomes after BPA, including NYHA‐FC, 6MWD, NT‐proBNP, and pulmonary hemodynamics assessed by right heart catheterization. Clinical parameters described were measured at baseline and 12 months after the last BPA. The poor responder (Patient 1) was a 54 years old female with NYHA‐FC II, 6MWD = 419 m, NT‐pro‐BNP = 68 pg/mL, mPAP = 25 mmHg, PAWP = 4 mmHg, CO = 7.3 L/min and PVR = 2.9:WU. The good responder (Patient 2) was a 59 years old female, NYHA‐FC III, 6MWD = 204 m, NT‐pro‐BNP = 4922 pg/mL, mPAP = 58 mmHg, PAWP = 35 mmHg, CO = 2.7 L/min and PVR = 8.5 WU. The PAWP of Patient 2 was deemed unreliable due to the absence of cardiac abnormalities on echocardiography. The patients underwent three and five BPA sessions, respectively, and were both treated with dual PH‐specific medication. At 12‐month follow‐up, Patient 1 showed NYHA FC II (0), 6MWD 496 m (+77), NT pro‐BNP = 128 pg/mL (60), mPAP = 21 mmHg (−4 mmg), PAWP = 13 mmHg (+9 mmHg), CO = 6.9 L/min (−0.4 L/min) and PVR = 1.2 WU (−1.7 WU). Patient 2 showed NYHA FC II (−1), 6WMD = 456 m (+252), NT pro‐BNP = 200 pg/mL (−4722), mPAP = 20 mmHg (−38), PAWP = 9 mmHg, CO = 6.0 L/min (+3.3) and PVR = 1.8 WU (−6.7). Deltas are stated in between parenthesis. Patient 1 showed an increase of 3.2% in AVXLA and 1% increase in AVXSA. Patient 2 showed a decrease of 12.9% in AVXLA and 9.6% increase in AVXSA. LungQ was able to detect and quantify the arterial reverse remodeling occurring in a patient who responded good to BPA as opposed to a poor responder. This proof of concept underlines the need for further investigation and potential predictive value of LungQ after BPA.

## A150 MAPPING OF DISEASE‐SPECIFIC ENDOTHELIAL AND STROMAL CELL POPULATIONS RESPONSIBLE FOR INTIMAL, MEDIAL AND ADVENTITIAL ARTERIAL REMODELING DURING DEVELOPMENT OF PULMONARY ARTERIAL HYPERTENSION

Duncan J. Stewart^1^, Nicholas D Cober^1^, Emma McCourt^1^, Rafael Soares Godoy^1^, Yupu Deng^1^, Ken Schlosser^1^, Anu Situ^1^, David P Cook^2^, Sarah Eve Lemay^3^, Timothy Klouda^4^, Ke Yuan^4^, Sébastien Bonnet^3^

^1^Ottawa Hospital Research Institute, Ottawa, Canada, ^2^University of Ottawa, Faculty of Medicine, Ottawa, Canada, ^3^Institut Universitaire de Cardiologie et de Pneumologie de Quebec, Quebec City, Canada, ^4^Boston Children's Hospital, Harvard Medical School, Boston, USA

Pulmonary arterial hypertension (PAH) is a severe and lethal pulmonary vascular disease characterized by arteriolar pruning and occlusive vascular remodeling leading to increased pulmonary vascular resistance and eventually right heart failure. While endothelial cell (EC) injury and apoptosis are known triggers for this disease, the mechanisms by which they lead to complex arterial remodeling remain obscure. We employed multiplexed single‐cell RNA sequencing (scRNA‐seq) at 1, 3, 5, and 8 weeks during the onset and progression of disease in a model of severe PAH to identify mechanisms involved in the development of occlusive arterial lesions. Sprague‐Dawley rats received 20 mg/kg SU5416 (SU) subcutaneously, followed by 3 weeks of exposure to chronic hypoxia (10% O_2_). Hemodynamic measurements and micro‐CT assessment of arterial pruning were performed at baseline, 1, 3, 5, and 8 weeks, and lungs were digested and dissociated into single cells, which were sequenced using the 10x genomics platform. There was a significant loss of arterial volume as early as 1‐week by micro‐CT, preceding any evidence of occlusive arteriopathy, consistent with early arteriolar dropout. Maximal arterial pruning was seen by 5 to 8 weeks, with signs of progressive occlusive remodeling. Analysis of the scRNA‐seq data resolved 44 lung cell populations, with widespread early transcriptional changes at 1 week affecting endothelial, stromal, and immune cell populations. Notably, this included the emergence of a ‘de‐differentiated’ (dD)‐EC population that was characterized by low expression of the endothelial‐restricted transcription factor ETS‐related gene (ERG), a principal determinant of EC identity, together with reduced expression of canonical endothelial genes, including cadherin‐5 and claudin‐5 that play a critical role in endothelial integrity and barrier function. Interestingly, RNA velocity analysis revealed strong vectors within the dD‐EC cluster leading to fibroblast clusters, consistent with endothelial‐mesenchymal transition, (EndMT) likely contributing to adventitial arterial remodeling. In contrast, at later timepoints (5 and 8 weeks), there was a surprising normalization in transcriptomic profiles in nearly all cell populations, with the notable exception of ‘activated’ arterial ECs (aA‐ECs), which continued to exhibit high persistent differential gene expression characterized by a hyper‐proliferative growth dysregulated gene expression profile. This activated EC population was characterized by high expression of Tm4sf1, a gene implicated in cancer cell growth. Tm4sf1 was also expressed by a ‘smooth muscle (SM)‐like’ pericyte cluster, which emerged after 3 weeks, originating from classical pericytes based on trajectory analysis. Both aA‐ECs and SM‐like pericytes showed marked expansion during the development of PAH and, by immunofluorescence staining, were highly localized to regions of complex arterial remodeling in both the rat model and PAH patients, with aA‐ECs responsible for intimal occlusive lesions whereas SM‐like pericytes formed broad bands of medial muscularization. Therefore, multiplexed scRNA‐seq at multiple timepoints during the development of PAH revealed 3 disease‐specific vascular cell populations, aA‐ECs, SM‐like pericytes, and dD‐ECs, that are responsible for intimal, medial, and adventitial arterial remodeling, respectively, the latter involving EndMT. Moreover, Tm4sf1 is uniquely expressed by aA‐ECs and SM‐like pericytes, making it an attractive, novel target for therapeutic strategies designed to reverse intimal and medial arterial remodeling.

## A151 KNOCKDOWN OF PPARΓ IN DISTAL PULMONARY ARTERIAL SMOOTH MUSCLE CELLS FROM PAH PATIENTS LEADS TO DISTURBANCES IN MITOCHONDRIAL METABOLISM AND PATHOLOGICAL CELL PROLIFERATION

Renzhi Su^1^, Jigisha Patel^1^, Michael Simons^2^, Lucie Clapp^1^

^1^University College London, London, UK, ^2^Yale University, New Haven, USA

Pulmonary arterial hypertension (PAH) is defined by its pathological condition, an elevation in mean pulmonary arterial (PA) pressure above 25 mmHg at rest or 30 mmHg with exercise. Although the aetiology of PAH remains unknown, abnormal muscularisation of distal and medial precapillary arteries is one of the most typical pathological changes. Peroxisome proliferator‐activated receptor gamma (PPARγ) is a type II nuclear receptor widely accepted as a key modulator in PAH. Not only can it crosstalk with transforming growth factor beta (TGFβ) and bone morphogenetic protein receptor type 2 (BMPR2) signalling pathways, but it also serves as an important target of prostacyclin (PGI2) analogues. However, the role of PPARγ in distal pulmonary arterial smooth muscle cells (PASMCs) from PAH patients is poorly understood. Primary cell lines of distal PASMCs were isolated by enzymatic dissociation from the explanted lungs of patients with PAH who received a bilateral lung or heart/lung transplant. Small interfering RNA of PPARγ (siPPARγ) was used to knock down PPARγ gene expression, and scrambled RNA was used as a negative control. Gene expression differential analysis and GO clustering analysis of bulk RNA sequencing data showed that one‐third of the genes whose expression levels were significantly changed in the siPPARγ group were categorised in ‘cell cycle’ related clusters. Moreover, the mitochondrial metabolism‐related GO clusters, such as ‘glycolysis/gluconeogenesis’, ‘response to oxygen levels’, and ‘fatty acid oxidation’, were also highlighted with a significant difference in the siPPARγ group compared to the negative control. The Roche xCELLigence Real‐Time Cell Analyzer (RTCA) DP instrument was used to monitor in real‐time migration of PAH PASMCs' and invasion process. Compared to the control group, the absence of PPARγ expression significantly increased cellular invasion/migration. The western blot results showed that the expression of CDK2, a G1/S phase checkpoint biomarker, was also significantly increased. These results indicate the PAH PASMCs enter a more proliferative phenotype after knocking down PPARγ. Surprisingly, the protein expression level of phosphofructokinase 1 platelet isoform (PFKP), a glycolysis biomarker, was significantly decreased in the siPPARγ group, as well as the smooth muscle cell contractile biomarker SM22. The seahorse assay was performed to investigate the mitochondrial function by testing the extracellular acidification rate, the oxygen consumption rate, and the fatty acid oxidation (FAO). The results showed that PPARγ knockdown significantly suppressed glycolytic function and mitochondrial respiration and abrogated the external FAO. These results indicate the PPARγ knockdown aggravates the pathological migration of PAH PASMCs, and the smooth muscle cell contractile phenotype was further diminished. The cells seemed to enter a pathological replication state characterised by rapid growth with low energy consumption due to the loss of normal smooth muscle functions.

## A152 EXPLORING FAMILY DYNAMICS AND ETHICAL CONSIDERATIONS IN THE PAH DIAGNOSTIC JOURNEY: INSIGHTS FROM IN‐DEPTH SEMI‐STRUCTURED INTERVIEWS

Emilia Maria Swietlik^1^, Michaela Fay^2^, Nicholas W Morrell^1^

^1^University of Cambridge, Cambridge, UK, ^2^MF Research Consultancy, Newcastle, UK

Establishing a diagnosis is crucial in medical practice, shaping patients’ experiences and guiding treatment. However, many individuals, particularly those with rare diseases, face prolonged diagnostic journeys. Genetic diagnosis often offers prognostic information and facilitates counselling and relative testing. The NIHR BioResource ‐ Rare Diseases (NBR) Study and the Cohort study in idiopathic and hereditary pulmonary arterial hypertension (PAH Cohort study) aimed to improve diagnosis and treatment for PAH, successfully uncovering disease cause in 25% of idiopathic cases. Yet, research on family dynamics and ethical considerations in the diagnostic process for PAH remains limited. Using purposive sampling, stakeholders from the NBR and PAH Cohort studies were recruited to take part in semi‐structured interviews and focus groups, which were recorded, transcribed, anonymised, and analysed thematically using MAXQDA software following the principles of Grounded Theory (Glaser and Strauss 2009). Higher‐level themes and subthemes were identified through the grouping of codes. Based on the interviews with 63 participants, we were able to identify the following key themes: lengthy diagnostic journeys, the influence of personality traits on coping with a PH diagnosis, complexities of family life, and moral and ethical dilemmas. Five stages emerged in the diagnostic journey: health complaints, which lead to seeking help from GPs, misdiagnosis, relief with the correct diagnosis, and mixed feelings about genetic results and coming to terms with having a life‐altering disease. Complexities of family life were explored, highlighting the consequences of the diagnosis on family dynamics and planning. Genetic testing of relatives played a prominent role in these discussions, with two distinct attitudes towards testing offspring having been identified: proactive advocates and gatekeepers. Advocates of genetic testing viewed it as a chance to proactively prepare for potential disease onset in the event of a positive result. They held optimism that their participation in the research could pave the way for preventive or curative treatments. The gatekeeper attitude was shaped by three primary dispositions: a sense of responsibility to protect children from the weight of knowing their genetic risk, feelings of guilt or a wish to avoid blame for passing on the disease or causing emotional distress related to genetic status awareness, and the impact of loose family connections on the decision to approach relatives for testing. The study underscores the challenges faced during the diagnostic journey in PAH. To improve patient outcomes, it is crucial to enhance disease awareness, establish well‐defined diagnostic pathways, and integrate genetic diagnostics into the clinical workup. The diagnostic process can be expedited by leveraging clinical infrastructure to support research. Moreover, adopting a patient and family‐centred approach is vital to improve the overall disease experience and promote the engagement of relatives in genetic research. These strategies collectively contribute to advancing the understanding and management of PAH.

## A153 UNDERSTANDING WHAT DRIVES GENETIC STUDY PARTICIPATION: PERSPECTIVES OF PATIENTS, CARERS, AND RELATIVES

Emilia Maria Swietlik^1^, Michaela Fay^2^, Nicholas W Morrell^1^

^1^University Of Cambridge, Cambridge, UK, ^2^MF Research Consultancy, Newcastle, UK

While genetics has long been integrated into various subspecialties of respiratory medicine, the importance of genetic research and testing in understanding the fundamental mechanisms of Pulmonary Arterial Hypertension (PAH) and developing effective treatment strategies has recently gained prominence. This study examined attitudes towards genetic research among patients, caregivers, and relatives and gathered feedback for enhancing genetic study delivery and smooth integration of the results into clinical practice. Participants were purposively selected from the NBR and PAH Cohort studies, representing a range of roles, ages, genders, and mutation statuses. We conducted a total of 53 semi‐structured interviews and focus groups involving patients, clinicians, and researchers from nine UK PH centres. Interviews were audio‐recorded, transcribed, and anonymised. Following the principles of Grounded Theory (Glaser and Strauss 2009), transcripts were thematically coded to reveal relevant themes and sub‐themes. The thematic coding process was undertaken by two authors using MAXQDA (2022) software, and the code system was iteratively developed in a series of data analysis sessions. Higher‐level themes and subthemes were identified through the grouping of codes. Additionally, clustering analysis was conducted to identify patient groups that shared similar motivations and barriers to participation. Several key themes emerged from the interview data, shedding light on various aspects of participation in genetic research in PAH. These themes reflected participants' understanding of the aims of the study, attitudes toward genetic research and testing more generally, knowledge of the disease itself, motivations and barriers to participating in genetic research, awareness of and interest in consent procedures, and the use of personal and genetic data, as well as the process of communicating individual genetic results and their impact on family members and relationships. Reasons influencing genetic research participation fell broadly into the following categories: the outward‐looking or altruistic desire to contribute to science and improve health outcomes for others; one's personal experience of the protracted diagnostic journey and resulting trust in and gratitude toward PH teams; as well as hopes and concerns for family members and a sense of belonging to a wider community. Clustering analysis produced distinct clusters based on the presence of barriers and motivators for research participation; however, it was observed that hardly any patients shared identical sets of attitudes, emphasising the need for personalised approaches to recruitment. Most patients reported poor engagement with study‐related materials, including consent forms. Patients who received individual genetic results expressed satisfaction with the process, whereas those who did not were disappointed with the lack of feedback. Reflecting on patients’ perspectives on genetic study delivery, we provide recommendations for improving the process. Increasing genetic research participation and enhancing its integration into clinical practice requires adapting engagement strategies, clear and empathetic communication, and support from healthcare providers, policymakers, and researchers. Further research should explore the impact of genetic research on families, contributing to a more comprehensive understanding of this evolving field.

## A154 UNLOCKING THE POTENTIAL OF GENETIC RESEARCH IN PULMONARY ARTERIAL HYPERTENSION: INSIGHTS FROM CLINICIANS, RESEARCHERS AND STUDY TEAM

Emilia Maria Swietlik^1^, Michaela Fay^2^, Nicholas W Morrell^1^

^1^University Of Cambridge, Cambridge, UK, ^2^MF Research Consultancy, Newcastle, UK

While genetics has been incorporated into various subspecialties of respiratory medicine for some time, the significance of genetic research and testing in comprehending the fundamental mechanisms of Pulmonary Arterial Hypertension (PAH) and formulating effective treatment approaches has only recently come to the forefront. This study aims to explore attitudes toward genetic research, assess clinicians’ abilities and confidence in explaining genetic research findings, and examine their influence on the pace, success, and patient and relative participation in genetic studies. Ultimately, this research seeks to understand how these factors impact the integration of genetic insights into clinical practice. Participants were purposively selected from the NBR and PAH Cohort studies, representing a range of roles, ages, genders, and mutation statuses. We conducted a total of 53 semi‐structured interviews and focus groups involving 63 patients, clinicians, and researchers from nine UK PH centres. Interviews were audio‐recorded, transcribed, and anonymised. Employing inductive analysis, we aimed to comprehensively capture participant perspectives. Following the principles of Grounded Theory (Glaser and Strauss 2009), transcripts were thematically coded to reveal relevant themes and sub‐themes. The thematic coding process was undertaken by two authors using MAXQDA (2022) software, and the code system was iteratively developed in a series of data analysis sessions. Higher‐level themes and subthemes were identified through the grouping of codes. Additionally, clustering analysis was conducted to identify patient groups that shared similar motivations and barriers to participation. From the interview data, several key themes emerged, providing valuable perspectives into specific challenges within the realm of genetic research, ranging from study design, recruitment, and consent procedures to the return of individual genetic results. Additionally, participants reflected on both the successes of these studies and the future directions of genetic research. The analysis highlighted the critical importance of fostering collaborative networks firmly rooted in existing clinical and research infrastructure in rare disease study setups. Strong governance was identified as a prerequisite for achieving success. Furthermore, the significance of trust‐building, personalised communication, and transparency among stakeholders was underscored. The study offered valuable insights into the motivating and hindering factors to participant recruitment and consent procedures. Lastly, the findings gathered from processes surrounding the return of individual genetic results, genetic counselling, and the recruitment of relatives provided invaluable lessons regarding the integration of genetics into clinical practice within the field of respiratory medicine. This in‐depth analysis yields valuable understanding applicable to similar studies in the field, shedding light on the complexities of genetic research and the evidence‐practice gap. Findings from the interview data underscore the value of collaboration and training within the study, revealing lessons learned and future research prospects in PAH genetics. This ongoing exploration promises enhanced diagnosis and treatment for PAH, offering hope for improved outcomes. Notably, the NBR and PAH Cohort studies serve as pioneering models in rare disease research, with potential applicability to other conditions.

## A155 EXCESSIVE NUCLEAR MATRIX ANCHORING HAMPERS ENDOTHELIAL SHEAR PLASTICITY IN PULMONARY ARTERIAL HYPERTENSION

Corey Wittig^1^, Lauren Schmidt^5^, Harm Jan Bogaard^7^, Kirk Hansen^5^, Kurt Stenmark^6^, Robert Szulcek^1^

^1^Institute of Physiology, Charité – Universitätsmedizin Berlin, corporate member of Freie Universität Berlin and Humboldt Universität zu Berlin, Charitéplatz 1, Berlin, Germany, ^2^Deutsches Herzzentrum der Charité, Department of Cardiac Anesthesiology and Intensive Care Medicine, Augustenburger Platz 1, Berlin, Germany, ^3^German Centre for Cardiovascular Research (DZHK), Partner Site Berlin, Berlin, Germany, ^4^German Center for Lung Research (DZL), Berlin, Germany, ^5^Biochemistry and Molecular Genetics, University of Colorado, Denver, USA, ^6^Cardiovascular Pulmonary Research Laboratories, Departments of Pediatrics and Medicine, University of Colorado Anschutz Medical Campus, Denver, USA, ^7^Department of Pulmonary Diseases, Amsterdam UMC, VU University Medical Center, Amsterdam Cardiovascular Sciences (ACS), Amsterdam, The Netherlands

A pathophysiological hallmark of pulmonary arterial hypertension (PAH) is extensive lung vascular remodeling and a subsequent increased pulmonary blood pressure and flow. We have shown that the pulmonary endothelium of PAH patients is prone to shear‐induced cell damage in vitro due to faulty morphological shear‐adaptability. We hypothesize that increased nuclear matrix anchoring in PAH results in a reduced ability of human pulmonary microvascular endothelial cells (HPMVEC) to adjust to disease‐typical supra‐physiological high shear stress (HSS) rendering the cells in a constant state of injury and repair that promotes the pathological lung vascular changes. Whole human lung samples (*n* = 5 control; *n* = 8 PAH) were obtained from lung transplantations and autopsies (METC: 2012/306) and submitted to global proteomic analysis. Control and PAH‐HPMVEC were subjected to 12 h of physiological fluid shear stress (2.5 dyn/cm^2^), followed by 120 h of HSS (15 dyn/cm^2^). siRNA was used for targeted gene silencing in PAH‐HPMVEC for 72 h, following which they were subjected to the same flow regime. The PAH whole lung proteome exhibits significantly increased proteins associated with the nuclear architecture, with 19 proteins in the nuclear domain identified as significantly overexpressed in PAH. Using GO analysis, significant enrichment was found in “nuclear matrix anchoring at nuclear membrane” (*p* = 4.23E−5), “integral component of nuclear inner membrane” (*p* = 2.87E−7), and “lamin binding” (*p* = 7.05E−6). Partial least squares‐discriminant analysis was applied to cluster control versus PAH samples. From this, “VIP” proteins were identified as driving cluster separation, with multiple Linker of Nucleoskeleton and Cytoskeleton (LINC complex) proteins in the top 15, including SUN1/2, SYNE1, and EMD. In addition to the whole lung lysates, SUN1 protein was also found to be significantly overexpressed in PAH‐HPMVEC (*p* = 0.02, FC = 3.06), but transcriptional expression was normal. Subjecting healthy and PAH cells to 120 h HSS confirmed a delayed morphological shear adaptation of PAH‐HPMVEC, with 41.7% fewer adapted cells at 72 h HSS (*p* = 0.04), resolving after 120 h (*p* = 0.15). Silencing of SUN1 in PAH‐HPMVEC rescued the shear response to healthy levels at 72 h, increasing from 57.5% to 72.5% shear‐adapted PAH‐HPMVEC. Our validated whole lung proteome analysis reveals a posttranslational upregulated nuclear matrix‐binding signature in PAH lung samples and HPMVEC, with an emphasis on the LINC complex. Furthermore, we confirm that PAH‐HPMVEC have faulty mechanoadaptive capabilities, which can be rescued by silencing of the core LINC complex protein SUN1. In agreement with our hypothesis, we will further investigate whether this hampered cell plasticity promotes shear‐induced injury and pulmonary vascular remodeling.

## A156 THE IMPACT OF PULMONARY ARTERIAL HYPERTENSION ON HOSPITALIZATIONS FOR COVID‐19 INFECTED PATIENTS: A NATIONWIDE ANALYSIS IN THE UNITED STATES

Omar Tamimi^1^, Deepa Gotur^2^, Zeenat Safdar^2^

^1^Houston Methodist Hospital, Department of Medicine, Houston, USA, ^2^Houston Methodist Lung Center, Department of Medicine, Division of Pulmonary and Critical Care, Houston Methodist Hospital, Houston, USA

This investigation was undertaken to determine morbidity and mortality in pulmonary arterial hypertension (PAH) patients hospitalized with COVID‐19 infection. A comprehensive retrospective analysis of 2020 hospital admissions was conducted using discharge data obtained from the National Inpatient Sample, Healthcare Cost and Utilization Project, Agency for Healthcare Research and Quality. Patients with a principal diagnosis of COVID‐19 and a secondary diagnosis of PAH were identified through the International Classification of Diseases, 10th revision, Clinical Modification codes. Statistical significance was established at *p* < 0.05. The statistical analyses were carried out employing STATA‐17, with adjustments made for all baseline demographics and comorbidities, including hypertension, diabetes mellitus (DM), chronic obstructive pulmonary disease (COPD), coronary artery disease (CAD), and chronic kidney disease (CKD). Among the 1,018,915 adult individuals hospitalized with COVID‐19 in 2020, 155 patients presented with a diagnosis of PAH. There were no statistically significant differences in terms of age, gender, race, insurance status, or median household income between the two groups. However, patients in the PAH cohort exhibited higher Charlson comorbidity index scores of at least 3 (65% vs. 28%) and a lower proportion of chronic hypertension (52% vs. 68%) compared to the non‐PAH cohort. There were no disparities in comorbid CAD, COPD, DM, morbid obesity, or CKD between the two groups. Out of the total COVID‐19 hospitalizations, 113,180 patients died. Among patients admitted with a principal diagnosis of COVID‐19, the presence of PAH was not associated with an elevated risk of mortality compared to those without PAH (adjusted odds ratio [OR] 1.1 [95% CI 0.4–3.2], *p* = 0.87). Additionally, patients with both COVID‐19 and PAH exhibited no statistically significant differences in the odds of requiring mechanical ventilation (adjusted OR 2.23 [95% CI 0.9–5.3], *p* = 0.07), vasopressor therapy (adjusted OR 1.4 [95% CI 0.2–11], *p* = 0.76), acute kidney injury necessitating renal replacement therapy (DD‐AKI) (adjusted OR 1.3 [95% CI 0.17–9.9], *p* = 0.8), mean length of hospital stay (LOS) (11.1 vs. 7.5 days, adjusted difference 3.1 [95% CI −3.8 to 10.1], *p* = 0.37), or mean total hospitalization charges ($195,815 vs. $79,082, adjusted mean difference 107,146 [95% CI −93,939 to 308,232], *p* = 0.29). In the pre‐vaccination area, PAH patients hospitalized with SARS‐CoV‐2 infection demonstrated no increase in mortality but exhibited higher Charlson comorbidity index scores compared to COVID‐19 patients without PAH. Further investigations are warranted to study PAH patient population in the postvaccination era to better understand the impact of outcomes in these patients. Our study underscores that during the pre‐vaccination era, PAH did not confer higher mortality for PAH patients admitted with COVID‐19 infection.

## A157 PULMONARY ARTERIAL HYPERTENSION PATIENTS WITH CLINICAL WORSENING HAVE A DISTINCT GUT MICROBIOME COMPARED TO PATIENTS WITH STABLE DISEASE

Heena Shah^1^, Daphne Moutsoglou^1^, E Kenneth Weir^1^, Alexander Khoruts^1^, Thenappan Thenappan^1^

^1^University of Minnesota, Minneapolis, USA

Pulmonary arterial hypertension (PAH) patients have a less diverse and pro‐inflammatory gut microbiome compared to healthy subjects, but it is unclear whether the altered gut microbiome predicts poor clinical outcomes. To determine whether gut microbial diversity and taxonomy predict clinical worsening in PAH. We defined clinical worsening as heart failure (HF) hospitalization, parenteral prostacyclin initiation, lung transplantation, or death. PAH patients with clinical worsening have distinct taxonomic alterations at baseline compared to stable PAH patients, and less diverse gut microbiome predicts clinical worsening. We studied 85 PAH patients. We performed 16S rRNA sequencing on stool samples. Patients received standard of care PAH therapy and were followed closely every 3–6 months. We collected data on HF hospitalization, parenteral prostacyclin initiation, lung transplantation, and death from the date of stool collection. We used Mothur and MetaboAnalyst programs for microbiome analyses. Cox proportional analyses were used to determine the association between gut dysbiosis and clinical worsening. Over a median follow‐up of 1.8 years, 29 (34%) had clinical worsening, and 56 (66%) had stable PAH. Alpha diversity (median Shannon index: 3.4 vs. 3.5, *p* = 0.06, median Chao index: 257 vs. 278, *p* = 0.41, and median Inverse Simpson index: 16.8 vs. 19.3, *p* = 0.06) and beta diversity indices at baseline were not significantly different between patients with clinical worsening and stable PAH. Genus‐level data at baseline showed distinct clustering via orthogonal partial least squares discriminant analysis of patients with clinical worsening and stable PAH. The top 5 genera that differentiated PAH patients with clinical worsening from those who had stable disease include Streptococcus, Lachnospira, Enterobacteria, Dialister, and Atopostipes. Patients who had clinical worsening had higher abundance of Streptococcus, Lachnospira, Enterobacteria, Atopostipes, Rikenellaceae, Lactobacillus, Truperella, Mogibacterium, Oribacterium and lower abundance of Dialister, Parabacteroide, Puniceicoccace, and Collinsella compared to patients with stable PAH. Patients with Shannon diversity index in the lowest tertile had a trend towards higher risk of clinical worsening when compared to those with Shannon index in the middle and highest tertiles (Hazard ratio: 1.95, 95% CI: 0.91–4.18, *p* = 0.08). Conclusions: PAH patients with clinical worsening have a distinct gut microbiome signature at the genus level compared to those with stable disease with no significant difference in diversity. Gut microbiome may help identify PAH patients who are at risk of clinical worsening. Further interventional studies are needed to determine whether there is a true cause‐and‐effect relationship between gut dysbiosis and clinical worsening in PAH. Patients with clinical worsening have a less diverse gut microbiome with distinct taxonomic alterations compared to stable PAH patients at baseline. We performed 16S rRNA sequencing on stool samples from 85 PAH patients. Patients were followed closely every 3–6 months and received standard of care PAH therapy. We collected data on HF hospitalization, parenteral prostacyclin initiation, lung transplant, and death from the date of stool collection. We used Mothur and MetaboAnalyst programs for microbiome analyses. Of the 85 patients, 29 had clinical worsening, and 56 had stable PAH over a median follow‐up of 1.8 years. Alpha diversity (median Shannon index: 3.38 vs. 3.48, *p* = 0.06, median Chao index: 257 vs. 278, *p* = 0.41, and median Inverse Simpson index: 16.85 vs. 19.33, *p* = 0.06) and beta diversity indices were not significantly different between patients with clinical worsening and stable PAH at baseline. Genus‐level data showed distinct clustering via orthogonal partial least squares discriminant analysis of patients with clinical worsening and stable PAH. PAH patients with clinical worsening had a higher relative abundance of Streptococcus, Lachnospira, Enterobacteria, Atopostipes, Rikenellaceae, Lactobacillus, Truperella, Mogibacterium, Oribacterim and lower relative abundance of Dialister, Planococcus, Eikenella, Parabacteroide, Puniceicoccace, and Collinsella compared to patients with stable PAH. PAH patients with clinical worsening have a distinct gut microbiome signature at the genus level compared to those with stable disease with no difference in diversity. Further interventional studies are needed to determine whether this is a consequence of severe disease or driver of clinical worsening. Gut microbiome may help identify PAH patients who are at risk of clinical worsening.

## A158 SAFETY AND FEASIBILITY OF MICROBIOTA TRANSPLANT THERAPY IN PATIENTS WITH PULMONARY ARTERIAL HYPERTENSION (GUT‐PAH STUDY)

Daphne Moutsoglou, Gretchen Peichel, Madelyn Blake, Amanda Kabbage, Brenda Vang, Kurt Prins, Felipe Kazmirczak, Kenneth E Weir, Alexander Khoruts, Thenappan Thenappan

^1^University Of Minnesota, Minneapolis, USA

Pulmonary arterial hypertension (PAH) patients have altered microbiota that could drive pulmonary vascular remodeling. Microbiota transplant therapy (MTT) could improve the altered PAH gut microbiome and pulmonary vascular disease. To determine the safety and feasibility of MTT in PAH patients. MTT is safe and feasible in PAH. PAH patients received 7 days of encapsulated MTT (compound MTP‐101C), dosed at ~2 × 10^11^ bacteria daily from a healthy donor. Stool and blood samples were collected on days 0, 30, and 180, and a quality‐of‐life assessment (EmPHasis‐10), 6‐min walk test, and echocardiography were obtained on days 0 and 180. Monitoring occurred daily for 2 weeks during dosing and then monthly for 6 months. Primary endpoints included safety and feasibility, and exploratory endpoints included donor microbiota engraftment, changes in emPHasis‐10 score, 6‐min walk distance, and right ventricular function by echocardiography. Eleven patients were enrolled and received MTT. The mean age was 50 ± 15 years, and 64% were females. PAH etiologies included: idiopathic (55%), congenital heart (27%), and connective tissue (18%) disease. Seventy‐three percent of patients were on dual, and 27% were on triple combination therapy. All patients completed the 7‐day dosing of MTT and were able to adhere to the protocol. MTT was safe and well‐tolerated without severe (grade 3) or serious adverse events attributed to MTT. The most common side effects related to MTT were diarrhea (27%), nausea (18%), dyspepsia (18%), and tenesmus (9%). Median engraftment of donor bacteria was 20.2 ± 13.3% at 30 days and 17.7 ± 20.2% at 180 days post‐MTT. Changes in 6‐min walk distance, EmPHasis‐10, and right ventricular fractional area change between pre‐and post‐MTT were 19 ± 44 m, −2.4 ± 5.3, and −0.2 ± 3%, respectively. Conclusions: MTT is safe and feasible in PAH with no serious or severe adverse events. Further study will need to determine how to maximize donor engraftment, and whether MTT could be used as an adjunctive PAH treatment.

## A159 SMOOTH MUSCLE CELL PROLIFERATION AND MEDIAL HYPERPLASIA IN PAH IS DRIVEN BY NOTCH3‐BMPR INTERACTIONS VIA DOWNSTREAM EFFECTORS ID1, ID3, AND HES‐5

Cristian Puerta^1^, Yu Zhang^1^, Nolan Winicki^1^, Moises Hernandez^1^, Patricia Thistlethwaite^1^

^1^Division of Cardiothoracic Surgery, University of California, San Diego, La Jolla, USA

One of the characteristics of pulmonary arterial hypertension (PAH) is the medial hyperplasia in small pulmonary arteries. In heritable PAH, aberrant BMPR2 signaling in small pulmonary artery muscle cells (sPAMC) results in low levels of the intracellular peptide ID1. For non‐heritable PAH, the NOTCH3–HES‐5 signaling pathway has been implicated to promote vascular smooth muscle cell proliferation. Previous studies have demonstrated that HES‐5 and ID1 are peptides belonging to the basic helix‐loop‐helix family and that they act as transcriptional repressors of cell cycle inhibitor genes. We sought to explore the roles and interactions of HES‐5 and ID1 in PAH. The half‐life of ID proteins was assessed in human sPASMCs that either constitutively‐expressed or minimally‐expressed HES‐5. To understand whether this effect was due to direct binding between HES‐5 and ID1, co‐immunoprecipitation experiments were performed. Immunofluorescent staining was used to detect the intracellular location and semi‐quantitative levels of ID1 and HES‐5 in sPASMCs. Proximity ligation assay (PLA) was utilized to examine the possible endogenous in‐situ interactions of ID1‐HES5. Overexpression of HES‐5 significantly reduced the half‐life of ID1 and ID3 peptides, with minimal effect on ID2 in sPAMCs. All four proteins localized to the nucleus of human sPASMCs. Immunofluorescence staining confirmed elevated levels of HES‐5 and reduced levels of ID1 and ID3 in the medial layer of PAH small pulmonary arteries compared to non‐PAH small pulmonary arteries. PLA assay of PAH sPASMCs demonstrated that HES‐5 and ID1, as well as HES‐5 and ID3, physically bind to each other at the level of 40 nanometers, suggesting that HES‐5 binding to ID1 or ID3 targets these peptides for degradation. Constitutive NOTCH3 signaling and high intracellular HES‐5 levels drive vascular smooth muscle cell proliferation in nonfamilial PAH by binding to and degrading intracellular ID proteins. Loss of ID1 and ID3 results in an increase of inhibitory transcription factors, which ultimately decreases the production of tumor suppressors within the cell. Our study demonstrates cross‐regulation between the NOTCH3 pathway and the BMPR2 pathway in nonfamilial PAH, at the level of their downstream effectors: ID1, ID3, and HES‐5. Further studies should illuminate how these two pathways control the PAH disease process.

## A160 IDENTIFICATION OF MOLECULAR MECHANISMS DRIVING PHYSIOLOGICAL REVERSE PULMONARY VASCULAR REMODELING IN PULMONARY HYPERTENSION DUE TO LEFT HEART DISEASE

Donghai Tian^1,2,3^, Mariya M Kucherenko^1,2,3^, Pengchao Sang^1,2,3^, Netra nambiar‐veetil^1,2,3^, Christoph knosalla^1,3^, Wolfgang M Kuebler^2,3^

^1^Department of Cardiothoracic and Vascular Surgery, Deutsches Herzzentrum der Charité (DHZC), Berlin, Germany, ^2^Institute Of Physiology, Charité‐universitätsmedizin Berlin, Berlin, Germany, ^3^German Centre for Cardiovascular Research (DZHK) ‐ partner site Berlin, Berlin, Germany

Pulmonary hypertension (PH) due to left heart disease (PH‐LHD) is the most common form of PH that ultimately leads to right ventricular (RV) failure, contributing to morbidity and mortality of LHD patients. Pulmonary vascular remodeling further aggravates PH‐LHD by increasing pulmonary vascular resistance (PVR). Yet, implantation of left ventricular assist devices or heart transplantation can normalize PVR in PH‐LHD patients over time, indicating that unloading of the pulmonary circulation reverses pulmonary vascular remodeling via physiological mechanisms that are not yet understood but may point towards novel therapeutic strategies in PH. (1) To characterize reverse pulmonary arterial (PA) and RV remodeling in an animal model of aortic banding/debanding; (2) To identify associated molecular mechanisms driving reverse PA remodeling. To enable molecular and interventional studies in vivo, we developed a rat model of reverse vascular remodeling in PH‐LHD by initial surgical aortic banding (AoB) followed after 3 weeks by surgical debanding (Deb). Reverse remodeling of PA and RV was evaluated by hemodynamic assessment and (immuno‐)histological analyses of lung and heart tissue samples. Gene candidates driving reverse PA remodeling were identified by bulk‐RNA sequencing of sham, AoB, and Deb PA samples and genes uniquely expressed in Deb PA samples were considered as potential candidates driving reverse remodeling. The functional relevance of identified candidates was then tested in primary PA smooth muscle cells (PASMC) isolated from sham and AoB rats. Hemodynamic assessment and histological analyses identified normalization of pulmonary and RV hemodynamics and reverse pulmonary vascular and RV wall remodeling in Deb versus AoB rats. Immunostaining for Ki67 revealed reduced SMC proliferation rates in PAs of Deb versus AoB rats; in the RV, wall thickness, width, and area of cardiomyocytes were decreased in Deb versus AoB rats. RNA sequencing identified PPARGC1A (PPARγ Coactivator 1‐α) and ESRRG (estrogen‐related receptor γ) as candidates that may drive reverse PA remodeling by re‐establishing mitochondrial biogenesis and homeostasis in PASMC. In vitro, activation of PPARγ and ESRRγ inhibited the proliferation of PASMC isolated from PH‐LHD rats. Surgical aortic debanding can reverse pulmonary vascular remodeling and RV hypertrophy in a preclinical model of PH‐LHD. Activation of PPARγ and ESRRγ were identified as potential drivers of reverse PA remodeling by re‐establishing mitochondrial biogenesis and homeostasis in PASMC. Funding: Supported by grants from the German Center for Cardiovascular Research (DZHK), the German Research Foundation (DFG), and the Berlin Institute of Health (BIH).

## A161 THE ROLE OF PSYCHOLOGICAL SAFETY & BIASES IN TREATMENT CHOICE AND HEALTHCARE—AN INTERACTIVE SESSION

Klodiana Daphne Tona^1^

^1^Radboudumc, Nijmegen, The Netherlands

A safe working environment is essential in medical care and research. This includes psychological safety, i.e., creating an environment where healthcare providers feel respected, comfortable expressing ideas, asking questions, and challenging existing knowledge without fear of judgment or reprisal. When a highly safe working environment is also combined with high‐performance accountability (i.e., healthcare providers are eager to perform high‐quality research, provide better services to the patients, and set high standards for their work), this leads to a learning environment, a context that facilitates learning, innovation, growth. Unsurprisingly, such an environment leads to high‐quality services, reduced biases, better outputs, reduced error rates and thus higher survival rates for patients. In an interactive discussion, 1. the psychological factors that affect healthcare providers will be examined (including possible biases that affect decision‐making and could be a matter of life‐or‐death), 2. the current status/practices of different countries, and 3. what factors can contribute to a better healthcare culture in your own environment to improve the care of patients with PAY.

## A162 THE RELATIONSHIP BETWEEN TSH LEVELS AND STRESS DUE TO A LARGE‐SCALE INVASION IN UKRAINE IN PATIENTS WITH PH

Olena Torbas^1^, Ganna Radchenko, Yurii Botsiuk, Serhii Prohonov, Yuriy Sirenko

^1^NSC Institute of cardiology, clinical and regenerative medicine n.a. Acad. M.D. Strazhesko, Ukraine

It is known that long‐term stress can provoke the development of hypothyroidism since stress hormones can block the function of the thyroid gland. Patients with pulmonary hypertension (PH) were among those who suffered the most from stress and anxiety during the year of the full‐scale invasion of Ukraine because of fear of being left without drugs and special medical care. We also know that subclinical hypothyroidism in patients with PH leads to the worsening of this disease. Aim: The purpose of the analysis was to evaluate the average level of thyroid‐stimulating hormone (TSH) in patients admitted to a specialized PH center during the year 2022 (full‐scale invasion) and to compare this data with previous years' results. We analyzed the data of the single registry of the PH adult center in Kyiv from Jan 2015–Jan 2023. Throughout the year of the large‐scale invasion, the PH center continued its work and provided specialized medical care. We divided the entire database of 571 patients into groups according to the year of inclusion—2015, 2016, 2017, 2018, 2019, 2020, 2021, and 2022 (Table 1). About 70% of the included patients had TSH results used in this analysis. Statistical analysis was done using the IBM SPSS 26.0 software package. All groups were comparable according to age, gender, and BMI (parameters potentially influencing TSH level). All groups also were comparable according to the structure of the PH group (see Table 1), so we may conclude that the structure of patients included each year was almost similar. We have found relatively higher levels of TSH in patients included in 2022 compared to other years (3.87 ± 0.67 mIU/L vs 2.56 ± 0.43; 2.07 ± 0.24; 2.78 ± 0.42; 2.75 ± 0.73; 2.22 ± 0.19; 2.66 ± 0.34; and 2.17 ± 0.41 mIU/L in 2015, 2016, 2017, 2018, 2019, 2020, 2021, and 2022 respectively), but this difference was significant only for difference with 2016 (*p* < 0.05). Moreover, a slightly higher level of TSH was also observed for those included in previous years but were hospitalized again in 2022 due to worsening (3.24 ± 0.22 mIU/L vs 2.42 ± 0.21 in 2015–2021), but it was not significant. The short follow‐up during the stress period and the small number of patients were limitations of this analysis. We observed a moderate, statistically nonsignificant trend towards increased TSH during the last year in all patients with PH. As the war on the territory of Ukraine continues, we expect an additional increase in TSH in 2023. Nevertheless, the data we obtained demonstrate that it is better to involve an expert endocrinologist in the multidisciplinary team of the PH expert center, especially when there could be an influence of severe stressful situations.

## A163 GROWTH DIFFERENTIATION FACTOR 15(GDF‐15): A COMPREHENSIVE ASSESSMENT OF THE BIOMARKER'S ROLE IN ADULT AND PEDIATRIC PULMONARY ARTERIAL HYPERTENSION SEVERITY AND SURVIVAL

Guillermo Torres‐Viera^1^, MS Kate Schole^1^, Megan Griffiths^2^, Jun Yang^1^, Stephanie Brandal^1^, Rachel Damico^1^, Dhananjay Vaidya^1^, Catherine Simpson^1^, Todd Kolb^1^, Stephen Mathai^1^, Michael Pauciulo^3^, William Nichols^3^, David Ivy^4^, Eric Austin^5^, Paul Hassoun^1^, Allen Everett^1^

^1^Johns Hopkins Hospital, Baltimore, USA, ^2^University of Texas Southwestern, Dallas, USA, ^3^Cincinnati Children's Hospital Medical Center, Cincinnati, USA, ^4^Children's Hospital Colorado, Denver, USA, ^5^Vanderbilt University Medical Center, Nashville, USA

Pulmonary arterial hypertension (PAH) is a progressive disease characterized by vascular remodeling resulting in right heart failure. The diagnostic and prognostic utility of Growth differentiation factor 15 (GDF‐15), a member of the transforming growth factor‐B superfamily, in this disease has been previously elucidated. This study was aimed at exploring the utility of GDF‐15 as a PAH biomarker between WSPH subtypes as well as in the pediatric population. Serum GDF‐15 was measured in the PAH Biobank (*N* = 2428, 147 pediatric), healthy controls (*N* = 50 adults, 114 pediatric), and longitudinal cohorts from Vanderbilt (*N* = 120) and Children's Hospital of Colorado (*N* = 60). Clinical, hemodynamic, and echocardiographic data was used to evaluate associations between GDF‐15 and PAH severity and survival. GDF‐15 discriminated PAH from healthy controls. Increased GDF‐15 (age/sex‐adjusted regression) was associated with decreased 6‐min walk distance in adult (−29.2 m) and pediatric cohorts (−145.7 m). Adjusted Cox hazards model revealed significantly increased mortality risk in adults (HR 3.9, <0.001) and children (HR 8.9, *p* = 0.01). Most significant divergence in KM survival curves was noted in adult IPAH patients. Similar to adult data, increased GDF‐15 was associated with the worst outcomes in the pediatric IPAH population (HR 17.3, *p* = 0.01). By use of large adult and pediatric PAH cohorts, this study revealed significant differences in GDF‐15 levels between healthy pediatric and adult controls and PAH counterparts, as well as its significant associations with survival in children and adults. Despite known associations between GDF‐15 and connective tissue disease particularly, worse outcomes were noted in IPAH subtype. These differences may offer additional prognostic value in the clinical evaluation and treatment of PH.

## A164 EARLY CARDIAC ADAPTATION AND MORPHOLOGY OF UNAFFECTED BMPR2 CARRIERS IN THE ABSENCE OF PULMONARY ARTERIAL HYPERTENSION

Eszter Nóra Tóth^1,2^, Md Lucas Celant^1,2^, Samara Jansen^1,2^, Lilian Meijboom^2,3^, Anton Vonk Noordegraaf^1,2^, Frances de Man^2,4^, Harm Jan Bogaard^1,2^

^1^Department of Pulmonary Medicine, Amsterdam UMC, Amsterdam, the Netherlands, ^2^Pulmonary Hypertension and Thrombosis, Amsterdam Cardiovascular Sciences, Amsterdam, the Netherlands, ^3^Department of Radiology and Nuclear Medicine, Amsterdam UMC, Amsterdam, the Netherlands, ^4^PHEniX Laboratory, Department of Pulmonary Medicine, Amsterdam UMC, Amsterdam, the Netherlands

Mutations in the gene encoding for BMPR2 are a major genetic risk factor for hereditary pulmonary arterial hypertension (PAH). Despite similar afterload, patients with BMPR2‐associated PAH have more right ventricular (RV) impairment when compared to patients with idiopathic PAH In addition, novel transgenic BMPR2 rat models have demonstrated the presence of PH‐independent intrinsic RV dysfunction, implying that BMPR2 may play a direct role in cardiac structure and function. Therefore, the aim of our study was to investigate whether structural and functional differences could already be observed in healthy mutation carriers in comparison to healthy controls. Twenty‐nine BMPR2 mutation carriers (mean age 43 ± 15 years, 58% female) and 20 healthy controls (mean age 43 ± 18 years, 45% female) underwent cardiac magnetic resonance imaging (cMRI) and right heart catheterization (RHC) as part of a multimodal, ongoing, prospective screening program. Images were analyzed to assess cardiac volumetrics and mass to compare with healthy controls. Using the single beat method, pressure–volume (PV) loops were constructed to assess load‐independent RV function, RHC data was compared with an existing control group (Trip et al. ERJ 2015) and included only healthy subjects. BMPR2 mutation carriers had lower indexed right ventricular end diastolic—(65 ± 14 ml/m2 vs 78 ± 18 ml/m2; *p* = 0.004), end systolic—(28 ± 8 mL/m^2^ vs 34 ± 11 ml/m^2^; *p* = 0.034) and left end‐diastolic volumes (60 ± 3 ml/m^2^ vs. 69 ± 14 mL/m^2^; *p* = 0.023) than control subjects. Myocardial strain analysis at baseline demonstrated an increased global right ventricular circumferential strain (−16 ± 3% vs. −13 ± 3%; *p* = 0.013) in BMPR2 mutation carriers. There was no difference observed in left ventricular strain. BMPR2 mutation carriers had a significantly higher mPAP (17 ± 2 mmHg vs. 14 ± 2 mmHg; *p* = 0.0008) when compared to controls, but none of the included subjects met the criteria for the diagnosis of PAH at baseline. Evaluation of PV‐loops showed that BMPR2 mutation carriers had a significantly higher afterload (Ea) (0.27 ± 0.08 vs. 0.21 ± 0.06; *p* = 0.033) and lower RV‐pulmonary artery (PA) coupling (Ea/Ees)(1.36 ± 0.37 vs. 2.43 ± 1.45; *p* = 0.013), than healthy controls. Contractility (Ees) trended lower in BMPR2 mutation carriers but was not significant (0.35 ± 0.10 vs. 0.49 ± 0.26; *p* = 0.072). During the follow‐up period, two BMPR2 mutation carriers developed PAH. Neither patient had clinical or echocardiographic signs indicative of PAH. Both patients were diagnosed using RHC and were promptly started on double therapy consisting of a PDE5 inhibitor and an endothelin receptor antagonist. BMPR2 mutation carriers have smaller cardiac volumes and show signs of RV‐PA uncoupling. Additionally, elevated mPAP in unaffected BMPR2 carriers could be a consequence of subclinical changes in the pulmonary vascular bed. These results suggest that carrying a genetic mutation in BMPR2 is associated with structural cardiac changes even in the prelude or absence of PAH.

## A165 SGLT‐2 INHIBITORS FOR THE TREATMENT OF IDIOPATHIC PULMONARY ARTERIAL HYPERTENSION: A PROOF OF CONCEPT STUDY

Eszter Nóra Tóth^1,2^, Erik Duijvelaar^1,2^, Keimei Yoshida^1,2,5^, Marielle van der Veerdonk, Lilian Meijboom^2,4^, Harm Jan Bogaard^1,2^

^1^Department of Pulmonary Medicine, Amsterdam UMC, Amsterdam, the Netherlands, ^2^Pulmonary Hypertension and Thrombosis, Amsterdam Cardiovascular Sciences, Amsterdam, the Netherlands, ^3^Department of Cardiology, Amsterdam UMC, Amsterdam, the Netherlands, ^4^Department of Radiology and Nuclear Medicine, Amsterdam UMC, Amsterdam, the Netherlands, ^5^Kyushu University, Department of Cardiovascular Medicine, Graduate School of Medical Sciences, Fukuoka, Japan

Pulmonary arterial hypertension (PAH) is a progressive disease characterized by obliteration of the pulmonary vasculature resulting in right ventricular (RV) failure. In rat models of PAH induced by monocrotaline (MCT) and Sugen‐hypoxia, administration of sodium‐glucose cotransporter 2 (SGLT‐2) inhibitors, namely empagliflozin, resulted in decreased pulmonary artery pressure and decreased mortality. Additionally, empagliflozin has been shown to decrease pulmonary artery pressures independent of loop diuretics in patients with left heart failure. The effects of empagliflozin in patients with PAH remain unknown. Our objective was to assess the safety and therapeutic potential of empagliflozin in patients with PAH. This is an ongoing open‐label single‐arm interventional proof‐of‐concept study. Idiopathic and hereditary PAH patients above 18 years of age with WHO functional class of II–III were eligible for participation provided that they were stable on standard therapy for at least 90 days before inclusion and had no severe renal or hepatic impairment. Hemodynamic criteria for inclusion were a mean pulmonary artery pressure (mPAP) ≥ 20 mmHg at rest, pulmonary arterial wedge pressure (PAWP) ≤ 15 mmHg, and pulmonary vascular resistance (PVR) ≥ 3 wood units (WU). Prior use of SGLT‐2 inhibitors, hypotension, the use of supplemental oxygen, or a history of severe hypoglycemia were among the exclusion criteria. Patients received a daily dose of 10 mg empagliflozin and were followed during a 12‐week period. The primary study endpoints were comprised of study feasibility, drug safety and drug tolerability. Secondary endpoints were assessed with health‐related quality of life (HRQoL) questionnaires, laboratory testing, 6‐min walk distance (6MWD), transthoracic echocardiography, and cardiac MRI. Out of the planned total of eight patients, six have already been enrolled over a 6‐month screening period. During the study, two serious adverse events occurred. Both adverse events afflicted the same patient and were deemed to be unrelated to the use of empagliflozin. Empagliflozin was well‐tolerated by all enrolled patients. There were no differences in cardiac function, 6MWD, or N‐terminal brain natriuretic peptide (NT‐proBNP) levels in the patients who completed the first 12‐week follow‐up period. HRQoL questionnaires did not show significant improvement after 12 weeks of empagliflozin use. Data from additional participants is pending. Empagliflozin appears to be safe and well‐tolerated in patients with PAH. Although the study was not designed to establish clinical efficacy, there were no signals for clinical improvements.

## A166 RXFP1 AGONISM AMELIORATES EXPERIMENTAL PH BY ATTENUATING TGFΒ‐MEDIATED SMOOTH MUSCLE PHENOTYPIC MODULATION

Georgios Triantafyllou^1,2^, Peiran Yang^1^, Lai‐Ming Yung^1^, Courtney Myhr^3^, Stephanie Kim^1^, Zachary Augur^1^, Christopher VanDeusen^4^, Geoffrey Bocobo^1^, Teresa Dinter^1^, Jean Cavallo^5^, David Chain^5^, Shannon Dwyer^5^, Mark Southwood^6^, Zhiqiang Jia^5^, Nicholas Morrell^6^, Stephane Illiano^5^, Marc Ferrer^7^, Kenneth Wilson^7^, Noel Southall^7^, Philip Sanderson^7^, Irina Agoulnik^3^, Juan Jose Marugan^7^, Alexander Agoulnik^3^, Paul Yu^1^

^1^Cardiovascular Research Center, Division of Cardiology, Department of Medicine, Massachusetts General Hospital, Harvard Medical School, Boston, USA, ^2^Division of Pulmonary and Critical Care Medicine, Brigham and Women's Hospital, Harvard Medical School, Boston, USA, ^3^Department of Human and Molecular Genetics, Herbert Wertheim College of Medicine, Florida International University, Miami, USA, ^4^Omdana Therapeutics, Boston, USA, ^5^Sanofi, Paris, France, ^6^Cambridge University, Cambridge, UK, ^7^National Center for Advancing Translational Sciences, National Institutes of Health, Rockville, USA

Pulmonary arterial hypertension (PAH) is characterized by irreversible, progressive obliteration of the pulmonary arterial vascular bed, which increases right ventricular (RV) afterload ultimately leading to RV failure and death. Pulmonary vascular remodeling in PAH entails aberrant extension, proliferation and hypertrophy of pulmonary artery smooth muscle cells (PASMC) and neointimal remodeling, both known to involve transforming growth factor‐beta (TGF‐β) and SMAD2/3 signaling. Agonism of the relaxin family peptide receptor‐1 (RXFP1) with relaxin‐2 (RNL2) inhibits extracellular matrix synthesis and fibroblast activation by attenuating SMAD2 activation by TGF‐β1. The clinical potential of RLN2 is limited by its short half‐life and parenteral administration. ML290, a small molecule allosteric agonist of human RXFP1 with favorable pharmacokinetics, decreased collagen synthesis, smooth muscle actin expression, and stellate cell proliferation in a humanized RXFP1 knock‐in (KI) mouse model of hepatic fibrosis. We hypothesized that RXFP1 receptor activation by ML290 could attenuate TGFβ‐SMAD2/3 signaling to reduce pulmonary vascular remodeling and pulmonary hypertension (PH) in RXFP1 KI mice. RXFP1 expression was assessed by immunohistochemistry in human PAH and control lung sections. Primary cultured human PASMC were exposed to TGF‐β1 (1 ng/mL) and ML290 (10 μM). Gene expression was measured by RT‐ PCR and immunoblot. Cell migration was assessed using Platypus. RXFP1 KI mice were exposed to SU5416 (20 mg/kg sc) and hypoxia (SuHx, FIO2 = 0.1) for 3 weeks while receiving ML290 (30 mg/kg/day IP) or vehicle. Invasive hemodynamics, Fulton's index and pulmonary vascular remodeling were assessed. RXFP1 was expressed in the medial layer of pulmonary arteries of human lungs and enriched in PAH lungs. Given localization of RXFP1 to the tunica media we examined the in‐vitro effects of RXFP1 agonism in PASMCs. ML290 treatment of PASMCs blunted TGFβ1‐induced SMAD2/3 phosphorylation, and attenuated TGFβ1‐mediated expression of contractile function genes (Acta2, Calponin, Caldesmon) and fibrosis (Fibronectin1, Col1α2), and inhibited PASMC migration and proliferation. Treatment of SuHx‐exposed RXFP1 KI mice with ML290 attenuated PH (RVSP: 35.1 ± 2.3 vehicle‐treated vs 29.6 ± 3.3 ML290‐treated, *p* < 0.0001, *n* = 4–7), decreased RV hypertrophy (Fulton index: 0.3 ± 0.02 vehicle‐treated vs. 0.27 ± 0.02 ML290‐treated, *p* < 0.01, *n* = 4–6), and percentage of muscularized vessels (23.4% ± 3 vehicle‐treated vs 8.5% ± 2.7 ML290‐treated, *p* < 0.01, *n* = 3–5). ML290 treatment ameliorates experimental PH by attenuating pulmonary arteriolar remodeling, antagonizing TGFβ1‐mediated plasticity, migration and proliferation of PASMC.

## A167 GENETIC AND EPIGENETIC REGULATION OF COL18A1 3'UTR IN PULMONARY ARTERIAL HYPERTENSION

Tijana Tuhy^1^, Anjira Ambade^1^, Catherine Simpson^1^, Paul Hassoun^1^, Rachel Damico^1^

^1^Division of Pulmonary and Critical Care Medicine, Department of Medicine, Johns Hopkins University, Baltimore, USA

Pulmonary arterial hypertension (PAH) is a vasculopathy with a poor prognosis. Expression of collagen alpha‐1 (XVII) and endostatin (ES), both encoded by the COL18A1 gene, are increased in the right ventricle (RV) and circulation in human PAH and are associated with right ventricular (RV) dysfunction and increased mortality. Published and preliminary work by our group has identified single‐nucleotide polymorphisms (SNP) in COL18A1 linked to phenotype, including cardiac output, and expression in PAH. This study sought to define the regulation of one of these polymorphisms and its relationship to COL18A1/ES expression in vivo and in vitro. RV tissue was obtained from endomyocardial biopsies (EMB) derived from PAH and control patients at time of right heart catheterization. Male Wistar rats were randomized to Sugen 5416 (single dose) and chronic hypoxia (Su/CH) versus vehicle and housing in room air. Hemodynamic measurements and heart and lung tissues were obtained at 7, 14, and 21 days. Human pulmonary microvascular endothelial cells (PEC) were exposed to ambient air or hypoxia (4%O_2_, 5%CO_2_, 91%N), or treated with endothelin‐1 (ET‐1, a potent vasoactive peptide), at increasing concentrations. Expression of miR in EMB, rodent tissues, and PECs was quantified by RT‐QPCR. Finally, pathways analysis software was used to determine additional putative targets of candidate miR that may be relevant to PAH development or progression. A SNP in the 3′ untranslated region (UTR) is predicted to disrupt the binding of multiple micro‐RNA (miR), including miR‐637, miR‐1270, miR‐4270, and miR‐663b. In Su/CH rats, miR‐637 expression was significantly decreased in the RV tissues at 14 and 21 days compared to controls (*p* = 0.04). No significant difference between groups was seen in miR‐4270 or miR‐663b expression at any time point. In response to hypoxia in vitro, miR‐637 expression in PECs decreased 0.8‐fold (*p* = 0.02, 95% CI −1.67 to −0.14). No significant change was seen in miR‐1270, miR‐4270, or miR‐663b. When treated with ET‐1, miR‐637, miR‐4270, and miR‐663b expression in PECs decreased significantly. Specifically, miR‐637 expression decreased 0.63‐fold in response to treatment with endothelin‐1 at 10 nM (*p* = 0.005, 95% CI −1.110 to −0.2925). miR‐4270 expression decreased 0.8‐fold and 0.7‐fold in response to treatment with ET‐1 at 1 and 10 nM, respectively (*p* = 0.003 and 0.001). miR‐663b decreased 0.7‐fold and 0.59‐fold in response to treatment with ET‐1 (*p* < 0.001). Pathways analysis of candidate miRs demonstrated genes involved in EGFR tyrosine kinase inhibitor resistance, MAPK signaling, PI3K‐Akt signaling, and glutamatergic synapse signaling. Polymorphisms in COL18A1 have been linked to differences in ES expression, hemodynamics, and outcomes in PAH. RS17004785 in the 3'UTR of COL18A1 increases mRNA stability and predictably disrupts miR binding, including miR‐637. MiR‐637 is decreased in the RV in human and Su/CH rats, is suppressed by hypoxia and ET‐1 in vitro, and inversely correlates with COL18A1/ES in vivo. Further, the identified miR, including miR‐637, are linked to signaling pathways previously implicated in PAH pathobiology. Our work implicates 3'UTR polymorphisms in mRNA stability potentially via loss of miR‐mediated gene regulation and lays the foundation for investigating the contribution of miR‐637 in disease‐associated COL18a1/ES expression.

## A168 PRIOR LUNG INFLAMMATION INDUCED BY INTRANASAL DOUBLE‐STRANDED RNA IN THE SUGEN‐HYPOXIA MODEL OF PULMONARY HYPERTENSION ENHANCED PULMONARY VASCULAR REMODELLING

Helena Turton^1^, Laura West^1^, Nadine Arnold^1^, Carl Wright^1^, Sheila Francis^1^, Helen Marriott^1^, Allan Lawrie^2^, Roger Thompson^1^

^1^University Of Sheffield, Sheffield, UK, ^2^Imperial College London, London, UK

Inflammation and alterations in innate immunity are key drivers of pulmonary vascular remodelling. Innate immunity can be regulated by prior activation of the immune system, but it is not known whether innate re‐programming following viral inflammation could impact upon vascular remodelling and contribute to long‐term sequela such as PAH. To investigate if prior lung inflammation induced by double‐stranded RNA (dsRNA) alters the severity of pulmonary hypertension (PH) in the Sugen‐hypoxia (SuHx) mouse model. C57BL/6J male mice were instilled with a single dose of intranasal (IN) synthetic dsRNA, (100 μg/50 μL poly(I:C)), before induction of SuHx on day 9. Some mice were killed at 24‐h and 9 days post IN poly(I:C) instillation for quantification of pulmonary immune responses including bronchoalveolar lavage fluid (BALF) immune cell counts, cytokine analysis, and lung tissue histology. Other mice were killed on day 30 (after IN poly(I:C) and 3‐week SuHx exposure) for PH haemodynamic phenotyping via left and right cardiac catheterisation. Estimated pulmonary vascular resistance (ePVR) was calculated using right ventricular end‐systolic pressure (RVESP) to estimate mean pulmonary artery pressure, left ventricular end‐diastolic pressure (LVEDP), and LV cardiac output (LV CO). Pulmonary vascular remodelling morphometry was acquired by α‐SMA staining and percentage muscularisation via ABEVG staining in pulmonary vessels (<50 μm) and right ventricular (RV) hypertrophy was calculated using Fulton's index. A single dose of IN poly(I:C) induced inflammation in BALF 24 h after instillation as assessed by increased total cell count, percentage of and total number of neutrophils, iNOS and CD206 positive cells and KC concentration, before returning to baseline levels 9 days later. RVESP was not altered in SuHx mice previously instilled with IN poly(I:C) compared to saline controls; however, ePVR was increased. Furthermore, SuHx mice previously instilled with IN poly(I:C) had significantly enhanced pulmonary vascular muscularisation and RV hypertrophy compared to saline controls. Previous instillation of a single dose of IN poly(I:C) provoked subtle changes in the PH haemodynamic phenotype and enhanced pulmonary vascular remodelling. This work implies that prior viral lung inflammation could alter susceptibility to or severity of pulmonary hypertension and future work will include a multi‐hit model, using mice with genetic susceptibility to PH.

## A169 30 HOURS AT HIGH ALTITUDE (2500 M) IN PATIENTS WITH PULMONARY ARTERIAL OR CHRONIC THROMBOEMBOLIC PULMONARY HYPERTENSION—ADVERSE EVENTS AND EFFECT OF OXYGEN THERAPY

Simon R. Schneider^1^, Julian Müller^1^, Meret Bauer^1^, Laura Mayer^1^, Michael Furian^1^, Esther I. Schwarz^1^, Konrad E Bloch^1^, Mona Lichtblau^1^, Silvia Ulrich^1^

^1^University Hospital Zurich, Zürich, Schweiz

High altitude (HA) travel is popular, but hypobaric hypoxia may harm patients with pulmonary vascular disease (PVD) defined as pulmonary arterial or distal chronic thromboembolic pulmonary hypertension (PAH/CTEPH). Twenty‐seven stable PVD patients (44% female, 74% PAH, 26% distal CTEPH) stayed for 30 h at 2500 m. Altitude‐related adverse events (AEHA) defined as severe hypoxemia (SpO_2_ < 80% >30 min), any intercurrent illness, or acute mountain sickness (AMS) were noted, and severe hypoxemia subsequently treated with supplemental oxygen therapy (SOT, 3 L/min). 6‐min walk distance (6MWD) and pulmonary artery pressure (PAP) were assessed at 470 m and the second day at 2500 m according to a cross‐over RCT. The main outcomes were the occurrence of AEHA and the effects of SOT. Secondary outcomes were changes between 2500 versus 470 m stratified by SOT‐indication. AEHA occurred in 14/27 patients (severe hypoxemia 10 (9 nocturnal), mild AMS 4, combined 3). Patients felt subjectively well for 30 h at 2500 m, 10/27 on SOT. The second day at 2500 m vs. 470 m, patients without SOT revealed [mean ± SD; mean‐difference (95%‐CI)] higher PAP 61 ± 23 versus 40 ± 19; 21(7 to 35) mmHg, lower SpO_2_ 89 ± 3 versus 96 ± 2%, −7 (−9 to −5)% and similar 6MWD. Patients on SOT restored baseline values with the exception of a lower 6MWD [485 ± 78 vs. 558 ± 85; −73(−144 to −2) m]. Low‐risk PVD‐patients tolerated a weekend‐getaway to 2500 m for up to 30‐h generally well, and none needed evacuation. In case of severe hypoxemia, SOT reverses SpO_2_ and sPAP to LA‐levels, but not 6MWD. The results of this field study help to counsel PVD‐patients for HA‐sojourns and call for future longer‐term studies. (Clinicaltrials. gov: NCT05107700 & NCT05112172).

## A170 PATIENTS WITH PULMONARY ARTERIAL OR CHRONIC THROMBOEMBOLIC PULMONARY HYPERTENSION PERMANENTLY LIVING AT >2500 M

Silvia Ulrich^1^, Mona Lichtblau^1^, Esther I Schwarz^1^, Elisabeth Cajamarca^2^, Rodrigo Hoyos^2^

^1^University Hospital Zurich, Zurich, Switzerland, ^2^Hospital Carlos Andrade Marin, Quito, Ecuador

Over 80 Mio people worldwide live >2500 m, amongst which as least as many patients with pulmonary vascular disease (PVD) defined as pulmonary arterial or chronic thromboembolic pulmonary hypertension (PAH/CTEPH) as elsewhere. Whether PVD‐patients living at high altitude have altered disease characteristics due to hypobaric hypoxia is unknown. In a cross‐sectional study conducted at the Hospital Carlos Andrade Marin in Quito, Ecuador, located at 2840 m, we included prevalent ambulatory patients with PAH or CTEPH visiting the clinic 1/2022‐7/2023. We retrieved diagnostic right heart catheterisation data, treatment, and risk factors including NYHA functional class (FC), 6‐min walk distance (6MWD), and NT‐brain natriuretic peptide (BNP) at baseline and last follow‐up. We included 36 PVD‐patients (83% women, 32 PAH, 4 CTEPH, mean ± SD age 44 ± 13 years, living altitude 2831 ± 58 m revealing baseline values: PaO_2_ 8.2 ± 1.6 kPa, PaCO_2_ 3.9 ± 0.5 kPa, SaO_2_ 91 ± 3%, mean pulmonary artery pressure 53 ± 16 mmHg, pulmonary vascular resistance 16 ± 4 WU, 50% FC II, 50% FC III, 6MWD 472 ± 118 m, BNP 490 ± 823 ng/L. Patients were treated with sildenafil (100%), bosentan (33%), calcium channel blockers (33%), diuretics (69%), oxygen (nocturnal 53%, daytime 11%), for 1628 ± 1186 days. Last visit values FC (II 75%, III 25%), 6MWD of 496 ± 108 m, BNP of 576 ± 5774 ng/L. Compared to PVD‐registries, ambulatory PVD‐patients living >2500 m revealed similar blood gases and relatively low and stable risk factor profiles despite severe hemodynamic compromise, suggesting that favourable outcome is achievable for highlanders with PVD. Future studies should focus on long‐term outcomes in PVD‐patients dwelling >2500 m.

## A171 THE PREVALENCE OF PULMONARY HYPERTENSION IN POST‐TUBERCULOSIS AND ACTIVE TUBERCULOSIS POPULATIONS: A SYSTEMATIC REVIEW AND META‐ANALYSIS

Jennifer Kate Van Heerden^1,2^, Elizabeth Louw^3^, Friedrich Thienemann^4,5^, Mark Engel^6,7^, Brian Allwood^3^

^1^Nuffield Department of Surgical Sciences, University of Oxford, Oxford, UK, ^2^Wellcome Centre for Infectious Diseases Research in Africa, Institute of Infectious Disease and Molecular Medicine, University of Cape Town, Cape Town, South Africa, ^3^Division of Pulmonology, Department of Medicine, Faculty of Medicine and Health Sciences, Stellenbosch University and Tygerberg Hospital, Cape Town, South Africa, ^4^General Medicine and Global Health, Department of Medicine, and Cape Heart Institute, Faculty of Health Sciences, University of Cape Town and Groote Schuur Hospital, Cape Town, South Africa, ^5^Department of Internal Medicine, University Hospital Zurich, University of Zurich, Zurich, Switzerland, ^6^Department of Medicine, and Cape Heart Institute Faculty of Health Sciences, University of Cape Town, Cape Town, South Africa, ^7^South African Medical Research Council, Cape Town, South Africa

In addition to the permanent lung damage that frequently complicates active or previous pulmonary tuberculosis, pulmonary vascular disease is an under‐recognised complication of tuberculosis. The prevalence of tuberculosis‐associated pulmonary hypertension has not previously been quantified, resulting in an under‐appreciated burden of disease. We aimed to estimate the prevalence of pulmonary hypertension in post‐tuberculosis and active tuberculosis populations. In this systematic review and meta‐analysis, we searched PubMed/Medline, Cochrane Library, EBSCOhost, Scopus, African Journals Online, and Google Scholar, with no language restriction, for available literature published after 1950. Eligible studies described adult participants (≥16 years), with documented evidence of active or prior tuberculosis, diagnosed with pulmonary hypertension. Study quality was assessed using a risk of bias tool specifically developed for prevalence studies. Aggregate prevalence estimates with 95% confidence intervals (CI) were synthesised using a random‐effects meta‐analysis model, incorporating the Freeman–Tukey transformation. Subgroup analysis was conducted to ascertain prevalence estimates in specific patient populations. This study is registered with PROSPERO, reference number CRD42023395311. We identified 1452 unique records, of which 34 met our inclusion criteria. Twenty‐three studies, with an acceptable risk of bias and where pulmonary hypertension was diagnosed at right heart catheterisation or echocardiography, were included in the meta‐analysis. In post‐tuberculosis studies (14/23), the prevalence of pulmonary hypertension was 67.0% (95%CI 50.8–81.4) in patients with chronic respiratory failure; 42.4% (95%CI 31.3–54.0) in hospitalised or symptomatic patients; and 6.3% (95%CI 2.3–11.8) in non‐healthcare‐seeking outpatients (I² = 96%). There was a lower estimated prevalence of pulmonary hypertension in studies of populations with active tuberculosis [9.4% (95%CI 6.3–13.0), *I*² = 84%], and prevalence estimates varied from 7.0% (95%CI 1.3–16.4) (*I*² = 91%) in studies including outpatients only to 10.4% (95%CI 6.6–14.8) (*I*² = 80%) in studies including hospitalised patients. Our results highlight the significant burden of pulmonary hypertension in post‐tuberculosis and active tuberculosis populations. We emphasise the need for increased recognition of tuberculosis‐associated pulmonary hypertension and additional high‐quality prevalence data.

## A172 HEMODYNAMICALLY DERIVED RIGHT VENTRICULAR DIASTOLIC STIFFNESS PREDICTS MORTALITY IN PULMONARY ARTERIAL HYPERTENSION

Rebecca Vanderpool^1^, Zilu Liu^1^, Charles Fauvel^1^, Shili Lin^1^, Priscilla Correa‐Jaque^1^, Manreet Kanwar^2^, Jidapa Kraisangka^3^, Adam Perer^4^, Allen D. Everett^5^, Raymond Benza^6^

^1^The Ohio State University, Columbus, USA, ^2^Allegheny General Hospital, Pittsburgh, USA, ^3^Mahidol University, Thailand, ^4^Carnegie Mellon University, Pittsburgh, USA, ^5^The John Hopkins University, Baltimore, USA, ^6^Mount Sinai, New York City, USA

Survival in pulmonary arterial hypertension (PAH) is associated with right ventricular (RV) function rather than pulmonary vascular resistance (PVR). At present, RV function is not incorporated in current risk assessment models, and studies evaluating RV function are generally limited to single‐center studies. The aim was to assess hemodynamic measures of diastolic RV function in the context of multi‐center PAH clinical trials and to investigate sex‐associated differences. Baseline clinical and hemodynamic data were harmonized data from five PAH clinical trials (*n* = 2499 patients across COMPASS3, EARLY, GRIPHON, MAESTRO, and SERAPHIN). RV diastolic stiffness coefficient (*β*) and end‐diastolic elastance (Eed) were estimated by fitting the end‐diastolic pressure–volume relationship [*P* = α(*eVβ *− 1)]. Due to limitations in collected hemodynamics in clinical trials, RV end‐diastolic pressure was estimated by RA pressure, and cardiac output‐derived stroke volumes were used in the single‐beat pressure–volume analysis. Sex‐associated differences were investigated in Eed and advanced RV hemodynamics including arterial elastance (Ea) and RV stroke work (RVSW). The cohort was also split into four groups based on Eed quartiles (quartile 1: 0.09 ± 0.05 mmHg/mL, quartile 4: 0.25 ± 0.05 mmHg/mL, quartile 3: 0.46 ± 0.09 mmHg/mL, and quartile 4: 1.33 ± 0.85 mmHg/mL). Data are presented as mean ± standard deviation or median [interquartile range]. All‐cause mortality was used in survival analysis. Hemodynamic data was harmonized in *N* = 1764 patients with PAH (47 ± 16 years and 14% deaths (*n* = 249) with 1.7 [0.6–2.6] years follow‐up). Three quarters of the cohort are female (76.5%). In a subset of participants (*n* = 97), right atrial pressure significantly correlates with RV end‐diastolic pressure (*R* = 0.45, *p* < 0.001). In the whole cohort, the median Eed was 0.33[0.17–0.65] mmHg/m. NT proBNP increased from 502 [230–1067] ng/L in Eed_quartile 1 to 1351 [727–2560] ng/L in Eed_quartile 4. Six min walk distance significantly decreased from 390 [327–439] m in Eed_quartile 1 to 343 [268–407] m in Eed_quartile 4. Eed_quartile 4 had the highest mortality compared to other quartiles (log‐rank: *p* < 0.0001). When comparing across sexes, females (76.5%, *n* = 1350) had decreased diastolic stiffness compared to males (Eed: 0.32 [0.16–0.62] mmHg/mL vs. 0.39 [0.20–0.76] mmHg/mL, *p* < 0.05, respectively). Patterns of Eed‐associated changes in arterial elastance and RV stroke work are different in males and females. With regard to mortality, males and females had similar 1‐year mortality in Eed_quartiles 1 and 2. Survival in males and females start to deviate, with males having increased 1‐year mortality compared to females in Eed_quartiles 3 and 4. Right atrial pressure‐derived RV diastolic stiffness is a novel marker of right ventricular function that is significantly associated with mortality. The sex‐associated differences in Eed and mortality highlight the utility of Eed in combination with other key risk factors to identify novel RV function sub‐phenotypes in patients with PAH.

## A173 UNLOCKING THE CARDIO‐PROTECTIVE ROLE OF SMYD2 IN PULMONARY HYPERTENSION

Swathi Veeroju^1^

^1^Excellence Cluster Cardiopulmonary Institute, Giessen, Germany

Swathi Veeroju^1^, Baktybek Kojonazarov^1,2^, Argen Mamazhakypov^1^, Akylbek Sydykov^1^, Aysel Ashir^1^, Nabham Rai^1^, Sandra Breuils‐Bonnet^3^, Jochen Wilhelm^1^, Stefan Guenther^4^, Hossein Ardeschir Ghofrani^1^, Norbert Weissmann^1^, Thomas Braun^4^, Steeve Provencher^3^, Sebastien Bonnet^3^, Werner Seeger^1,2,4^, Tatyana Novoyatleva^1^* and Ralph T. Schermuly^1^*

^1^Department of Internal Medicine, Justus‐Liebig University Giessen, Excellence Cluster Cardio‐Pulmonary Institute (CPI), Member of the German Center for Lung Research (DZL), Giessen, Germany, ^2^Institute for Lung Health, Giessen, Germany, ^3^Pulmonary Hypertension and Vascular Biology Research Group, Institut Universitaire de Cardiologie et de Pneumologie de Québec, Université Laval, Department of Medicine, Québec, Canada, ^4^Max Planck Institute for Heart and Lung Research, Bad Nauheim, Germany

* Equal contribution

Right ventricle (RV) failure plays a detrimental role in the mortality of patients suffering from pulmonary arterial hypertension (PAH), yet our understanding of underlying molecular mechanisms driving RV failure in PAH remains unclear. We aimed to investigate the role of Smyd2 in adverse RV remodeling in PAH. Expression analysis of human RV tissues revealed that HIF‐1α and Smyd2 were downregulated in patients with decompensated RVs. Smyd2 showed a significant correlation with Tricuspid Annular Plane Systolic Excursion (TAPSE) in decompensated RV. Our hypoxia studies have shown that exposure to hypoxia‐induced Smyd2 expression in neonatal rat cardiomyocytes (CMs) and cardiac microvascular endothelial cells (CMECs). Interestingly, we found that silencing Smyd2 downregulated the expression of HIF‐1α under hypoxia. Through in silico analysis, we identified a Smyd2‐specific methylation motif within the HIF‐1α protein (PAS A domain), suggesting HIF‐1α as a potential substrate for Smyd2 methylation. Further analysis using in vitro methyltransferase assays and mass spectrometry analysis confirmed that Smyd2 methylates HIF‐1α at lysine (K94). Experiments with Smyd2 Y240F (catalytic dead mutant) and HIF‐1α K94R mutants provided further evidence that Smyd2 specifically targets K94 for HIF‐1α protein methylation. Smyd2 was found to facilitate hypoxia‐induced HIF‐1α K94 methylation and subsequent nuclear translocation of HIF‐1α. Silencing of Smyd2 in both Cand CMECs prevented hypoxia‐mediated HIF‐1α K94 protein methylation and stabilization. In vitro Matrigel assays indicated that conditioned media from Cwith Smyd2 overexpression enhanced the formation of loops, nodes, and tube junctions in human umbilical vein endothelial cells. To investigate the therapeutic potential of Smyd2 in RV failure, we performed AAV‐mediated Smyd2 overexpression in the pulmonary artery banding (PAB) mice model. We observed that Smyd2 overexpression significantly improved cardiac function with improved TAPSE, cardiac index, and stroke volume index. Histo‐morphometric analysis revealed decreased RV fibrosis and cardiomyocyte hypertrophy with a maintained capillary number. Plasma levels of Serpine E1 and Thrombospondin‐2 were reduced in PAB mice upon smyd2 overexpression. Additionally, RNA sequencing analysis of RV tissue from Smyd2‐overexpressing mice revealed a decrease in glycolytic pathways and an activation of cardioprotective genes such as Smad6, Neuregulin 1 (Nrg1), Ncam1, and Fgfr2, suggesting that the beneficial effects of Smyd2 are primarily caused by its impact on fibrosis and glycolysis. Our study demonstrates a pivotal role of Smyd2 for stabilizing HIF‐1α and facilitating cardioprotection in RV hypertrophy.

## A174 EFFECTS OF THE RENIN‐ANGIOTENSIN‐SYSTEM (RAS) INHIBITORS ON RIGHT VENTRICULAR FUNCTION IN PATIENTS WITH ACUTE PULMONARY EMBOLISM

Dingyi Wang^1^, Guohui Fan^1^, Zhenguo Zhai^1^, Yinong Chen^1^

^1^China‐Japan Friendship Hospital, Beijing, China

Right ventricular (RV) dysfunction is highly prevalent after pulmonary embolism (PE). Renin‐angiotensin‐system (RAS) inhibitors contribute to improving ventricular remodeling in patients with left ventricular (LV) dysfunction and reducing mortality in those with pulmonary hypertension. The aim of this study is to explore the efficacy of the RAS inhibitors on RV function in patients with acute PE. Patients with confirmed acute PE were retrospectively enrolled from China–Japan Friendship Hospital between August 1, 2016 and October 31, 2021. Patients were divided into two groups based on whether they were prescribed angiotensin‐converting enzyme inhibitors (ACEIs) or angiotensin receptor blockers (ARBs) for over 3 months due to comorbidities before the onset of acute PE. Demographics, comorbidities, risk stratification, echocardiographic and laboratory tests, and outcomes between two groups were analyzed. Sub‐group analyses were conducted among patients with hypertension or chronic cardiovascular diseases (including coronary artery disease, chronic heart failure, and atrial fibrillation). RV dysfunction was defined as follows: RV/LV diameter ratio >0.9 measured by echocardiography or computed tomographic pulmonary angiography (CTPA), tricuspid annular plane systolic excursion (TAPSE) < 17 mm, or pulmonary arterial systolic pressure (PASP) > 35 mmHg measured by echocardiography. Follow‐up parameters of echocardiography at 3 months, 6 months, and longer were collected and described. All the statistical analyses were performed by SPSS 25.0. A total of 291 patients with acute PE were included. Among them, 59 (20.3%) participants had been prescribed long‐term usage of ACEIs or ARBs before PE onset. During hospitalization, RV dysfunction was observed in 111 (38.1%) individuals. There was no statistically significant difference in risk stratification or prevalence of RV dysfunction between the two groups. However, TAPSE in ACEIs/ARBs group was higher compared to non‐ACEIs/ARBs group, regardless of whether comorbid with hypertension (20.69 mm vs. 17.21 mm, *p* = 0.023) or other cardiovascular diseases (20.68 mm vs. 15.97 mm, *p* = 0.018). In the subgroup analysis of patients with RV dysfunction, TAPSE in ACEIs/ARBs group was also higher (19.95 mm vs. 17.07 mm, *p* = 0.039), while PASP was lower (42 mmHg vs. 50 mmHg, *p* = 0.033). Follow‐up echocardiography was available in 54 patients with RV dysfunction at 3 months or longer. The overall improvement of parameters related to RV function in the ACEIs/ARBs treatment group was better than that in non‐ACEIs/ARBs group, although no statistically significant difference was observed. Patients who had background ACEIs/ARBs usage exhibited a reduced severity of RV dysfunction after acute PE during hospitalization, suggesting a potential protective impact of the RAS inhibitors on RV function. Those been prescribed the RAS inhibitors also showed better RV function during follow‐up period. Thus, the RAS inhibitors may be a choice for the prevention or treatment for RV dysfunction after acute PE with further investigation warranted.

## A175 PULMONARY ENDOTHELIAL INFLAMMATION DYSREGULATES SRC FAMILY KINASE SIGNALING TO INCREASE COLLAGEN 22 PRIOR TO THE DEVELOPMENT OF SEVERE PULMONARY HYPERTENSION

Bradley Wertheim^1^, Rui‐Sheng Wang^2^, Elena Arons^1^, Nirmal Sharma^1^, Kathleen Haley^1^, George Alba^6^, Andriy Samokhin^3,5^, Robert Padera^4^, Joseph Loscalzo^5^, Bradley Maron^3,5^

^1^Department of Medicine, Division of Pulmonary and Critical Care Medicine, Brigham and Women's Hospital, Harvard Medical School, Boston, USA, ^2^Department of Medicine, Channing Division of Network Medicine, Brigham and Women's Hospital, Harvard Medical School, Boston, USA, ^3^Department of Medicine, University of Maryland School of Medicine and the University of Maryland‐Institute for Health Computing, Baltimore, USA, ^4^Department of Pathology, Brigham and Women's Hospital, Harvard Medical School, Boston, USA, ^5^Department of Medicine, Division of Cardiovascular Medicine, Brigham and Women's Hospital, Harvard Medical School, Boston, USA, ^6^Department of Medicine, Division of Pulmonary and Critical Care Medicine, Massachusetts General Hospital, Harvard Medical School, Boston, USA

In end‐stage pulmonary arterial hypertension (PAH), inflammation is associated with endothelial dysfunction, fibrotic vascular remodeling, and right ventricular failure. Data on the molecular mechanisms by which inflammation regulates fibrosis signaling pathways in early‐stage PAH are lacking but may be important for developing treatments that prevent progression to end‐stage disease. We hypothesized that in early‐stage PAH, inflammation induces pulmonary artery endothelial cell (PAEC) dysfunction that leads to vascular fibrosis before hemodynamic evidence of severe pulmonary hypertension (PH). Sprague Dawley rats were administered vehicle (V) control or monocrotaline (60 mg/kg, day 0) to induce inflammatory PAH. Cardiac catheterization, tissue harvest, and PAEC transcriptomic analysis occurred on days 15 (early PAH) or 23 (advanced PAH). Compared to control, the right ventricular systolic pressure (RVSP) was increased incrementally in early PAH and substantially in advanced PAH (25.2 ± 1.2 vs. 33.2 ± 2.7 vs. 67.4 ± 8.3 mmHg, *N* = 6/condition, *P* = 6.5 × 10^−5^), and was directionally similar to pulmonary arteriolar collagen quantity by trichrome stain (56.5 ± 2.2 vs. 231 ± 27 vs. 680 ± 58 arbitrary units, *N* = 6/condition, *P* = 1.3 × 10^−8^). Network analysis of differentially expressed PAEC genes identified C‐terminal src kinase (Csk), an inhibitor of the oncoprotein Src, as a key fibrosis node in early‐stage PAH in silico. Next, human PAECs (HPAECs) were transfected with siRNA against Csk (siCsk) or scrambled control (Scr), and then treated with V‐control or an inflammatory (INFLM) stimulus (lipopolysaccaride, 0.03 mg/mL + interferon‐γ, 50 ng/mL + interleukin‐1β, 50 ng/mL) for 24 h. By bulk RNA‐seq analysis, INFLM was enriched for endophenotypes reported in PAH patients, including epithelial‐to‐mesenchymal transition, IL‐6/JAK/STAT‐3 signaling, and hypoxia (false discovery rate [FDR] <0.0001 vs. V‐control). However, INFLM also upregulated multiple collagen mRNAs, including collagen 22 (Col22A1, log2fold‐change[FC] 4.6, FDR = 1.05 × 10^−132^), which is reported in fibroproliferative pathophenotypes (e.g., malignancy and scleroderma), but unstudied in PAH. Transfection of HPAECs with siCsk increased Col22A1 mRNA expression by 37% versus siScr in INFLM‐treated cells (FDR = 3.1 × 10^−6^, *N* = 6). In HPAECs treated with INFLM + adenovirus to overexpress Csk, we observed a 42% decrease in Col22A1 protein expression (*p* < 0.0001) versus V‐control, confirming inhibition of Col22A1 by Csk. Treatment of HPAECs with the Src inhibitor SU‐6656 (2 μM) for 24 h attenuated INFLAM‐dependent upregulation of Col22A1 by 55% compared to INFLM + DMSO (*p* < 0.014, *N* = 4), suggesting that INFLM impairs Csk‐Src signaling to regulate Col22A1. Compared to control, Col22A1 expression increased progressively in early and advanced PAH by 1.8‐ (*p* < 0.008, *N* = 6) and 9.4‐fold (*p* = 0.005), respectively. To explore a clinical correlate to our findings, we focused next on human PAH paraffin‐embedded lung sections (obtained at autopsy or transplant) and observed that pulmonary arterial Col22A1 was upregulated by 22.7‐fold (*p* < 0.03, *N* = 4) versus discarded lung donor tissue. Inflammation promotes Col22A1 expression in HPAECs in vitro, which is regulated by the Csk‐Src axis. Increased Col22A1 is observed in inflammatory PAH arterioles before the development of severe PH, as well as in human arterioles from patients with end‐stage PAH. Identifying Csk‐Src as a novel target to mitigate vascular fibrosis may have important therapeutic implications for early‐stage PAH.

## A175 INCREASES IN PEAK STEPS MEASURED BY ACTIGRAPH AFTER ADDING ORAL TREPROSTINIL: RESULTS FROM THE ADAPT REGISTRY

James White^1^, Scott Seaman^2^, James Gagermeier^3^, Andrew Wang^2^, Meredith Broderick^2^

^1^University Of Rochester Medical Center, Rochester, USA, ^2^United Therapeutics Corporation, Research Triangle Park, USA, ^3^Loyola University, Maywood, USA

Pulmonary arterial hypertension (PAH) patients have low physical activity time when measured with wrist‐worn activity trackers in the home setting. Even though changes in activity seem like a feasible outcome in PAH, there are no controlled pharmacologic studies showing increased activity after adding therapy. Peak steps, a measure of capacity for activity, is a novel approach to look for changes in activity in the home setting. Peak steps have been shown to increase after adding FDA‐approved therapy in PAH as well as during a behavioral intervention study. The ADAPT registry evaluated activity changes using a wrist‐worn ActiGraph after adding open‐label oral treprostinil (TRE) therapy. In Group 1 PH patients already taking approved therapy, activity was measured at baseline and again 2–12 months later in the ADAPT registry, a multicenter, prospective, observational study evaluating oral TRE. Study visits were associated with routine clinic visits. Wear time was 10 days per study visit. Peak steps were calculated by rank ordering steps by 1‐min epochs (interval) per day (whether or not contiguous). Peak 5 min was defined as the number of steps in the most active 5 min during the day, and were not necessarily contiguous. Daily values were averaged per study visit. Wear time of at least 3 days of 10 h per day was included for analysis. We analyzed the top 1–20 min of steps (e.g., 3, 5, 10, and 20 min, whether or not contiguous) and daily steps. Continuous variables were compared using the Wilcoxon rank test and Spearman's rank correlation coefficient.

Baseline activity was measured in 19 patients from 11 sites. Follow‐up occurred during the first year of the COVID‐19 pandemic, which limited in clinic testing. The median time of follow‐up was 6 months after baseline in 14 patients. The total daily TRE dose was 12 mg. Baseline 6‐min walk distance was 382 m with an emPHasis‐10 score of 17. Four patients were functional class III; 10 were FC I/II. At follow‐up, there was no change in mean daily steps from baseline to follow‐up (1432 vs 2199 steps, *p* = 0.30). Peak 5 min (284 vs 358 steps, *p* = 0.02) and peak 20 min (784 vs 1007 steps, *p* = 0.007) increased after adding TRE. There was a strong correlation with peak 20 min and 6MWD (*r* = 0.73, *p* < 0.001) and emPHasis‐10 (*r* = −0.67, *p* < 0.001). Because of limited in‐person clinical testing during the pandemic, changes in 6‐min walk distance, risk score, and EmPHasis‐10 score could not be compared to actigraphy parameters. In a multicenter observational study, ‘peak steps’ increased after adding oral TRE, while mean daily steps were unchanged. Mean daily steps are likely influenced by many factors (behavior, COVID limiting activity); detecting therapy‐associated change may be difficult. Changes in ‘peak steps’ may be a measure of capacity correlating more with physiologic improvements.

## A176 DEFICIENT RNA EDITING INDUCES ABERRANT PULMONARY ARTERY ENDOTHELIAL INNATE IMMUNITY ACTIVATION IN PULMONARY ARTERIAL HYPERTENSION

Chen‐shan Woodcock^1,2^, Giovanni Maroli^4,5^, Hyunbum Kim^6^, Ying Tang^1,2^, Yi Yin Tai^1,2^, Satoshi Okawa^1,7,8^, Shu‐Ting Cho^7^, Robert Lafyatis^3^, Caroline Chauvet^9^, Tatiana Kudryashova^1,10^, Elena Goncharova^10^, Thomas Bertero^9^, Ke Yuan^6^, Soni Pullamsetti^4,5^, Stephen Chan^1,2^

^1^Center for Pulmonary Vascular Biology and Medicine, Pittsburgh Heart, Lung, and Blood Vascular Medicine Institute, University of Pittsburgh School of Medicine, Pittsburgh, USA, ^2^Division of Cardiology, Department of Medicine, University of Pittsburgh School of Medicine and University of Pittsburgh Medical Center, Pittsburgh, USA, ^3^Division of Rheumatology, Department of Medicine, University of Pittsburgh School of Medicine and University of Pittsburgh Medical Center, Pittsburgh, USA, ^4^Department of Internal Medicine, Member of the German Center for Lung Research (DZL), Member of the Cardio‐Pulmonary Institute (CPI), Justus Liebig University, Giessen, Germany, ^5^Max Planck Institute for Heart and Lung Research, Member of DZL, Member of CPI, Germany, ^6^Division of Pulmonary Medicine, Boston Children's Hospital, Harvard Medical School, Boston, USA, ^7^Department of Computational and Systems Biology, University of Pittsburgh School of Medicine, Pittsburgh, USA, ^8^McGowan Institute for Regenerative Medicine, University of Pittsburgh School of Medicine, Pittsburgh, USA, ^9^Université Côte d'Azur, CNRS, IPMC, Valbonne, France, ^10^Lung Center, Division of Pulmonary, Critical Care and Sleep Medicine, University of California, Davis School of Medicine, Sacramento, USA

Pulmonary arterial hypertension (PAH) is a substantial health issue with no cure, characterized by elevated pulmonary artery (PA) resistance and vascular remodeling. Early apoptosis of pulmonary artery endothelial cells (PAECs) is a key driver of vascular remodeling in the pathogenesis of PAH, but its regulation is poorly defined. Adenosine‐to‐Inosine (A‐to‐I) RNA editing is one posttranscriptional machinery that is critical for controlling gene regulation and RNA hemostasis. Adenosine deaminase acting on RNA 1 (ADAR1) is an RNA editing enzyme that converts adenosine to inosine (A‐to‐I) in RNA transcripts. While emerging evidence indicates reduced levels of ADAR1 are observed in the endothelial layer of human PAH lungs and lack of cellular ADAR1‐mediated RNA editing stimulates aberrant cytosolic innate immunity response leading to apoptosis, the exact RNA editing targets and molecular mechanisms regulating pulmonary endothelial survival and immunity are unknown. ADAR1 was downregulated in the pulmonary vascular endothelium and lung tissue of human and rodent PAH. A significantly reduced level of A‐to‐I RNA editing was observed in human PAH lungs. By RNA sequencing of PAECs after ADAR1 knockdown, we identified the circadian gene Nocturnin (NOCT) as a direct ADAR1 target. NOCT transcripts were found to carry two active A‐to‐I RNA editing sites in the 3'‐untranslated region. ADAR1 deficiency or A‐to‐I RNA editing inactivity stabilized NOCT transcripts. By single‐cell RNA sequencing of PAH lung tissues, NOCT RNA editing was found to be reduced, while NOCT transcript and protein levels were increased. Correspondingly, ADAR1 silencing in vitro increased NOCT expression, thus activating RNA‐sensing innate immunity signaling and PAEC apoptosis; NOCT silencing reversed these changes. Forced NOCT expression phenocopied this effect, upregulating RNA‐sensing innate immunity signaling molecules melanoma differentiation‐associated gene 5 (MDA5), interferon regulatory factor 7 (IRF7), and interferon‐stimulated genes (ISGs), and increasing apoptosis in PAECs. In vivo, the ADAR1 inhibitor 8‐aza‐adenosine (Aza) promoted worsened indices of pulmonary hypertension (PH) and upregulated endothelial NOCT and IRF7 in chronically hypoxic PH mice. Notably, genetic deletion of NOCT reversed such PH induction by ADAR1 inhibitor Aza, emphasizing the crucial role of NOCT in ADAR1‐mediated pathogenesis. In summary, ADAR1 deficiency promotes NOCT gene dysregulation that leads to induce aberrant innate immunity signaling, PAEC apoptosis, and PAH pathogenesis. These findings provide substantial impetus to target the endothelial ADAR1‐NOCT axis for more effective diagnostics and therapeutics for PAH.

## A177 IMPACT OF INOS‐ABLATION IN ENDOTHELIAL CELLS ON THE DEVELOPMENT OF CIGARETTE SMOKE‐INDUCED PULMONARY HYPERTENSION AND EMPHYSEMA

Cheng‐Yu Wu^1^, Marija Gredic^1^, Stefan Hadzic^1^, Siddartha Doswada^1^, Anis Cilic^1^, Edma Loku^1^, Vidya Lakshmi^1^, Werner Seeger^1^, Friedrich Grimminger^1^, Hossein‐Ardeshir Ghofrani^1^, Ralph T Schermuly^1^, Natascha Sommer^1^, Simone Kraut^1^, Norbert Weissmann^1^

^1^Cardio‐Pulmonary Institute (CPI), Universities of Giessen and Marburg Lung Center (UGMLC), Member of the German Center for Lung Research (DZL), Justus Liebig University (JLU), Giessen, Germany

Chronic obstructive pulmonary disease (COPD) remains a leading cause of death worldwide. Major causes of COPD are chronic exposure to toxic gases and/or fine particulate matter from cigarette smoke (CS) and polluted air. COPD comprises several disease phenotypes including chronic bronchitis and pulmonary emphysema. Notably, up to 90% of COPD patients show pulmonary vascular involvement leading to pulmonary hypertension (PH). It has been suggested that pulmonary vascular remodelling, a hallmark of PH, can occur before development of COPD in smokers, as shown in animal model of CS‐induced emphysema. One key pathomechanism is triggered by nitrosative/oxidative stress. Previously, inducible nitric oxide synthase (iNOS) was identified as a key enzyme of PH and emphysema development upon CS exposure in mice. Interestingly, iNos‐ablation in bone marrow‐derived (BMD) cells prevented CS‐induced PH development, whereas deletion of iNOS in non‐BMD cells protected mice from CS‐induced emphysema. Elevated iNOS levels were observed in pulmonary vessels of CS‐exposed mice and human COPD lungs. However, the contribution of increased iNOS expression in lung vessels to CS‐induced emphysema and PH development remains unresolved. Against this background, we isolated endothelial cells from mouse lung vessels and confirmed that iNOS expression was increased upon lipid component of CS (l‐CSE) exposure. Next, we aimed to determine the role of iNOS in pulmonary endothelial cells during CS‐induced PH and emphysema development. We used transgenic animals with iNos deleted in Tie2+ cells and exposed them to CS for 8 months. The results showed that after 8 months of CS exposure, Tie2+ cell‐specific iNos‐deficient mice had slightly lower right ventricular systolic pressure compared to control mice. In addition, lung compliance was increased in CS‐exposed mice, independent of iNos‐deletion in Tie2+ cells. Further studies will focus on examining the alterations in lung vasculature and alveolar structure of these mice, and pinpointing the downstream molecular pathways affected by iNos‐ablation in Tie2+ cells. This project is funded by the Deutsche Forschungsgemeinschaft (DFG, German Research Foundation) – Projektnummer 268555672 – SFB 1213, A07.

## A178 SCREENING AND EVALUATION OF SELECTED APPROVED DRUGS AND KINASE MODULATORS AS POTENTIAL INHIBITORS OF AK4

Magdalena Wujak^1,2^, Tomasz Bronk^1^, Mateusz Fido^3^, Marietta Podbielska^1^, Mateusz Kwiatkowski^4^, Michał Marszałł^1^, Joanna Kowalska^3^, Norbert Weissmann^2^, Anna Kozakiewicz‐Piekarz^5^

^1^Department of Medicinal Chemistry, Collegium Medicum in Bydgoszcz, Faculty of Pharmacy, Nicolaus Copernicus University in Toruń, Bydgoszcz, Poland, ^2^Universities of Giessen and Marburg Lung Center (UGMLC), Member of the German Center for Lung Research (DZL), Excellence Cluster Cardio‐Pulmonary Institute (CPI), Justus‐Liebig University, Giessen, Germany, ^3^Division of Biophysics, Institute of Experimental Physics, Faculty of Physics, University of Warsaw, Warsaw, Poland, ^4^Department of Plant Physiology and Biotechnology, Faculty of Biological and Veterinary Sciences, Nicolaus Copernicus University in Toruń, Toruń, Poland, ^5^Department of Biomedical and Polymer Chemistry, Faculty of Chemistry, Nicolaus Copernicus University, Toruń, Poland

Pulmonary hypertension (PH) is a progressive and incurable disease characterized by pulmonary vascular remodelling and lung vasculopathy. Mitochondrial dysfunction and metabolic reprogramming are associated with the progression and occurrence of PH and may point out new therapeutic targets and strategies. Similar underlying pathological mechanisms occur in cancer, which itself or its treatment may cause PH. Mitochondrial adenylate kinase 4 (AK4) is a nucleotide monophosphate kinase catalyzing the phosphoryl transfer between adenine nucleotides. AK4 has emerged as a diagnostic and prognostic marker and therapeutic target for different types of cancer. In non‐small cell lung cancer, it promotes glycolytic shift and tumor progression via AMPK inhibition and HIF‐1α stabilization. Our previous study showed that AK4 is increased in pulmonary vessels of patients with idiopathic pulmonary arterial hypertension and regulates HIF‐1α and Akt signaling pathways to drive a pro‐proliferative and glycolytic phenotype of pulmonary arterial smooth muscle cells. RNA interference or genetic engineering are the only available strategies to study the pathophysiological role of AK4 in human diseases. The discovery of the first AK4 inhibitors and development of safe pharmacological approaches could vastly accelerate basic and translational research on the AK4 therapeutic potential in PH. Finally, a recent study reported that a profound deficiency of AK1 is associated with impaired ventricular energetics and maladaptive right ventricular remodelling. This finding highlights the concept of developing inhibitors that specifically target AK4 without affecting AK1 function. Against this background, we screened a hand‐picked collection of approved drugs and kinase modulators for their potential inhibitory activity against human AK4. For this purpose, we established a fast and efficient system for the production and purification of this enzyme. Two AK activity assays were developed for compound library screening, using high‐performance liquid chromatography and luciferin/luciferase‐based assay. Among clinically approved drugs, afatinib, regorafenib, and suramin demonstrated more than 50% inhibition of the AK4 enzymatic activity at 20 µM compound concentration in the presence of ADP as substrate. Suramin exhibited the highest inhibitor potency (IC50 of 4.97 ± 0.45 μM) with 90% inhibition at 25 μM concentration. Molecular docking was applied to predict the possible binding sites of suramin to AK4. We found that suramin did not bind to the active site of AK4, indicating other than a competitive mechanism of inhibition. In addition, drug molecules from both predicted populations interacted with amino acid residues of the LID domain, which is the most structurally variable domain in human adenylate kinases. Surprisingly, suramin inhibited AK1 with comparable efficiency, thus excluding it as a selective AK4 modulator. Screening of the protein kinase inhibitor collection showed that more compounds inhibited AK4 than AK1. Strikingly, the most differential effects in AK1 and AK4 inhibition were related to ATP competitive inhibitors, including p38 MAPK and Src family kinase inhibitors. Further studies are needed to assess the inhibitor selectivity against other AK isoenzymes and identify a new type of noncompetitive AK4 modulators. This research was supported by The National Science Centre (Poland) [grant No. 2017/25/B/ST4/00376] and The Initiative of Excellence – Research University Programme [grant No. 67/2023/Grants4NCUStudents].

## A179 NEUTROPHIL EXTRACELLULAR TRAPS INDUCED ENDOTHELIAL TO MESENCHYMAL TRANSITION AS A POTENTIAL MOLECULAR MECHANISM IN HYPOXIC PULMONARY HYPERTENSION

Yi Ye^1,2^, Song Yu^3^, Ri‐Li Ge^1,2^, Tana Wuren^1,2^

^1^Research Center for High Altitude Medicine, Qinghai University, Xining, China, ^2^Qinghai Provincial Key Laboratory for Application of High‐Altitude Medicine, Xining, China, Xining, China, ^3^Affiliated Hospital of Southwest Jiaotong University, The General Hospital of Western Theater Command, Chengdu, China

Chronic exposure to hypoxia, whether due to residence at high altitudes or underlying diseases, has emerged as a significant cause and promoter of pulmonary hypertension. During prolonged exposure to hypoxia, the infiltration of circulating immune cells and the expression of pro‐inflammatory factors contribute to immune inflammatory dysregulation in the pulmonary arteries. Inflammation and endothelial‐to‐mesenchymal transition (EndMT) are notable features of endothelial dysfunction in hypoxic pulmonary hypertension (HPH). Neutrophil extracellular traps (NETs), which are produced during neutrophil death, have been implicated in the pathogenesis of multiple vascular disorders, yet their role in HPH remains understudied. To address this gap, we aimed to uncover evidence of NETs formation, also called NETosis, in pulmonary hypertension and explore the potential protective effects of cl‐amidine, a potent inhibitor of NETs, against EndMT in HPH mice. Plasma and serum samples were collected from patients with HPH to measure circulating neutrophils and markers of NETs. To further investigate the underlying mechanisms, two animal models were utilized, the chronic hypoxia mouse model and the Sugen/hypoxia mouse model. Followed by assessing the efficacy of CI‐amidine, an inhibitor of NETs, in reversing pulmonary vascular remodeling. Lung tissues were harvested and subjected to flow cytometry and ELISA testing to determine NETosis. We further examined the protein levels of endothelial and mesenchymal markers using western blot analysis to confirm the induction of EndMT in HPH by NETosis. Patients with HPH showed elevated levels of circulating neutrophils and NETs markers, which were similarly observed in HPH mice with increased neutrophils and NETosis. HPH mice exhibited significant hemodynamic changes and extensive pulmonary vascular remodeling. Furthermore, western blot analysis and IHC staining confirmed increased endothelial cells transition to a mesenchymal phenotype. Notably, the administration of a specific NETs inhibitor via intraperitoneal injection successfully reduced neutrophil infiltration and effectively prevented and reversed the pulmonary hypertensive phenotype, as well as attenuated expression of EMT markers. NETs may play a contributory role in the vascular pathology of HPH, characterized by the activation of local pro‐inflammatory processes and endothelial dysfunction through EndMT. Inhibiting NETosis may provide the novel therapeutic target for HPH management.

## A180 THERAPEUTIC IMPLICATIONS OF CORRECTING PULMONARY VASCULAR RESISTANCE FOR BLOOD VISCOSITY IN PEDIATRIC PULMONARY ARTERIAL HYPERTENSION

Zhuoyuan Xu^1^, Hong Gu^1^, Yihua He^1^

^1^Beijing Anzhen Hospital, Capital Medical University, Beijing, China

Pulmonary vascular resistance (PVR) is essential for diagnosing and managing pulmonary arterial hypertension (PAH). As hemoglobin levels are commonly abnormal in PAH patients, blood viscosity may be abnormal and may affect PVR. To assess PVR corrected for blood viscosity (cPVR) and evaluate how the correction for blood viscosity affects treatment decisions in a large group of pediatric PAH patients. All pediatric patients undergoing right heart catheterization at a tertiary center were included. Blood viscosity was calculated according to the Hutton formula, and PVR corrected for blood viscosity. Overall, 742 children (median age 6.2 years; 63.7% female) were included. Of these, 115 patients had idiopathic PAH, 82 closed shunt defects, and 545 had PAH with patient shunt lesions. Median viscosity was 11.0 units (IQR 9.4–13.6). Uncorrected median PVR was 6.6 WU (IQR 2.0–15.6 WU), and median PVR corrected for blood viscosity was 7.3 WU (IQR 2.5–14.9 WU). In 271 patients (36.6%), corrected PVR changed by ≥2 WU compared to raw measured PVR (33.4% of shunt lesions vs. 45.2% of non‐shunt lesion patients). In the CHD PAH patients with patent shunts requiring decisions on shunt closure, 14% moved above or below the threshold of 5 WU, and 40% crossed the threshold of 10 WU after correcting for blood viscosity. Pediatric PAH patients commonly have abnormal blood viscosity, and this significantly affects PVR. Correcting PVR for viscosity has major potential therapeutic implications and should be included in the routine decision‐making process, especially for anemic and erythrocytotic patients.

## A181 INVESTIGATION ON THE PATHOGENIC ROLE OF IL‐17 MEDIATED ACTIVIN RECEPTOR ACTIVATION IN CONNECTIVE TISSUE DISEASE‐ASSOCIATED PULMONARY ARTERIAL HYPERTENSION

Jun Yang^1^, Shuliang Jing^1^, Junyan Qian^2^, Jingyuan Zhang^1^, Lie Wang^1^

^1^Zhejiang University School Of Medicine, Hangzhou, China, ^2^Peking Union Hospital, Beijing, China

Autoimmune diseases, such as connective tissue diseases, including systemic lupus erythematosus, are recognized by increased autoantibodies, associated pulmonary arterial hypertension (PAH), and numerous complications that contribute to a reduction in survival rates. TGF‐β family ligand Activin A induces CD4 + T cells to differentiate into pathogenic Th17 cells and secrete IL‐17, which further promotes tissue inflammation. However, its involvement in the Th17‐related mechanism of pulmonary vascular disease injury remains elusive. Elisa assay showed that the increased levels of IL‐17 released from CD4 + T cell and Activin A in peripheral blood and lung tissue from connective tissue disease‐associated PAH (CTD‐PAH) patients, which further promote the activation of target genes such as CTGF in pulmonary vascular endothelial cells. In the present study, we used pristane along with hypoxia to induce the CTD‐PH mouse model. Compared to the control group, the CTD‐PH mice had elevated levels of autoantibodies, right ventricular systolic pressure, and total pulmonary vascular resistance index. We applied a novel Activin receptor inhibitor WJ to treat the mice and found significantly improved hemodynamic indices including enhanced cardiac output, and reduced pulmonary vascular resistance. Additionally, the expression of IL‐17 in lung tissue and serum was also significantly reduced upon treatment. In vitro studies showed that hPVECs underwent EndoMT (endothelial‐to‐mesenchymal transition) with the stimulation of IL‐17 and Activin A. When the endothelial cells were treated with inhibitor WJ, they had decreased expression of the CTGF, Col1α1, and reduced inflammatory factors gene IL‐6. In conclusion, this study provides a potential treatment for connective tissue disease‐associated pulmonary hypertension by investigating the pathogenesis of the disease.

## A182 RIGHT HEART FAILURE WITH PRESERVED EJECTION FRACTION: A NEW CLINICAL PHENOTYPE WITH SYMPTOMATIC AND PROGNOSTIC RELEVANCE

Athiththan Yogeswaran^1^, Zvonimir Rako^1^, Hossein‐Ardeschir Ghofrani^1,2^, Bruno Brito da Rocha^1^, Daniel Zedler^1^, Werner Seeger^1^, Selin Yildiz^1^, Henning Gall^1^, Nils Kremer^1^, Robert Naeije^3^, Manuel Jonas Richter^1^, Khodr Tello^1^

^1^Department of Internal Medicine, Justus‐Liebig‐University Giessen, Universities of Giessen and Marburg Lung Center (UGMLC), Member of the German Center for Lung Research (DZL), Giessen, Germany, ^2^Department of Pneumology, Kerckhoff Heart, Rheuma and Thoracic Center, Bad Nauheim, Germany, Bad Nauheim, Germany, ^3^Erasme University Hospital, Brussels, Belgium

Right heart failure (rHF) is an important contributor to symptoms and mortality in pulmonary hypertension (PH). Despite extensive studies to right ventricular (RV) systolic function, the evaluation of RV diastolic function has been comparatively neglected due to its complexity. However, our hypothesis is that RV diastolic dysfunction is clinically important, independently of RV systolic function. Thus, we introduce a novel clinical phenotype termed “right heart failure with preserved ejection fraction” (rHFpEF). We included all consecutive patients suspected of having PH included in the EXERTION study. Comprehensive assessments involved both resting and exercise right heart catheterization, incorporating pressure‐volume loop measurements, and echocardiography. We performed correlation analyses, univariate and multivariate regression analysis, and receiver operating curves to explore the clinical relevance of rHFpEF. Kaplan–Meier analyses were conducted to assess clinical worsening. A total of 61 patients were included, 66% of whom were female. Multivariate binomial regression showed that only Eed is independently associated with dyspnea on exertion; notably, RV contractility and RV‐pulmonary artery coupling were not. Therefore, we defined patients with RV‐EF ≤ 37% as rHFrEF (reduced EF) and patients with preserved EF but impaired diastolic reserve on exercise as rHFpEF; all other patients were classified as controls. rHFpEF patients had less severe impairment in pulmonary hemodynamics compared to rHFrEF patients. However, rHFpEF patients still had significant dyspnea on exertion, impaired ESC/ERS risk, and significantly higher clinical worsening rates. Using echocardiography, rHFpEF could be diagnosed with high specificity and positive predictive values (92% and 88%, respectively). Taken together, rHFpEF is a novel phenotype based on exercise diastolic dysfunction with impaired diastolic reserve with high clinical relevance as it appears to be the major determinator of dyspnea. In addition, rHFpEF is associated with adverse events, underscoring the prognostic relevance of this new clinical phenotype.

## A183 NEUROINFLAMMATION MEDIATES COGNITIVE DYSFUNCTION IN PULMONARY HYPERTENSION

Ping Yuan^1^

^1^Department of Cardio‐Pulmonary Circulation, Shanghai Pulmonary Hospital, School of Medicine, Tongji University, Shanghai, China

Recently, we first identified cognitive impairment in patients with pulmonary hypertension (PH), which was associated with the occurrence and outcomes of PH. However, the underlying mechanism remains uncertain. The functions and histopathologis of MCT‐PH rat brains were examined by behavior tests (Morris Water Maze (MWM), Y maze (YM), open field test (OFT)) and molecular biological investigations, respectively. PH rats had more platform crossover numbers in MWM, fewer alternation numbers in YM, and more central zone entries in OFT than controls (all *p* < 0.001, Figure 1). There were moderate to severe correlations between the animal performances and right ventricular systolic pressure and Fulton index (all *p* < 0.05). The levels of vascular marker CD31 and that of pro‐inflammatory factors were significantly down‐ and upregulated, and hyperactivation of Iba1+ microglia in PH rat cortex. The study confirmed PH rat brains had cerebral small vessel atrophy and neuroinflammation, which may be the potential mechanism for PH‐induced cognitive impairment.

## A184 THE GLOBAL PREVALENCE OF PULMONARY HYPERTENSION – RATIONALE AND STUDY DESIGN

Katarina Zeder^1,2,3^, Ana O Mocumbi^4,5^, Judith Namuyonga^6,7^, Veranyuy Ngah^8^, Peter Nyasulu^8,9^, Geoff Strange^10,11,12,13^, Simon Stewart^5,14^, Bradley A Maron^2,3,15^

^1^Ludwig Boltzmann Institute for Lung Vascular Research and Division of Pulmonology, Department of Internal Medicine, Medical University of Graz, Graz, Austria, ^2^Division of Cardiovascular Medicine and Institute for Health Computing, University of Maryland, School of Medicine, Baltimore, USA, ^3^Division of Cardiovascular Medicine, Brigham and Women's Hospital and Harvard Medical School, Boston, USA, ^4^Universidade Eduardo Mondlane, Maputo, Mozambique, ^5^Instituto Nacional de Saúde, Marracuene, Mozambique, ^6^Department of Paediatric Cardiology, Uganda Heart Institute, Kampala, Uganda, ^7^Department of Paediatrics and Child Health, Makerere University College of Health Sciences, Kampala, Uganda, ^8^Division of Epidemiology and Biostatistics, Department of Global Health, Faculty of Medicine and Health Sciences, Stellenbosch University, Stellenbosch, South Africa, ^9^Division of Epidemiology and Biostatistics, School of Public Health, Faculty of Health Sciences, University of the Witwatersrand, Johannesburg, South Africa, ^10^Department of Cardiology, Royal Prince Alfred Hospital, Sydney, Australia, ^11^Heart Research Institute, Sydney, Australia, ^12^School of Medicine, The University of Notre Dame, Fremantle, Australia, ^13^Faculty of Medicine and Health, University of Sydney, Sydney, Australia, ^14^Institute for Health Research, University of Notre Dame Australia, Fremantle, Australia, ^15^Divisions of Cardiology and Pulmonary and Critical Care Medicine, VA Boston Healthcare System, West Roxbury, USA

Pulmonary hypertension (PH) is a heterogenous but specific disease that frequently complicates various diseases that are known to play a crucial role in global health burden. PH is an independent driver of increased mortality in these diseases and thus global health burden. Despite the clinical importance of PH to huge populations worldwide, contemporary estimates on the global prevalence of “all cause” PH are lacking and therefore PH is currently not listed as a major cause of cardiovascular mortality by the World Health Organization (WHO), American Heart Association, American College of Cardiology or the Center of Disease Control and Prevention. This knowledge gap is due, in part, to a pervasive misconception among many global health disease researchers that PH is synonymous with pulmonary arterial hypertension, which, is a (very) rare form of PH. Furthermore, current available data on the prevalence of PH is limited by data based mainly on right heart catheterization (RHC), a modality that is invasive and lacks broad availability worldwide. We hypothesize that due to sparse data, there is a gross underestimation of the actual prevalence of worldwide PH, which is akin to rates of other common diseases including systemic hypertension, ischemic heart disease, stroke, and general respiratory disorders. This is a global collaborative study. The globe will be divided into 10 Earth Regions (North America+Canada, Central America, Latin America, Western Europe, Eastern Europe, North Africa+Middle East, Sub‐Saharan Africa, East Asia, South Asia, Australia+Oceania). We will perform independent systematic literature searches according to PRISMA guidelines in 10–15 selected countries per region. Countries will be chosen based on population size and socioe‐conomic index. For each country, we will assess the prevalence of PH risk diseases (based on the five WHO PH groups) via literature search. Then, the prevalence of PH in each of these risk diseases will be assessed. We will search the databases Pubmed (Medline), Embase, Web of Science, and LILACS for original studies in humans of all ages without language restriction from 1973 (first definition of PH) until December 31, 2023. Three search strings will be used independently for each database: (1) MESH [hypertension, pulmonary] AND “name of the country”, (2) “echocardiogr*” AND “country”, (3) “right heart cath*” OR “pulmonary cath*” AND “country”. We will include all studies that report on PAP either by echocardiography or RHC. Animals studies, basic research, or where PH is defined by billing codes or use of unclear PH definition will be excluded. Covidence will be used for study selection and data extraction, which will be conducted independently and in duplicate by at least two researchers. Output variables include country, age, sex, race, cohort size, study design (retrospective/prospective), setting (urban/rural/both), location (hospital/community), random sampling (yes/no), definition of PH, studied PH risk cohort, socioeconomic‐index (high/middle/low), modality (echo/RHC), publication language, study quality (journal‐impact‐factor) and publication year. Findings from this study are positioned to advance PH as a major cause of global disease burden and in doing so identify new opportunities for international research that improve PH diagnosis, prognostication, and clinical pathways across different regions.

## A185 REPLACEMENT OF MICROVASCULAR BY MACROVASCULAR ENDOTHELIAL CELLS DRIVES DISTAL VESSEL MUSCULARIZATION IN PULMONARY HYPERTENSION

Qi Zhang^1^, Nobuhiro Yaoita^1^, Arata Tabuchi^1^, Shaofei Liu^1^, Shiau‐Haln Chen^2^, Qiuhua Liu^1^, Julie Rodor^2^, Sara Timm^3^, Niklas Hegemann^1,4,5^, Hebatullah Laban^5,6^, Toren Finkel^7^, Troy Stevens^8^, Diego F. Alvarez^9^, Lasti Erfinanda^1^, Marc de Perrot^10^, Mariya Kucherenko^1,4,5^, Christoph Knosalla^4,5^, Matthias Ochs^3,11^, Stefanie Dimmeler^5,12^, Thomas Korff^5,6,13^, Subodh Verma^14,15^, Andrew H. Baker^2^, Wolfgang M. Kuebler^1,5,15,16^

^1^Institute Of Physiology, Charité – Universitätsmedizin Berlin, Berlin, Germany, ^2^Centre for Cardiovascular Science, University of Edinburgh, Edinburgh, UK, ^3^Core Facility Electron Microscopy, Charité ‐ Universitätsmedizin, Berlin, Germany, ^4^Deutsches Herzzentrum der Charité (DHZC), Germany, ^5^Deutsches Zentrum für Herz‐Kreislauf‐Forschung e.V. (DZHK), Germany, ^6^Institute of Physiology and Pathophysiology, Department of Cardiovascular Physiology, Heidelberg University, Heidelberg, Germany, ^7^Department of Medicine, Division of Cardiology, University of Pittsburgh, Pittsburgh, USA, ^8^Department of Physiology and Cell Biology, College of Medicine, University of South Alabama, Mobile, USA, ^9^Department of Physiology and Pharmacology, College of Osteopathic Medicine, Sam Houston State University, Conroe, USA, ^10^Department of Surgery, Toronto General Hospital, University of Toronto, Toronto, Canada, ^11^Institute of Functional Anatomy, Charité ‐ Universitätsmedizin, Berlin, Germany, ^12^Institute for Cardiovascular Regeneration, Centre for Molecular Medicine, Goethe University, Frankfurt am Main, Germany, ^13^European Center for Angioscience (ECAS), Medical Faculty Mannheim, Heidelberg University, Heidelberg, Germany, ^14^Division of Cardiac Surgery, St. Michael's Hospital, University of Toronto, Toronto, Canada, ^15^Keenan Research Centre, St. Michael's Hospital, University of Toronto, Toronto, Canada, ^16^Departments of Surgery and Physiology, University of Toronto, Toronto, Canada

Endothelial cell (EC) apoptosis and proliferation of apoptosis‐resistant cells are hallmarks of pulmonary hypertension (PH). Yet, why some ECs die and others proliferate, and the relevance of this phenomenon for vascular remodeling is unclear. We hypothesized that this differential response may i) relate to different cell subsets, namely pulmonary artery (PAEC) versus microvascular endothelial cells (MVEC), ii) relate to differential EC responses to autophagic stress, and iii) cause replacement of MVEC by PAEC and subsequent muscularization in distal pulmonary arterioles. EC subset‐specific responses to chronic hypoxia were assessed by single‐cell RNA sequencing (scRNA‐seq) of murine lungs. Proliferative versus apoptotic responses to hypoxia, activation and role of autophagy were assessed in human and rat PAEC and MVEC, and in precision‐cut lung slices of wild type mice or mice with an endothelial‐specific deficiency in the autophagy gene Atg7 (Atg7EN‐KO). Abundance of PAEC versus MVEC in precapillary microvessels was assessed by the presence of Weibel‐Palade bodies (WPb; PAEC) versus staining for Griffonia simplicifolia (GS‐IB4; MVEC) in mouse lungs, and by staining for cytoplasmic tyrosine‐protein kinase BMX (BMX; PAEC) versus carbonic anhydrase 4 (CA4; MVEC) in human lungs, respectively. The role of endothelial autophagy in PH was tested in chronic hypoxic Atg7EN‐KO mice, and in sugen‐hypoxia (SuHx) rats treated with the ATG7 inhibitor ATG7‐IN‐2. Both in vitro and in vivo, PAEC and MVEC responded differentially to hypoxia with PAEC displaying markers of proliferation (Ki67, survivin, PCNA) and MVEC those of apoptosis (cleaved caspase 3, cleaved PARP1, annexin V), respectively. scRNA‐seq analyses support these findings in that hypoxia induced an antiapoptotic, proliferative phenotype in arterial EC, while capillary EC exhibited a propensity for cell death. In in vitro co‐culture experiments, these differential responses resulted in the progressive replacement of MVEC by PAEC. Similar replacement was evident in vivo as an increased abundance of PAEC and reduced number of MVEC in precapillary arterioles of chronic hypoxic mice, SuHx rats, and IPAH patients relative to appropriate controls. Comprehensive genomic and marker analyses did not reveal indications of MVEC transitioning to PAEC as an alternative scenario to replacement. Conditioned medium from hypoxic PAEC, yet not MVEC promoted pulmonary artery smooth muscle cell (PASMC) proliferation and migration in a platelet‐derived growth factor‐dependent manner. MVEC replacement by PAEC was prevented in hypoxic Atg7EN‐KO mice or ATG7‐IN‐2 treated SuHx rats, which showed normalization of distal vessel muscularization and PH. Autophagic activation by hypoxia induces in parallel PAEC proliferation and MVEC apoptosis. These differential responses cause a progressive replacement of MVEC by PAEC in precapillary pulmonary arterioles, thus providing a macrovascular context which in turn promotes PASMC proliferation and migration, ultimately driving distal vessel muscularization and the development of PH. Supported by the German Centre for Cardiovascular Research (DZHK), the German Research Foundation (DFG) in the framework of CRC TR84, subproject A04 and KU1218/12‐1, and the Chinese Scholarship Council (CSC).

## A186 SYNTHESIS OF CEO2@BSA NANOCLUSTERS FOR INHIBITION OF PULMONARY MICROVASCULAR ENDOTHELIAL CELL DYSFUNCTION AND IMPROVEMENT OF RAT VASCULAR RECONSTITUTION

Hui Zhao^1^, Wenhui Wu, Ping Yuan, Yuqing Miao

^1^School of Materials and Chemistry & Institute of Bismuth and Rhenium, University of Shanghai for Science and Technology, Yangpu district, Shanghai, China, ^2^Department of Cardio‐Pulmonary Circulation, Shanghai Pulmonary Hospital, School of Medicine, Tongji University, Yangpu district, Shanghai, China

Pulmonary microvascular endothelial cells (PMECs) dysfunction, the important pathophysiological feature of pulmonary arterial hypertension (PAH), can be caused by many harmful stimulating factors including increase in the level of reactive oxygen species (ROS). These pathological features offer tremendous promise for nanotechnology. Prepare a nano antioxidant for eliminating ROS. An ultra‐small CeO2@BSA nanocluster was synthesized by protein bionic method as an antioxidant. The ROS (H2O2, ·O2− and ·OH) scavenging ability of CeO2@BSA nanocluster was determined using kits. The effects of CeO2@BSA on cell proliferation, apoptosis, and migration were assessed using CCK‐8 assay, flow cytometry, and scratch test, respectively. Subsequently, SD rats modeled with monocrotaline were treated with CeO2@BSA, and the therapeutic efficacy was evaluated using echocardiography, right cardiac catheterization, and HE staining sections. The outstanding ROS scavenging ability and biocompatibility of the ultrasmall CeO2@BSA nanocluster were confirmed in PMECs under hypoxia. CeO2@BSA inhibited the proliferation and migration of PMECs and promoted apoptosis in hypoxia. The results of echocardiography, right cardiac catheter, and HE staining showed that CeO2@BSA had a significant therapeutic effect on PAH rats. The CeO2@BSA nanocluster could mitigate hypoxia‐induced PMECs dysfunction, thus demonstrating great potential for the treatment of PAH.

## A187 COMPREHENSIVE BIOINFORMATICS ANALYSIS OF THE CROSSTALK BETWEEN CHRONIC OBSTRUCTIVE PULMONARY DISEASE AND PULMONARY ARTERIAL HYPERTENSION

Yanghong Zheng^1^, Jie Zhou^2^, Zibire Fulati^3^, Jing Hua^1^, Xiangyu Chen^1^, Chunmei Feng^1^, Xia Fang^1^, Dong Liu^4^, Feilong Wang^1^, Qiang Li^1^, Na Wang^1^, Yingqun Ji^1^

^1^Department of Pulmonary and Critical Care Medicine, Shanghai East Hospital Affiliated to Tongji University, Tongji University School of Medicine, Pudong New Area, China, ^2^Department of Hematology, Tongji Hospital Affiliated to Tongji University, Tongji University School of Medicine, Putuo District, China, ^3^Department of Echocardiography, Shanghai Institute of Cardiovascular Diseases, Shanghai Institute of Medical Imaging, Zhongshan Hospital Affiliated to Fudan University, Xuhui District, China, ^4^Department of Pulmonary and Critical Care Medicine, Peking University China‐Japan Friendship School of Clinical Medicine, China

Pulmonary hypertension (PH) is classified into five distinct groups, and patients often exhibit overlapping symptoms arising from multiple comorbidities and mixed manifestations within these subgroups. This study aimed to reveal the common genetic interactions between chronic obstructive pulmonary disease (COPD, which can progress into group 2 PH) and pulmonary arterial hypertension (PAH) (group 1 PH), explore the regulatory network connecting these two PH subgroups and identify potential interventional targets for patients with PH who have overlapping pathogenesis. An analysis of Differentially Expressed Genes (DEGs) was conducted using datasets GSE76925 and GSE117261. Subsequently, Gene Ontology (GO) function enrichment analysis and Kyoto Encyclopedia of Genes and Genomes (KEGG) pathway analysis were performed based on the common DEGs shared between COPD and PAH. Furthermore, a Protein‐Protein Interaction (PPI) network was established. The weighted gene co‐expression network analysis (WGCNA) was applied to identify gene modules with high biological significance associated with both diseases. The overlapping genes between the commonly shared DEGs and the top three module eigengenes (ME) were extracted for further analysis. Next, a miRNA regulatory network with KEGG annotations and a transcription factor regulation network were established. Two validation datasets were used for the comparison study, and the between‐group t‐test was used to evaluate the reliability of the analysis results. In total, 311 commonly shared DEGs were identified. After taking intersection of DEGs and genes in WGCNA, nine genes were identified. In KEGG analysis of miRNA regulatory network, seven overlapped signaling pathways (HIF‐1 signaling, p53 signaling, PI3K‐Akt signaling, focal adhesion, neurotrophins signaling, cancer, and T cell receptor signaling) were identified. Nine genes, RPL35A, RPL15, STAT4, GZMK, CD40LG, CD5, GZMA, TRAT1, and BCL2, were commonly altered in both COPD and PAH, among which six and seven genes significantly differed in COPD and PAH validation cohorts respectively. Conclusion: Our study demonstrated the mutual molecular signaling pathways between COPD and PAH and illustrated the potential pathogenesis mechanism for overlapping PH subgroups.

## A188 BMP9 REGULATES ENDOTHELIAL EXPRESSION OF MEDIATORS OF PULMONARY VASCULAR REMODELING

Ying Zhong^1^, Luca Troncone^1^, Taylor Convington^1^, Peiran Yang^1^, Elizabeth Shin^1^, Lillian Worst^1^, Shreyas Rajesh^1^, Ana Zeghibe^1^, Megan McNeil^1^, Stephanie Kim^1^, Geoffrey Bocobo^1^, Robert Szulcek^2^, Harm Bogaard^3^, Paul Yu^1^

^1^Harvard Medical School, Massachusetts General Hospital, Boston, USA, ^2^Laboratory of in vitro modeling systems of pulmonary and thrombotic diseases, Institute of Physiology, Charité – Universitätsmedizin Berlin, corporate member of Freie Universität Berlin and Humboldt‐Universität zu Berlin, and German Center for Lung Research (DZL), and Deutsches Herzzentrum der Charité – Medical Heart Center of Charité and German Heart Institute Berlin, Department of Cardiac Anesthesiology and Intensive Care Medicine, Berlin, Germany, ^3^Pulmonary Medicine Division, Amsterdam University Medical Center, Amsterdam, Netherlands

Loss‐of‐function mutations in GDF2/BMP9 are found in heritable pulmonary arterial hypertension (PAH), suggesting a protective role, while protective or pathogenic effects are found in experimental PAH studies depending on context. The net contribution of BMP9 in PAH thus remains controversial. BMP9 regulates the secretome of pulmonary microvascular endothelial cells (PMVEC) to contribute to pulmonary artery smooth muscle cell (PASMC) phenotype modulation and pulmonary vascular remodeling. We tested the function of BMP9 in vivo using recombinant BMP9, BMP9/BMP10 trap ALK1‐Fc, and BMP9 neutralizing antibody before and after the development of pulmonary hypertension (PH) in distinct experimental models. We analyzed the impact of BMP9 in vitro on the PMVEC transcriptome, and its potential contribution to pulmonary vascular remodeling via intersection with human and experimental PAH lung transcriptomes, and tested the impact of BMP‐regulated EC‐derived genes on PASMC phenotype and growth. ALK1‐Fc and anti‐BMP9 prevented or reversed experimental PH administered before or after the onset of experimental PH, and attenuated medial hypertrophy and muscularization of small arterioles ( < 50 µm). Recombinant disulfide‐linked BMP9 was not protective in SUGEN/Hypoxia (SU‐Hx)‐ or monocrotaline (MCT)‐induced experimental PH, in contrast to previous studies using incompletely disulfide‐linked BMP9, suggesting dissociated monomers might function as competitive BMP9 antagonists. Consistent with this interpretation, BMP9 upregulated PMVEC genes associated with pulmonary vascular remodeling in experimental and human PAH including CXCL12, IGFBP4, ET‐1, VEGF‐A, and PDGF‐BB. Stimulation of PMVEC with BMP9 induced a contractile phenotype (CNN1; TAGLN) in co‐cultured PASMC. Administration of ALK1‐Fc or anti‐BMP9 attenuated the expression of several of these growth factors in SU‐Hx PH. In experimental PH ALK1‐Fc and ACTRIIA‐Fc but not anti‐BMP9 elicited peripheral edema, a phenotype associated with the use of ALK1‐Fc in human trials. In Sv129 mice prone to arteriovenous malformations (AVMs), ACTRIIB‐Fc and ALK1‐Fc elicited marked AVM phenotypes resembling phenotypes of hereditary hemorrhagic telangiectasia (HHT) syndrome, whereas anti‐BMP9 did not. BMP9 contributes to experimental PH by regulating the expression of EC‐derived molecules modulating PASMC growth and phenotype. In contrast to broad strategies that inhibit BMP9 and BMP10 associated with HHT mimicry in clinical trials and rodent PH models, treatment with anti‐BMP9 was well‐tolerated. Selective antagonism of BMP9 represents a potential therapeutic strategy for human PAH.

## A189 TRANSCRIPTOMIC ANALYSIS OF PERIPHERAL BLOOD IDENTIFIES SMOKING‐RELATED MOLECULAR ABNORMALITIES IN INTERSTITIAL LUNG DISEASE (ILD) AND ILD‐ASSOCIATED PULMONARY HYPERTENSION

Iryna Zhyvylo^1^, Namita Sood^1^, Janelle Vu Pugashetti^2^, Ssu‐Wei Hsu^1^, Justin Oldham^2^, Ching‐Hsien Chen^1^, Elena A. Goncharova^1^

^1^Division of Pulmonary, Critical Care and Sleep Medicine, Department of Internal Medicine, University of California, Davis, USA, ^2^Division of Pulmonary and Critical Care Medicine, Department of Internal Medicine, University of Michigan, Ann Arbor, USA

Pulmonary hypertension (PH) due to chronic lung disease and/or hypoxemia (Group 3 PH) is the second most common form of PH. PH is a frequent complication of interstitial lung diseases (ILD), including idiopathic pulmonary fibrosis (IPF), which worsens morbidity and mortality. Smoking has a detrimental effect on the transplant‐free survival of patients with progressive fibrosing ILD and IPF. Smoking‐related molecular alterations in patients with ILD and ILD‐PH are not well understood. The goal of this study was to characterize gene expression changes associated with smoking in patients with ILD‐PH. RNA sequencing analysis of peripheral whole blood samples from 226 patients with ILD (39.4% with PH; 59.3% smokers, 40.3% with IPF) was performed followed by DESeq2 analysis to identify differentially expressed genes (DEG) and pathway analysis. The study was conducted under the protocol approved by the University of California Davis Institutional Review Board. All samples and patient data for this study were obtained following written informed consent from study participants. Seven years follow‐up of 134 smokers and 92 non‐smokers with ILD demonstrated that transplant‐free survival was significantly higher in non‐smokers compared to smokers (*p* < 0.0353). Smokers with ILD‐PH (*n* = 55) had a trend of worsening transplant‐free survival compared to non‐smokers (*n* = 34). Observed difference, however, did not reach statistical significance. A median transplant‐free survival of smokers with ILD and ILD‐PH were 5.4 and 3.6 years, respectively. Transcriptomic analysis of ILD cohort identified 170 differentially expressed genes (DEGs) (FC > 1.5, *p* < 0.05), in smokers compared to non‐smokers (89 up‐ and 81 downregulated). Ten significantly up‐ and downregulated genes in smokers were [RASL12, SNORA59A, POLR1A, CATSPER2, FAM8A3P, NEO1, SLC9A8, OR4C11, RNF113B, RYBP] and [DNAJB2, IGHV3‐7, PCDHA1, IFNA6, IFNE, ZNF618, SMAP1, SPATA2, HNF1B, ACTL10], respectively. In the ILD‐PH group, 205 DEGs were identified, 82 of which were up‐ and 123 were downregulated in smokers compared to non‐smokers. Out of all identified DEGs, top 10 upregulated genes were ADAM28, AGAP4, AK4P4, ALPL, ANKRD36BP2, ARHGAP17, B3GNT2, BHLHE22, CCDC24, COLCA1, and top 10 downregulated genes were FPR3, GALNT11, EFCAB6, STK32B, HCRT, PLA2G2F, PTPN11, C1QTNF5, C1QTNF9, GPR173. A pilot comparison of smokers and non‐smokers with IPF‐PH (*n* = 24 and *n* = 9, respectively) identified 352 upregulated and 264 downregulated DEGs in smokers versus non‐smokers, which predominantly belonged to the metabolic pathways, profibrotic signaling, immune response, proliferation, and necroptosis. The top 10 upregulated genes were [TMTC1, HIST1H3G, TRIM28, SYNGR3, SCL25A6P3, CHTOP, TRIM35, TIMP1, HCRTR1, ABALON], and the most downregulated genes were [KCNMB4, GCNT1P5, C1QTNF5, C1QTNF9, PLA2G2F, KCTD16, PEX16, DNAJB2, SNX3P1Y, MPND]. The diffusing capacity for carbon monoxide (DLCO), which is an independent predictor of survival in PH, was significantly lower in smokers compared to non‐smokers with IPF‐PH (*p* = 0.0262), but not in other groups. Transcriptome analysis could be used to identify smoking‐associated DEGs in settings of ILD, ILD‐PH, and IPF‐PH. Further studies are needed to determine gene expression signatures of smokers with PH, evaluate potential correlation with PH severity, and determine potential target pathways for therapeutic intervention.

